# Access to C(sp^3^) borylated and silylated cyclic molecules: hydrogenation of corresponding arenes and heteroarenes†

**DOI:** 10.1039/d4ra00491d

**Published:** 2024-04-02

**Authors:** Arzoo Chhabra, Sabrina Reich, Timothy M. Shannon, Robert E. Maleczka, Milton R. Smith

**Affiliations:** a Department of Chemistry, Michigan State University 578 S Shaw Lane East Lansing Michigan 48824 USA maleczka@chemistry.msu.edu

## Abstract

This paper presents a simple and cost-effective hydrogenation method for synthesizing a myriad of cycloalkanes and saturated heterocycles bearing boryl or silyl substituents. The catalyst used are heterogeneous, readily available, bench stable, and recyclable. Also demonstrated is the application of the method to compounds that possess both boryl and silyl groups. When combined with Ir-catalyzed sp^2^ C–H borylation, such hydrogenations offer a two-step complementary alternative to direct sp^3^ C–H borylations that can suffer selectivity and reactivity issues. Of practical value to the community, complete stereochemical analyses of reported borylated compounds that were never fully characterized are reported herein.

## Abstract 2

Readily available and bench stable catalyst. Tolerates a variety of functional groups including orthogonal functionality. Circumvents the limitations of sp^3^ C–H borylation.

## Graphical abstract

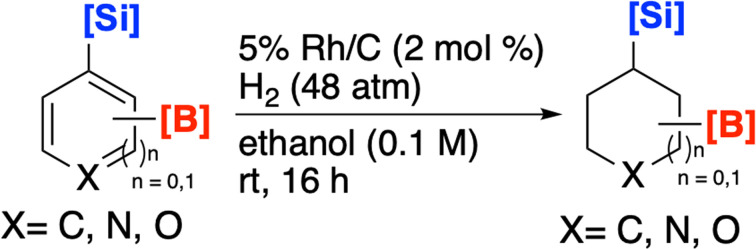

## Introduction

Organoboron compounds are often pivotal intermediates in the synthesis of natural products, biologically relevant molecules and compounds used in material science.^1^ Such broad applications come from the ability of C–B bonds to be readily transformed to C–OH, C–NH_2_, C–C bonds, *etc.* Additionally, boryl groups present on sp^3^ carbons can undergo couplings with retention or inversion of stereochemistry, Matteson homologation reactions, *etc.*^2^ Owing to their synthetic utility, the pursuit of new methods and strategies for making borylated compounds is an active area of research.

Among the various borylation methods, catalytic sp^2^ C–H borylations are widely used for the preparation of borylated arenes and heteroarenes. In contrast, catalytic sp^3^ C–H borylations to afford borylated cycloalkanes and saturated heterocycles are relatively underdeveloped. Many sp^3^ C–H borylation methods are highly substrate limited requiring the presence of a directing group (*e.g.* 2-alkyl pyridines, alkyl amines, benzylic compounds, cyclopropyl amides) and/or demand excess starting materials or reagents.^3–6^

**Fig. 1:**
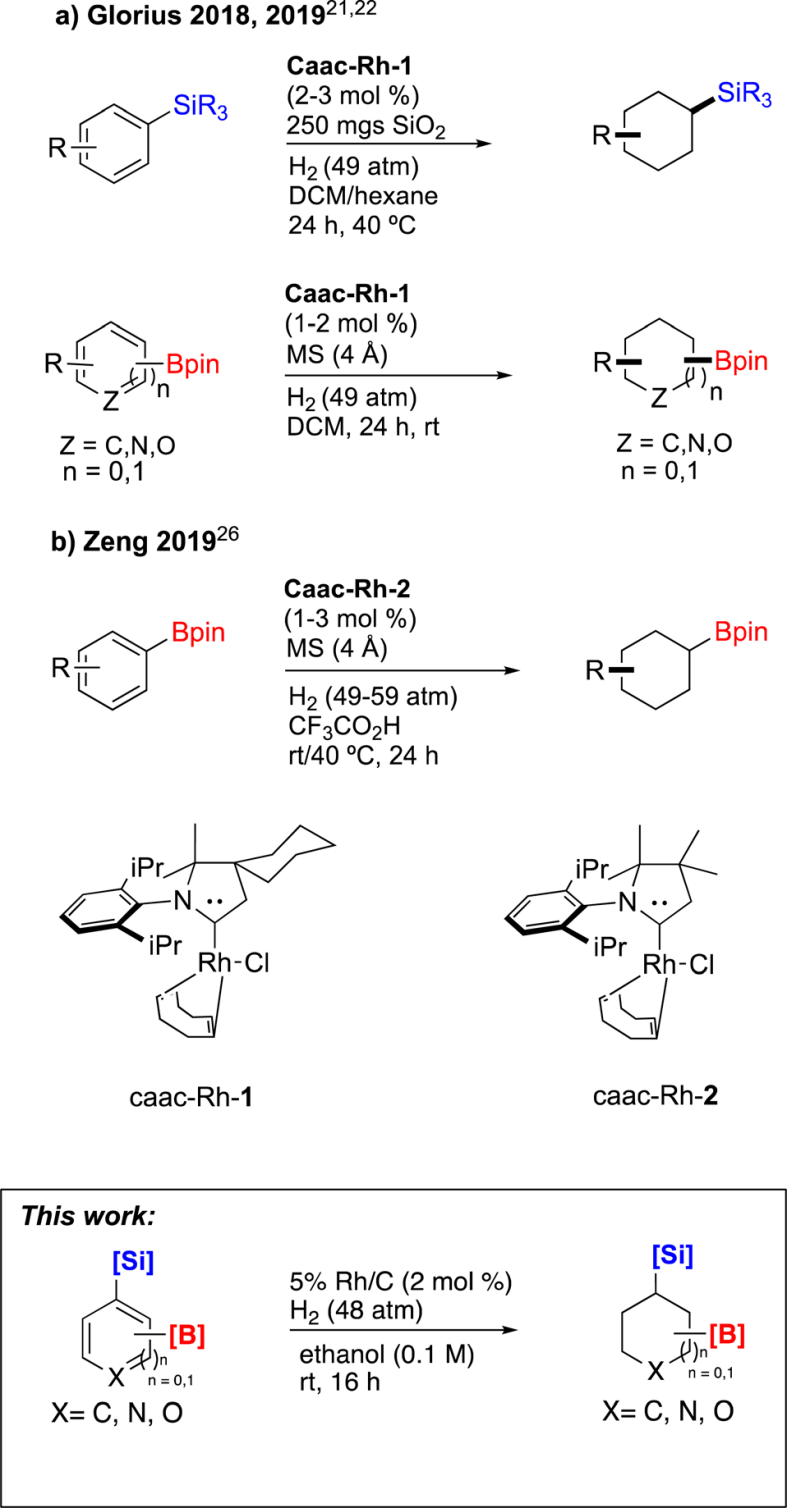
Strategy to access sp^3^ C–H borylated and silylated substrates *via* arene/heteroarene hydrogenation using Rh catalysts.

In recent years, some of these restraints have been eased. The Hartwig group has established directing group free sp^3^ C–H borylation chemistry that encompasses a broader substrate scope, including alkanes, ethers, protected amines, alcohols, and carbocycles. Their method affords good selectivity and when executed with an excess of boron reagent good reactivity. In addition, all reported examples were run in cyclooctane. As the authors note, this restricted functionalization of polar molecules due to poor solubility.^7^ Schley and coworkers were able to overcome the limitation of excess of boron reagent through the thoughtful application of dipyridylarylmethane as ligand.^8^ In an alternative strategy, sp^3^ C–H borylated compounds can be accessed by hydrogenating corresponding borylated arenes, which themselves can be obtained *via* iridium catalyzed sp^2^ C–H borylations. Hydrogenation of arenes and heteroarenes have been carried out in the past *via* transition metal (Pd, Ru, Pt and Rh) catalysis.^9–20^ However, none of these reports demonstrated the hydrogenation on borylated or silylated arenes or heteroarenes. On this front, Glorius and coworkers pioneered the catalytic heterogeneous hydrogenation of boryl- and silyl-arenes/heteroarenes by a cyclic (alkyl)(amino)carbene rhodium complexes (Fig. 1).^21–25^ In similar work, Zeng showed hydrogenation of borylated arenes and heteroarenes using a related Rh-catalyst (Fig. 1).^26^ Glorius' catalyst was utilized by Bach's group to hydrogenate one borylated 2-oxindole among other benzofused N-heterocycles, 2,5-diketopiperazine, and 3,4-dihydroquinolones.^27–29^

Glorius also reported hydrogenations using Rh/C in their optimization studies on six borylated substrates (five benzenes and one pyridine).^21^ Catalysis with Rh/C showed full conversion with TBS protected 4-Bpin phenol, but as the authors noted the other arenes tested and the pyridine gave diminished yields (17–33%). Separately,^10^ Rh/C catalyzed hydrogenation of six silylated benzenes were examined. It was reported that two of the six gave 0% of the corresponding cyclohexanes, but three substrates were saturated in 25, 35, and 40% yields respectively. In contrast, the hydrogenation of *n*-hexyl ether of 4-TMS-phenol was achieved in 90% yield with Rh/C in hexane (*vs.* 16% in EtOH) and could be further optimized up to 97% using Rh/Al_2_O_3_.

Other recent reports on saturating arenes and heteroarenes that do not bear boryl or silyl substituents include the work of Handa, who used [Ir(cod)Cl]_2_ to hydrogenate phosphine oxide scaffolds with Ir nanoparticles being the active catalyst.^30^ A cooperative heterogeneous and homogeneous strategy for asymmetric hydrogenations of arenes and heteroarenes has also been developed, which has been applied to a single borylated substrate (benzofuran).^31–33^ The hydrogenation of fluoropyridines using Pd(OH)_2_, in acidic media has also been reported.^34^

While we considered all prior art cited above, Glorius' results motivated us to fully evaluate bench stable and commercially available Rh-catalysts, *e.g.* Rh/C or Rh/Al_2_O_3_, or other standard hydrogenation catalysts against a larger substrate set of arenes and heteroarenes with boryl or silyl substituents. We were further inspired by Glorius' recent report on hydrogenation of arenes that were bisfunctionalized with germyl and boryl, and germyl and silyl,^35^ and sought to hydrogenate previously unexplored heteroarenes bearing both boryl and silyl substituents.

## Results and discussion

As the catalytic hydrogenations of pyridines using Rh/C is well established,^36^ we began our study by subjecting an ethanolic mixture of 3-borylated pyridine 1a to 5% Rh/C under a hydrogen atmosphere (Table 1; entry 1). As piperdines are known to poison Rh-catalysis^36,37^ and since pyridinium salts hydrogenate more readily than the free base,^38^ HCl was added to the reaction mixture. After 1 h, the pyridine ring was fully saturated to afford the desired borylated piperidine (2a) along with the deboronated product (3a). Rhodium on alumina (entry 2) afforded a similar mix of 2a and 3a, but the reaction was incomplete after 2 hours. Though platinum oxide has long been used to hydrogenate pyridines, reactions catalyzed by Pt_2_O and Pt/C met with an increased amount of deboronation (entries 3 and 4).

**Table 1:** Hydrogenation catalysts screening^a^

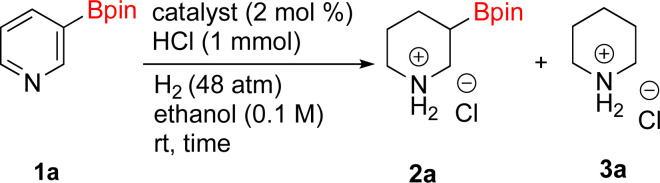						
Entry	Starting material	Product 2a	Product 3a	Catalyst	Ratio^b^ of 2a : 3a : 1a	Time
1	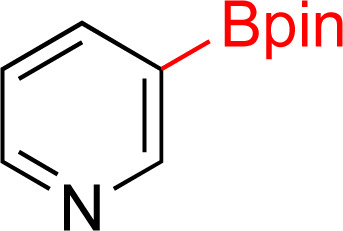	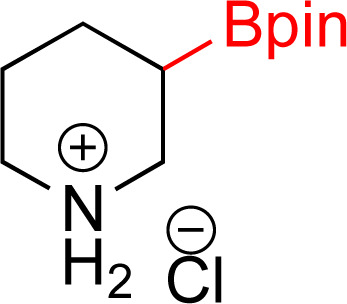	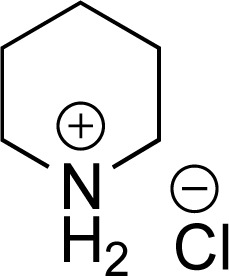	5% Rh/C (2 mol%)	72 : 28 : 0	1 h
2	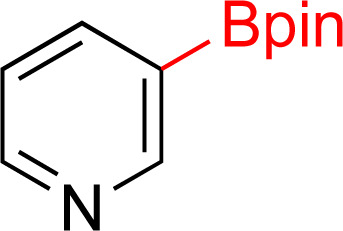	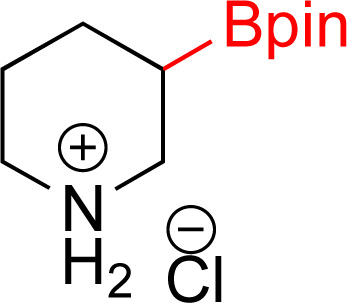	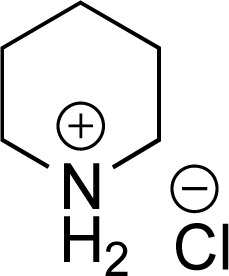	5% Rh/Al_2_O_3_ (2 mol%)	50 : 15 : 35	2 h
3	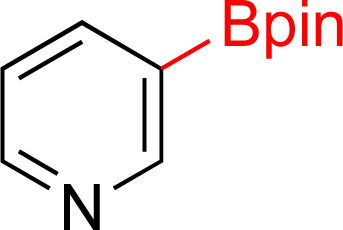	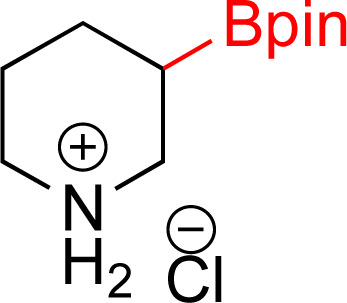	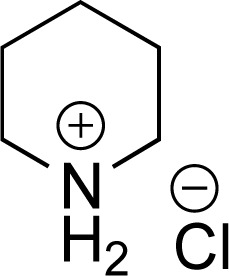	PtO_2_ (2 mol%)	32 : 32 : 36	2 h
4	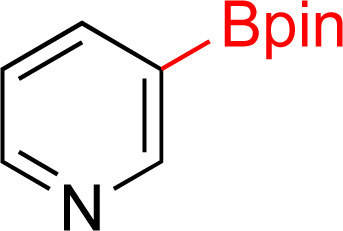	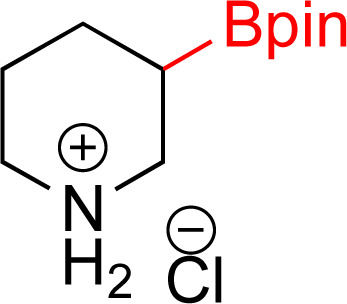	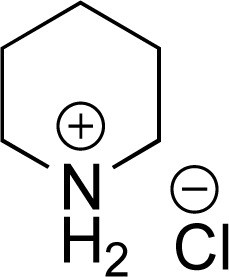	10% Pt/C (2 mol%)	24 : 38 : 38	2 h
5	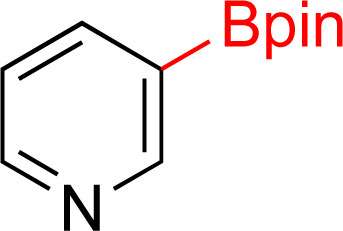	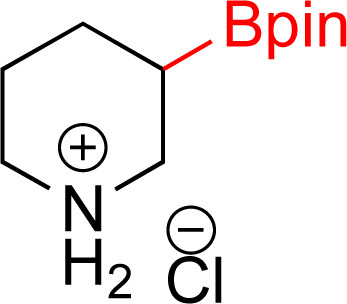	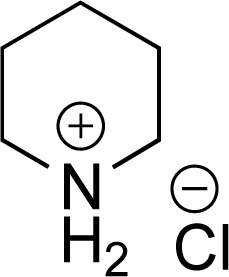	10% Pd/C (2 mol%)	0 : 0 : 100	2 h

a (4,4,5,5-Tetramethyl-1,3,2-dioxaborolan-2-yl)pyridine (0.25 mmol), hydrochloric acid (1 mmol), hydrogen gas (48 atm), ethanol (2 mL), 5% Rh/C (10 mg).

b Relative composition based on ^1^H-NMR.

Catalytic hydrogenations of pyridines with palladium typically demand higher temperatures (70–80 °C) and higher catalyst loads,^39^ so it was not entirely surprising that 10% Pd/C failed to effect hydrogenation (entry 5). W2 RANEY® nickel^40^ and Ru-catalysts^41^ were not tested given the precedent for hydrogenations of pyridines with those catalysts requiring pressures > 1000 psi.

With Rh/C proving fastest at hydrogenating 1a, reactions with this catalyst were screened against different solvents (Table 2). In addition to ethanol, 1a could be hydrogenated in methanol, dichloromethane, dioxane, and THF. Yields of 2a were observed to be in order of ethanol/methanol > THF > dioxane > dichloromethane. This trend is consistent with previous reports on hydrogenations of non-polar substrates in polar solvents, where it has been shown that higher yields correlate to higher activity coefficients.^42^ Methanol was chosen as the reaction solvent because of the greenness of the solvent.^43^ Unfortunately, with all solvents deboronation remained a problem, with 3a being the only observed product when the reaction was run in dichloromethane for 36 hours. As metal mediated protiodeboronation by Brønsted acids and Lewis acids is well known,^44^ the reaction was run without HCl (Table 2; entry 2). This led to no reaction. Reducing the amount of conc. HCl in the reaction or using dry HCl (Table S1†) resulted in similar 2a : 3a ratios as those observed in entry 1, Table 1. Other Brønsted and Lewis acids were also tested (Tables S1 and S2†), but none solved the deboronation problem. Lastly, we explored the idea that by starting with a halogenated 3-borylpyridine the reaction conditions would *in situ* generate 1 equiv. of HX, which would immediately form the pyridinium and not promote deboronation. Thus 3-bromo-5-(4,4,5,5-tetramethyl-1,3,2-dioxaborolan-2-yl)pyridine (2b) was hydrogenated under the standard, albeit HCl free, conditions. Though debromination and hydrogenation occurred as predicted, deboronation was not eliminated. Unable to eliminate deboronation, we reevaluated the early catalyst screening data. Catalysis using Rh/Al_2_O_3_ (entry 2, Table 1) was slower than Rh/C, but afforded a better ratio of 2a to 3a. Therefore, as we looked at the hydrogenation of additional borylated pyridines, Rh/Al_2_O_3_ was employed as catalyst and reactions were run for 16 h. As shown in Table 3 (entries 2–5) 2c, 2d, 2e, and 2f were all formed >80% yield with no deboronation. Of note was the hydrogenation of 1f, which was carried out without the addition or *in situ* generation of a Brønsted acid. Presumably, the methyl groups at C2 and C6 inhibit poisoning of the catalyst. Furthermore, 2f was formed as single diastereomer, the stereochemistry of which as determined to be all *cis* by oxidizing the boronate ester to a hydroxy group and comparing that product to analogous literature compounds.^45,46^

**Table 2:** Solvent screen for hydrogenation^a^

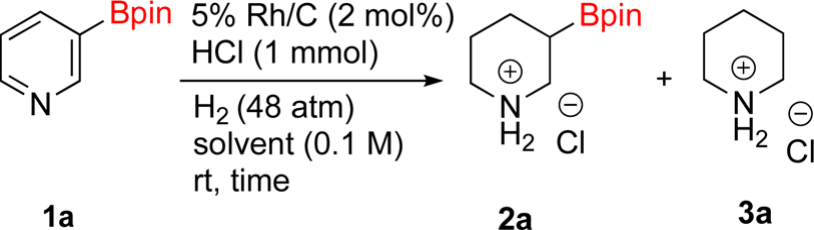					
Entry	Solvent	Time (h)	2a^b^ (%)	3a^b^ (%)	1a^b^
1	EtOH	16	75	25	0
2	EtOH^c^	2	0	0	100
2	DCM	36	0	100	0
3	Dioxane	7	47	53	0
4	THF	7	65	35	0
5	MeOH	2	75	25	0

a (4,4,5,5-Tetramethyl-1,3,2-dioxaborolan-2-yl)pyridine (0.25 mmol), HCl (1 mmol), H_2_ (48 atm), solvent (2 mL), 5% Rh/C (2 mol%), rt.

b Relative composition determined by ^1^H-NMR.

c Run without HCl.

**Table 3:** Hydrogenation of pyridines^a^

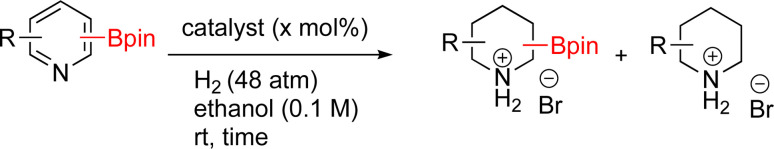				
Entry	Starting material	Products (yield%)	Catalyst	Time
1	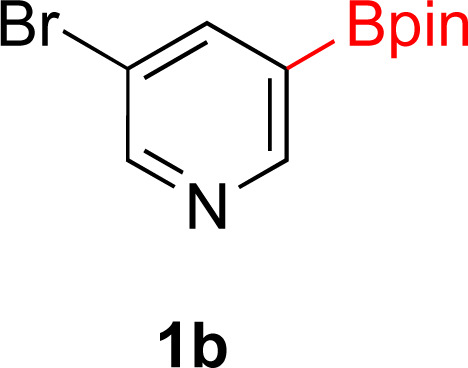	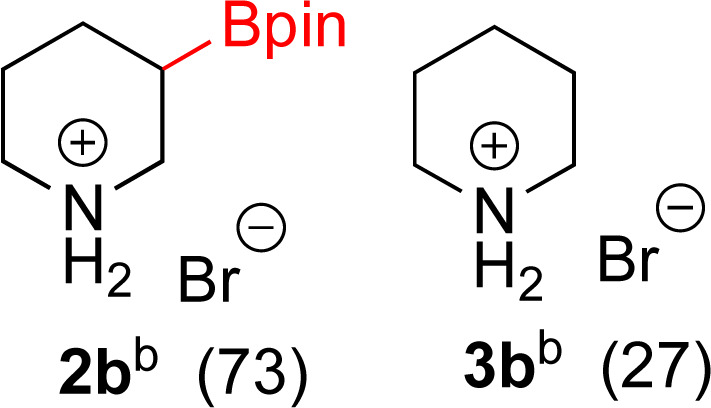	5% Rh/C (2 mol%)	16 h
2	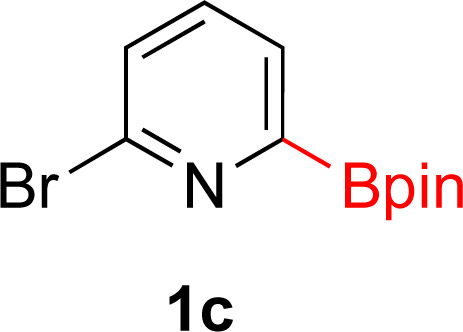	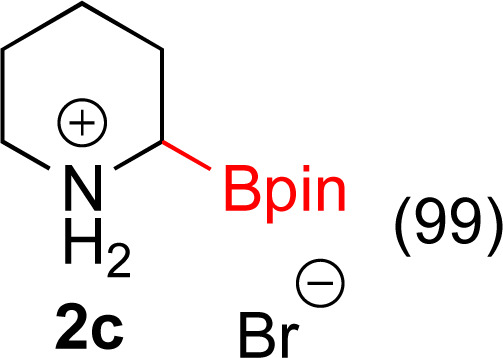	5% Rh/C (2 mol%)	16 h
3	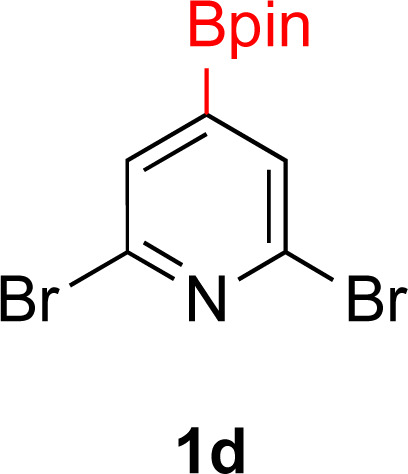	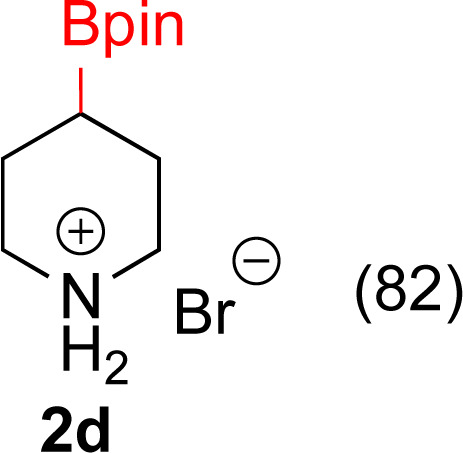	5% Rh/Al_2_O_3_ (8 mol%)	16 h
4^c^	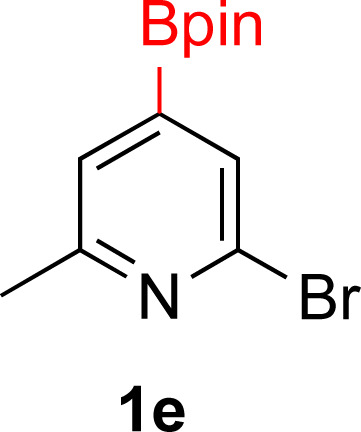	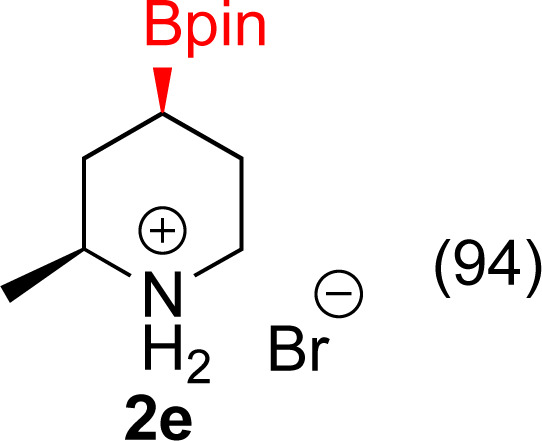	5% Rh/Al_2_O_3_ (4 mol%)	16 h
5	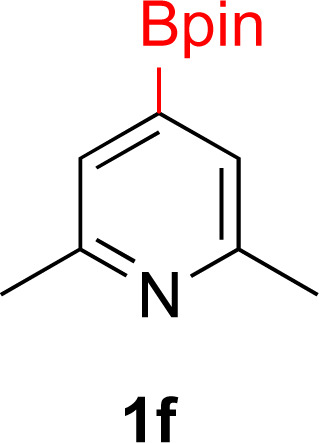	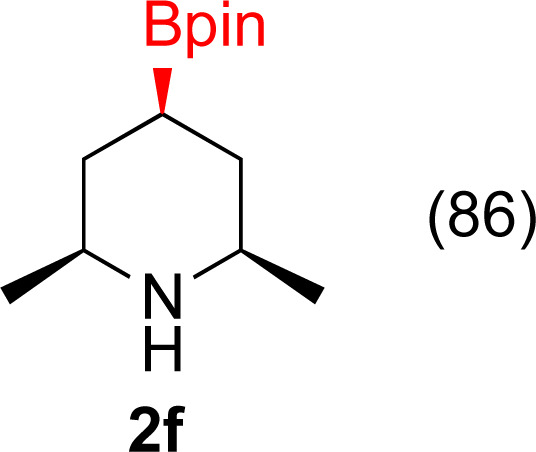	5% Rh/Al_2_O_3_ (4 mol%)	40 h

a Isolated yields and relative stereochemistry shown, starting material (0.5 mmol), ethanol (5 mL).

b Isolated as a mixture.

c Run at 0.3 mmol scale.

**Scheme 1:**
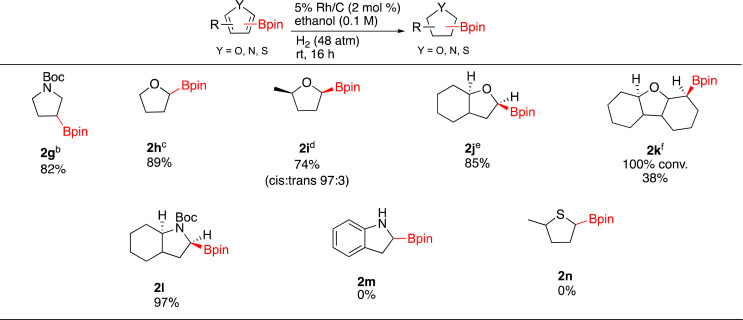
Hydrogenation of borylated heteroarenes^*a*^. ^*a*^Reaction conditions: 0.5 mmol substrate, relative stereochemistry shown, isolated yields shown. ^*b*^*N*-Boc-3-3-(4,4,5,5-tetramethyl-1,3,2-dioxaborolan-2-yl)-pyrrole (1.0 g, 3.4 mmol), Rh/C (170 mg, 2 mol%) EtOH (10 mL, 0.3 M). ^*c*^13 h, 8% OBpin monomer, ^*d*^1 mmol substrate, Rh/C (1 mol%), 6.3% HOBpin byproduct ^*e*^solvent = hexane, *P* = 54 atm, ∼20% OBpin monomer ^*f*^8% deborylated product, solvent = hexane, *P* = 59 atm, *T* = 60 °C, *t* = 21 h.

Hydrogenation of different 5 membered borylated heterocycles were then investigated (Scheme 1). Under Rh/C catalysis, *N*-Boc-3-(4,4,5,5-tetramethyl-1,3,2-dioxaborolan-2-yl)-pyrrole (1g) was successfully hydrogenated (73% yield) on 1 gram scale without any observable loss of the Bpin. As Pd/C has been shown to selectively hydrogenate the pyrrole ring on nicotyrine,^47^ the catalytic hydrogenation of 1g was also run with 10% Pd/C and 2g was afforded in 82% yield. We also attempted a one pot Ir-catalyzed borylation/hydrogenation sequence using Boc-pyrrole as the starting substrate. In practice, running the hydrogenation step on the crude borylation mixture was not successful as only 1g was observed. Nonetheless, it should be noted that the CH borylation of Boc-pyrrole gives 1g in 90% yield. Thus, the 74% two-step combined yield of 2g from Boc-pyrrole compares favorably to 54% yield obtained in the Ir-catalyzed sp^3^ C–H borylation of Boc-pyrrolidine.^7^

Moving on to other heterocycles, hydrogenation of 2-borylated furan (1h) occurred with 100% conversion to 2h. Isolation of 2h (89%) was complicated by the presence of HOBpin or oligomers thereof (∼8%). In this case, Rh/C is not as efficient as the Glorius catalyst, which affords 2h from 1h in 98% yield.^21,26^ It is worth nothing that when hydrogenating 1h at 2.5 mmol scale the catalyst could be recycled five times with reproducible results after each cycle. Direct sp^3^ Ir-catalyzed CH borylations of tetrahydrofuran gives the C-3 borylated product,^7^ in contrast, the sp^2^ Ir-catalyzed CH borylation of furan gives the C-2 borylated product. This highlights the potential for direct borylation and borylation/hydrogenation approaches to be complementary.

Hydrogenations of methyl substituted 1i, benzofuran 1j and dibenzofuran 1k were then carried out. Compound 2i was obtained in 74% yield as a 97 : 3 *cis*/*trans* mixture and as before a minor amount of boron byproduct (∼6%) was present in the isolated material. The *cis* stereochemistry of the major product was ascertained by COSY and 1D NOE NMR. Interestingly, hydrogenation of 1j generated the fully saturated borylated octahydrobenzofuran 2j again contaminated by the boron byproduct (78 : 22 by NMR). Compound 1k underwent full conversion affording 2k with 8% of the deboronated product. Though a minor product, the presence of the deboronated material made purification of 2k challenging and thus pristine 2k was isolated in only 38% yield. We were also able to generate 2l as an octahydroindole in 97% yield. The stereochemistry of hydrogen α to the nitrogen was found to be *cis* with the bridgehead hydrogen by 1D-NOE experiments. An unprotected borylated indole and a 2-borylated methylthiophene failed to produce saturated products 2m and 2n respectively, only starting material observed by ^1^H-NMR in both cases.

**Scheme 2:**
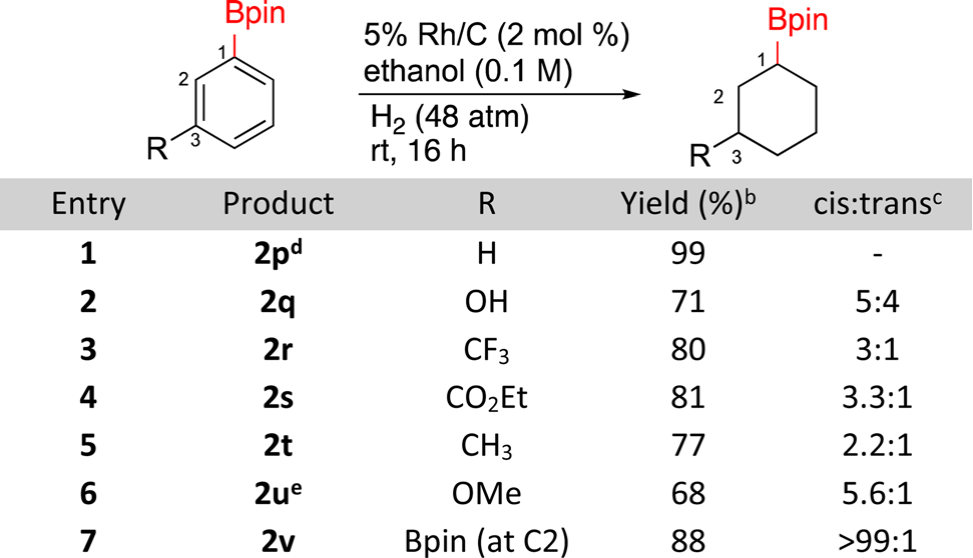
Hydrogenation of borylated benzenes^*a*^. ^*a*^Run on 0.5 mmol substrate. ^*b*^Isolated yields. ^*c*^*Cis*/*trans* ratios determined by ^1^H NMR on the crude reaction product. ^*d*^1 mmol substrate, Rh/C (1 mol%). ^*e*^22% demethoxylated byproduct.

The hydrogenation of borylated arenes was also carried out (Scheme 2). All borylated arenes tested were easily hydrogenated with Rh/C and functional groups like esters, methoxy, alkyl, trifluoroalkane and alcohols were well tolerated. For compounds 2q, 2r, 2s, 2t, and 2u low ratios of *cis* and *trans* products were obtained with the *cis* diastereomers being major. The assignment for 2q was made by comparisons to a known silylated derivative.^48^ To do so for compounds 2r, 2t, and 2u, each of these products were oxidized to their alcohols,^49^ which were then compared to previously reported *cis* and *trans* alcohols. It is worth noting that when 2u was generated using caac-Rh-2 as the catalyst, a yield of 55% yield was observed *vs.* the 68% with Rh/C. Diastereoselectivity was similar in both cases (∼6 : 1).^26^ The major stereoisomer of 2s being *cis* was confirmed using 1D-NOE. The major stereochemistry of bisborylcyclohexane 2v was made by direct comparison to literature data.^50^

**Scheme 3:**
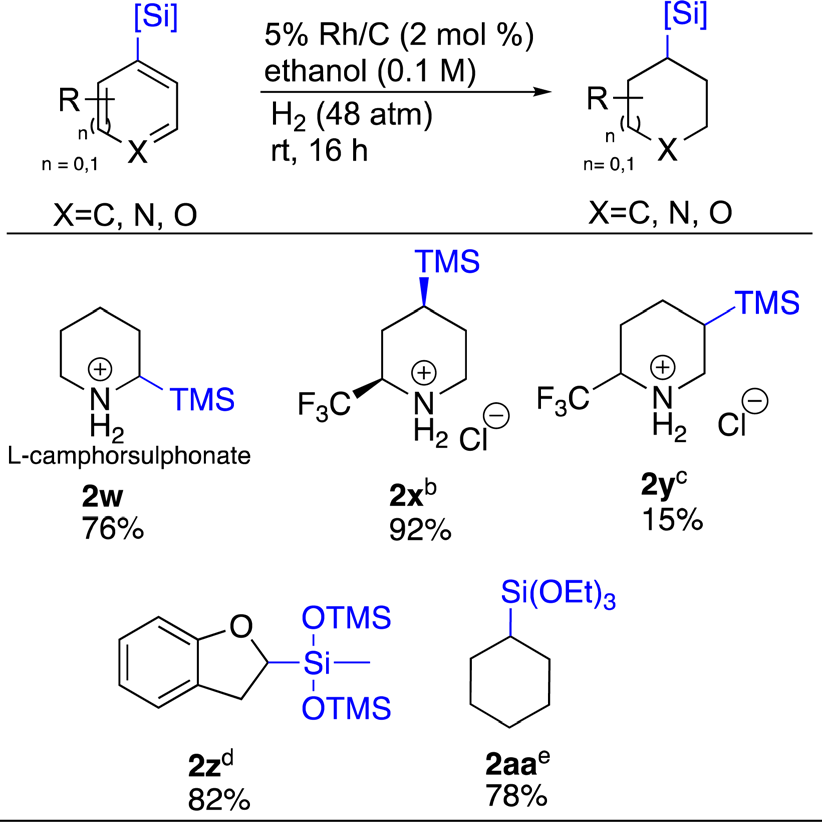
Hydrogenation of silylated arenes^*a*^. ^*a*^Reaction conditions: heterocycle (0.5 mmol), relative stereochemistry shown, all yields are isolated. ^*b*^Rh/Al_2_O_3_ (4.5 mol%), starting pyridine (0.2 mmol). ^*c*^Desilylated product observed. Ratio of desilylated : silylated = 5 : 1. ^*d*^Pd/C (10 mol%). ^*e*^Rh/Al_2_O_3_ (2 mol%).

Carbon–silicon bonds, similar to carbon–boron bonds, are also versatile as they can be transformed to various functional groups such as hydroxy, amine, halogen and aryl.^51^ Thus, we sought to apply the same chemistry to organosilicon bearing heterocycles (Scheme 3). TMS-substituted pyridines 2-TMS 1w and 4-TMS 1x were easily hydrogenated with Rh/C and Rh/Al_2_O_3_ respectively, demonstrating the utility of both these catalysts for such substrates. Compound 1w was hydrogenated as a salt of camphor sulphonate. This was to see whether adding an optically active acid could induce chirality in the hydrogenated product. This proved not to be the case as a 1 : 1 mixture of enantiomers was obtained. Surprisingly, 1y, which only differs from 1x by the position of the TMS group gave 2y as a 1 : 5 mixture with the desilylated material being major as determined by LCMS and ^19^F-NMR. Two siloxanes, 1z and 1aa were also tested. Compound 1z presented the opportunity to probe complementary reactivity beyond just the ability to saturate siloxane containing hetero arenes.

As Rh/C led to the saturation of both rings of 1j, for 1z we employed Pd/C to see if 2,3-dihydrobenzofuran 2z could be formed selectively.^25,52,53^ We were delighted to see this hypothesis realized. Catalytic hydrogenation of arene 1aa showcased how catalyst choice matters. For this substrate, Pd/C only returned starting material. Rh/C saturated the ring, but the siloxane was lost. In contrast, Rh/Al_2_O_3_ successfully provided 2aa in 78% yield.^54^

Dual functionalized compounds that incorporate both silyl and boryl group have shown potential in facilitating diverse reaction pathways due to their orthogonal reactivity. An example of such reactivity was demonstrated by Hartwig where Bpin was converted to a Boc protected amine without compromising silyl groups. In another example, the silyl group was manipulated without compromising the Bpin group.^55^

**Scheme 4:**
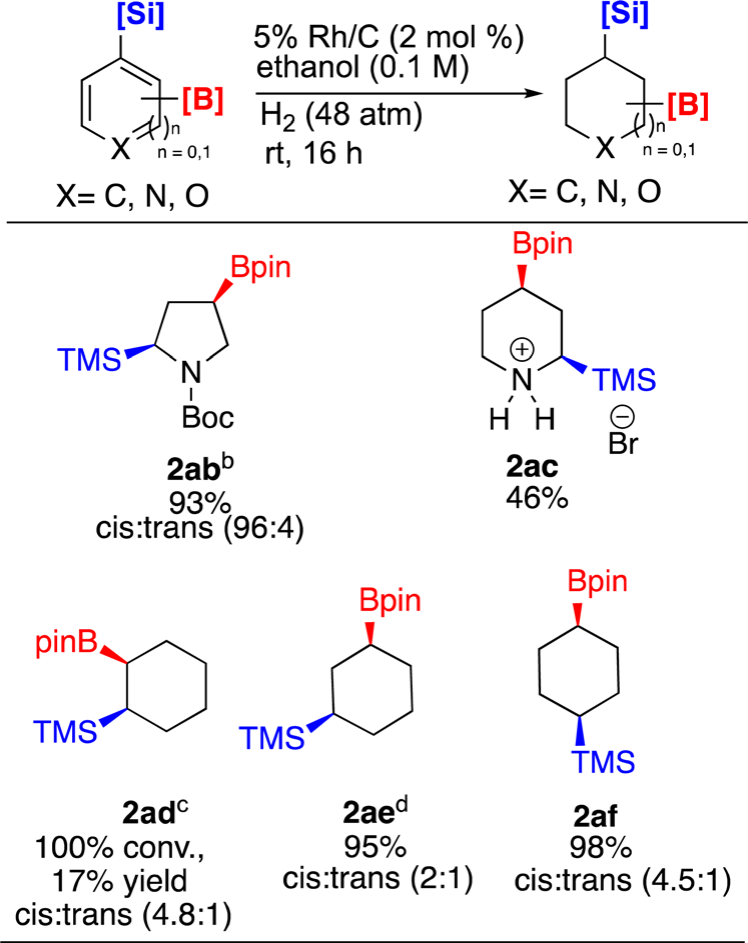
Hydrogenation of borylated and silylated arenes and heteroarenes^*a*^. ^*a*^Reaction conditions: substrate (0.5 mmol), relative stereochemistry shown, all yields are isolated. ^*b*^*t* = 48 h, Rh/C (4 mol%). ^*c*^12.5% desilylation observed. ^*d*^93 : 7 (product : desilylated product ratio) observed.

Given such demonstrations of Si/B dual functionality, we prepared and then hydrogenated 1ab–1af under Rh/C catalysis (Scheme 4). To drive the hydrogenation of pyrrole 1ab to full conversion, increasing the reaction time to 48 h and doubling the catalyst loading to 4 mol% was required. This enabled isolation of 2ab in 93% yield with a high *cis* : *trans* ratio (96 : 4). The *cis* configuration was confirmed by NOE and 2D NMR experiments. Hydrogenation of 2-bromo-4-(Bpin)-6-(TMS)pyridine 1ac was also carried out. As seen earlier, ring saturation was accompanied by debromination affording 2ac as its HBr salt. Compound 2ac was isolated in 46% yield by precipitating out the product using ethyl acetate. The 100% *cis* stereochemistry was determined by 1D-NOE.

Lastly, we examined the three substituted benzenes bearing boryl and silyl group in *ortho*, *meta*, and *para* arrangements. All three converted to the corresponding cyclohexanes, albeit with some differences. The reaction to produce *ortho*2ad showed 100% conversion to product (4.8 : 1 *cis* : *trans*) by ^1^H-NMR, however ∼12.5% of the material had desilylated. Difficulty in isolating 2ad by flash column chromatography was experienced. In contrast, 2ae (2 : 1 *cis* : *trans*) was isolated in higher yield, but some desilylation still occurred. As the amount of desilylation when forming 2ae was lower than that experienced with 2ad, we experimented to see if this side reaction could be eliminated. Unfortunately, we were unable to lessen desilylation by reducing the reaction time from 16 h to 8.5 h. Interestingly compound 2af (4.5 : 1 *cis*:*trans*) was isolated in 98% yield with no desilylation. We note the observed diastereoselectivities of 2ae and 2af were lower (2 : 1 *vs.* 4 : 1 for 2ae) and (4.5 : 1 *vs.* 7 : 1 for 2af) than those observed with Glorius' catalyst,^22^ whereas 2ad was formed with a comparable *cis* : *trans* ratio. We also synthesized *tert*-butyl 2-(4,4,5,5-tetramethyl-1,3,2-dioxaborolan-2-yl)-5-(trimethylsilyl)-1*H*-pyrrole-1-carboxylate and trimethyl(6-(4,4,5,5-tetramethyl-1,3,2-dioxaborolan-2-yl)dibenzo[*b*,*d*]furan-4-yl)silane compounds of dual functionality however their hydrogenation reactions under several different reaction conditions did not yield the desired hydrogenated products. They gave largely a mixture of deboronation and desilylation side products.

## Conclusion

In summary, Rh/C, Rh/Al_2_O_3_, and other readily available catalysts can affect the hydrogenation of borylated and silylated arenes and heteroarenes. Demonstrations of these catalysts to substrates bearing both boryl and silyl group were also shown to be viable. Catalyst selection is often key to successful hydrogenations, especially for 5- and 6-membered heterocycles. In some cases, minimizing unwanted loss of the boron or silicon substituent can also be achieved through catalyst choice. The scope, including limitations disclosed, and comparisons made to the pioneering work of Glorius and Zheng can help guide practitioners as to whether to employ more complex Rh-catalysts or those tested herein. Spectroscopic data reported herein for new molecules as well as previously described compounds where full characterization data were lacking may also prove valuable for those who find those compounds of interest.

## Experimentals

### General remarks

Unless indicated otherwise all reactions were carried out in oven-dried glassware with magnetic stirring and monitored by GC-MS or ^1^H-NMR/^19^F-NMR. Tetrahydrofuran was freshly distilled from sodium/benzophenone under nitrogen. Diisopropylamine was freshly distilled from calcium hydride. *n*-BuLi was used as a 2.5 M solution in hexanes. Flash column chromatography was performed with silica gel (230–400 mesh). Spectra taken in CDCl_3_ were referenced to 7.26 ppm in ^1^H NMR and 77.2 ppm in ^13^C{^1^H} NMR, C_6_D_6_ was referenced to 7.16 ppm in ^1^H NMR and 128.4 ppm in ^13^C{^1^H} NMR, C_7_D_8_ was referenced to 7.17 ppm in ^1^H NMR and 128.9 ppm in ^13^C{^1^H} NMR, CD_2_Cl_2_ was referenced to 5.30 ppm in ^1^H NMR and 53.5 ppm in ^13^C{^1^H} NMR. Resonances for the boron-bearing carbon atom were not observed due to quadrupolar relaxation. All coupling constants are apparent *J* values measured at the indicated field strengths in Hertz (s = singlet, d = doublet, t = triplet, q = quartet, dd = doublet of doublets, ddd = doublet of doublet of doublets, bs = broad singlet).

High-resolution mass spectra (HRMS) were obtained at the Michigan State University Mass Spectrometry Service Center using electrospray ionization (ESI+ or ESI−) on quadrupole time-of-flight (Q-TOF) instruments. Low resolution mass spectra were obtained on GCMS-QP2010 SE Shimadzu instrument. Melting points were measured in a capillary melting point apparatus and are uncorrected.

### General procedure A for synthesis of starting materials *via* iridium catalysis (1d–1g and 1z)

In a nitrogen filled glove box in a vial (5 mL)/round bottom (50 mL) loaded with a stir bar was added bis(1,5-cyclooctadiene)di-μ-methoxydiiridium(i), bis(pinacolato)diboron or bis(trimethylsiloxy)methylsilane, di-*tert*-butylbipyridine followed by addition of substrate in THF. The vial/round bottom was closed, removed from the glove box, connected to a Schlenk line, and placed in an oil bath. The solution was stirred under nitrogen at 70–80 °C for 16–48 h. The reaction mixture was concentrated by rotary evaporation and purified by passing through a silica plug or flash column chromatography.

#### 2,6-Dibromo-4-(4,4,5,5-tetramethyl-1,3,2-dioxaborolan-2-yl)pyridine (1d)

A modified general procedure A^56^ was followed with [IrOMe(cod)]_2_ (15.5 mg, 0.02 mmol, 0.25 mol%), dtbpy (12.5 mg, 0.04 mmol, 0.5 mol%), B_2_pin_2_ (2.13 g, 9.3 mmol) and THF (2 mL). To this solution was added 2,6-dibromopyridine (1.37 g, 9.3 mmol) and the resulting solution was stirred at 80 °C for 48 h. Upon completion, the reaction mixture was concentrated by rotary evaporation and the residue purified by silica gel chromatography (1% MeOH/DCM). Fractions containing the desired product were combined and the volatiles evaporated to afford a white solid. (1.19 g, 57% yield). ^1^H NMR (500 MHz, CDCl_3_) *δ* 7.76 (s, 2H), 1.33 (s, 12H). ^13^C{^1^H} NMR (126 MHz, CDCl_3_) *δ* 140.9, 132.0, 85.4, 25.2. ^11^B NMR (160 MHz, CDCl_3_) *δ* 29.5. NMR data matched those reported in the literature.^56^

#### 2-Bromo-6-methyl-4-(4,4,5,5-tetramethyl-1,3,2-dioxaborolan-2-yl)pyridine^57^ (1e)

General procedure A was followed with bis(1,5-cyclooctadiene)di-μ-methoxydiiridium(i) (27 mg, 0.04 mmol, 0.4 mol%), dtbpy (24 mg, 0.09 mmol, 0.9 mol%), pinacol borane (1.4 g, 11 mmol, 1.1 equiv.), THF (6 mL) and 2-bromo-6-methylpyridine (1.7 g, 10 mmol). The solution was stirred under nitrogen at 70 °C for 24 h. Upon completion, the reaction mixture was concentrated by rotary evaporation and the residue purified by silica gel chromatography (10 : 90 EtOAc/hexane). Fractions containing the desired product were combined and the volatiles evaporated to afford a white solid (2.5 g, 86% yield). ^1^H NMR (500 MHz, CDCl_3_) *δ* 7.62 (s, 1H), 7.43 (s, 1H), 2.52 (s, 3H), 1.33 (s, 12H). ^13^C{^1^H} NMR (126 MHz, CDCl_3_) *δ* 159.6, 141.6, 130.1, 127.2, 84.9, 25.0, 24.1. ^11^B NMR (160 MHz, CDCl_3_) *δ* 30.3 mp = 101–102 °C.

#### 2,6-Dimethyl-4-(4,4,5,5-tetramethyl-1,3,2-dioxaborolan-2-yl)pyridine (1f)

General procedure A was followed with [IrOMe(cod)]_2_ (17 mg, 0.02 mmol, 0.25 mol%), B_2_pin_2_ (2.52 g, 10 mmol, 1 equiv.), dtbpy (13 mg, 0.05 mmol, 1.0 mol%) and THF (10 mL). To this solution was added 2,6-lutidine (2 mL, 10 mmol) and the resulting solution was stirred for 16 hours at 70 °C. After rotary evaporation, the residue was purified with Kugelrohr distillation at 60 °C (0.2 mm Hg) to yield a white solid (1.42 g, 61% yield). ^1^H NMR (500 MHz, CDCl_3_) *δ* 7.31 (s, 2H), 2.52 (s, 6H), 1.35 (s, 12H). ^13^C{^1^H} NMR (126 MHz, CDCl_3_) *δ* 157.2, 125.3, 84.4, 25.0, 24.4. ^11^B NMR (160 MHz, CDCl_3_) *δ* 30.7. NMR data matched those previously reported.^58^

#### *tert*-Butyl 3-(4,4,5,5-tetramethyl-1,3,2-dioxaborolan-2-yl)-1*H*-pyrrole-1-carboxylate (1g)

A modified general procedure A^45^ was followed with [IrOMe(cod)]_2_ (20 mg, 0.015 mmol, 0.25 mol%), B_2_pin_2_ (3.0 g, 12 mmol, 1 equiv.), dtbpy (16 mg, 0.03 mmol, 0.5 mol%) and THF (10 mL). To this mixture was added *n*-Boc-pyrrole (2 mL, 12 mmol). The solution was stirred for 16 hours at 60 °C. THF was removed *via* rotary evaporator, the residue was passed through a silica plug (DCM), and then concentrated on rotary evaporator and dried under high vacuum to yield a white solid (2.1 g, 70% yield). ^1^H NMR (500 MHz, CDCl_3_) *δ* 7.65 (t, *J* = 1.7 Hz, 1H), 7.27 (m, 1H), 6.47 (dd, *J* = 3.1, 1.5 Hz, 1H), 1.58 (s, 9H), 1.32 (s, 12H). ^13^C{^1^H} NMR (126 MHz, CDCl_3_) *δ* 148.8, 129.0, 120.9, 116.3, 84.0, 83.5, 28.1, 24.9. ^11^B NMR (160 MHz, CDCl_3_) *δ* 29.3. NMR data matched those previously reported.^45^

#### 3-(Benzofuran-2-yl)-1,1,1,3,5,5,5-heptamethyltrisiloxane (1z)

General procedure A was followed with 2,3 benzofuran (590 mg, 5 mmol), bis(trimethylsiloxy)methylsilane (1.2 g, 5.5 mmol, 1.1 equiv.), norborene (475 mg, 5 mmol, 1 equiv.), [Ir(OMe)cod]_2_ (35.5 mg, 0.05 mmol, 1.1 mol%), dtbpy (31 mg, 0.13 mmol, 2.4 mol%), THF (1 mL). The reaction was heated at 80 °C for 24 hours. The solution was concentrated by rotary evaporation and the residue purified by flash column chromatography (hexanes : ethyl acetate 95 : 5). Fractions containing the desired product were evaporated and dried under high vacuum to yield a clear colorless oil (1.3 g, 76% yield). ^1^H NMR (500 MHz, CDCl_3_) *δ* 7.60 (d, *J* = 7.6 Hz, 1H), 7.51 (d, *J* = 8.3 Hz, 1H), 7.29 (t, *J* = 6.9 Hz, 1H), 7.21 (t, *J* = 7.4 Hz, 1H), 7.01 (s, 1H), 0.35 (s, 3H), 0.13 (s, 18H). ^13^C{^1^H} NMR (126 MHz, CDCl_3_) *δ* 160.8, 157.8, 127.8, 124.7, 122.5, 121.5, 116.5, 111.6, 1.9, 0.1. ^29^Si NMR (99 MHz, CDCl_3_) *δ* 10.0, −43.2. ^1^H and ^13^C{^1^H} NMR values were consistent with those previously reported.^46^

### General procedure for synthesis of starting materials *via* pinacol coupling (1j, 1l, 1q, 1ae, 1af)

To an oven dried round bottom flask (50 mL)/vial (5 mL) containing a stir bar was added pinacol (1 equiv.) and the corresponding boronic acid (1 equiv.) in hexane/DCM. The resulting solution was stirred for 2–48 hours at room temperature. The product was isolated by passing through a Celite plug or washing with water.

#### 2-(Benzofuran-7-yl)-4,4,5,5-tetramethyl-1,3,2-dioxaborolane (1j)

Benzofuran-7-ylboronic acid (1.5 g, 9.3 mmol) and pinacol (1.2 g, 10.1 mmol, 1.1 equiv.) in DCM (30 mL) were consecutively added. The resulting solution was stirred overnight at room temperature. The solvent was concentrated by rotary evaporation to yield a white solid that was pure by NMR and used as is in the subsequent reaction (2.2 g, 99% yield). ^1^H NMR (500 MHz, CDCl_3_) *δ* 7.64 (d, *J* = 7.8 Hz, 1H), 7.58 (d, *J* = 8.3 Hz, 1H), 7.41 (s, 1H), 7.39–7.31 (m, 1H), 7.29–7.20 (m, 1H), 1.40 (s, 12H); ^13^C{^1^H} NMR (126 MHz, CDCl_3_) *δ* 157.6, 127.6, 126.1, 122.8, 122.0, 119.7, 112.1, 84.8, 24.9; ^11^B NMR (160 MHz, CDCl_3_) *δ* 27.7. NMR data matched those previously reported.^59^

#### *tert*-Butyl 2-(4,4,5,5-tetramethyl-1,3,2-dioxaborolan-2-yl)-1*H*-indole-1-carboxylate (1l)

(1-(*tert*-Butoxycarbonyl)-1*H*-indol-2-yl)boronic acid (1.0 g, 3.83 mmol) and pinacol (497 mg, 4.21 mmol, 1.1 equiv.) in DCM (15 mL) were consecutively added and the mixture stirred for 5 h at room temperature. The solvent was removed by rotary evaporation and the solid residue was stirred with water (1 mL) to remove excess pinacol. After the water was decanted off, the remaining solid was dissolved in DCM and the solution dried over MgSO_4_. After filtering off the MgSO_4_, the volatiles were removed by rotary evaporation to yield a yellowish solid (1.06 g, 80.6% yield). ^1^H NMR (500 MHz, CDCl_3_) *δ* 7.94 (d, *J* = 8.3 Hz, 1H), 7.54 (dd, *J* = 7.8, 1.0 Hz, 1H), 7.31–7.24 (m, 1H), 7.19 (t, *J* = 7.5 Hz, 1H), 6.85 (s, 1H), 1.70 (s, 9H), 1.41 (s, 12H); ^13^C{^1^H} NMR (126 MHz, CDCl_3_) *δ* 151.3, 136.7, 131.4, 124.5, 122.4, 121.2, 115.8, 115.0, 84.3, 84.2, 28.3, 24.9; ^11^B NMR (160 MHz, CDCl_3_) *δ* 29.1; mp 87–88 °C; GCMS for C_14_H_18_BNO_2_ calcd [M + H]^+^˙ 243.14 found 243.15.

#### 3-(4,4,5,5-Tetramethyl-1,3,2-dioxaborolan-2-yl)phenol (1q)

(3-Hydroxyphenyl)boronic acid (551 mg, 4 mmol) and pinacol (473 mg, 4 mmol) in hexane were consecutively added. The resulting solution was stirred for two hours at room temperature. The product was isolated by filtration through a Celite plug followed by removal of the volatiles by rotary evaporation to yield a white solid (790 mg, 89%). ^1^H NMR (500 MHz, CDCl_3_) *δ* 7.38 (d, *J* = 7.0 Hz, 1H), 7.28–7.22 (m, 2H), 6.95 (ddd, *J* = 8.0, 2.8,1.0 Hz, 1H), 4.64 (s, 1H), 1.34 (s, 12H); ^13^C{^1^H} NMR (126 MHz, CDCl_3_) *δ* 155.1, 129.4, 127.3, 121.2, 118.4, 84.1, 25.0;^57^^11^B NMR (160 MHz, CDCl_3_) *δ* 31.1; mp 85–87 °C; GCMS for C_12_H_17_BO_3_ calcd [M]^+^˙ 220.13 obtained 220.15. ^1^H and ^13^C{^1^H} NMR data were consistent with those previously reported.^60^

#### Trimethyl(3-(4,4,5,5-tetramethyl-1,3,2-dioxaborolan-2-yl)phenyl)silane (1ae)

A modified literature procedure was used for the synthesis.^22^ (3-(Trimethylsilyl)phenyl)boronic acid (1.0 g, 5.1 mmol) and pinacol (733 mg, 6.2 mmol, 1.2 equiv.) in hexane (20 mL) and DCM (7 mL) were consecutively added. (Note: DCM was added because the reagents were insoluble in hexane). The resulting solution was stirred for 27 h at room temperature. The volatiles were removed by rotary evaporation to yield a yellowish liquid. (1.4 g, 74% yield). ^1^H NMR (500 MHz, CDCl_3_) *δ* 7.96 (s, 1H), 7.80 (d, *J* = 7.5 Hz, 1H), 7.62 (d, *J* = 7.4 Hz, 1H), 7.36 (t, *J* = 7.4 Hz, 1H), 1.35 (s, 12H), 0.28 (s, 9H); ^13^C{^1^H} NMR (126 MHz, CDCl_3_) *δ* 139.8, 139.7, 136.4, 135.5, 127.2, 83.9, 25.0, −0.9; ^11^B NMR (160 MHz, CDCl_3_) *δ* 31.6; ^29^Si NMR (99 MHz, CDCl_3_) *δ* 4.0. ^1^H and ^13^C{^1^H} NMR data were consistent with those previously reported^61^ and ^11^B NMR data were also consistent those previously reported.^62^ (Note: ref. 47 reports the Bpin at 1.54 in the tabulated data however in the spectrum provided the peak appears at 1.34 ppm. Our carbon NMR data are shifted by 0.9 ppm downfield from those reported owing to CDCl_3_ being set to 78.1 ppm instead of 77.2 ppm).

#### Trimethyl(4-(4,4,5,5-tetramethyl-1,3,2-dioxaborolan-2-yl)phenyl)silane (1af)

A modified literature procedure was followed.^61^ (4-(Trimethylsilyl)phenyl)boronic acid (500 mg, 2.6 mmol) and pinacol (303 mg, 2.6 mmol, 1.0 equiv.) in hexane (20 mL) were consecutively added. The resulting solution was stirred for 2 h at room temperature. The solvent was removed by rotary evaporation to yield a white solid (682 mg, 95% yield). This material was used as is in the subsequent reaction. ^1^H NMR (500 MHz, CDCl_3_) *δ* 7.79 (d, *J* = 7.2 Hz, 2H), 7.53 (d, *J* = 7.2 Hz, 2H), 1.34 (s, 12H), 0.27 (s, 9H); ^13^C{^1^H} NMR (126 MHz, CDCl_3_) *δ* 144.4, 134.0, 132.7, 83.9, 25.0, −1.1; ^11^B NMR (160 MHz, CDCl_3_) *δ* 30.9; ^29^Si NMR (99 MHz, CDCl_3_) *δ* −3.9. ^1^H and ^13^C{^1^H} NMR data were consistent with those previously reported^61^ as were ^11^B NMR data.^62^ (Note CDCl_3_ reference reported in ref. 47 was set at 78.1 instead of 77.2 thus their tabular data are shifted by 1.0 ppm relative to ours. Similarly, a 0.7 ppm shift is observed in the ^1^H NMR spectrum).

### Synthesis of starting material (1k) *via* Miyaura borylation

#### 2-(Dibenzo[*b*,*d*]furan-4-yl)-4,4,5,5-tetramethyl-1,3,2-dioxaborolane (1k)

A modified literature procedure was followed.^63^ In a nitrogen filled glove box in a 10 mL oven dried Wheaton vial with a stir bar was added 4-bromodibenzofuran (247 mg, 1 mmol), B_2_pin_2_ (279.4 mg, 1.1 mmol, 1.1 equiv.), Pd(dppf)Cl_2_ (37 mg, 5 mol%, 0.05 mmol) and KOAc (294 mg, 3 mmol, 3 equiv.) in dioxane (5 mL). The resulting solution was taken outside the box and stirred for 16 h at 80 °C in an oil bath. The solution was concentrated by rotary evaporation, purified using flash column chromatography with hexane/ethyl acetate (2–4% gradient) solvents and concentrated and dried by rotary evaporation to yield a white solid (193 mg, 66% yield). ^1^H NMR (500 MHz, CDCl_3_) *δ* 8.06 (dd, *J* = 7.6, 1.4 Hz, 1H), 7.94 (dd, *J* = 7.6, 1.3 Hz, 1H), 7.90 (dd, *J* = 7.2, 1.4 Hz, 1H), 7.68 (dt, *J* = 8.2, 0.9 Hz, 1H), 7.45 (dt, *J* = 7.3, 1.3 Hz, 1H), 7.38–7.30 (m, 2H), 1.45 (s, 12H); ^13^C{^1^H} NMR (126 MHz, CDCl_3_) *δ* 160.6, 156.4, 134.5, 127.0, 124.0, 123.8, 122.6, 122.3, 120.5, 112.5, 84.2, 25.1; ^11^B NMR (160 MHz, CDCl_3_) *δ* 30.3. NMR data matched those previously reported.^64^

### Synthesis of starting materials *via* lithiation, silylation and borylation (1y, 1x, 1ab, 1ac, 1ad)

#### 2-Chloro-6-(trifluoromethyl)-3-(trimethylsilyl)pyridine (1y)

Synthesis of 1y was carried out using the reported literature procedure.^65^ Diisopropylamine (2 mL, 11.0 mmol) and (2-chloro-6-(trifluoromethyl))pyridine (2.0 g, 11.0 mmol) were consecutively added to a solution of *n*-BuLi (2.5 M in hexanes, 4.4 mL, 11.0 mmol) and THF (30 mL) at −85 °C. After 4 hours at −85 °C, chlorotrimethylsilane (3 mL, 2.7 g, 25 mmol) was added. The mixture was poured into water and extracted with DCM (3 × 20 mL). The combined organics were evaporated, and the residue purified by flash column chromatography on silica gel using hexane as the eluent. The product obtained after evaporation by rotary evaporation was a colorless liquid (2.25 g, 80% yield). 4-Chloro-6-trifluoromethyl-4-(trimethylsilyl)pyridine (1x) as a colorless liquid (128 mg, 5% yield) was obtained as a side product and was used as a starting material for 2x. ^1^H and ^13^C{^1^H} NMR data of 1y matched those previously reported.^65^ For 1x, while the observed aromatic protons were both shifted by 0.15 and 0.16 ppm relative to the reported in reference, carbon data matched those previously reported. Thus, we are confident in our assignment.^65^ For 1y: ^1^H NMR (500 MHz, CDCl_3_) *δ* 7.95 (d, 7.6 Hz, 1H), 7.58 (d, 7.5 Hz, 1H), 0.42 (s, 9H); ^13^C{^1^H} NMR (126 MHz, CDCl_3_) *δ* 157.3, 148.7 (q, *J* = 35.7 Hz), 146.4, 140.1, 120.9 (q, *J* = 274.2 Hz), 118.5, −1.34; ^19^F NMR (470 MHz, CDCl_3_) *δ* 68.3; ^29^Si NMR (99 MHz, CDCl_3_) *δ* −1.3. For 1x: ^1^H NMR (500 MHz, CDCl_3_) *δ* 7.64 (s, 1H), 7.58 (s, 1H), 0.3 (s, 9H); ^13^C{^1^H} NMR (126 MHz, CDCl_3_) *δ* 157.5, 151.7, 147.4 (d, *J* = 34.9 Hz), 131.9, 122.9 (q, *J* = 2.9 Hz), 121.1 (d, *J* = 274.6 Hz), −1.76.

#### *tert*-Butyl-2-(trimethylsilyl)-1*H*-pyrrole-1-carboxylate (1ab′)

The compound was prepared according to a reported procedure.^66^ Freshly distilled diisopropylamine (4 mL, 29.9 mmol) in THF was cooled to −78 °C under a nitrogen atmosphere. *n*-BuLi (2.5 M in hexanes, 12 mL, 30.0. mmol) was added slowly, and reaction mixture was allowed to warm to 0 °C for 10 min before re-cooling to −78 °C. *tert*-Butyl-1*H*-pyrrole (5.0 g, 29.9 mmol) was added dropwise to the solution prepared above. The reaction mixture was allowed to stir at −78 °C for 1 h before trimethylsilyl chloride (4 mL, 31.0 mmol) was added at −78 °C. The mixture was allowed to warm to room temperature, quenched with methanol, and stirred overnight. The solvent was then removed by rotary evaporation and the crude mixture was purified by short neck distillation at 110 °C, 0.05 atm to give a colorless oil (5.76 g, 80% yield). NMR data matched those previously reported.^66^

#### *tert*-Butyl-4-(4,4,5,5-tetramethyl-1,3,2-dioxaborolan-2-yl)-2-(trimethylsilyl)-1*H*-pyrrole-1-carboxylate (1ab)

A modified literature procedure was used to carry out the synthesis of 1ab.^66^ In a nitrogen filled glove box an oven dried round bottom flask (50 mL) was loaded with bis(1,5-cyclooctadiene)di-μ-methoxydiiridium(i) (14 mg, 0.02 mmol 0.5 mol%), bis(pinacolato)diboron (1.05 g, 4.2 mmol), di-*tert*-butyl bipyridine(11 mg, 0.04 mmol, 1 mol%), THF (10 mL) and a stir bar. To this solution was added *tert*-butyl-2-(trimethylsilyl)-1*H*-pyrrole-1-carboxylate (1 g, 4.2 mmol) (1ab′). The flask was placed in an oil bath and the reaction mixture stirred for 16 hours at 70 °C. THF was removed *via* rotary evaporation, the residue was passed through a silica plug (DCM). Rotary evaporation yielded a white solid (1.5 g, 96% yield). ^1^H NMR (500 MHz, CDCl_3_) *δ* 7.81 (s, 1H), 6.73 (s, 1H), 1.58 (s, 9H), 1.32 (s, 12H), 0.26 (s, 9H); ^13^C{^1^H} NMR (126 MHz, CDCl_3_) *δ* 149.5, 136.0, 133.4, 128.1, 83.7, 83.4, 28.1, 24.9, −0.2; ^29^Si NMR (99 MHz, CDCl_3_) *δ* 10.8; ^11^B NMR (160 MHz, CDCl_3_) *δ* 30.2; mp 93.8–94.2 °C; GCMS C_15_H_25_BNO_4_ calcd mass [M + H]^+^˙ 294.19 obtained 294.20. ^1^H and ^13^C{^1^H} NMR data were consistent with those previously reported in C_6_D_6_.^66^

#### 2-Bromo-6-(trimethylsilyl)pyridine (1ac′)

*n*-BuLi (2.5 M in hexanes, 3.4 mL, 8.5 mmol) was added dropwise to a stirred solution of 2,6 dibromopyridine (2.0 g, 8.4 mmol) in THF (40 mL) at −78 °C under nitrogen and subsequently warmed to −40 °C for 30 min before being cooled to −78 °C and dropwise addition of trimethylsilyl chloride (1 mL, 9.3 mmol) in THF (10 mL) *via* cannula. After 3 hours, the solution was allowed to warm to room temperature, filtered through Celite, washed with water (20 mL), dried and concentrated *in vacuo* to give 2-bromo-6-(trimethylsilyl)pyridine as yellow/brown oil (3.0 g, 79% yield). NMR data matched those previously reported.^67^

#### 2-Bromo-4-(4,4,5,5-tetramethyl-1,3,2-dioxaborolan-2-yl)-6-(trimethylsilyl)pyridine (1ac)

A nitrogen filled glove box an oven dried round bottom flask (50 mL) was loaded with bis(1,5-cyclooctadiene)di-μ-methoxydiiridium(i) (11 mg, 0.01 mmol, 0.25 mol%), bis(pinacolato)diboron (1.65 g, 6.5 mmol), di-*tert*-butyl bipyridine (9 mg, 0.03 mmol, 0.5 mol%), THF (10 mL) and a stir bar. To this solution was added 2-bromo-6-(trimethylsilyl)pyridine (1ac′) (2.0 g, 6.5 mmol). The resulting solution was heated to 70 °C *via* an oil bath and stirred for 16 hours. THF was removed by rotary evaporation, the residue passed through a silica plug (DCM) and evaporated to yield a white solid (2.0 g, 70% yield). ^1^H NMR (500 MHz, CDCl_3_) *δ* 7.71 (s, 2H), 1.35 (s, 12H), 0.32 (s, 9H); ^13^C{^1^H} NMR (126 MHz, CDCl_3_) *δ* 170.7, 143.8, 132.4, 132.3, 84.9, 25.0, 1.6, ^11^B NMR (160 MHz, CDCl_3_) *δ* 30.6; ^29^Si NMR (99 MHz, CDCl_3_) *δ* 4.3; mp 101.5–102.5 °C; HRMS (ESI) *m*/*z* calcd for C_14_H_24_BNBrSiO_2_ [M + H]^+^˙ 356.0852 found 356.0851.

#### Trimethyl(2-(4,4,5,5-tetramethyl-1,3,2-dioxaborolan-2-yl)phenyl)silane (1ad)

A modified literature procedure was used for this synthesis.^22^ HBpin (0.36 mL, 0.32 g, 2.47 mmol) was added to a mixture of catalyst [Pd(dppf)Cl_2_] (67.3 mg, 1.65 mol%), 2-(trimethylsilyl)phenyl trifluoromethanesulfonate (0.49 mL, 0.5 g, 1.65 mmol) and Hünig's base (0.66 mL, 489.7 mg, 3.78 mmol) in dioxane (4 mL) and the contents heated to 80 °C for 24 h. After cooling to room temperature sat. ammonium chloride ∼ 10 mL was added to neutralize the solution. The mixture was extracted with DCM (3 × 20 mL) and the organics then washed with H_2_O (50 mL). The organic layer was dried over Na_2_SO_4_. After filtration, the mixture was concentrated *via* rotary evaporation, and purified by flash column chromatography (10–20% Et_2_O and hexane). Product containing fractions were combined and concentrated by rotary evaporation to obtain a white solid (442 mg, 98% yield). ^1^H NMR (500 MHz, CDCl_3_) *δ* 7.91 (d, *J* = 7.3 Hz, 1H), 7.62 (d, *J* = 7.3 Hz, 1H), 7.40 (td, *J* = 7.4, 1.5 Hz, 1H), 7.35 (td, *J* = 7.4, 1.4 Hz, 1H), 1.35 (s, 12H), 0.34 (s, 9H). NMR data were consistent with those previously reported.^68^

#### 1,2-Bis(4,4,5,5-tetramethyl-1,3,2-dioxaborolan-2-yl)benzene (1v)

In a 20 mL oven dried sealed tube under nitrogen blanket with a stir bar was added (2-chlorophenyl)trimethylsilane (600 mg, 4.3 mmol), B_2_pin_2_ (1.1 g, 4.2 mmol, 1.0 equiv.), Pd_2_dba_3_ (80 mg, 2 mol%, 0.09 mmol), xphos (81 mg, 0.17 mmol, 4 mol%) and NaOAc (424 mg, 5.7 mmol, 1.2 equiv.) in dioxane (5 mL). The resulting solution was taken outside the box and stirred for 16 h at 120 °C in an oil bath. The solvent was removed by rotary evaporation and the residue purified by flash column chromatography eluting with DCM. Product containing fractions were combined and concentrated by rotary evaporation to yield a white solid (119 mg, 9% yield). ^1^H NMR (500 MHz, CDCl_3_) *δ* 7.64 (dd, *J* = 5.5, 3.3 Hz, 2H), 7.37 (dd, *J* = 5.5, 3.3 Hz, 2H), 1.37 (s, 24H). ^1^H NMR data were consistent with those previously reported and it also reports ^13^C and ^11^B NMR data.^69^

### General procedure B for hydrogenation

Arene/heteroarene (0.5 mmol), 5% Rh/C (25 mg, 2 mol%) or Rh/Al_2_O_3_ (50 mg, 4 mol%), and a stir bar were loaded into a 300 mL Parr reactor pressure vessel. Each run was flushed with hydrogen at least twice. Ethanol (5 mL) was added and the reactor was sealed and pressurized with hydrogen gas (48 atm). After 16 hours the reactor was opened and the reaction mixture filtered through Celite. The Celite was washed with methanol (3 × 5 mL) and the solvent was removed by rotary evaporation to yield the corresponding product. (Note: we chose 16 h for our reaction time owing to our preference for a common time point and related literature hydrogenations being typically run for 16–24 h. Reactions for individual substrates were not optimized.)

#### 3-(4,4,5,5-Tetramethyl-1,3,2-dioxaborolan-2-yl)piperidin-1-ium bromide (2b)

General procedure B was followed with 3-bromo-5-(4,4,5,5-tetramethyl-1,3,2-dioxaborolan-2-yl)pyridine (142 mg, 0.5 mmol) and 5% Rh/C (25 mg, 2 mol%) for 16 h. After purification the solvent was removed under vacuum to yield 134 mg of a white solid consisting of 73 : 27 molar mixture of 2b : 3b (106 mg of 2b). ^1^H NMR (500 MHz, D_2_O) *δ* 3.42–3.29 (m, 2H), 3.13 (t, *J* = 5.8 Hz, 1H, deboronated piperidine), 3.04–2.86 (m, 2H), 1.96–1.80 (m, 2H), 1.80–1.59 (m, 3H, deboronated piperidine), 1.55–1.42 (m, 1H), 1.42–1.32 (m, 1H), 1.19 (s, 12H); ^13^C{^1^H} NMR (126 MHz, D_2_O) *δ* 75.6, 45.9, 44.4, 44.3, 23.6, 23.4, 22.8, 22.1, 21.4; ^11^B NMR (160 MHz, D_2_O) *δ* 30.2; HRMS (ESI) *m*/*z* calcd for C_11_H_23_BNO_2_ [M − Br]^+^˙ 212.1821; found. 212.1826.

#### 2-(4,4,5,5-Tetramethyl-1,3,2-dioxaborolan-2-yl)piperidin-1-ium bromide (2c)

General procedure B was followed with bromo-6-(4,4,5,5-tetramethyl-1,3,2-dioxaborolan-2-yl)pyridine (142 mg, 0.5 mmol) and 5% Rh/C (25 mg, 2 mol%) for 16 h. After purification the solvent was removed under vacuum to yield a white solid (143 mg, 99% yield). ^1^H NMR (500 MHz, D_2_O) *δ* 3.37–3.28 (m, 1H), 3.13 (t, *J* = 6.0 Hz, 1H), 2.91 (td, *J* = 12.5, 3.1 Hz, 1H), 2.71 (dd, *J* = 12.6, 3.2 Hz, 1H), 1.93 (ddd, *J* = 14.3, 3.6, 1.6 Hz, 1H), 1.88–1.78 (m, 2H), 1.78–1.72 (m, 1H), 1.71–1.57 (m, 2H), 1.55–1.44 (m, 1H), 1.19 (s, 12H); ^13^C{^1^H} NMR (126 MHz, D_2_O) *δ* 75.5, 44.5, 24.3, 23.6, 22.5, 22.0; ^11^B NMR (160 MHz, D_2_O) *δ* 28.42. Mp 198–199 °C; HRMS (ESI) *m*/*z* calcd for C_11_H_23_BNO_2_ [M − Br]^+^˙ 212.1821; found. 212.1822.

#### 4-(4,4,5,5-Tetramethyl-1,3,2-dioxaborolan-2-yl)piperidin-1-ium bromide (2d)

General procedure B was followed with 2,6-dichloro-4-(4,4,5,5-tetramethyl-1,3,2-dioxaborolan-2-yl)pyridine (1d) (90 mg, 0.25 mmol) and 5% Rh/Al_2_O_3_ (50 mg, 8 mol%) for 16 h. MeOH was used for filtration through Celite to avoid deboronation and the solvent was removed under vacuum. The reaction mixture showed 85% conversion to product. Diethyl ether was added to the reaction mixture, which dissolved the starting material and not 2d. Decantation and drying by rotary evaporation yielded a white solid (60 mg, 82% yield). (Notes: adding ethyl acetate or hexane to the crude reaction mixture resulted in deboronation. Dissolving 1d in D_2_O also resulted in deboronation). While the HBr salt has not been reported, the HCl salt has been.^70^^1^H NMR (500 MHz, DMSO) *δ* 3.08 (d, *J* = 12.4 Hz, 2H), 2.87 (t, *J* = 11.5 Hz, 2H), 1.77–1.67 (m, 2H), 1.60–1.48 (m, 2H), 1.28 (s, 1H), 1.19 (s, 12H). ^13^C{^1^H} NMR (126 MHz, DMSO) *δ* 83.2, 43.7, 24.6, 23.4; ^11^B NMR (160 MHz, DMSO) *δ* 33.1. Mp 169–170 °C; GCMS for C_11_H_22_BNO_2_ (free base) calcd [M]^+^˙ 211.17 found 211.15.

#### 2-Methyl-4-(4,4,5,5-tetramethyl-1,3,2-dioxaborolan-2-yl)piperidin-1-ium bromide (2e)

General procedure B was followed with 2-bromo-6-methyl-4-(4,4,5,5-tetramethyl-1,3,2-dioxaborolan-2-yl)pyridine (1e) (85 mg, 0.3 mmol), 5% Rh/Al_2_O_3_ (50 mg, 4 mol%) for 16 h. After purification the solvent was removed under vacuum to yield a tan solid (86 mg, 94% yield). Stereochemistry was assumed to be *cis* based on analogy to 2f. ^1^H NMR (500 MHz, D_2_O) *δ* 3.41–3.35 (m, 1H), 3.24–3.08 (m, 1H), 2.93 (td, *J* = 13.0, 3.3 Hz, 1H), 2.02–1.84 (m, 2H), 1.53 (dq, *J* = 13.2, 4.1 Hz, 1H), 1.43–1.30 (m, 1H), 1.29–1.24 (m, 4H), 1.18 (s, 12H); ^13^C{^1^H} NMR (126 MHz, D_2_O) *δ* 75.5, 53.4, 45.1, 31.7, 23.6, 23.2, 18.7; ^11^B NMR (160 MHz, D_2_O) 30.2, mp 209–211 °C, HRMS (ESI) *m*/*z* calcd for C_12_H_25_BNO_2_ [M − Br]^+^˙ 226.1978 found 226.1986.

#### 2,6-Dimethyl-4-(4,4,5,5-tetramethyl-1,3,2-dioxaborolan-2-yl)piperidine (2f)

General procedure B was followed with 2,6-dimethyl-4-(4,4,5,5-tetramethyl-1,3,2-dioxaborolan-2-yl)pyridine (1f) (117 mg, 0.5 mmol), 5% Rh/Al_2_O_3_ (50 mg, 4 mol%) for 40 h. After purification the solvent was removed under vacuum to yield a white solid (102 mg, 86% yield). This reaction gave 81% conversion to product in 19 h, 95% conversion to product in 28 h and 100% conversion to product in 10 h. *Cis* diastereoselectivity of the compound was ascertained by oxidation of the boronic ester to and comparing the NMR data of the hydroxy bearing methine in 2f to analogous literature data reported for *cis* 2-methyl-6-propyl piperidin-4-ol^71^ and *cis* 2-methyl-6-nonylpiperidin-4-ol.^72^ Literature reports the chemical shift of the protons on the OH bearing carbon appearing at 3.65 (tt, *J* = 11.8, 4.5 Hz) ppm for *cis* 2-methyl-6-propyl piperidin-4-ol^56^ and at 3.66 (dddd, *J* = 11.0, 11.0, 4.6, 4.6) for *cis* 2-methyl-6-nonylpiperidin-4-ol.^56^ The corresponding chemical shift for 2f appears at 3.68 (tt, *J* = 11.1, 4.5 Hz) ppm. ^1^H NMR (500 MHz, CDCl_3_) *δ* 2.68–2.52 (m, 2H), 1.64 (d, *J* = 12.8 Hz, 2H), 1.20 (s, 12H), 1.03 (d, *J* = 6.2 Hz, 6H), 0.94 (q, *J* = 12.3 Hz, 3H); ^13^C{^1^H} NMR (126 MHz, CDCl_3_) *δ* 83.1, 53.5, 33.8, 24.9, 21.6; ^11^B NMR (160 MHz, CDCl_3_) *δ* 33.7; HRMS (ESI) *m*/*z* calcd for C_13_H_26_BNO_2_ [M]^+^˙ 239.2057 found 239.2059.

#### *tert*-Butyl 3-(4,4,5,5-tetramethyl-1,3,2-dioxaborolan-2-yl)pyrrolidine-1-carboxylate (2g)

General procedure B was followed with *tert*-butyl 3-(4,4,5,5-tetramethyl-1,3,2-dioxaborolan-2-yl)-1*H*-pyrrole-1-carboxylate (1g) (142 mg, 0.5 mmol), 10% Pd/C (15 mg, 3 mol%) for 16 h. After purification solvent was removed under vacuum to yield a clear oil (122 mg, 82% yield). A mixture of two rotational isomers was observed. Running this reaction on one gram scale using 5% Rh/C (138 mg) and 13 mL ethanol afforded 1 g in 73% yield. ^1^H NMR (500 MHz, C_6_D_6_) *δ* 3.74 (dd, *J* = 10.7, 8.5 Hz, 1H), 3.59–3.47 (m, 3H), 3.40 (t, *J* = 10.3 Hz, 1H), 3.32 (ddd, *J* = 10.6, 7.5, 3.2 Hz, 1H), 3.17 (td, *J* = 10.1, 6.8 Hz, 1H), 3.01 (td, *J* = 9.8, 7.0 Hz, 1H), 1.74–1.54 (m, 4H), 1.47 (d, *J* = 7.9 Hz, 18H), 1.44–1.34 (m, 2H), 0.98 (s, 24H, mixture of 2 rotamers); ^13^C{^1^H} NMR (126 MHz, C_6_D_6_) *δ* 154.3, 83.3, 78.3 (2 rotamer peaks), 48.4 (2 rotamer peaks), 47.0, 28.7, 27.7, 24.8; ^11^B NMR (160 MHz, C_6_D_6_) *δ* 33.4; NMR data were consistent with those previously reported.^73^ HRMS (ESI) *m*/*z* calcd for C_15_H_28_BNO_4_Na [M + Na]^+^˙ 320.2009 found 320.2013.

#### 4,4,5,5-Tetramethyl-2-(tetrahydrofuran-2-yl)-1,3,2-dioxaborolane (2h)

General procedure B was followed with 2-(furan-2-yl)-4,4,5,5-tetramethyl-1,3,2-dioxaborolane (100 mg, 0.5 mmol), 5% Rh/C (25 mg, 2 mol%) for 13 h. After purification, the solvent was removed using rotary evaporator to yield a clear oil (100% conversion, 8.3% OBpin monomer) which amounted to 93 mg of 2h (89% yield). ^1^H NMR (500 MHz, CDCl_3_) *δ* 4.10–3.76 (m, 1H), 3.62 (q, *J* = 7.7 Hz, 1H), 3.42 (dd, *J* = 10.9, 7.2 Hz, 1H), 2.11–2.01 (m, 1H), 1.92–1.83 (m, 2H), 1.77–1.64 (m, 1H), 1.28 (s, 12H), 1.24 (s, 1H, B byproduct); ^13^C{^1^H} NMR (126 MHz, CDCl_3_) *δ* 83.8, 74.9 (B byproduct), 68.9, 28.1, 26.2, 24.7 (B byproduct), 24.7 (Bpin C), 24.6 (Bpin C); ^11^B NMR (160 MHz, CDCl_3_) *δ* 32.3, 22.3 (B byproduct). NMR data were consistent with those previously reported.^21^

### Recyclability test experiment

#### Test 1

General procedure B was followed with 2-(furan-2-yl)-4,4,5,5-tetramethyl-1,3,2-dioxaborolane (500 mg, 2.5 mmol), 5% Rh/C (235 mg, 4 mol%) with 10 mL ethanol for 4 h. The catalyst was recycled by filtration using a sintered funnel and washed with EtOAc. After purification of the filtrate, the solvent was removed by rotary evaporation to yield a clear oil of 1h (100% conversion, 66% yield).

#### Test 2

The recovered catalyst from test 1 was used again with (500 mg, 2.5 mmol) with 10 mL ethanol for 4 h. The catalyst was recycled by filtration using a sintered funnel and washed with EtOAc. After purification of the filtrate, the solvent was removed by rotary evaporation to yield a clear oil (80% conversion to product, 414 mg of mixture, 82% crude yield).

#### Test 3

The recovered catalyst from test 2 was used again with (500 mg, 2.5 mmol) with 10 mL ethanol for 6 h. The catalyst was recycled by filtration using a sintered funnel and washed with EtOAc. After purification of the filtrate, the solvent was removed by rotary evaporation to yield a clear oil (100% conversion, 376 mg, 75% yield).

#### Test 4

The recovered catalyst from test 3 was used again with (500 mg, 2.5 mmol) with 10 mL ethanol for 5 h. The catalyst was recycled by filtration using a sintered funnel and washed with EtOAc. After purification of the filtrate, the solvent was removed by rotary evaporation to yield a clear oil (100% conversion, 421 mg, 84% yield).

#### Test 5

The recovered catalyst from test 4 was used again with (500 mg, 2.5 mmol) with 10 mL ethanol for 5 h. After purification, the solvent was removed by rotary evaporation to yield a clear oil (100% conversion, 427 mg, 84% yield).

##### 4,4,5,5-Tetramethyl-2-(5-methyltetrahydrofuran-2-yl)-1,3,2-dioxaborolane (2i)

General procedure B was followed with 4,4,5,5-tetramethyl-2-(5-methylfuran-2-yl)-1,3,2-dioxaborolane 208 mg, 1.0 mmol), 5% Rh/C (25 mg, 1 mol%) for 16 h. After purification solvent was removed by rotary evaporation to yield a clear oil (165 mg (with 6.3% HOBpin) amounting to 157 mg of 2i (74% yield). A diastereomeric ratio of 97 : 3 was measured by ^1^H NMR and GC. *Cis* stereochemistry was established by COSY and 1D NOE NMR experiments. In the NOE experiments, irradiating 3.63 ppm (H on C2) showed enhancement of the peak at 3.73 ppm (H on C5) and irradiating the peak 3.73 ppm (H on C5) showed enhancement of the peak at 3.63 ppm (H on C2). Byproduct HOBpin was confirmed by adding HOBpin to the NMR tube and observing the increase in intensity of the ^11^B peak for HOBpin. When Pd/C was used as a catalyst, 18% HOBpin was observed. Attempts to remove HOBpin by sublimation were unsuccessful. Hydrogenation using Rh/C gave only 6.3% HOBpin and reducing the time of reaction to 12.5 h further lowered the amount of HOBpin to 4%. ^1^H NMR (500 MHz, C_6_D_6_) *δ* 3.77 (dq, *J* = 12.8, 6.3 Hz, 1H), 3.67 (dd, *J* = 9.9, 7.9 Hz, 1H), 2.04–1.84 (m, 2H), 1.75–1.64 (m, 1H), 1.34–1.26 (m, 1H), 1.24 (d, *J* = 6.0 Hz, 3H), 1.028 (s, 6H, Bpin), 1.035 (s, 6H, Bpin); ^13^C{^1^H} NMR (126 MHz, C_6_D_6_) *δ* 83.4 (Bpin CH), 76.8 (Bpin CH), 34.0, 28.8, 24.9 (Bpin CH_3_), 24.8 (Bpin CH_3_), 21.2; ^11^B NMR (160 MHz, C_6_D_6_) *δ* 32.7; HRMS (ESI) *m*/*z* calcd for C_11_H_21_BO_3_Na [M + Na]^+^˙ 235.1481 found 235.1493.

##### 4,4,5,5-Tetramethyl-2-(octahydrobenzofuran-2-yl)-1,3,2-dioxaborolane (2j)

General procedure B was followed with 2-(benzofuran-2-yl)-4,4,5,5-tetramethyl-1,3,2-dioxaborolane (1j) (122 mg, 0.5 mmol), 5% Rh/C (25 mg, 2 mol%) dissolved in hexane (5 mL) for 16 h at 55 atm pressure. This compound was purified by passing the reaction mixture in ethyl acetate through Celite and evaporating the mother liquor by rotary evaporation to yield a clear oil (124 mg (with ∼20 mol% OBpin monomer *via*^1^H-NMR)) amounting to ∼107 mg of 2j (∼85% yield). ^1^H NMR (500 MHz, CDCl_3_) *δ* 3.73 (q, *J* = 4.1 Hz, 1H), 3.69 (dd, *J* = 10.9, 7.1 Hz, 1H), 2.22–2.12 (m, 1H), 2.05–1.97 (m, 1H), 1.97–1.89 (m, 1H), 1.61–1.46 (m, 5H), 1.40–1.31 (m, 1H), 1.27 (s, 6H), 1.25 (s, 6H), 1.25 (s, 3H, B byproduct) 1.23–1.17 (m, 2H); ^13^C{^1^H} NMR (126 MHz, CDCl_3_) *δ* 83.7, 83.1 (B byproduct), 78.3, 38.2, 34.5, 28.2, 28.21, 24.77 (Bpin CH_3_), 24.55 (B byproduct), 24.53 (Bpin CH_3_), 24.3, 21.0; ^11^B NMR (160 MHz, CDCl_3_) *δ* 32.7, 22.4 (B byproduct). The stereochemical assignment and NMR data were consistent with those previously reported.^21^

##### 2-(Dodecahydrodibenzo[*b*,*d*]furan-4-yl)-4,4,5,5-tetramethyl-1,3,2-dioxaborolane (2k)

General procedure B was followed with 4,4,5-trimethyl-2-(tetrahydrodibenzofuran-4-yl)-1,3,2-dioxaborolane (147 mg, 0.5 mmol) (1k), 5% Rh/C (25 mg, 2 mol%), H_2_ at 60 atm, using hexane (5 mL) as a solvent at 60 °C for 21 h. The catalyst was removed by passing through Celite using diethyl ether as a solvent (3 × 5 mL). The solvent was removed by rotary evaporation to give a colorless oil that contained product (100% conversion by ^1^H NMR) and 8% deboronated material (tetrahydrodibenzofuran) as determined by GCMS analysis. The desired compound was purified by flash column chromatography eluting with an ethyl acetate/hexane gradient (2–10%) to give a colorless oil (58 mg, 38%). Only the major isomer was isolated. The stereochemistry was determined to be *cis* using 2-D NMRs and 1D NOE (see S49†). All spectral data matched those previously reported^26^ except for the ^11^B chemical shift that was reported to be at 22.40, which is inconsistent with a C–boronic ester peak and is more likely boron byproducts. ^1^H NMR (500 MHz, CDCl_3_) *δ* 3.88 (t, *J* = 3.7 Hz, 1H), 3.82 (td, *J* = 10.5, 5.9 Hz, 1H), 2.29–2.16 (m, 1H), 1.87–1.75 (m, 2H), 1.68 (dt, *J* = 12.9, 3.5 Hz, 1H), 1.64–1.53 (m, 2H), 1.47 (tt, *J* = 12.8, 4.7 Hz, 5H), 1.42–1.35 (m, 1H), 1.33–1.25 (m, 2H), 1.22 (s, 6H), 1.23 (s, 7H) 1.19 (s, *J* = 4.6 Hz, 1H), 1.07 (qt, *J* = 12.7, 3.0 Hz, 1H); ^13^C{^1^H} NMR (126 MHz, CDCl_3_) *δ* 82.9, 77.6, 76.4, 42.0, 41.4, 28.6, 25.7, 25.1, 24.8 (Bpin C), 24.5 (Bpin C), 22.1, 21.3, 21.2, 19.6; ^11^B NMR (160 MHz, CDCl_3_) *δ* 33.4; GCMS C_18_H_31_BO_3_ calcd [M]^+^˙ 306.24 found 306.25.

##### *tert*-Butyl 2-(4,4,5,5-tetramethyl-1,3,2-dioxaborolan-2-yl)octahydro-1*H*-indole-1-carboxylate (2l)

General procedure B was followed with *tert*-butyl 2-(4,4,5,5-tetramethyl-1,3,2-dioxaborolan-2-yl)-1*H*-indole-1-carboxylate (1l) (174 mg, 0.5 mmol), 5% Rh/C (25 mg, 2 mol%) for 16 h. After purification solvent was removed by rotary evaporation to yield a colorless oil (170 mg, 97% yield). ^1^H NMR (500 MHz, CDCl_3_) *δ* 3.78 (dt, *J* = 11.6, 6.3 Hz, 1H), 3.74–3.68 (m, 1H), 3.62–3.50 (m, 1H), 3.18–3.01 (m, 2H), 2.33–2.18 (m, 2H), 2.07–1.97 (m, 2H), 1.89–1.77 (m, 2H), 1.76–1.52 (m, 8H), 1.46–1.39 (m, 19H, Boc of 2 rotamers), 1.37–1.28 (m, 2H), 1.27–1.18 (m, 25H, 2 Bpin rotamers), 1.18–1.07 (m, 3H); ^13^C{^1^H} NMR (151 MHz, toluene-d_8_, 80 °C) *δ* 154.4, 83.4, 78.2, 57.0, 28.9, 28.6, 26.8, 25.2, 24.8, 24.3, 21.4; ^11^B NMR (160 MHz, CDCl_3_) *δ* 32.5; GCMS C_15_H_25_BNO_4_ calcd [M − *t*Bu]^+^˙ 294.19 found 294.20. ^1^H and ^13^C{^1^H} NMR data are consistent with those previously reported,^9^ however that report only tabulated data for one rotamer although both the rotamers are observed in the spectrum.^21^

##### 2-Cyclohexyl-4,4,5,5-tetramethyl-1,3,2-dioxaborolane (2p)

General procedure B was followed with 4,4,5,5-tetramethyl-2-phenyl-1,3,2-dioxaborolane (210 mg, 1 mmol), 5% Rh/C (25 mg, 1 mol%) for 16 h. After purification solvent was removed by rotary evaporation to yield a clear oil (208 mg, 99% yield). ^1^H NMR (500 MHz, CDCl_3_) *δ* 1.69–1.62 (m, 2H), 1.62–1.53 (m, 3H), 1.37–1.26 (m, 5H), 1.23 (s, 12H), 1.01–0.92 (m, 1H); ^13^C{^1^H} NMR (126 MHz, CDCl_3_) *δ* 82.9, 28.1, 27.3, 26.9, 24.9; ^11^B NMR (160 MHz, CDCl_3_) *δ* 33.8. NMR data matched those previously reported.^21^

##### 3-(4,4,5,5-Tetramethyl-1,3,2-dioxaborolan-2-yl)cyclohexan-1-ol (2q)

General procedure B was followed with 3-(4,4,5,5-tetramethyl-1,3,2-dioxaborolan-2-yl)phenol (1q) (110 mg, 0.5 mmol), 5% Rh/C (25 mg, 2 mol%) for 16 h. After purification solvent was removed by rotary evaporation to yield a clear oil (80 mg, 71% yield). A 1.2 : 1 mixture of diastereomers was obtained. The major product was assumed to be the *cis* isomer based on a comparison of its ^1^H NMR to that of known^48^ TMS protected 2q. NMR data of the mixture are reported. ^1^H NMR^67^ (500 MHz, CDCl_3_) *δ* 3.79 (s, 1H), 3.59 (s, 1H), 1.96 (d, *J* = 13.0 Hz, 1H), 1.93–1.82 (m, 2H), 1.82–1.70 (m, 2H), 1.70–1.60 (m, 2H), 1.55–1.44 (m, 3H), 1.44–1.32 (m, 6H), 1.23 (s, 24H), 1.20–1.11 (m, 1H), 1.00 (t, *J* = 11.3 Hz, 1H), 0.91–0.79 (m, 1H); ^13^C{^1^H} NMR (126 MHz, CDCl_3_) *δ* 83.16, 83.13, 71.01, 68.29, 36.77, 35.99, 35.46, 34.50, 26.92, 26.78, 24.87, 24.83, 24.80; ^11^B NMR (160 MHz, CDCl_3_) *δ* 34.0. GCMS C_11_H_20_BO_2_ calcd [M − OH–Me]^+^˙ 195.16 found 195.05.

##### 4,4,5,5-Tetramethyl-2-(-3-(trifluoromethyl)cyclohexyl)-1,3,2-dioxaborolane (2r)

General procedure B was followed with 4,4,5,5-tetramethyl-2-(3-(trifluoromethyl)phenyl)-1,3,2-dioxaborolane (136 mg, 0.5 mmol), 5% Rh/C (25 mg, 2 mol%) for 16 h. After purification solvent was removed by rotary evaporation to yield a clear oil (109 mg, 80% yield). *Cis* and *trans* diastereomers were observed *via*^19^F NMR in a 3 : 1 *cis* to *trans* ratio. ^1^H NMR^66^ (500 MHz, CDCl_3_) *δ* 2.19–2.07 (m, 1H), 2.04–1.89 (m, 8H), 1.89–1.70 (m, 8H), 1.48–1.32 (m, 3H), 1.24 (s, 17H), 1.23 (s, 29H), 1.21–1.09 (m, 4H), 0.99–0.87 (m, 2H); ^13^C{^1^H} NMR (126 MHz, CDCl_3_) *δ* 129.1 (q), 83.4, 83.3, 42.7 (q, *J* = 26.1 Hz), 40.2 (q, *J* = 25.8 Hz), 27.0, 26.9, 26.2 (q, *J* = 2.5 Hz), 26.0, 25.2 (q, *J* = 2.6 Hz), 25.0 (q, *J* = 2.9 Hz), 24.9 (d, *J* = 4.3 Hz), 24.9 (d, *J* = 3.3 Hz), 24.0; ^19^F NMR (470 MHz, CDCl_3_) *δ* −73.61 (d, *J* = 8.8 Hz, 1H), −74.03 (d, *J* = 8.3 Hz, 3H); ^11^B NMR (160 MHz, CDCl_3_) *δ* 33.7; GCMS calcd for C_12_H_19_BO_2_F_3_ [M − Me]^+^˙ 263.14, found 263.20.

*Cis* and *trans* isomers were assigned by oxidation of the Bpin and then comparing spectra of the alcohols to those previously reported.^74^ The proton peak on the OH bearing carbon appeared at 4.23 ppm (t, *J* = 3.29 Hz) (lit. ^74^ 4.23 (brtt *J* = 3.5, 3.0 Hz) and for the *cis* isomer at 3.62 ppm (tt, *J* = 10.8, 4.2 Hz) (lit. ^74^ 3.60 ppm (tt, *J* = 10.7, 4.2 Hz)). The ratios of *cis* and *trans* isomers after oxidation (2.6 : 1) (*cis* : *trans*) was comparable to the ratio before oxidation.

##### Ethyl-3-(4,4,5,5-tetramethyl-1,3,2-dioxaborolan-2-yl)cyclohexane-1-carboxylate (2s)

General procedure B was followed with ethyl 3-(4,4,5,5-tetramethyl-1,3,2-dioxaborolan-2-yl)benzoate (116 mg, 0.42 mmol), 5% Rh/C (25 mg, 2 mol%) for 16 h. After purification solvent was removed by rotary evaporation to yield a clear oil (95 mg, 81% yield). ^1^H-NMR showed both *cis* and *trans* diastereomers that were isolated as a mixture in a 3.3 : 1 ratio. The major isomer was determined using 2D NMR and 1D NOE. The proton α to carboxyl group and the proton α to Bpin both showed NOEs with each other indicating a *cis* orientation. ^1^H NMR^81^ (500 MHz, CDCl_3_) *δ* 4.15–4.02 (m, 7H), 2.44–2.34 (m, 1H), 2.26–2.12 (m, 4H), 1.99 (d, *J* = 13.3 Hz, 4H), 1.90 (d, *J* = 12.8 Hz, 5H), 1.83–1.75 (m, 5H), 1.73 (d, *J* = 13.0 Hz, 4H), 1.68–1.58 (m, 4H), 1.56–1.42 (m, 5H), 1.40–1.27 (m, 12H), 1.25–1.17 (m, 57H), 1.14 (dt, *J* = 12.8, 3.0 Hz, 3H), 1.13–1.06 (m, 1H), 0.92 (dt, *J* = 11.4, 3.2 Hz, 3H), 0.87–0.78 (m, 1H) ^13^C{^1^H} NMR (126 MHz, CDCl_3_) *δ* 176.4, 176.3, 83.1, 60.1, 44.3, 41.4, 30.4, 29.5, 29.1, 29.0, 27.2, 27.1, 26.5, 24.9, 24.9, 24.8, 24.8, 24.5, 14.4, 14.3; ^11^B NMR (160 MHz, CDCl_3_) *δ* 33.2; GCMS calcd for C_14_H_24_BO_4_ [M − Me]^+^˙ 267.18, found 267.20.

##### 4,4,5,5-Tetramethyl-2-(3-methylcyclohexyl)-1,3,2-dioxaborolane (2t)

General procedure B was followed with 4,4,5,5-tetramethyl-2-(*m*-tolyl)-1,3,2-dioxaborolane (109 mg, 0.5 mmol), 5% Rh/C (25 mg, 2 mol%) for 16 h. After purification, solvent was removed under vacuum to yield a clear oil (86 mg, 77% yield). The *cis* and *trans* diastereomeric ratio was 2.2 : 1 *via* NMR and 2.6 : 1 *via* GC. ^1^H NMR^81^ (500 MHz, CDCl_3_) *δ* 2.17 (s, 1H), 1.77 (d, *J* = 13.0 Hz, 1H), 1.71 (d, *J* = 10.5 Hz, 8H), 1.67–1.55 (m, 5H), 1.43 (ddp, *J* = 10.1, 6.8, 3.3 Hz, 1H), 1.36–1.28 (m, 6H), 1.23 (s, 12H, Bpin, minor isomer), 1.22 (s, 26H, Bpin, major isomer), 1.19 (t, *J* = 2.8 Hz, 1H), 1.12–1.05 (m, 4H), 0.92 (tt, *J* = 12.8, 2.7 Hz, 4H), 0.87 (d, *J* = 6.6 Hz, 4H), 0.84 (d, *J* = 6.5 Hz, 9H); ^13^C{^1^H} NMR (126 MHz, CDCl_3_) *δ* 82.93, 82.87, 36.59, 36.06, 35.47, 35.14, 33.64, 30.98, 27.64, 27.58, 25.00, 24.93, 24.87, 24.86, 23.22, 22.46; ^11^B NMR (160 MHz, CDCl_3_) 33.8. GCMS calcd for C_12_H_22_BO_2_ [M − Me]^+^˙ 209.17 found 209.20. The NMR data of the mixture were consistent with those previously reported.^21^*Cis* and *trans* isomer assignments were confirmed by oxidation of the Bpin and comparing the spectra of the alcohols to those previously reported.^75^ The proton on the OH bearing carbon appeared at 4.05 ppm (1H, m) (lit. ^75^ 4.06 (m)) for the *trans* isomer and 3.57 ppm (lit. ^75^ 3.57 (m)) for the *cis* isomer. The *cis* and *trans* ratio after oxidation (3.3 : 1) was comparable to that before oxidation ratio (2.6 : 1, *via* GC).

##### 2-(3-Methoxycyclohexyl)-4,4,5,5-tetramethyl-1,3,2-dioxaborolane (2u)

General procedure B was followed with 2-(3-methoxyphenyl)-4,4,5,5-tetramethyl-1,3,2-dioxaborolane (117 mg, 0.5 mmol), 5% Rh/C (25 mg, 2 mol%) for 16 h. After purification, solvent was removed by rotary evaporation to yield a clear oil (104 mg with 22% (by GCMS)) demethoxylated cyclohexane-Bpin, which corresponded to 82 mg of 2u as a 5.6 : 1 *cis*/*trans* mixture (68% yield). For the *cis* isomer: ^1^H NMR (500 MHz, CDCl_3_) *δ* 3.33 (s, 3H), 3.08 (tt, *J* = 10.2, 4.0 Hz, 1H), 2.08 (d, *J* = 10.5 Hz, 1H), 1.97 (d, *J* = 13.8 Hz, 1H), 1.83–1.75 (m, 1H), 1.70–1.53 (m, 2H), 1.23 (s, 12H), 1.20–1.07 (m, 3H), 1.00–0.85 (m, 1H). (Note: the integration values reported were adjusted for the presence of overlapping signals from cyclohexane-Bpin.) ^13^C{^1^H} NMR (126 MHz, CDCl_3_) *δ* 83.0, 80.0, 55.6, 32.9, 32.4, 27.1, 25.7, 24.8; ^11^B NMR (160 MHz, CDCl_3_) *δ* 33.8; HRMS (ESI) *m*/*z* calcd for C_13_H_25_BO_3_Na [M + Na]^+^˙ 263.1794 found 263.1806. NMR data match those previously reported.^26^*Cis* and *trans* isomer assignments were confirmed by oxidation of the Bpin and comparing the spectra of the alcohols to those previously reported.^76^ The proton on the OH bearing carbon appeared at 3.69 ppm (dp, *J* = 8.1, 3.6 Hz) (lit. ^76^ 3.71 ppm (tt, *J* = 8.2, 3.9 Hz)) for the *cis* isomer and at 3.99 ppm (tt, *J* = 7.9, 3.7 Hz) (lit. ^76^ 4.02 ppm (tt, *J* = 7.9, 3.8 Hz)) for the *trans* isomer. The *cis* and *trans* ratios after oxidation was found to be 3.3 : 1.

##### *Cis*-1,2-bis(4,4,5,5-tetramethyl-1,3,2-dioxaborolan-2-yl)cyclohexane (2v)

General procedure B was followed with 1,2-bis(4,4,5,5-tetramethyl-1,3,2-dioxaborolan-2-yl)benzene (1v) (165 mg, 0.5 mmol), 5% Rh/C (25 mg, 2 mol%) for 6 h. After filtration solvent was removed under reduced pressure, mixture purified over flash column chromatography (hexane as eluent) and concentrated and dried over rotovap to give colorless oil (106 mg, 88% yield). ^1^H NMR (500 MHz, CDCl_3_) *δ* 1.69–1.49 (m, 5H), 1.49–1.35 (m, 5H), 1.22 (s, 24H); ^13^C{^1^H} NMR (126 MHz, CDCl_3_) *δ* 82.9, 28.2, 27.0, 25.0, 24.9; ^11^B NMR (160 MHz, CDCl_3_) *δ* 34.0. NMR data were consistent with those previously reported.^50^

##### 2-(Trimethylsilyl)piperidin-1-ium-(+)-camphorsulfonate (2w)

General procedure B was followed with 2-bromo-6-(trimethylsilyl)pyridine (115 mg, 0.5 mmol), 5% Rh/C (25 mg, 0.2 mol%), (+)-camphor sulfonic acid (CS) (116 mg, 0.5 mmol). After purification, solvent was removed under reduced pressure to give a colorless oil (181 mg, 76% yield). ^1^H NMR (500 MHz, D_2_O) *δ* 3.32–3.22 (m, 1H), 3.17 (d, *J* = 14.8 Hz, 1H, CS), 2.84 (td, *J* = 13.0, 2.4 Hz, 1H), 2.74 (d, *J* = 14.9 Hz, 1H, CS), 2.62–2.52 (m, 1H), 2.41–2.16 (m, 2H, CS), 2.06 (t, *J* = 4.5 Hz, 1H, CS), 1.99–1.90 (m, 1H, CS), 1.90–1.85 (m, 1H, CS), 1.85–1.73 (m, 3H), 1.66–1.28 (m, 5H, CS + compound), 0.94 (s, 3H, CS), 0.73 (s, 3H, CS), 0.04 (s, 9H); ^13^C{^1^H} NMR (126 MHz, D_2_O) *δ* 221.6 (CS), 58.3 (CS), 48.2, 48.0 (CS), 47.0 (CS), 46.4, 42.4 (CS), 42.1 (CS), 26.1 (CS), 24.3 (CS), 24.1, 22.8, 22.2, 18.8 (CS), 18.6 (CS), −4.9; ^29^Si NMR (99 MHz, D_2_O) *δ* 2.7; mp 122–124 °C; GCMS calcd for C_8_H_19_NSi [M − CS–H]^+^˙ 157.13 found 157.15.

##### 2-(Trifluoromethyl)-4-(trimethylsilyl)piperidine (2x)

General procedure B was followed with 2-chloro-6-(trifluoromethyl)-4-(trimethylsilyl)pyridine (1x) (44 mg, 0.17 mmol), 5% Rh/Al_2_O_3_ (20 mg, 4.5 mol%). After purification, the solvent was removed by rotary evaporation. Adding ethyl acetate formed a white solid precipitate which was dried under vacuum (44 mg, 92% yield). ^1^H NMR (500 MHz, D_2_O) *δ* 4.02–3.92 (m, 1H), 3.57 (dd, *J* = 12.5, 4.0 Hz, 1H), 3.07 (td, *J* = 13.0, 3.3 Hz, 1H), 2.19 (d, *J* = 14.0 Hz, 1H), 1.94 (d, *J* = 14.8 Hz, 1H), 1.67–1.45 (m, 2H), 1.00 (tt, *J* = 13.3, 3.4 Hz, 1H), 0.01 (s, 9H); ^13^C{^1^H} NMR (126 MHz, D_2_O) *δ* 123.1 (d, *J* = 280.4 Hz), 57.1 (q, *J* = 31.5 Hz), 46.1, 22.5, 22.1, 19.8, −5.1; ^19^F NMR (470 MHz, D_2_O) *δ* −75.47 (d, *J* = 6.6 Hz); ^29^Si NMR (99 MHz, D_2_O) *δ* 3.6; mp: decomposes at 237 °C; HRMS (ESI) *m*/*z* calcd for C_9_H_19_F_3_NSi [M − Cl]^+^˙ 226.1238 found 226.1239. Stereochemistry was ascertained by 2D NMR and the *cis* geometry determined by a 1D-NOE experiment. Irradiation of the proton at the carbon with a CF_3_ substituent showed enhancement of the proton at the carbon bearing TMS confirming the *cis* assignment.

##### 2-(Trifluoromethyl)-5-(trimethylsilyl)piperidine (2y)

General procedure B was followed with 2-chloro-5-(trifluoromethyl)-4-(trimethylsilyl)pyridine (1y) (126 mg, 0.5 mmol), 5% Rh/C (25 mg, 2 mol%). After purification, solvent was removed by rotary evaporation to yield a yellow oil (90 mg (mixture), 15% yield of 2y). A 5 : 1 ratio of desilylated to silylated product was observed in the ^1^H NMR, which was confirmed by LCMS (226.3 *m*/*z* for silylated material and 154.1 *m*/*z* for desilylated material) and ^19^F NMR. Spectral data of the mixture (2y + desilylated 2y): ^1^H NMR (500 MHz, D_2_O) *δ* 4.46–4.14 (m, 1H), 4.09–3.97 (m, 5H), 3.53 (d, *J* = 12.96, 5H), 3.40 (dd, *J* = 13.0, 4.2 Hz, 1H), 3.21–3.01 (m, 6H), 2.22 (s, 1H), 2.21–2.12 (m, 5H), 2.03–1.85 (m, 12H), 1.82–1.50 (m, 18H), 1.27–1.09 (m, 1H), 0.01 (d, *J* = 1.2 Hz, 9H); ^13^C{^1^H} NMR (126 MHz, D_2_O) *δ* 123.75, 51.4, 51.2, 45.1, 43.6, 30.1, 21.4, 21.4, 21.3, 21.0, 20.6, 20.1, 17.7, −4.9; ^19^F NMR (470 MHz, D_2_O) *δ* −68.51 (d, *J* = 8.9 Hz), −75.55 (d, *J* = 6.8 Hz). ^1^H-NMR data for the desilylated compound matched those previously reported.^77^^1^H NMR (500 MHz, D_2_O) 4.09–3.97 (m, 1H), 3.53 (d, *J* = 13.0, 1H), 3.21–3.01 (m, 1H), 2.21–2.12 (m, 1H), 2.03–1.85 (m, 2H), 1.82–1.50 (m, 3H);^19^F NMR (470 MHz, D_2_O) *δ* −75.6 (d, *J* = 6.7 Hz).

##### 3-(2,3-Dihydrobenzofuran-2-yl)-1,1,1,3,5,5,5-heptamethyltrisiloxane (2z)

General procedure B was followed with 3-(benzofuran-2-yl)-1,1,1,3,5,5,5-heptamethyltrisiloxane (1z) (169 mg, 0.5 mmol), 10% Pd/C (20 mg, 3 mol%), ethanol (5 mL) for 16 h. After purification, the solvent was removed by rotary evaporation to yield a yellowish oil (139 mg, 82% yield); ^1^H NMR (500 MHz, C_6_D_6_) *δ* 7.04 (d, *J* = 7.3 Hz, 1H), 7.01–6.94 (m, 1H), 6.87–6.81 (m, 1H), 6.77 (td, *J* = 7.4, 1.0 Hz, 1H), 4.14 (t, *J* = 11.1 Hz, 1H), 3.15 (dd, *J* = 15.0, 11.4 Hz, 1H), 3.03 (dd, *J* = 15.0, 10.8 Hz, 1H), 0.21 (s, 3H), 0.16 (s, 9H), 0.10 (s, 9H); ^13^C{^1^H} NMR (126 MHz, C_6_D_6_) *δ* 161.2, 124.7, 120.0, 109.6, 75.0, 31.1, 1.5, 1.4, −2.9; ^29^Si NMR (99 MHz, CDCl_3_) *δ* 9.1, 9.3, −31.62; HRMS (APCI) *m*/*z* calcd for C_15_H_29_O_3_Si_3_ [M]^+^˙ 341.1425 found 341.1428.

##### Cyclohexyltriethoxysilane (2aa)^54^

General procedure B was followed with triethoxy(phenyl)silane (120 mg, 0.5 mmol), 5% Rh/Al_2_O_3_ catalyst (25 mg, 2 mol%). After purification, the solvent was removed by rotary evaporation to yield a colorless oil (95.8 mg, 78% yield). Per Reaxys the only IR data were previously reported.^78^^1^H NMR (500 MHz, CDCl_3_) *δ* 3.82 (q, *J* = 7.0 Hz, 6H), 1.77 (d, *J* = 11.4 Hz, 2H), 1.70 (d, *J* = 12.1 Hz, 2H), 1.67 (s, 1H), 1.32–1.25 (m, 2H), 1.22 (t, *J* = 7.0 Hz, 12H), 0.81 (tt, *J* = 12.5, 3.1 Hz, 1H); ^13^C{^1^H} NMR (126 MHz, CDCl_3_) *δ* 58.6, 27.9, 27.0, 26.9, 23.0, 18.5; ^29^Si NMR (99 MHz, CDCl_3_) *δ* 48.5; GCMS for C_12_H_26_O_3_Si calcd [M]^+^˙ 246.17 found 246.10.

##### *tert*-Butyl-4-(4,4,5,5-tetramethyl-1,3,2-dioxaborolan-2-yl)-2(trimethylsilyl)pyrrolidine-1-carboxylate (2ab)

General procedure B was followed with *tert*-butyl 4-(4,4,5,5-tetramethyl-1,3,2-dioxaborolan-2-yl)-2-(trimethylsilyl)-1*H*-pyrrole-1-carboxylate (1ab) (182 mg, 0.5 mmol), 5% Rh/C (25 mg, 2 mol%). After 16 h, the reaction had run to 40% conversion of 1ab and was worked up. After filtration, solvent was removed by rotary evaporation. The resulting mixture was purified by silica flash column chromatography. Fractions containing the desired product were combined and the volatiles evaporated to yield a white solid (66 mg, 36% yield, 96 : 4 dr). Increasing the stirring speed from 300 rpm to 1000 rpm did not affect conversion. Increasing the pressure to 72 atm from 48 atm gave a 66% conversion and 60% yield. The reaction showed full conversion after 48 h with 4 mol% catalyst and 93–100% yields were obtained. The *cis* configuration of the compound was confirmed by 2D NMRs, COSY and 2D NOESY NMR. By 2D NOESY NMR, the peak at 2.93 ppm (H at C α to N with TMS) showed a stronger correlation with the neighboring proton at 1.96 ppm and a weaker correlation with the neighbor at 1.72 ppm. The peak at 1.96 ppm showed a correlation with the proton at 1.44 ppm whereas no correlation was observed with the proton at 1.72 ppm. Characterization of the major *cis* diastereomer: ^1^H NMR (500 MHz, C_6_D_6_) *δ* 3.85 (t, *J* = 9.6 Hz, 1H), 3.40 (t, *J* = 11.4 Hz, 1H), 2.97–2.87 (m, 1H), 2.00–1.89 (m, 1H), 1.72 (q, *J* = 12.6 Hz, 1H), 1.41 (s, 10H), 0.95 (s, 12H), 0.20 (s, 9H); ^13^C{^1^H} NMR (126 MHz, C_6_D_6_) *δ* 154.3, 82.8, 77.8, 50.1, 49.3, 32.3, 28.3, 24.4, −1.8; ^11^B NMR (160 MHz, C_6_D_6_) *δ* 33.4; ^29^Si NMR (99 MHz, C_6_D_6_) *δ* 1.7; mp 85–87 °C. HRMS (ESI) *m*/*z* calcd for C_18_H_36_BNO_4_Si [M]^+^˙ 369.2506 found 369.2510.

##### 4-(4,4,5,5-Tetramethyl-1,3,2-dioxaborolan-2-yl)-2-(trimethylsilyl)piperidin-1-ium bromide (2ac)

General procedure B was followed with 2-bromo-4-(4,4,5,5-tetramethyl-1,3,2-dioxaborolan-2-yl)-6-(trimethylsilyl)pyridine (1ac) (178 mg, 0.5 mmol), 5% Rh/C (25 mg, 2 mol%). After purification, solvent was concentrated by rotary evaporation and the product precipitated by adding ethyl acetate. After drying under vacuum a white solid was obtained (83 mg, 46% yield). Structure determination was made by 2D NMRs and the *cis* stereochemistry was ascertained by 1D NOE. ^1^H NMR (500 MHz, D_2_O) *δ* 3.22 (d, *J* = 12.4 Hz, 1H), 2.78 (dt, *J* = 12.79 Hz, 2.45 Hz, 1H), 2.52 (dd, *J* = 13.2 Hz, 2.45, 1H), 1.80 (d, *J* = 14.4 Hz, 2H), 1.56–1.33 (m, 2H), 1.12 (s, 12H), 1.09 (s, 1H), −0.02 (s, 9H); ^13^C{^1^H} NMR (126 MHz, D_2_O) *δ* 75.5, 48.6, 46.7, 25.7, 23.7, 23.6, −5.01; ^11^B NMR (160 MHz, D_2_O) *δ* 31.2; ^29^Si NMR (99 MHz, D_2_O) *δ* 2.7; mp: decomposes at 230 °C; HRMS (ESI) *m*/*z* calcd for C_14_H_31_BNSiO_2_ calcd [M − Br]^+^˙ 284.2217 found 284.2220.

##### *Cis*-trimethylsilyl-2-(4,4,5,5-tetramethyl-1,3,2-dioxaborolan-2-yl)cyclohexane (2ad)

General procedure B was followed with trimethyl(2-(4,4,5,5-tetramethyl-1,3,2-dioxaborolan-2-yl)phenyl)silane (1ad) (138 mg, 0.5 mmol), 5% Rh/C (25 mg, 2 mol%) at 48 atm pressure for 6 h. After purification, solvent was removed under reduced pressure and the residue purified by flash column chromatography (diethyl ether/hexane 1–10% gradient). Fractions containing the desired product were combined and the volatiles evaporated to give a colorless oil (86% conversion, 17% yield). ^1^H NMR (500 MHz, CDCl_3_) *δ* 1.92–1.83 (m, 1H), 1.72 (dtt, *J* = 12.8, 3.6, 1.8 Hz, 1H), 1.65–1.58 (m, 1H), 1.58–1.50 (m, 1H), 1.48–1.25 (m, 4H), 1.24 (d, *J* = 3.6 Hz, 12H), 1.19 (dt, *J* = 12.3, 3.8 Hz, 1H), 0.71 (dt, *J* = 12.7, 3.3 Hz, 1H), −0.04 (s, 9H); ^13^C{^1^H} NMR (126 MHz, CDCl_3_) *δ* 82.9, 31.1, 28.9, 26.3, 25.8, 25.1, 25.0, −1.8; ^11^B NMR (160 MHz, CDCl_3_) *δ* 34.1; ^29^Si NMR (99 MHz, CDCl_3_) *δ* 1.9. GCMS calcd for C_14_H_28_BO_2_Si [M − Me]^+^˙ 267.20 found 267.20. NMR data and the stereochemical assignment were consistent with those previously reported.^22^

##### Trimethyl(3-(4,4,5,5-tetramethyl-1,3,2-dioxaborolan-2-yl)cyclohexyl)silane (2ae)

General procedure B was followed with trimethyl(3-(4,4,5,5-tetramethyl-1,3,2-dioxaborolan-2-yl)phenyl)silane (1ae) (138 mg, 0.5 mmol), 5% Rh/C (25 mg, 2 mol%). After purification, the solvent was removed by rotary evaporation and compound obtained as a colorless liquid (141 mg (with ∼7% desilylated material observed *via* GC-MS)), which corresponded to 133.5 mg of 2ae (95% yield). The major diastereomer was identified by oxidizing the boronic ester. The peak for the proton α to the OH in the *cis* isomer was observed at 3.55 ppm (sept) (lit. ^79^ 3.42 ppm, sept) and the peak for proton α to OH was observed at 4.0 ppm (bs) (lit. ^79^ 3.96 ppm (bs)) for the *trans* diastereomer. *Cis*/*trans* ratios were before (2.1 : 1) and after (2.5 : 1) oxidation were comparable. Data of the mixture: ^1^H NMR (500 MHz, CDCl_3_) *δ* 1.91–1.53 (m, 6H), 1.42–1.27 (m, 2H), 1.27–1.20 (m, 14H), 1.19 (d, *J* = 10 Hz, 1H), 1.12–0.98 (m, 2H), 0.89 (t, *J* = 11.7 Hz, 1H), 0.53 (t, *J* = 12.6 Hz, 1H),–0.07 (s, 9H); ^13^C{^1^H} NMR (126 MHz, CDCl_3_) *δ* 82.9, 82.8, 28.7, 28.7, 28.3, 28.3, 28.1, 28.0, 27.5, 27.3, 27.1, 26.9, 25.2, 25.2, 24.9, 24.9, 24.8, −3.3, −3.4; ^11^B NMR (160 MHz, CDCl_3_) *δ* 34.0; ^29^Si NMR (99 MHz, CDCl_3_) *δ* 2.1, 2.3; GCMS for C_14_H_28_BO_2_Si [M − Me]^+^˙ calcd 267.20 found 267.20. The NMR data were consistent with those previously reported.^22^

##### Trimethyl(4-(4,4,5,5-tetramethyl-1,3,2-dioxaborolan-2-yl)cyclohexyl)silane (2af)

General procedure B was followed with trimethyl(4-(4,4,5,5-tetramethyl-1,3,2-dioxaborolan-2-yl)phenyl)silane (1af) (138 mg, 0.5 mmol), 5% Rh/C (25 mg, 2 mol%) at 48 atm pressure for 8.5 h. After purification, solvent was removed under reduced pressure to obtain a colorless oil (138 mg, 98% yield). A 4.5 : 1 *cis* to *trans* ratio was observed by ^1^H NMR and 6 : 1 *via* GC. Data for the major *cis* diastereomer: ^1^H NMR (500 MHz, CDCl_3_) *δ* 1.95–1.86 (m, 2H), 1.64–1.55 (m, 2H), 1.41–1.30 (m, 3H), 1.25 (s, 12H), 1.15 (qd, *J* = 12.8, 3.3 Hz, 2H), 0.56–0.47 (m, 1H), −0.09 (s, 9H). ^13^C{^1^H} NMR (126 MHz, CDCl_3_) *δ* 83.0, 29.2, 26.2, 25.9, 25.0, −3.5. ^11^B NMR (160 MHz, CDCl_3_) *δ* 34.6; ^29^Si NMR (99 MHz, CDCl_3_) *δ* 2.4, 2.2. ^1^H-NMR data were consistent with those previously reported.^22^ (Note: the major diastereomer was identified by oxidizing the boronic ester. The differentiable peak for the proton α to the OH in the *cis* isomer was observed at 4.05 ppm (s, 1H) (lit. ^80^ 4.07 ppm (s, 1H))). The proton α to the OH in the *trans* isomer was observed at 3.54–3.46 ppm (m, 1H) (lit. ^80^ 3.58–3.52 ppm (m, 1H)). By ^1^H NMR the *cis* : *trans* ratios were comparable before (4.5 : 1) and after (4.4 : 1) oxidation.

## Data availability

All underlying data is available in the article itself and its ESI.†

## Conflicts of interest

The authors declare the following competing financial interest(s): M. R. S. and R. E. M. own a percentage of BoroPharm, Inc.

## Supplementary Material

RA-014-D4RA00491D-s001
