# Supplementary material for: Access to C(sp3) borylated and silylated cyclic molecules: hydrogenation of corresponding arenes and heteroarenes
Source: RSC Adv. 2024 Apr 2;14(15):10590–607. doi: 10.1039/d4ra00491d (PMC10985595; doi:10.1039/d4ra00491d)

Electronic Supplementary Material (ESI) for RSC Advances.
This journal is © The Royal Society of Chemistry 2024

Supporting Information

Access to C(sp^3) borylated and silylated cyclic molecules: Hydrogenation of corresponding cycloarenes and heteroarenes

Arzoo Chhabra, Sabrina Reich, Timothy M. Shannon, Robert E. Maleczka Jr.*; Milton R. Smith, III

**Department of Chemistry, Michigan State University, 578 S Shaw Ln, East Lansing, Michigan, 48824, USA.*
maleczka@chemistry.msu.edu;

Table of contents

Table S1 and S2 Additives for hydrogenation of **1a.**……………………………………………………………………………….S2

^{1}H, ^{13}C, ^{11}B, ^{18}F, ^{29}Si NMR spectra……………………………………………….……………………………………….S4- S86

Table S1 Additives for the hydrogenation of 1a by Rh/C [a]

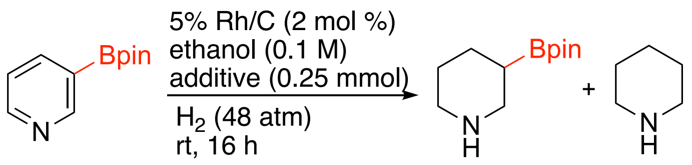

Entry	Additive (1 equiv)	Time (h)	1a (%)	2a (%)	1a' (%)
1	HCl	16	75	25	0
2	HCl (dry)	1	72	28	0
3	HCl + 1 mL H_2O	1	71	28	1
4	$MeSO_3H$	2	63	37	0
5	NH_4Cl	24	0	70	30
6	H_3PO_4	2	58	42	0
7	2,6-Dichloropyridine	36	38	62	0
8	Sulfuric Acid	2	39	61	0
9	Triflic acid	2	52	48	0

[a]Relative composition determined by ^{1}H-NMR
(4,4,5,5-tetramethyl1,3,2-dioxaborolan-2-yl)pyridine (0.25 mmol), Additive (0.25 mmol), Hydrogen gas (48 atm), ethanol (2 mL), 5% Rh/C (10 mg), rt

Table S2 Additives for the hydrogenation of 1a by Rh/C [a]

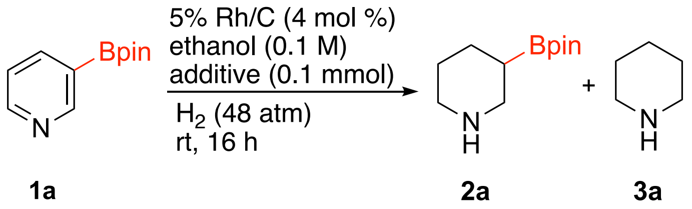

Entry	Additive (1 equiv)	1a (%)	2a (%)	1a’
1	Cerium (III) chloride	0	100	0
2	Magnesium chloride	0	30	70
3	Copper sulfate	20	18	62
4	Potassium Iodide	16	42	42
5	Zinc chloride	0	15	85
6	Iron powder (rusted)	0	15	85
7	Magnesium sulfate	0	17	83
8	Trimethyl borate	0	11	89
9	3Å molecular sieves	0	19	81

a) Conversion percentages determined by GCMS by comparing piperidine to starting material. 3-(4,4,5,5-tetramethyl-1,3,2-dioxaborolan-2-yl)pyridine (0.1 mmol), Additive (0.1 mmol), Hydrogen gas (700 psi), Ethanol (1 mL), 5% Rh/C (10 mg), rt.

^{1}H NMR (500MHz, $CDCl_3$) of 2,6-dibromo-4-(4,4,5,5-tetramethyl-1,3,2-dioxaborolan-2-yl)pyridine (1d)

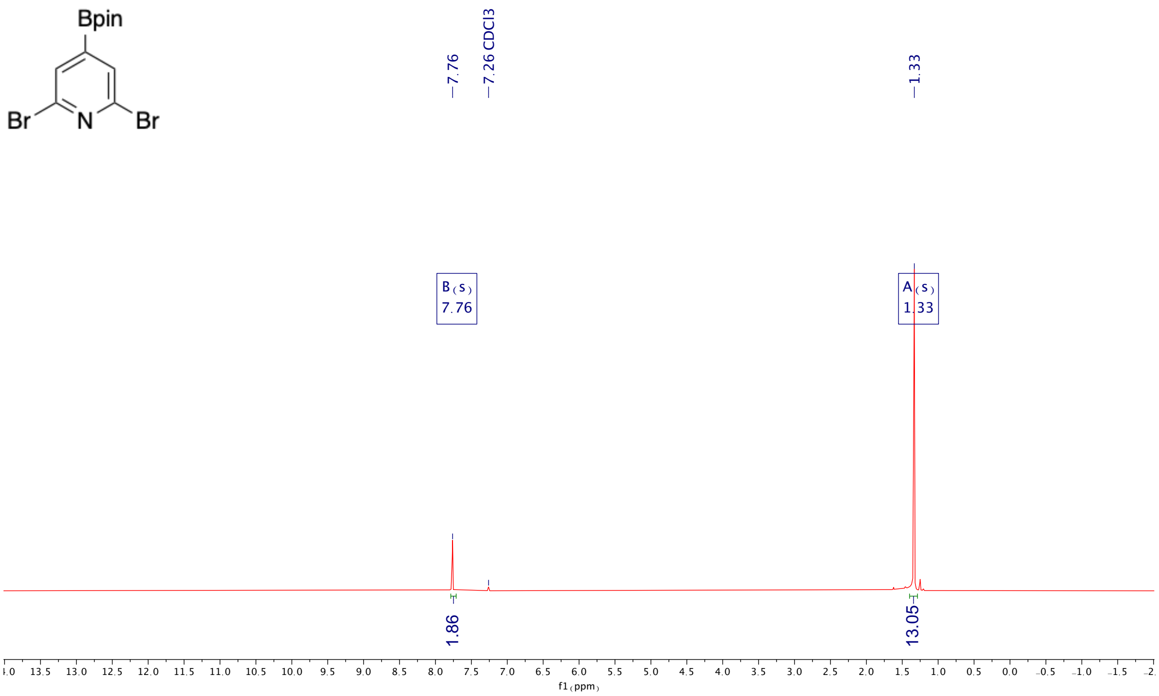

$^{13}C\{^{1}H\}$ (126 MHz, $CDCl_3$) NMR of 2,6-dibromo-4-(4,4,5,5-tetramethyl-1,3,2-dioxaborolan-2-yl)pyridine (1d)

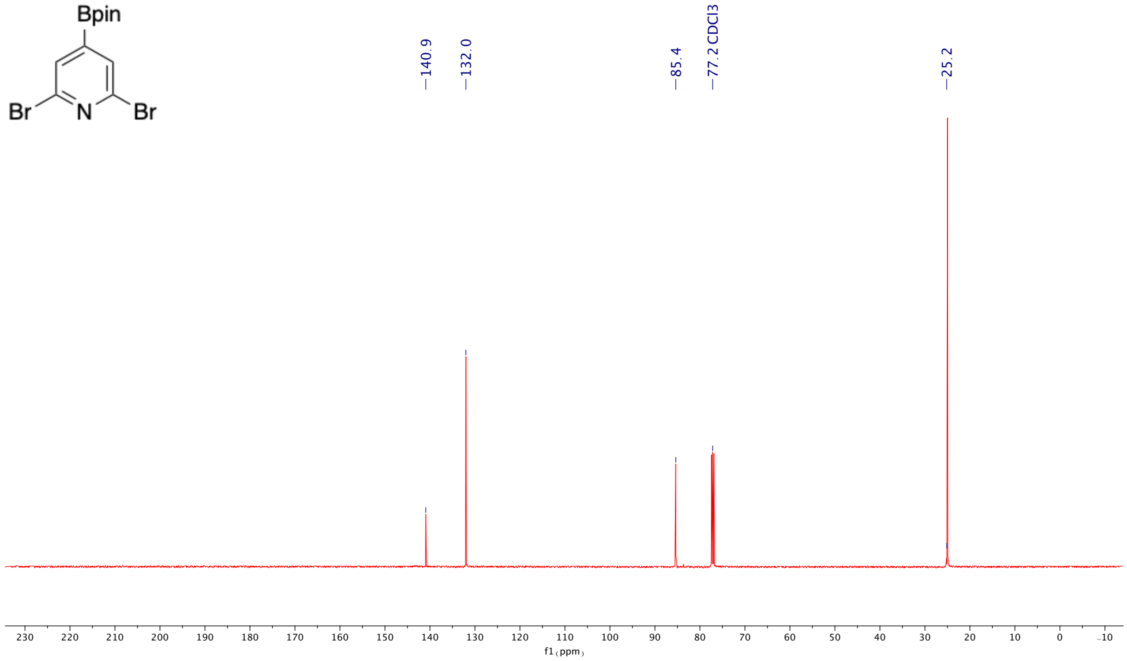

^{11}B NMR (160MHz, $CDCl_3$) of 2,6-dibromo-4-(4,4,5,5-tetramethyl-1,3,2-dioxaborolan-2-yl)pyridine (1d)

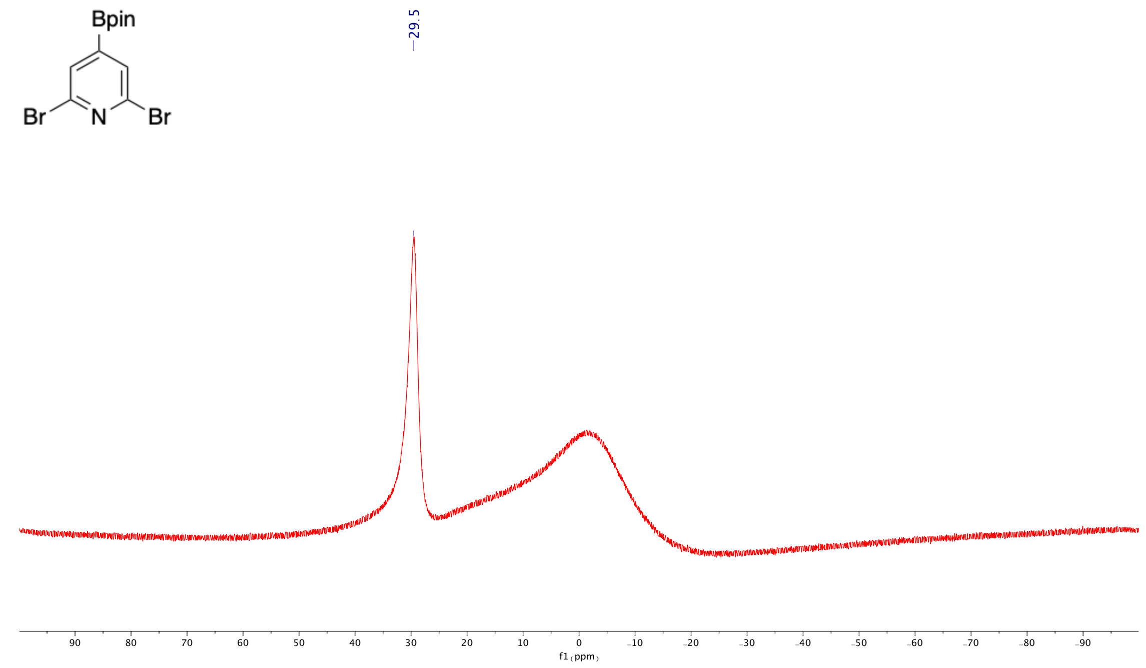

^{1}H NMR (500MHz, $CDCl_3$) of 2-bromo-6-methyl-4-(4,4,5,5-tetramethyl-1,3,2-dioxaborolan-2-yl)pyridine (1e)

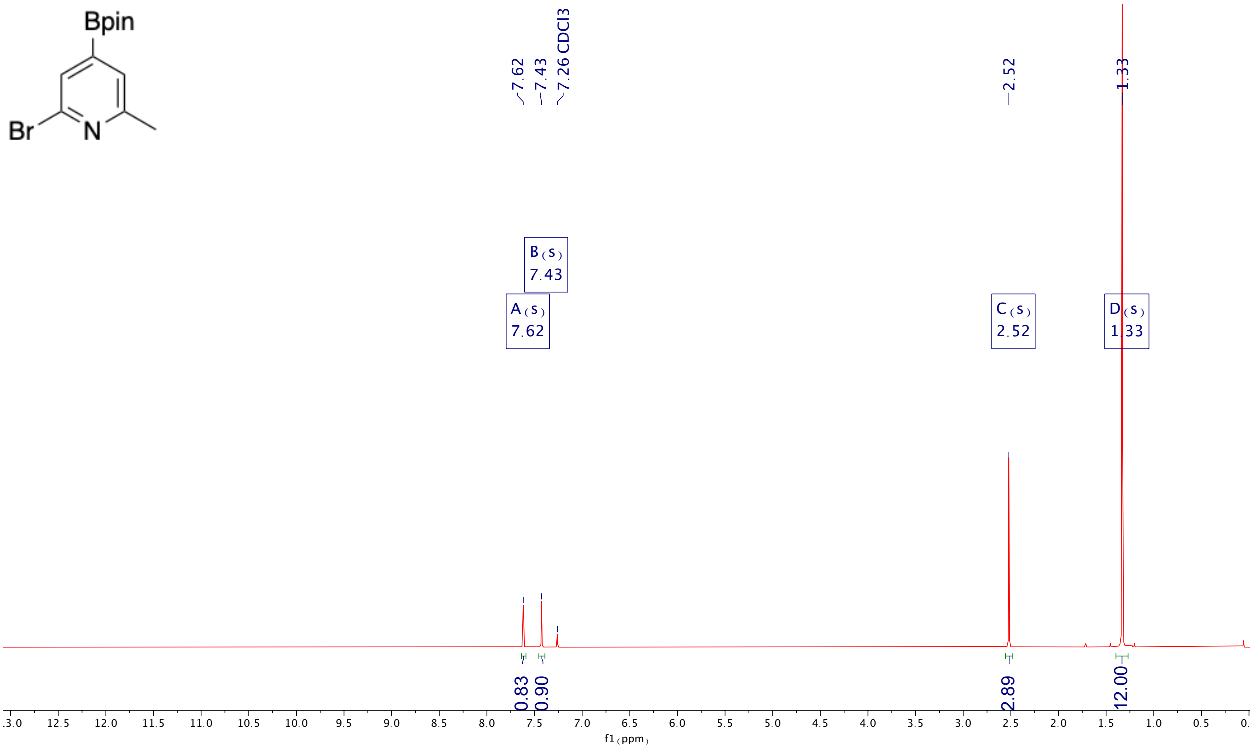

$^{13}C\{^{1}H\}$ (126 MHz, $CDCl_3$) NMR of 2-bromo-6-methyl-4-(4,4,5,5-tetramethyl-1,3,2-dioxaborolan-2-yl)pyridine (1e)

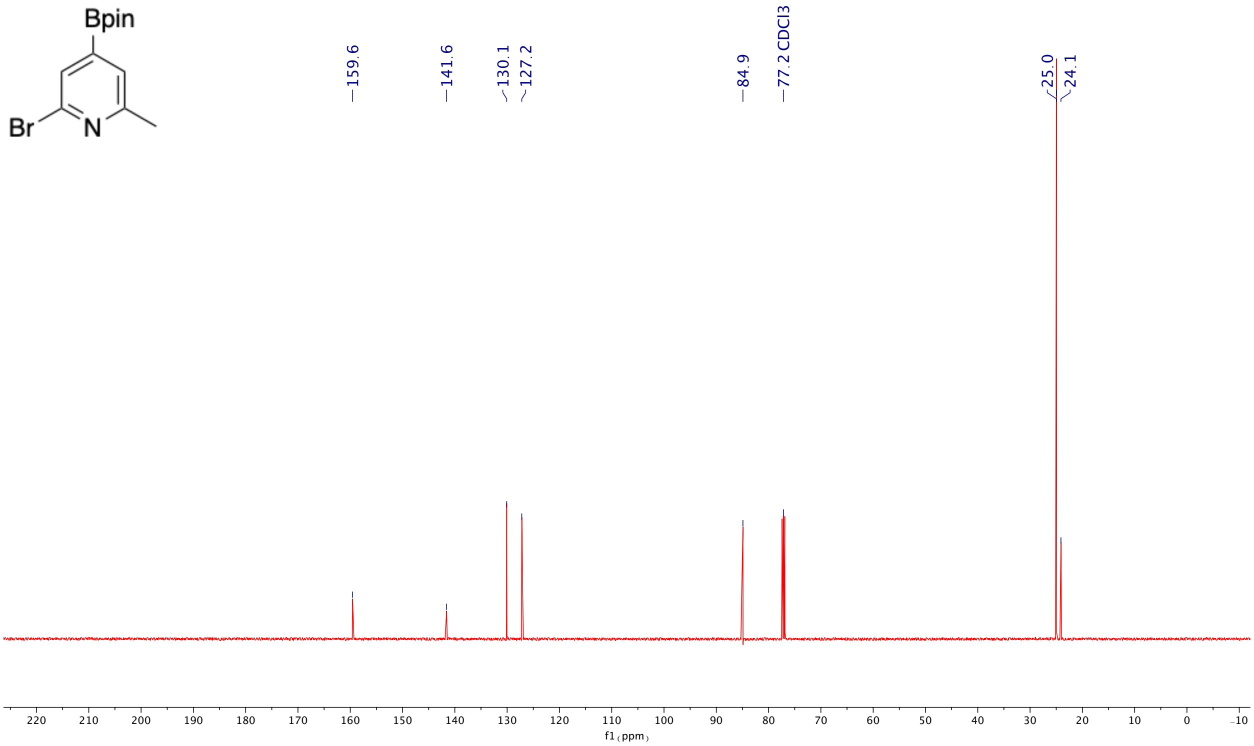

^{11}B NMR (160MHz, $CDCl_3$) of 2-bromo-6-methyl-4-(4,4,5,5-tetramethyl-1,3,2-dioxaborolan-2-yl)pyridine (1e)

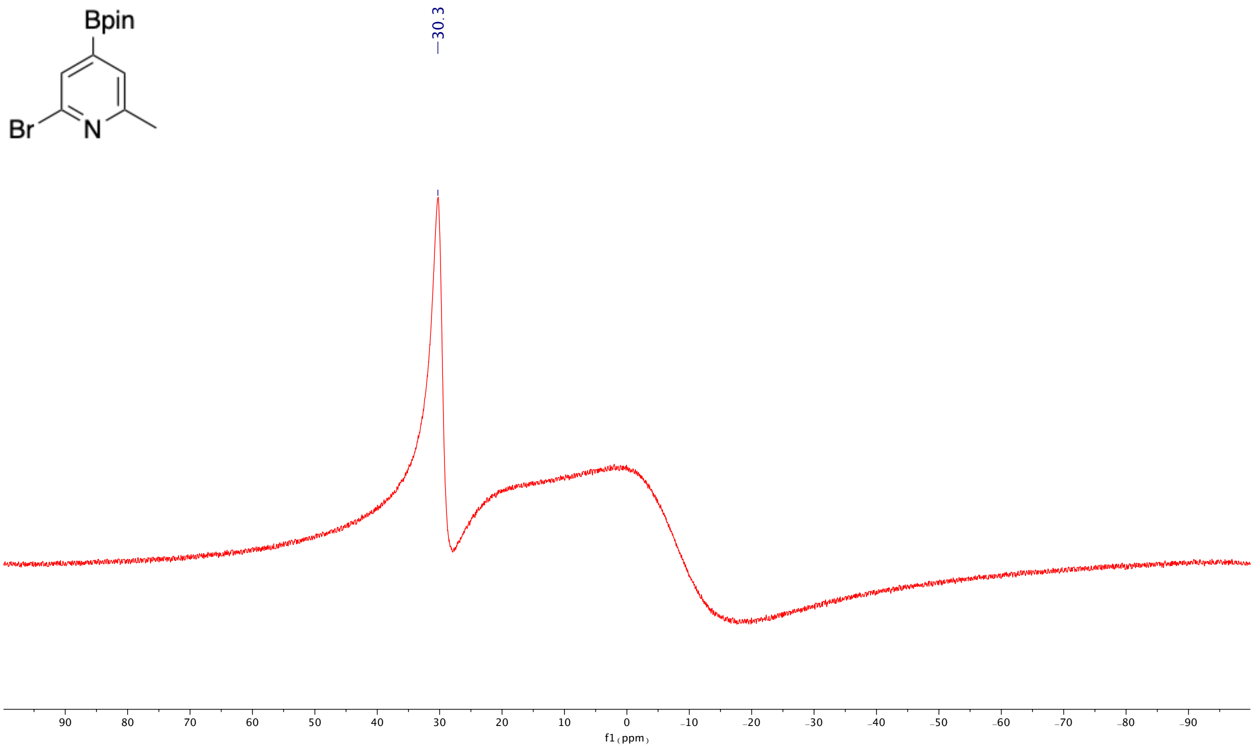

^{1}H NMR (500MHz, $CDCl_3$) of 2,6-dimethyl-4-(4,4,5,5-tetramethyl-1,3,2-dioxaborolan-2-yl)pyridine (1f)

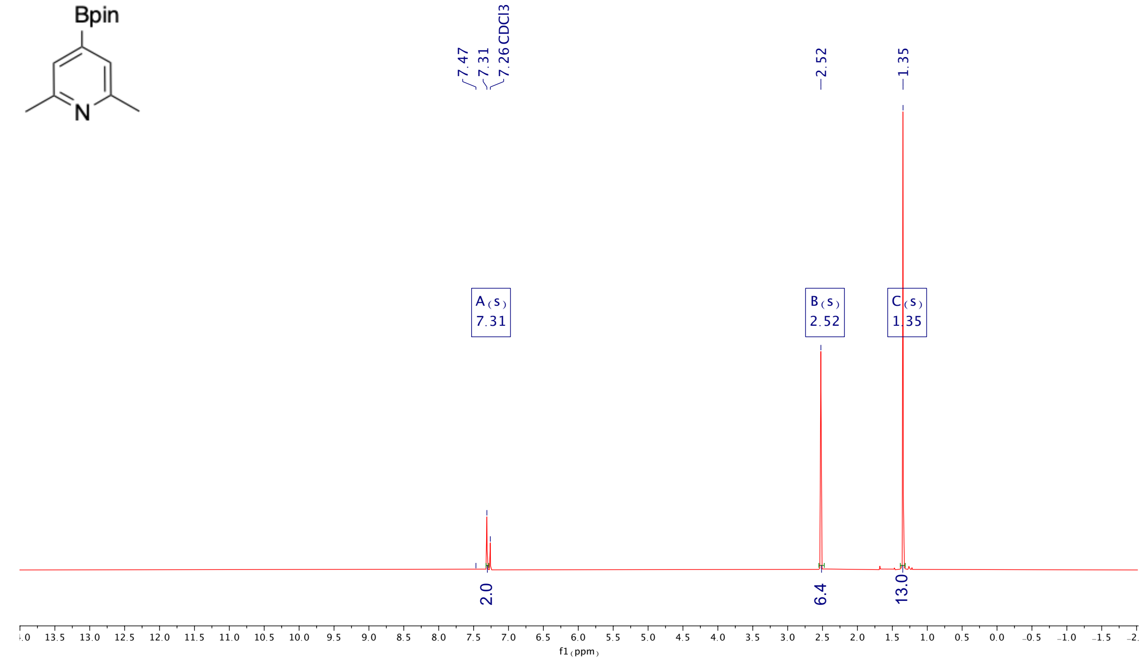

$^{13}C\{^{1}H\}$ (126 MHz, $CDCl_3$) NMR of 2,6-dimethyl-4-(4,4,5,5-tetramethyl-1,3,2-dioxaborolan-2-yl)pyridine (1f)

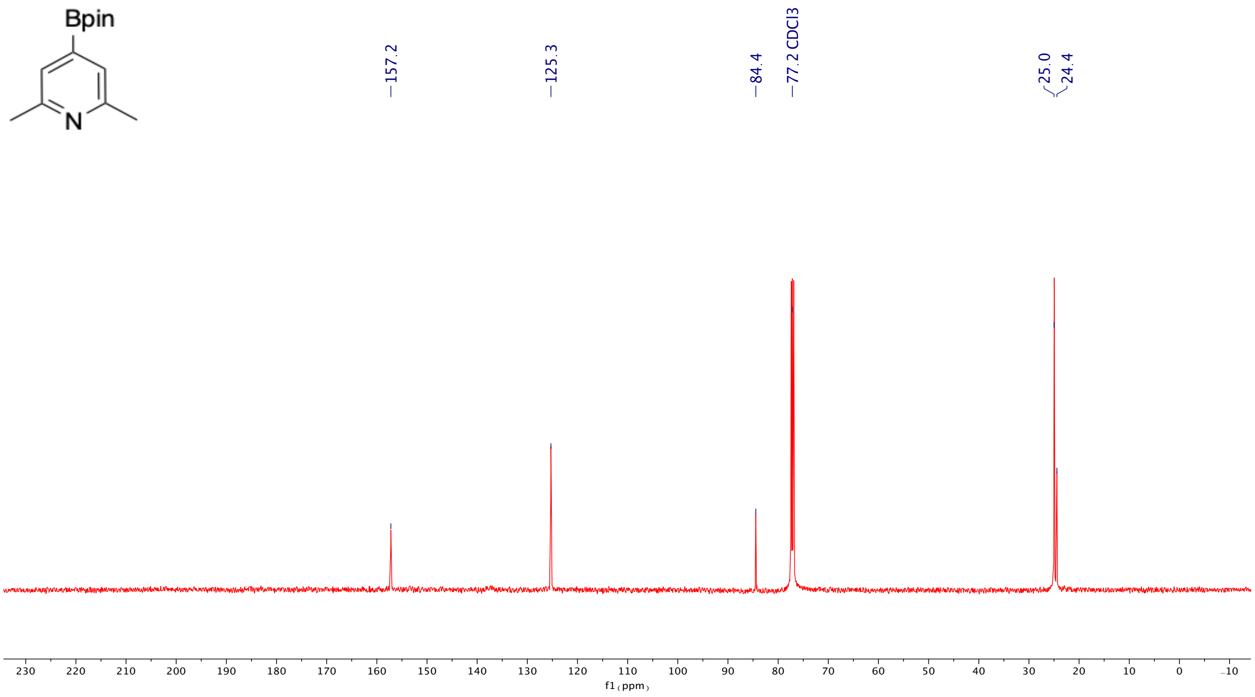

^{11}B NMR (160MHz, $CDCl_3$) of 2,6-dimethyl-4-(4,4,5,5-tetramethyl-1,3,2-dioxaborolan-2-yl)pyridine (1f)

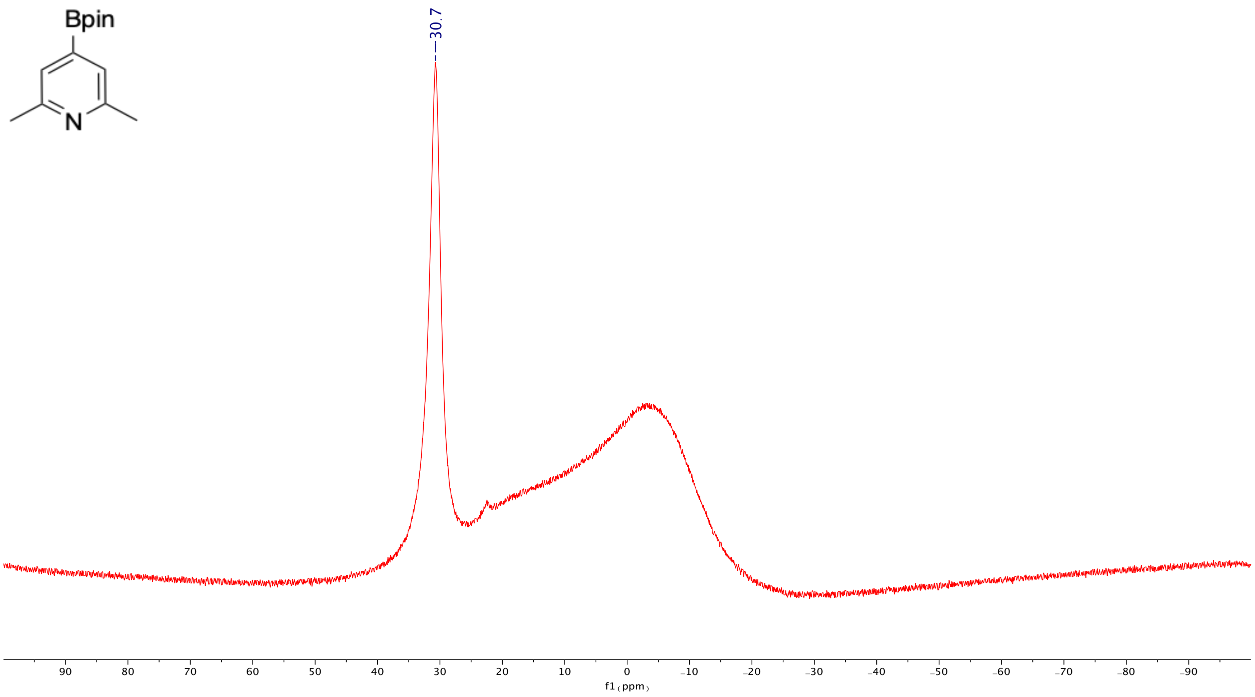

^1H NMR (500MHz, $CDCl_3$) of *Tert*-butyl 3-(4,4,5,5-tetramethyl-1,3,2-dioxaborolan-2-yl)-1*H*-pyrrole-1-carboxylate (1g)

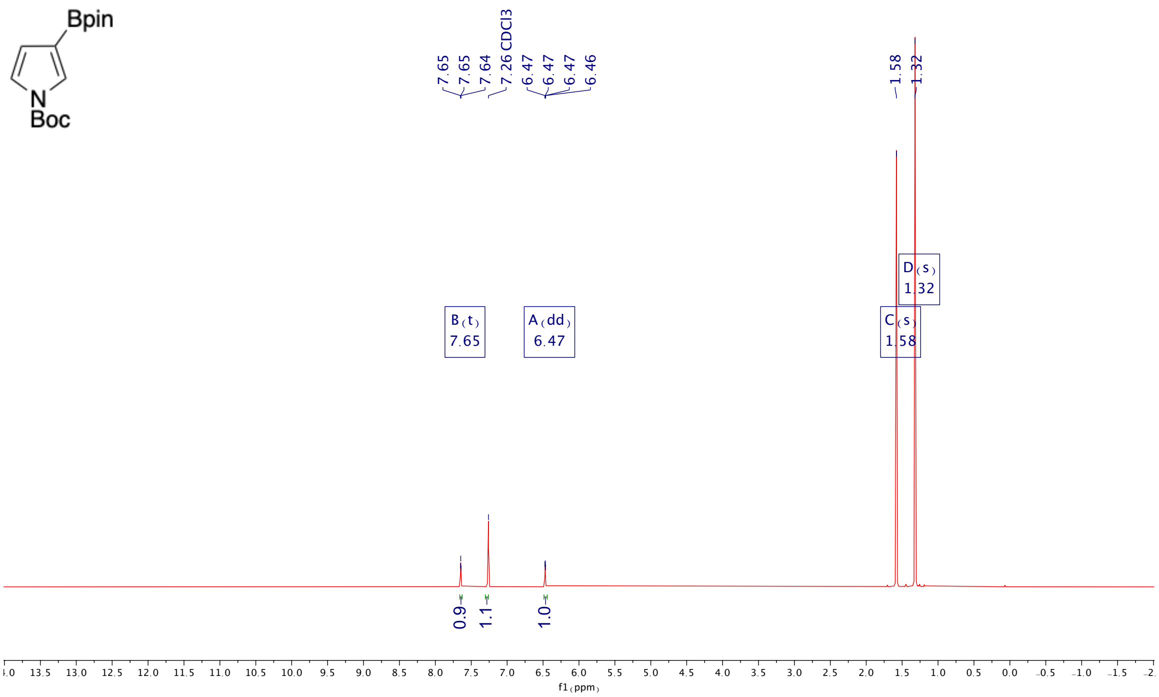

$^{13}C\{^{1}H\}$ (126 MHz, $CDCl_3$) NMR of *Tert*-butyl 3-(4,4,5,5-tetramethyl-1,3,2-dioxaborolan-2-yl)-1*H*-pyrrole-1-carboxylate (1g)

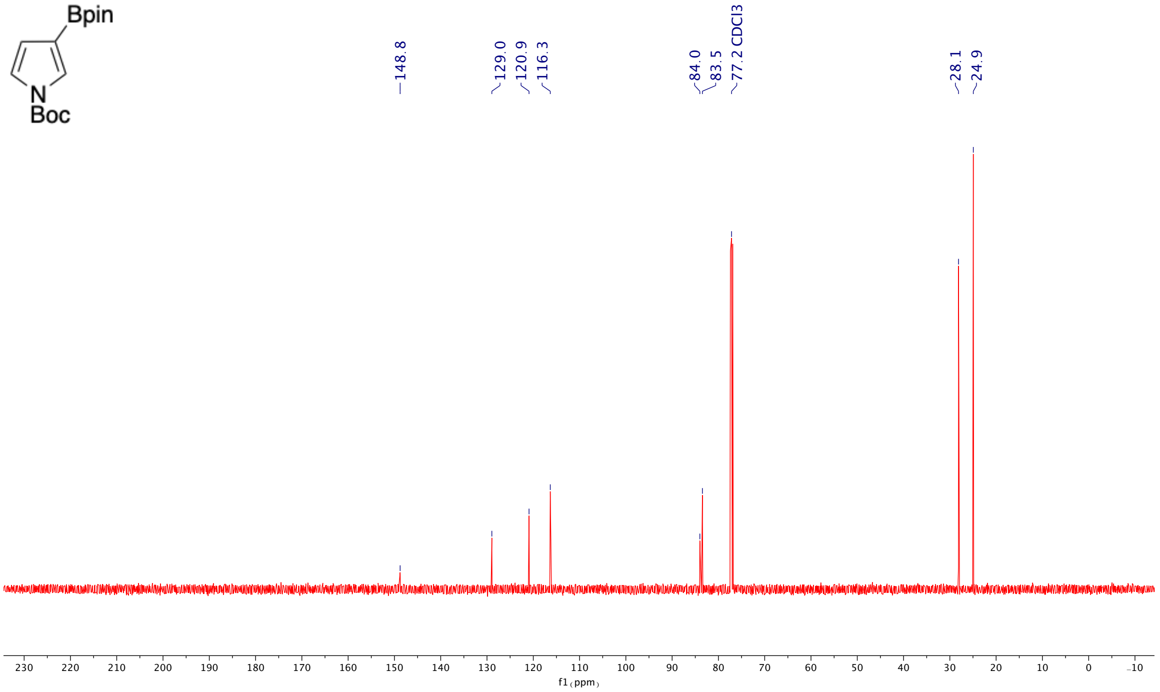

^{11}B NMR (160MHz, $CDCl_3$) of *Tert*-butyl 3-(4,4,5,5-tetramethyl-1,3,2-dioxaborolan-2-yl)-1*H*-pyrrole-1-carboxylate (1g)

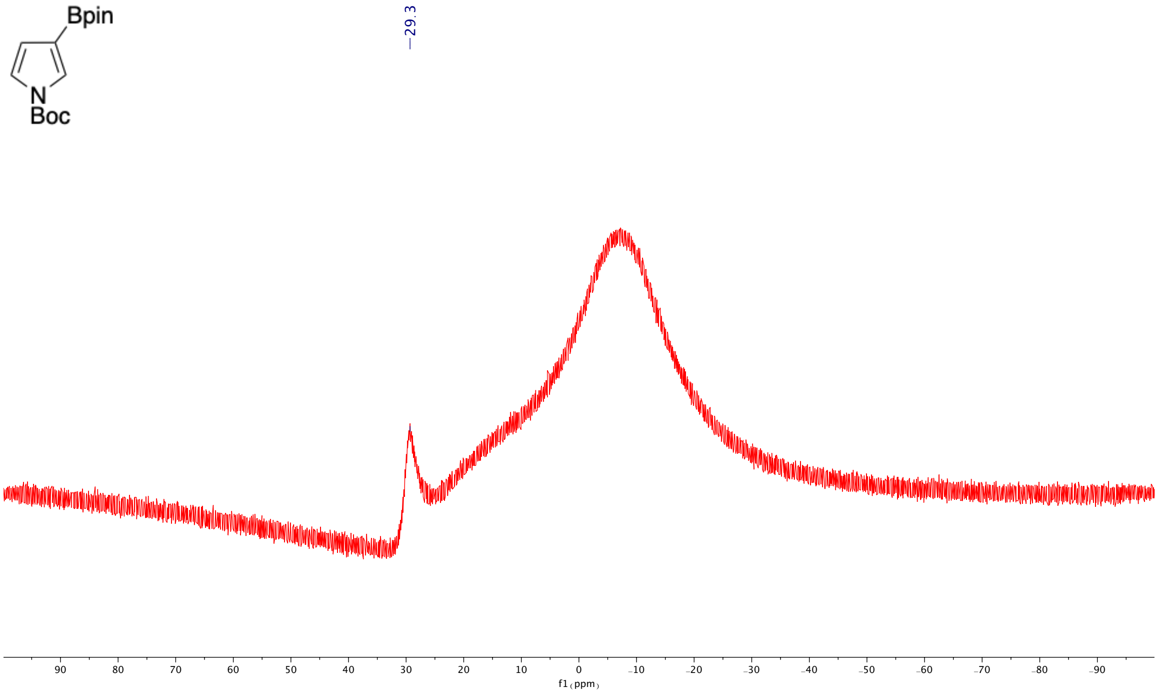

^{1}H NMR (500 MHz, $CDCl_3$) of 3-(benzofuran-2-yl)-1,1,1,3,5,5,5-heptamethyltrisiloxane (1z)

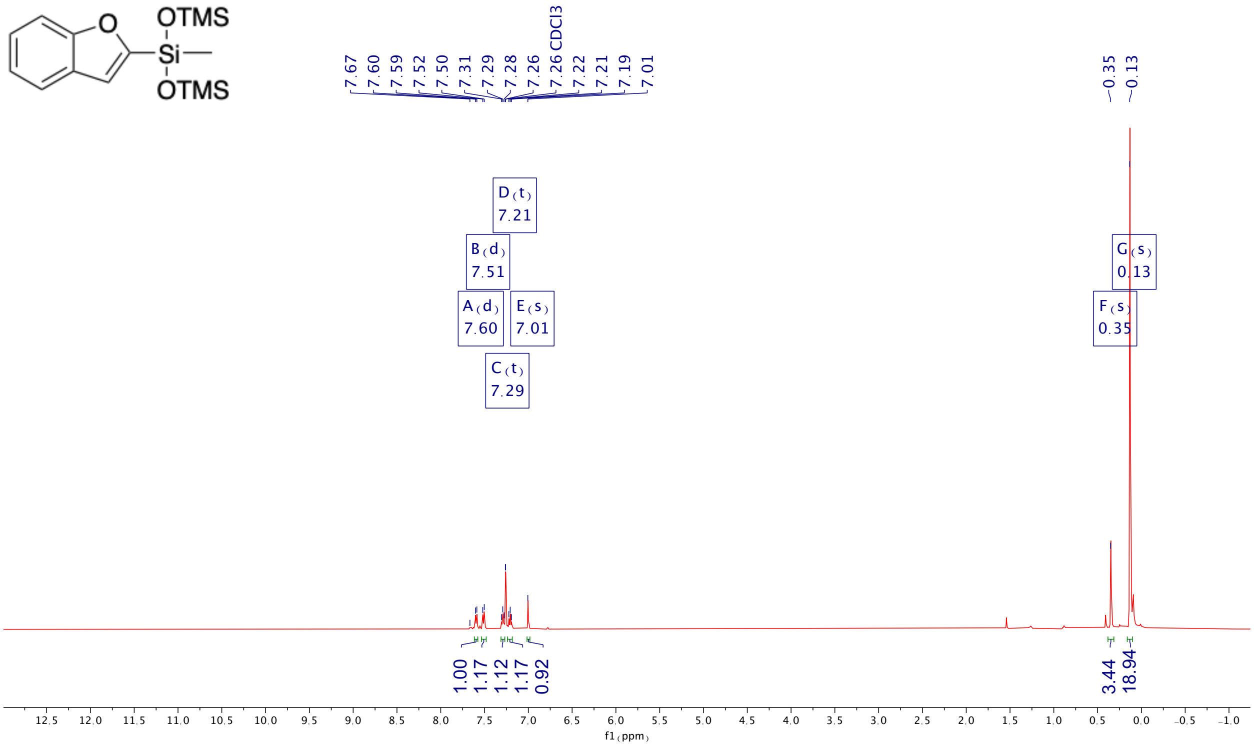

$^{13}C\{^{1}H\}$ (126 MHz, $CDCl_3$) NMR of 3-(benzofuran-2-yl)-1,1,1,3,5,5,5-heptamethyltrisiloxane (1z)

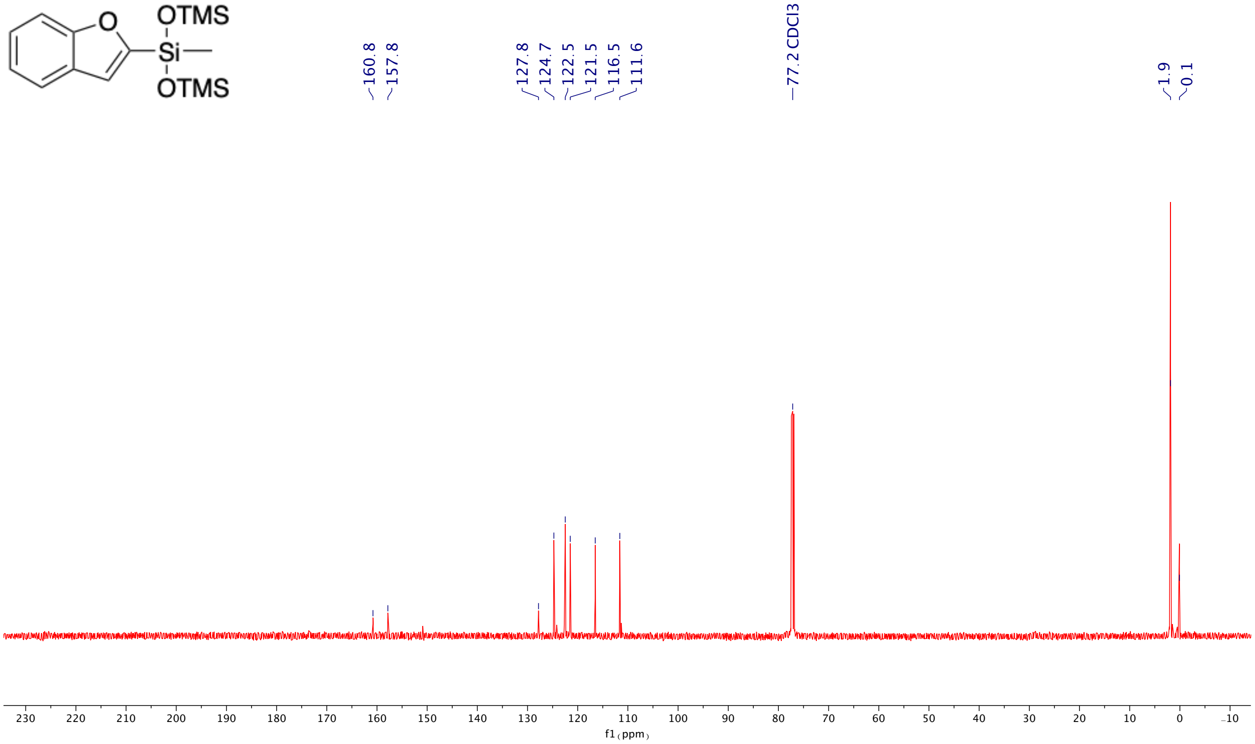

^{29}Si NMR (99MHz, $CDCl_3$) of 3-(benzofuran-2-yl)-1,1,1,3,5,5,5-heptamethyltrisiloxane (1z)

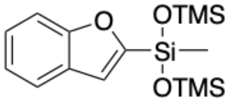

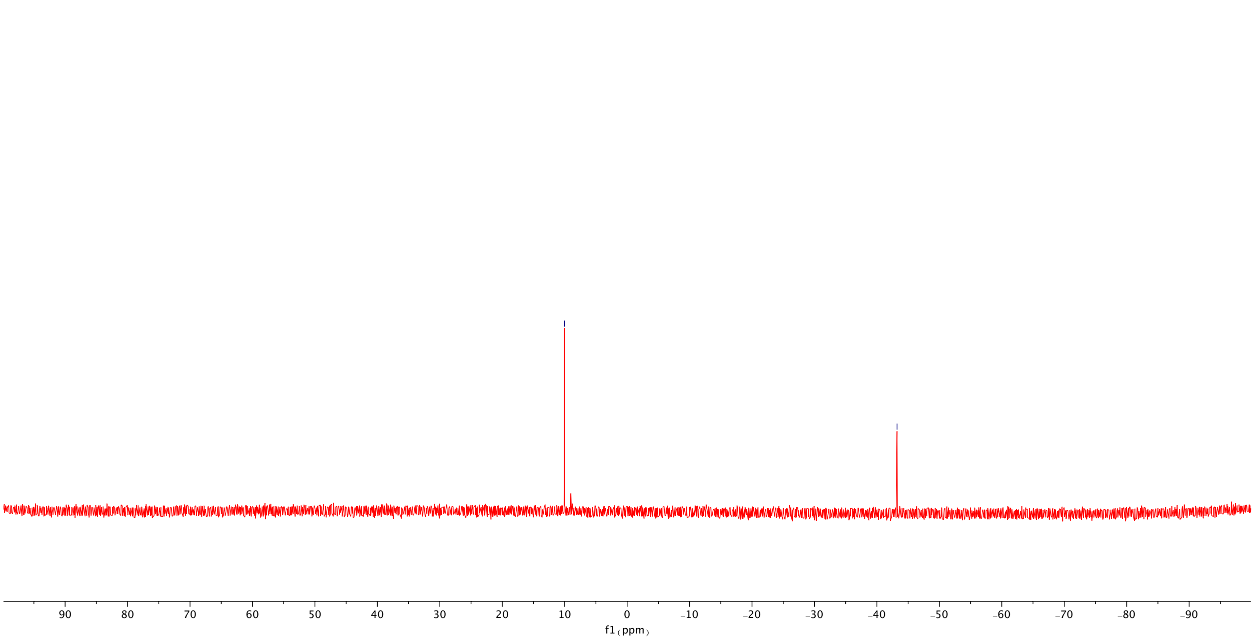

^{1}H NMR (500 MHz, $CDCl_3$) of 2-(benzofuran-7-yl)-4,4,5,5-tetramethyl-1,3,2-dioxaborolane (1j)

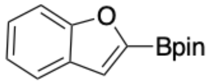

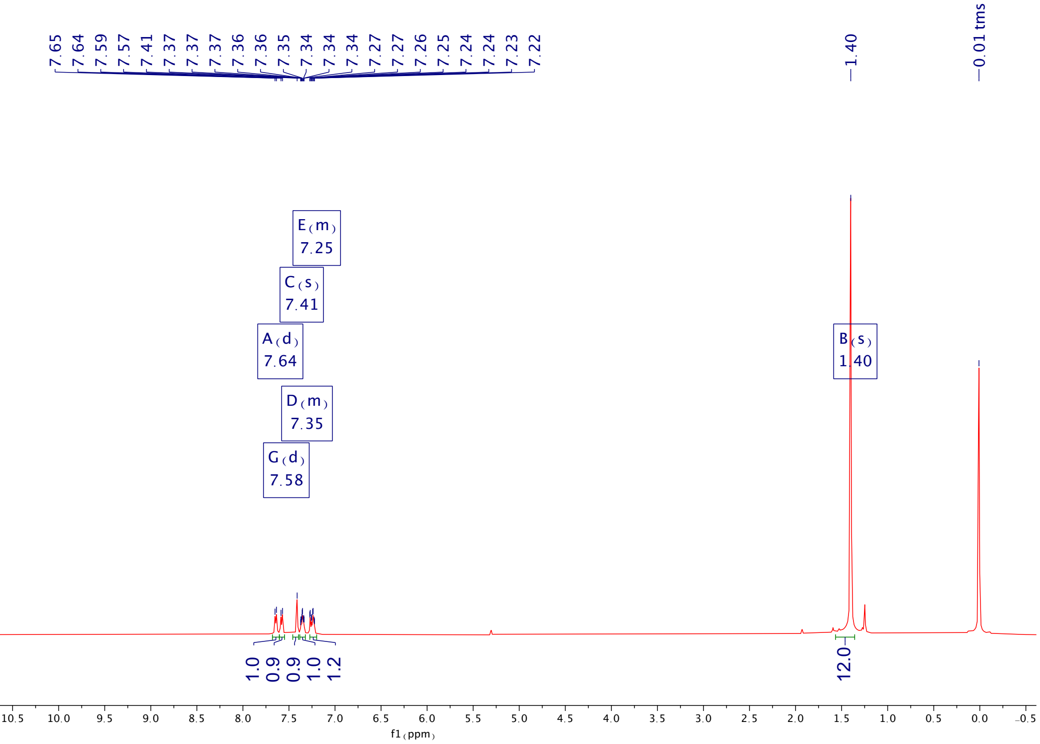

$^{13}C\{^1H\}$ (126 MHz, $CDCl_3$) NMR of 2-(benzofuran-7-yl)-4,4,5,5-tetramethyl-1,3,2-dioxaborolane (1j)

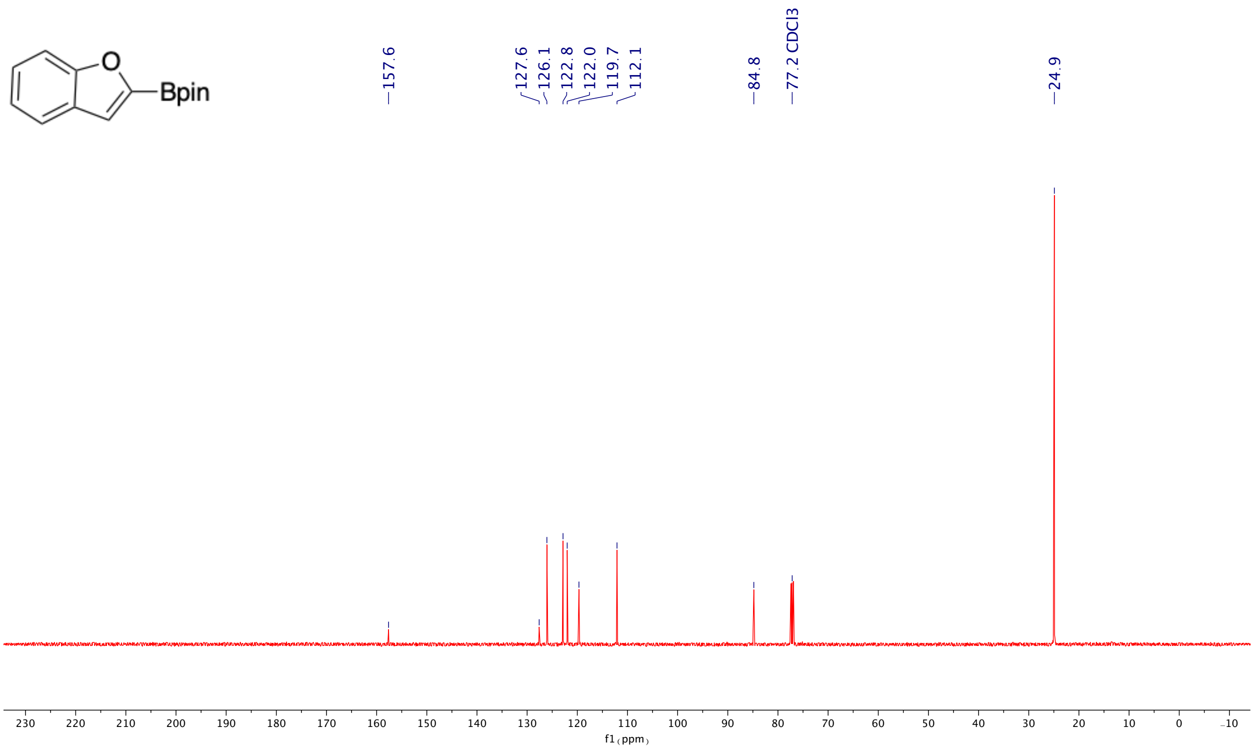

^{11}B NMR (160MHz, $CDCl_3$) of 2-(benzofuran-7-yl)-4,4,5,5-tetramethyl-1,3,2-dioxaborolane (1j)

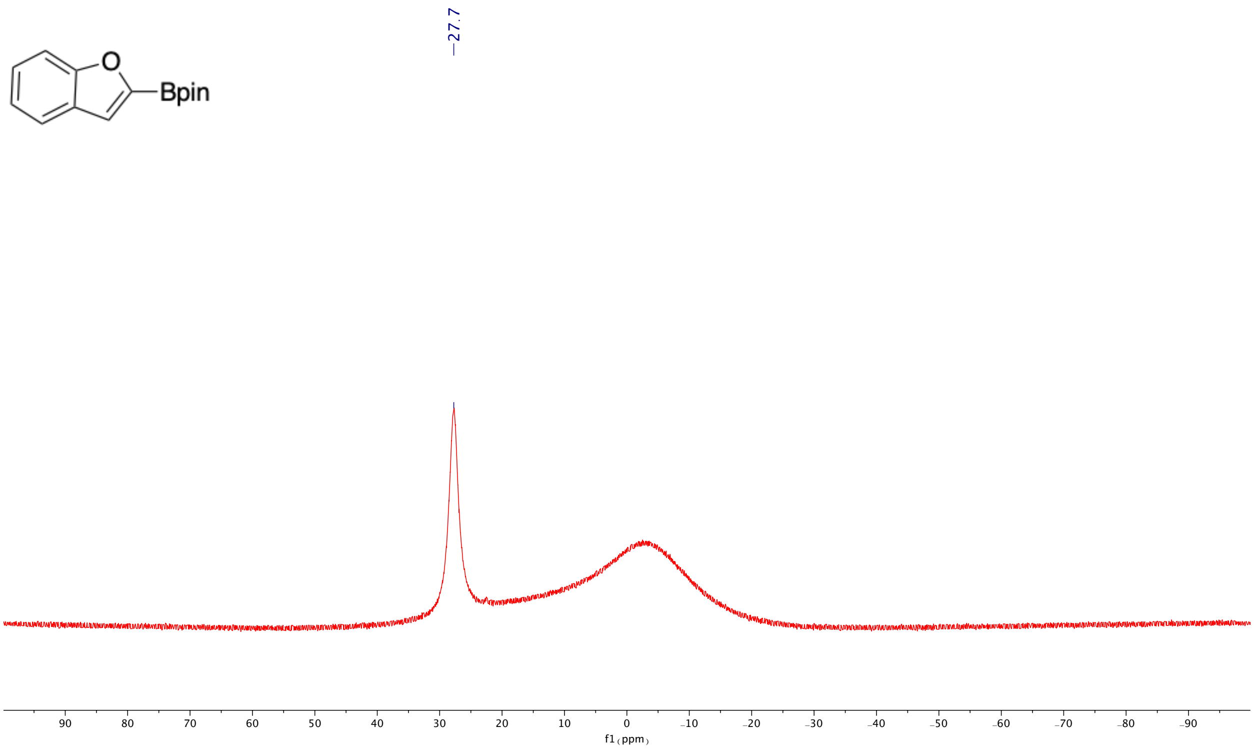

^{1}H NMR (500MHz, $CDCl_3$) of *Tert*-butyl 2-(4,4,5,5-tetramethyl-1,3,2-dioxaborolan-2-yl)-1*H*-indole-1-carboxylate (1l)

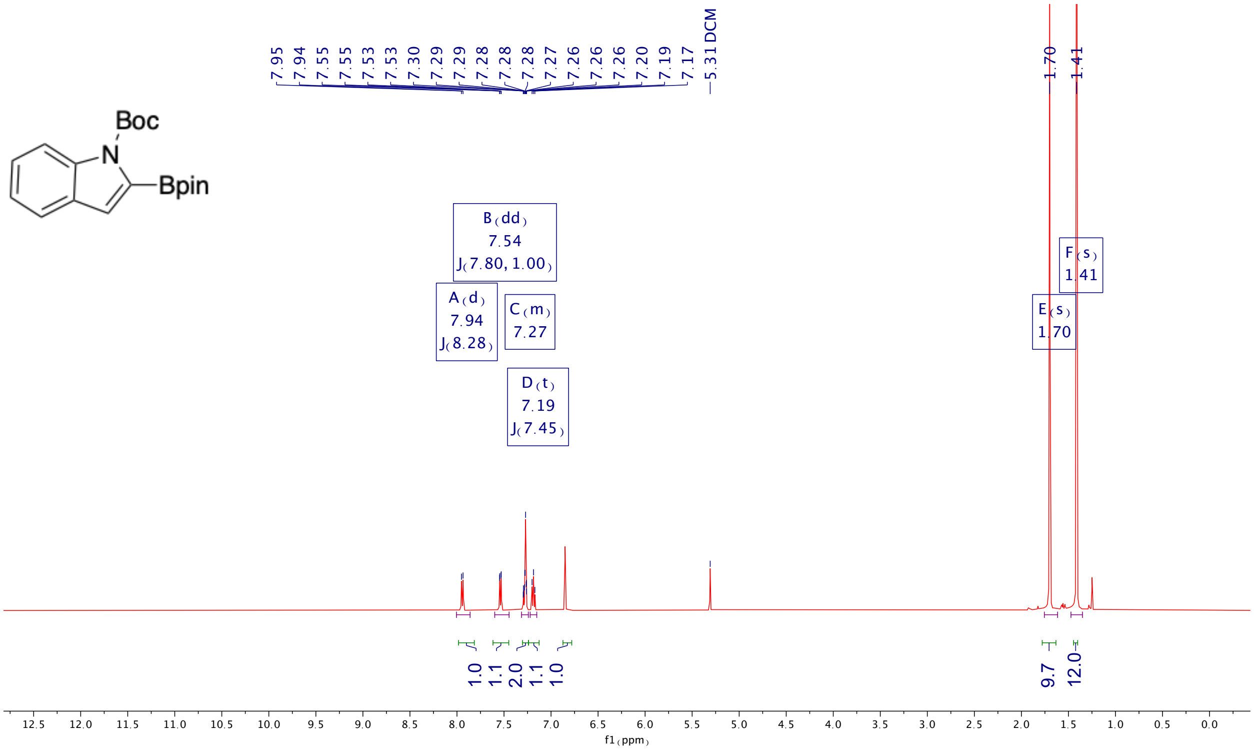

^{13}C{^{1}H} (126 MHz, $CDCl_3$) NMR of *Tert*-butyl 2-(4,4,5,5-tetramethyl-1,3,2-dioxaborolan-2-yl)-1*H*-indole-1-carboxylate (1l)

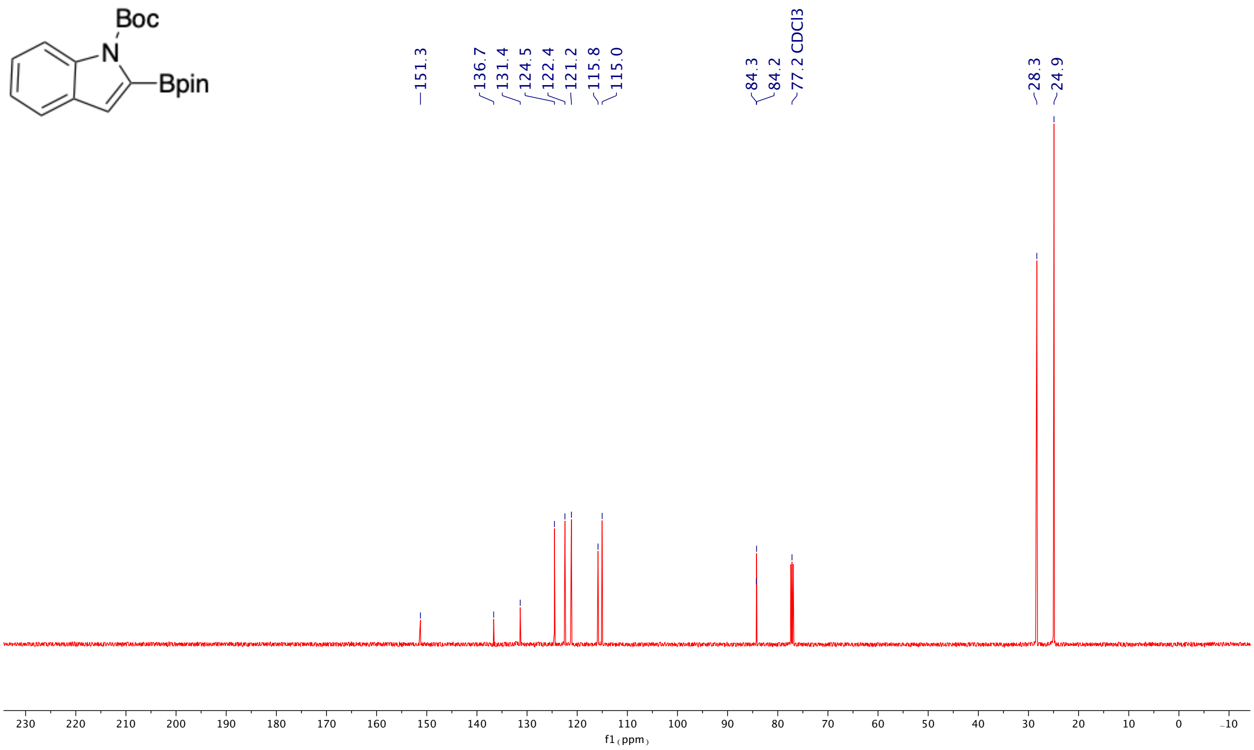

^{11}B NMR (160MHz, CDCl$_3$) of *Tert*-butyl 2-(4,4,5,5-tetramethyl-1,3,2-dioxaborolan-2-yl)-1*H*-indole-1-carboxylate (1l)

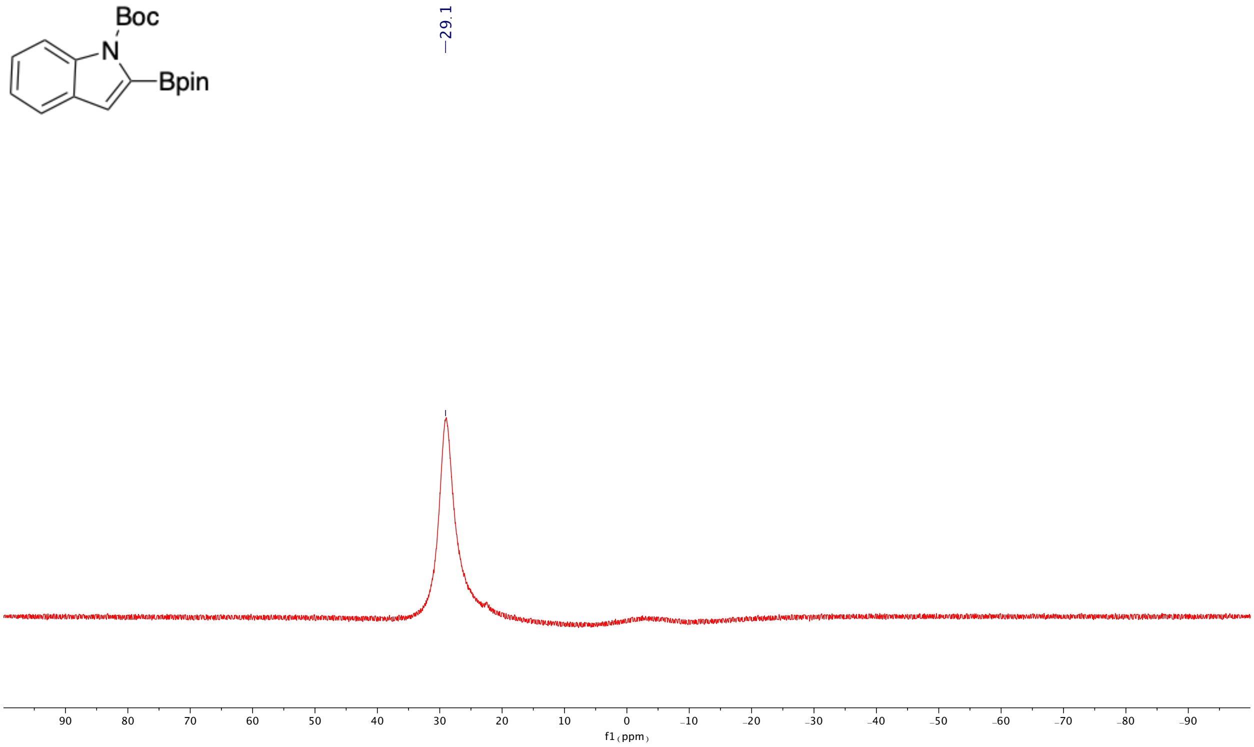

^{1}H NMR (500MHz, $CDCl_3$) of 3-(4,4,5,5-Tetramethyl-1,3,2-dioxaborolan-2-yl)phenol (1q)

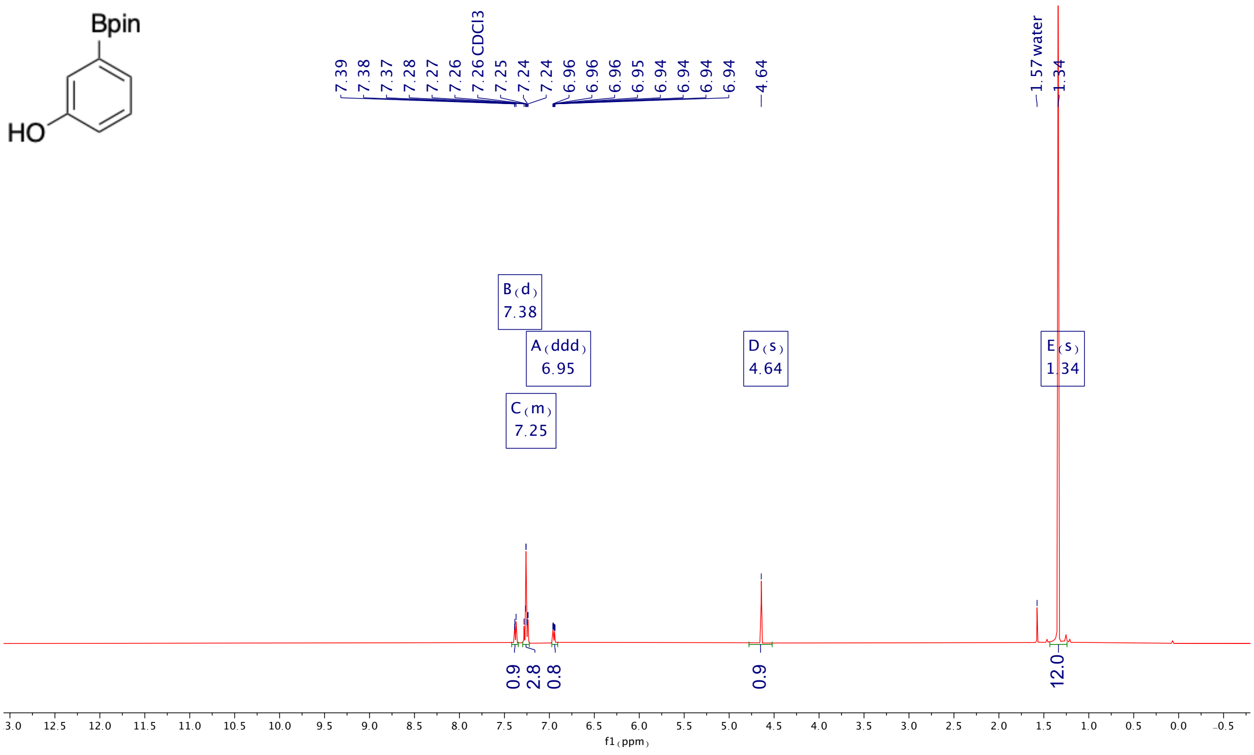

$^{13}C\{^{1}H\}$ (126 MHz, $CDCl_3$) NMR of 3-(4,4,5,5-Tetramethyl-1,3,2-dioxaborolan-2-yl)phenol (1q)

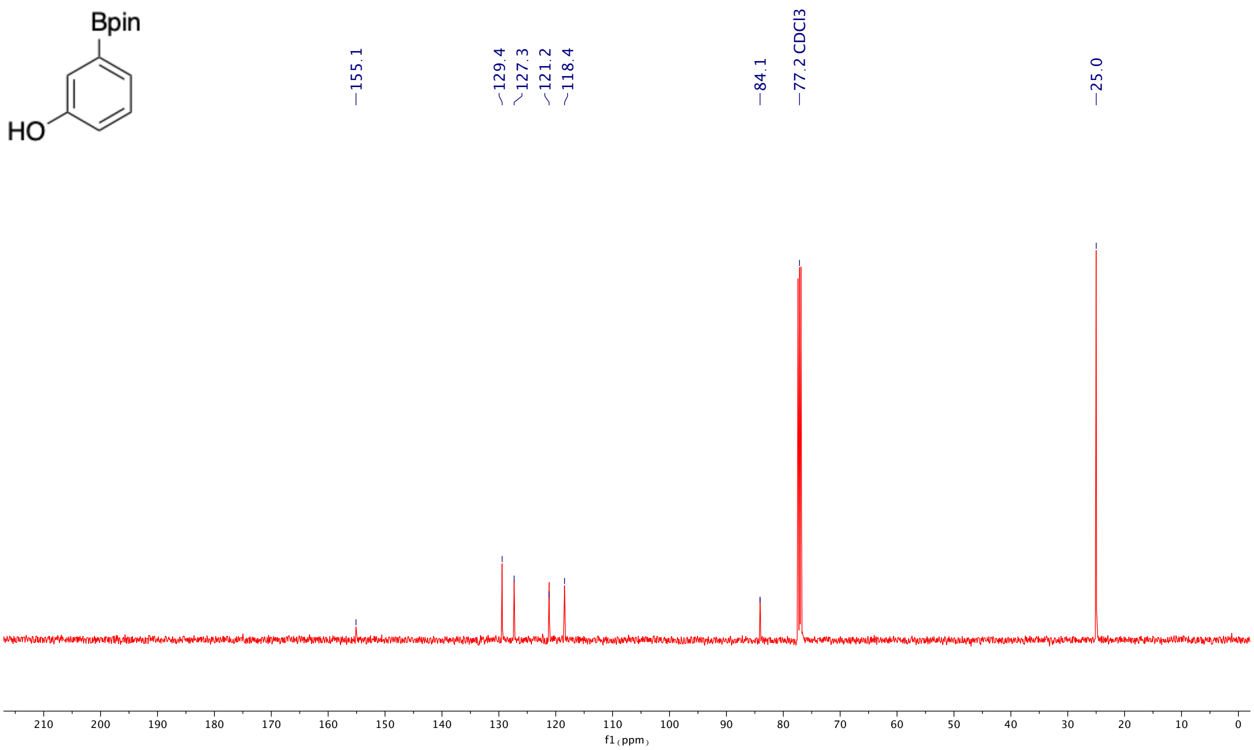

^{11}B NMR (160 MHz, $CDCl_3$) of 3-(4,4,5,5-Tetramethyl-1,3,2-dioxaborolan-2-yl)phenol (1q)

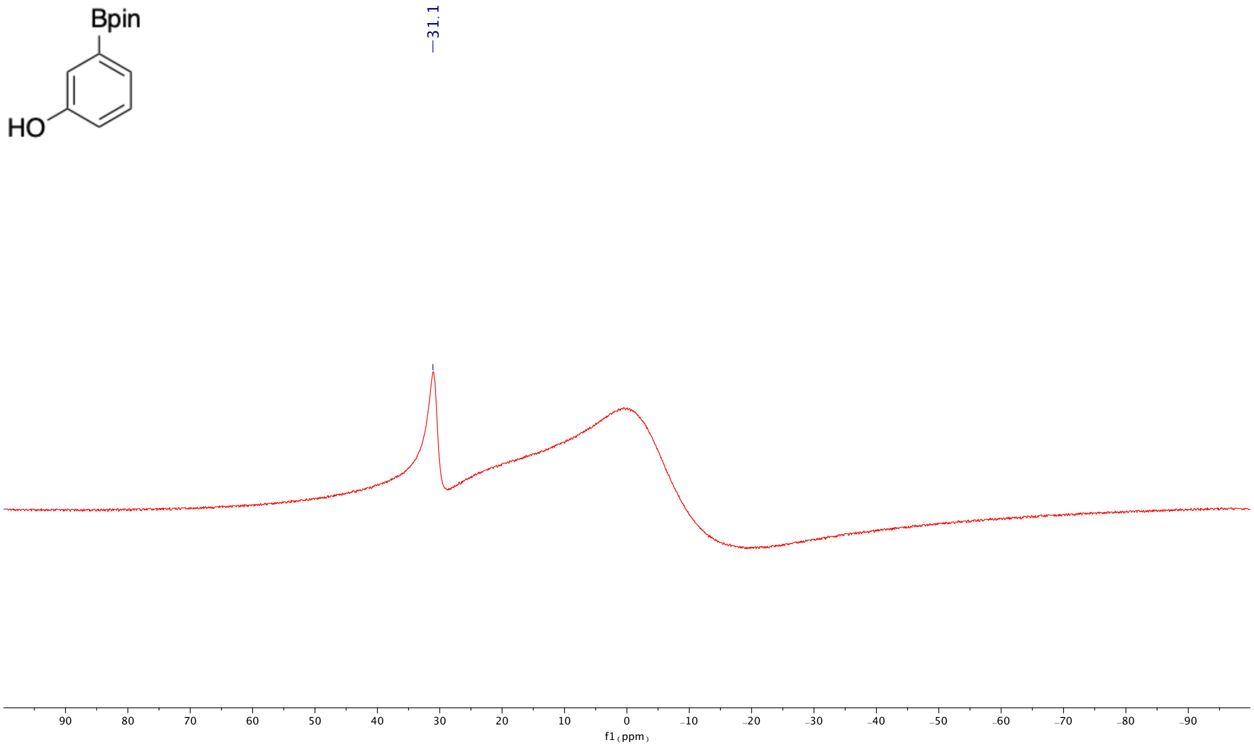

^{1}H NMR (500 MHz, $CDCl_3$) of trimethyl(3-(4,4,5,5-tetramethyl-1,3,2-dioxaborolan-2-yl)phenyl)silane (1ae)

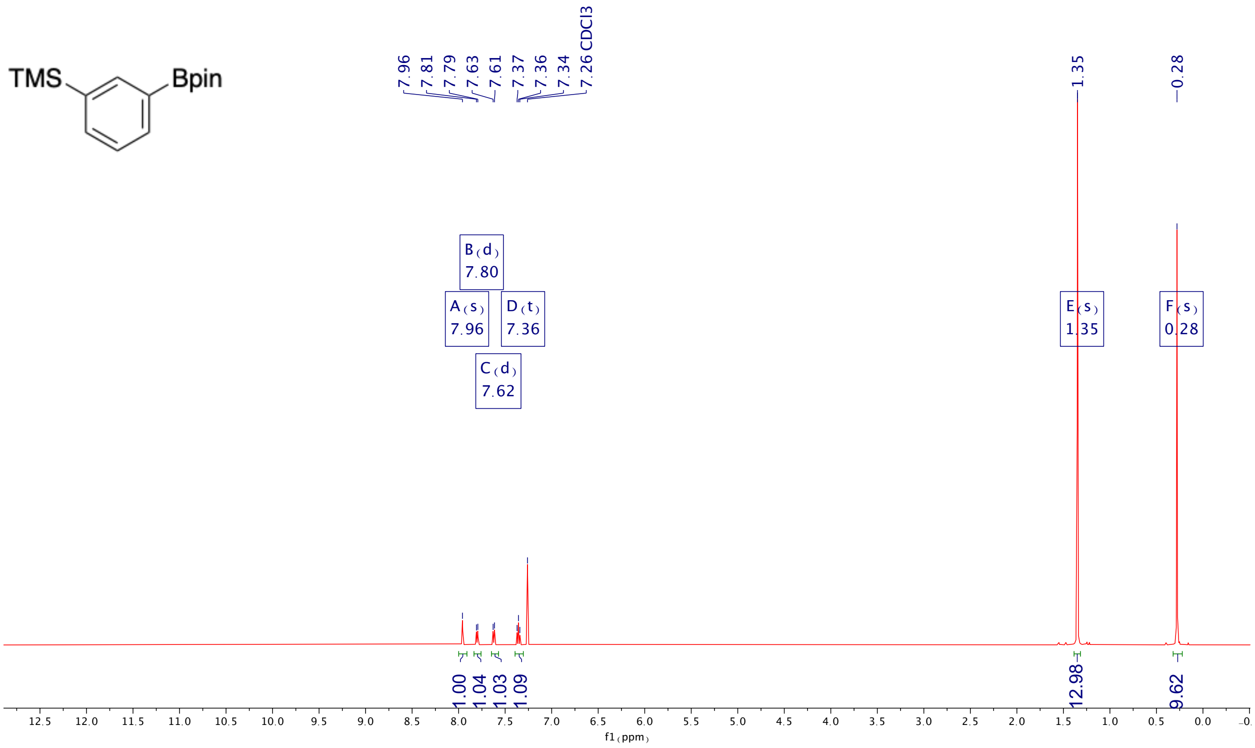

$^{13}C\{^1H\}$ (126 MHz, $CDCl_3$) NMR of trimethyl(3-(4,4,5,5-tetramethyl-1,3,2-dioxaborolan-2-yl)phenyl)silane (1ae)

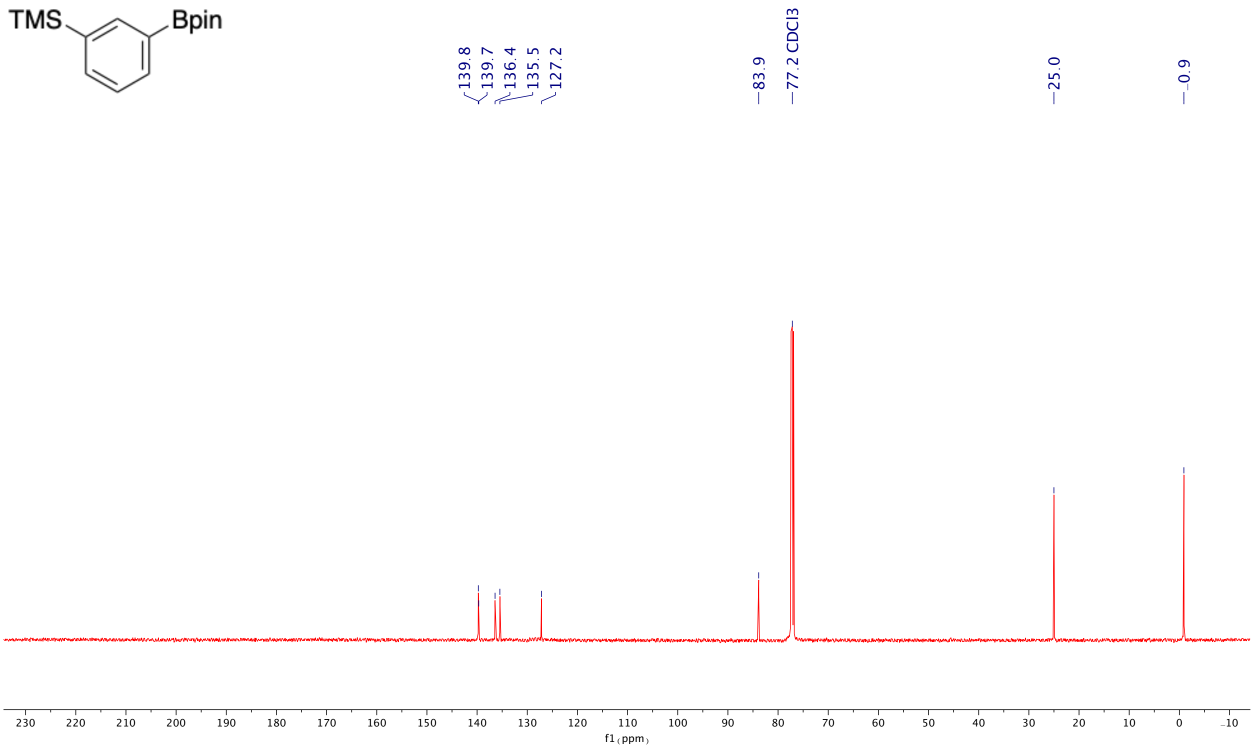

^{11}B NMR (160 MHz, $CDCl_3$) of trimethyl(3-(4,4,5,5-tetramethyl-1,3,2-dioxaborolan-2-yl)phenyl)silane (1ae)

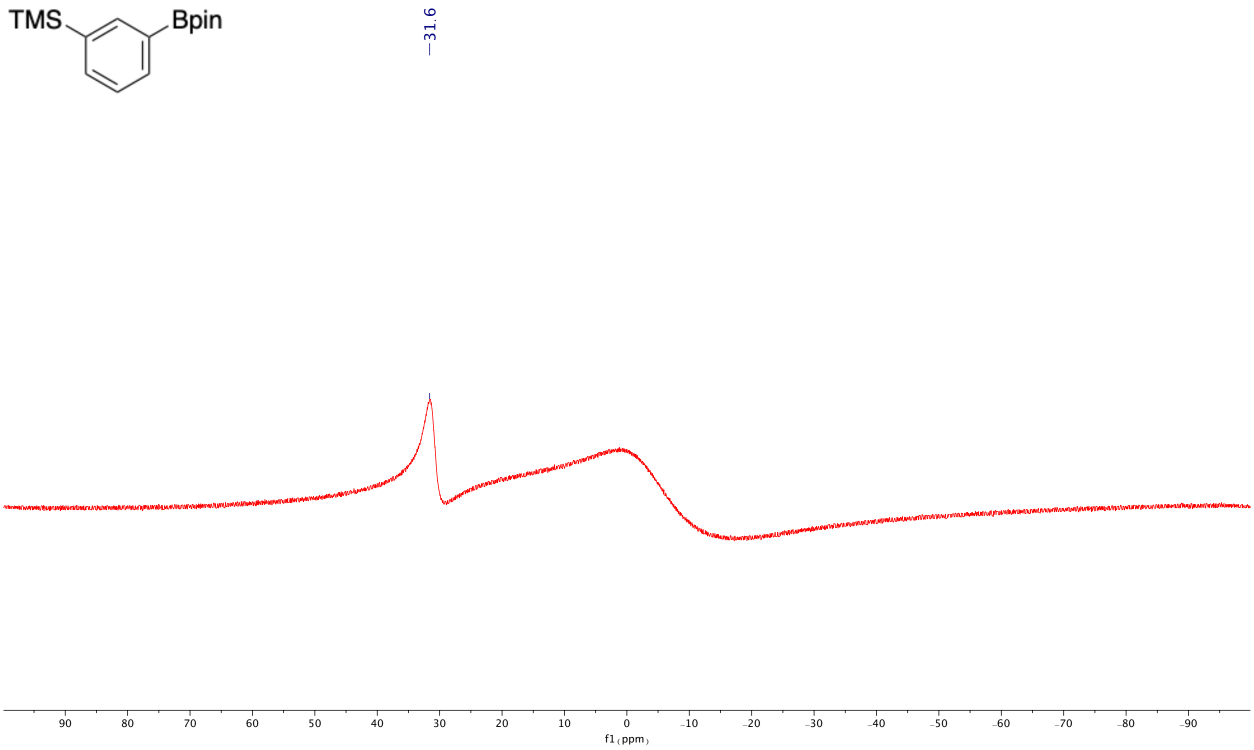

^{29}Si NMR (99 MHz, $CDCl_3$) of trimethyl(3-(4,4,5,5-tetramethyl-1,3,2-dioxaborolan-2-yl)phenyl)silane (1ae)

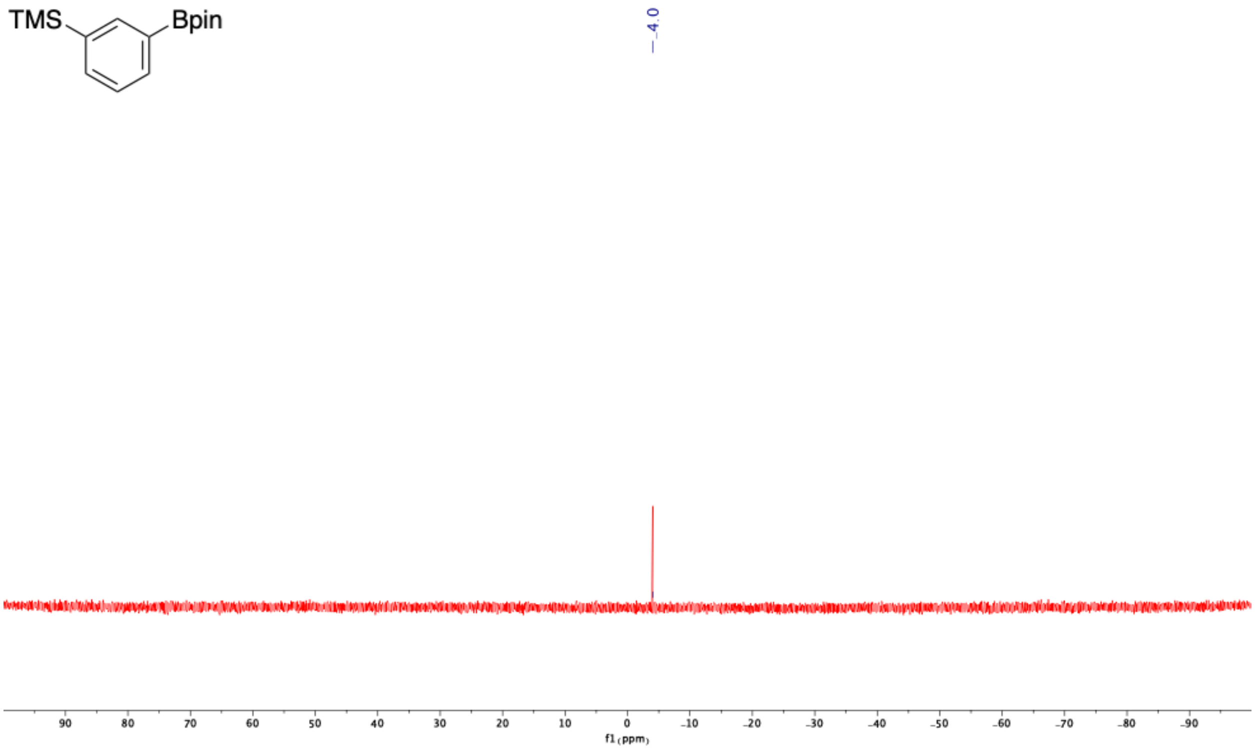

^{1}H NMR (500 MHz, $CDCl_3$) of trimethyl(4-(4,4,5,5-tetramethyl-1,3,2-dioxaborolan-2-yl)phenyl)silane (1af)

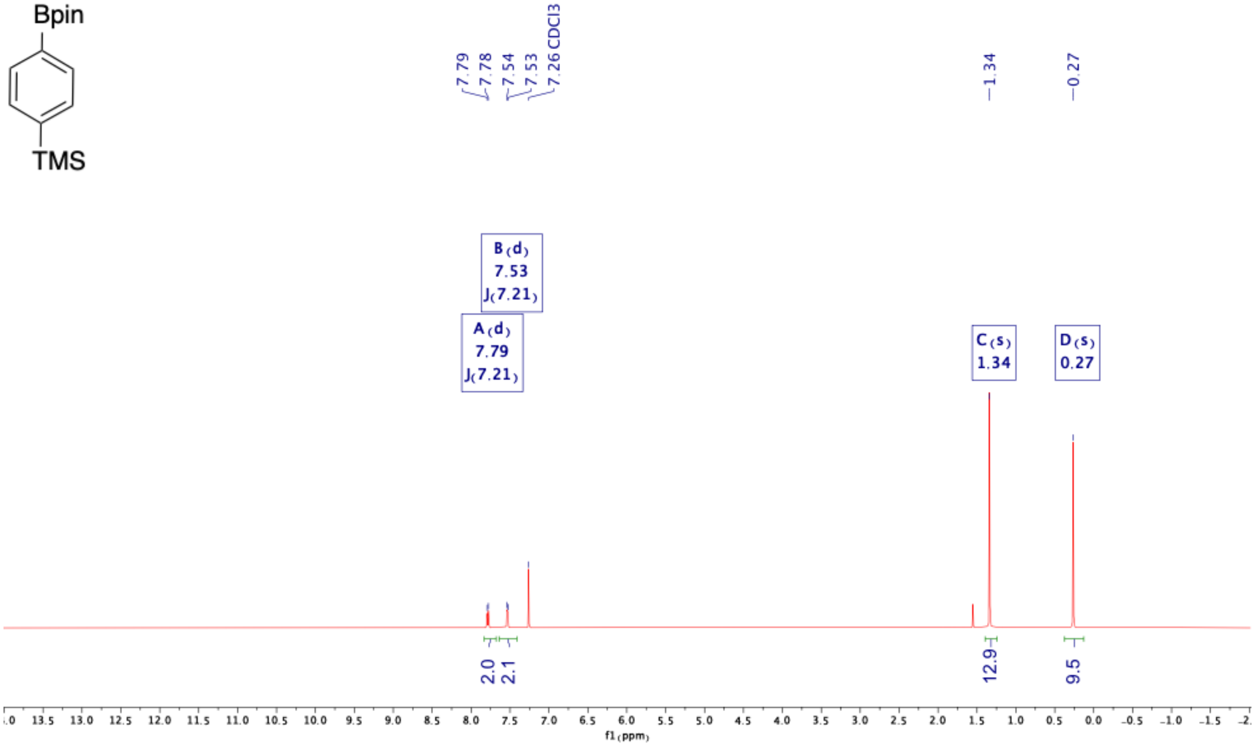

$^{13}C\{^{1}H\}$ (126 MHz, CDCl$_3$) NMR of trimethyl(4-(4,4,5,5-tetramethyl-1,3,2-dioxaborolan-2-yl)phenyl)silane (1af)

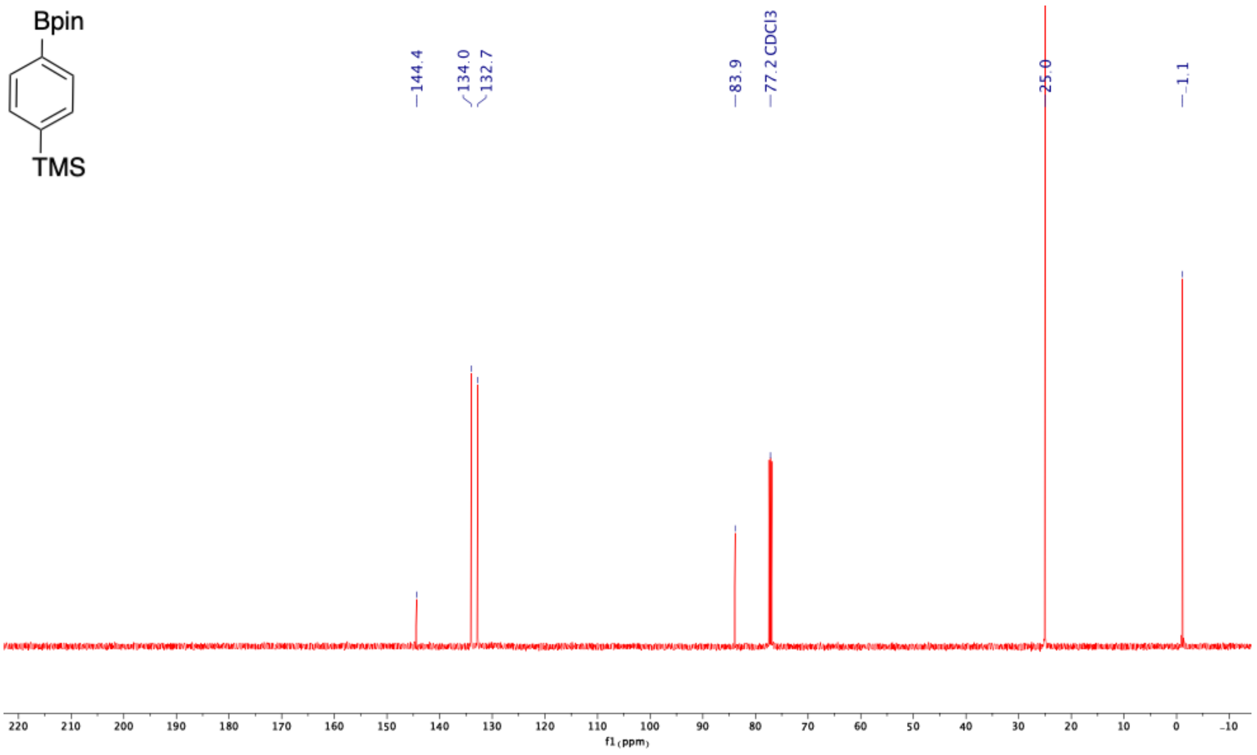

^{11}B NMR (160 MHz, CDCl$_3$) of trimethyl(4-(4,4,5,5-tetramethyl-1,3,2-dioxaborolan-2-yl)phenyl)silane (1af)

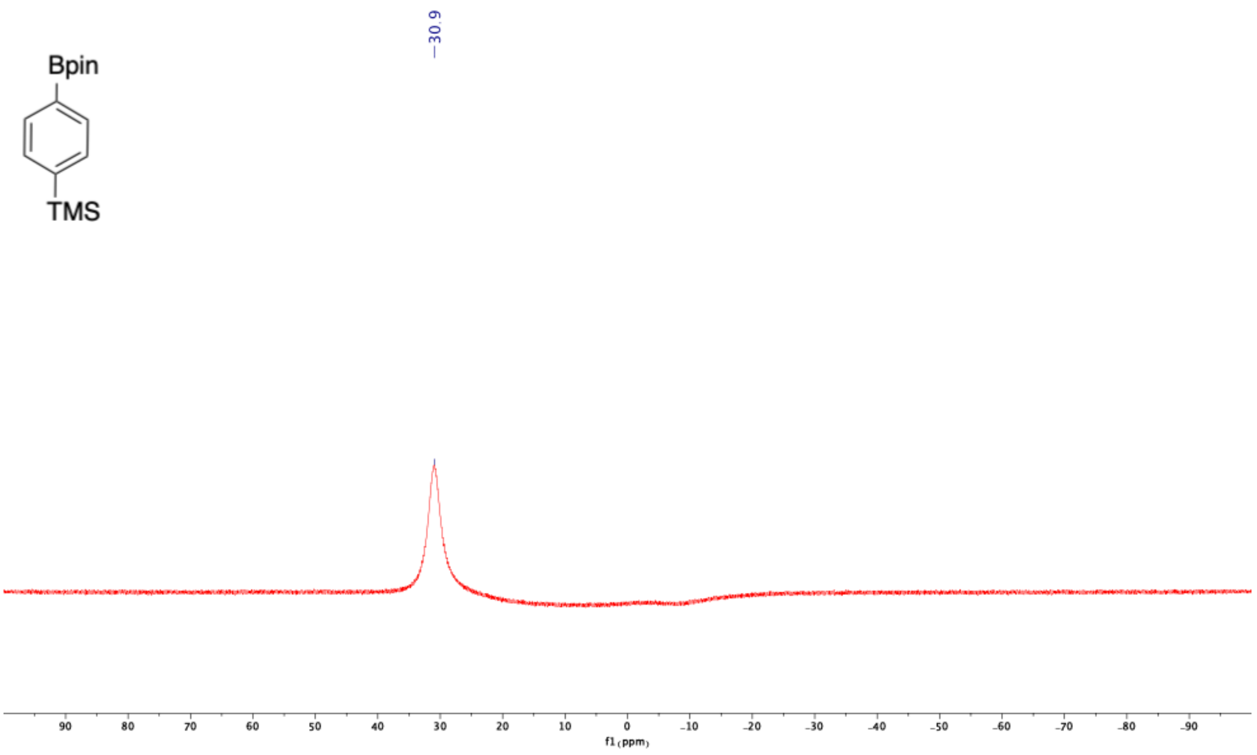

^{29}Si NMR (99 MHz, CDCl$_3$) of trimethyl(4-(4,4,5,5-tetramethyl-1,3,2-dioxaborolan-2-yl)phenyl)silane (1af)

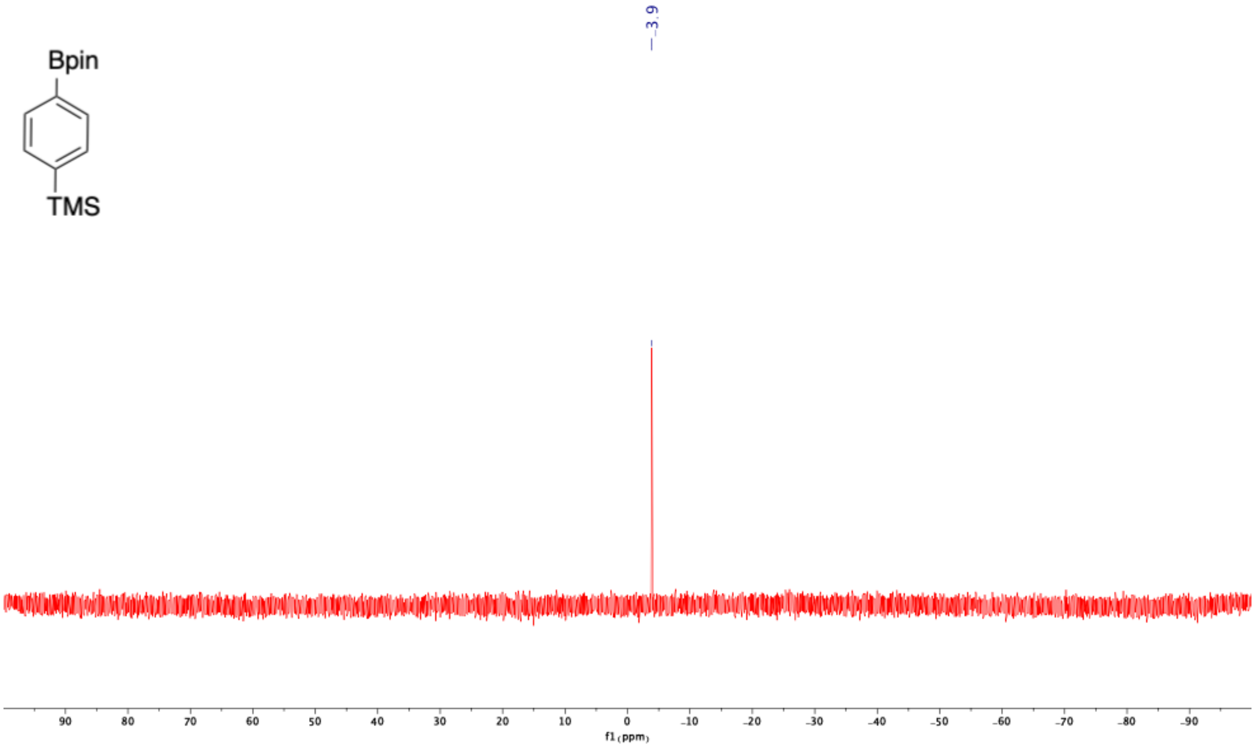

^{1}H NMR (500MHz, CDCl$_3$) of 2-(dibenzo[*b*,*d*]furan-4-yl)-4,4,5,5-tetramethyl-1,3,2-dioxaborolane (1k)

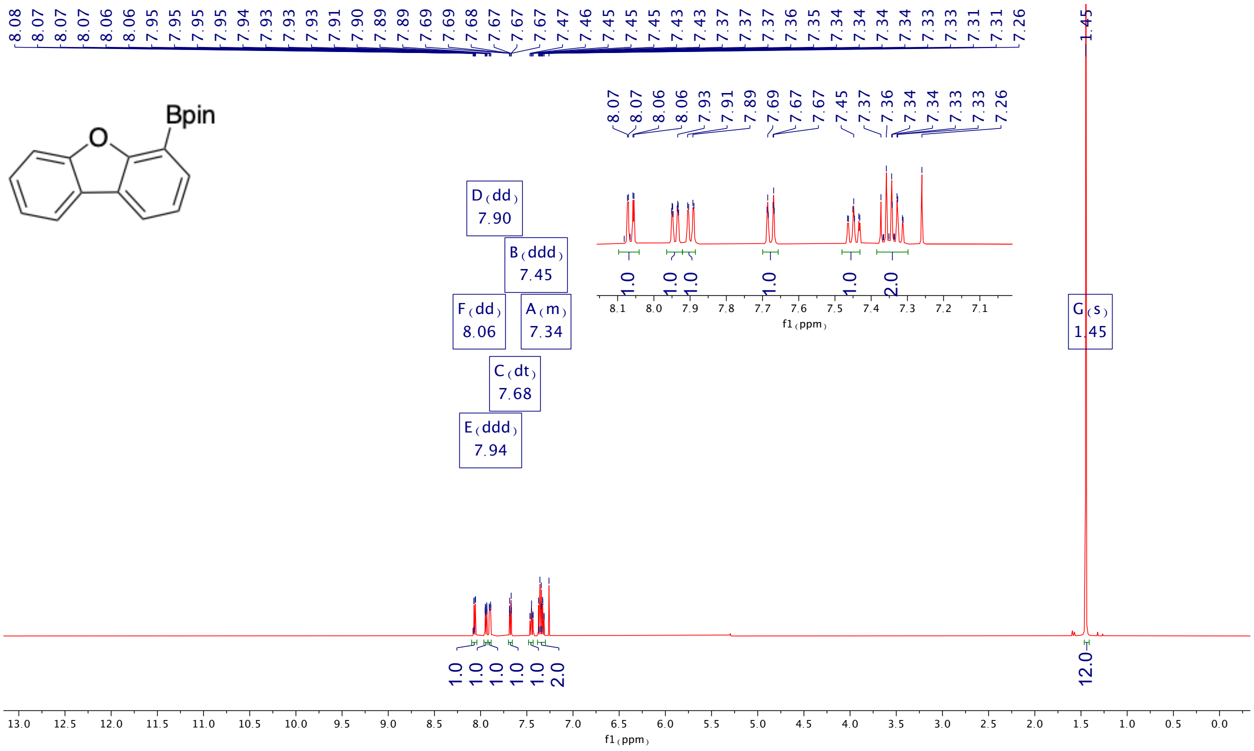

$^{13}C\{^{1}H\}$ (126 MHz, $CDCl_3$) NMR of 2-(dibenzo[*b*,*d*]furan-4-yl)-4,4,5,5-tetramethyl-1,3,2-dioxaborolane (1k)

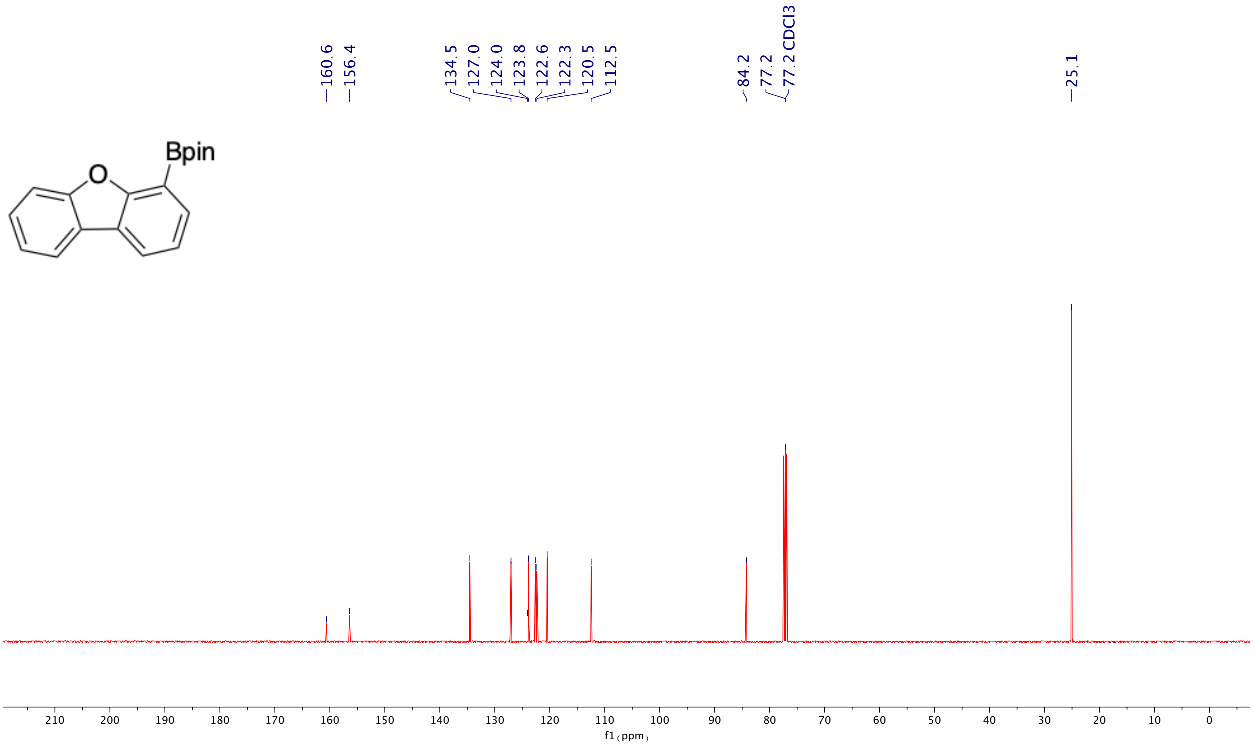

^{11}B NMR (160MHz, $CDCl_3$) of 2-(dibenzo[*b*,*d*]furan-4-yl)-4,4,5,5-tetramethyl-1,3,2-dioxaborolane (1k)

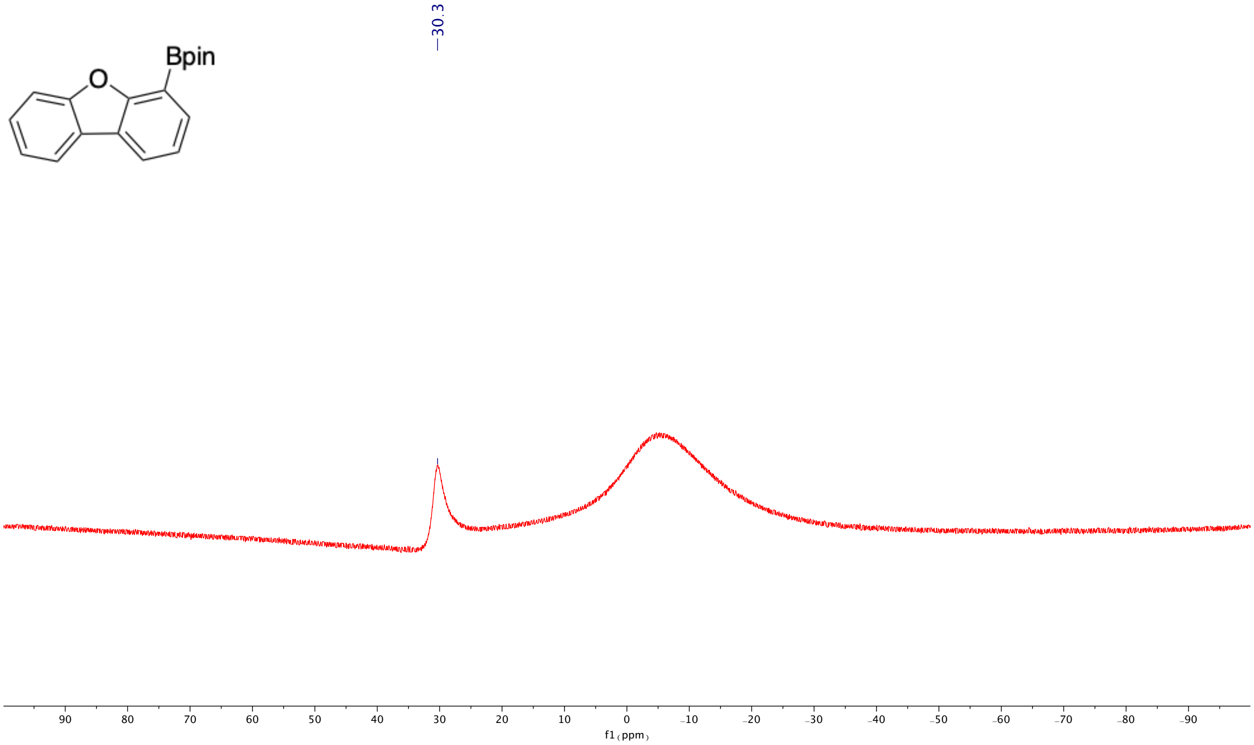

^{1}H NMR (500MHz, $CDCl_3$) 2-Chloro-6-(trifluoromethyl)-3-(trimethylsilyl)pyridine (1y)

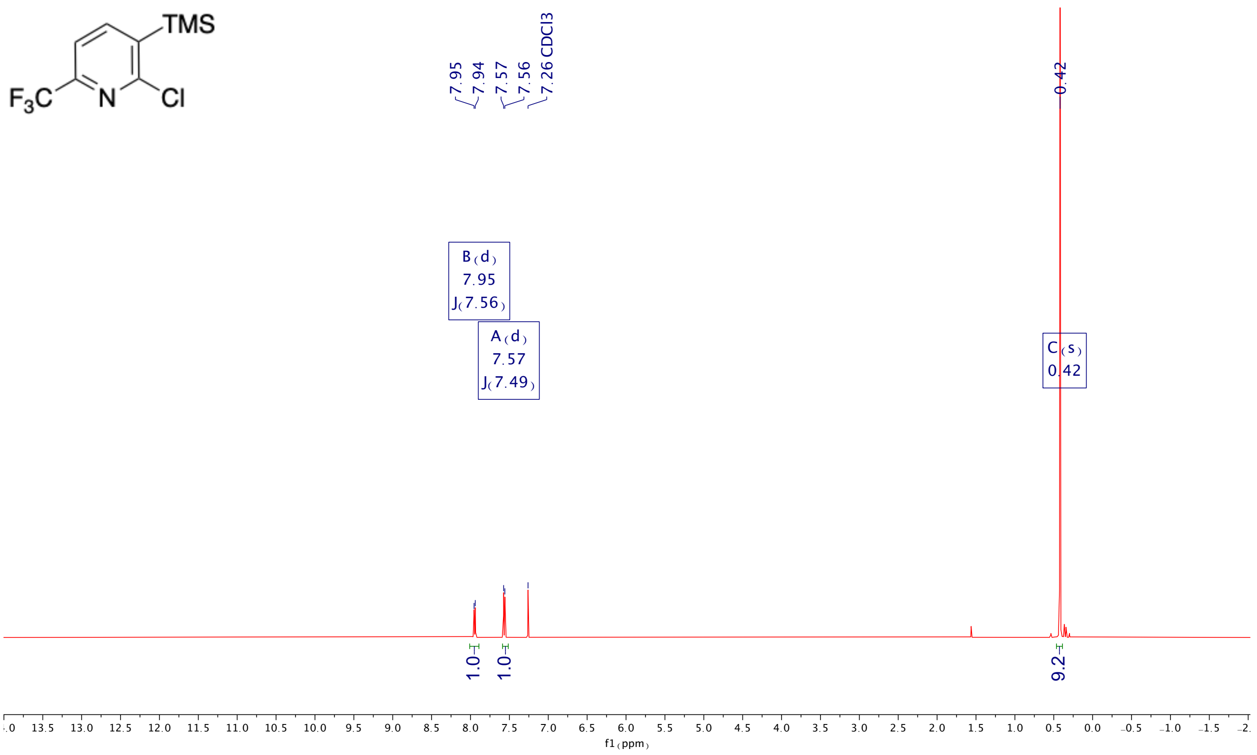

$^{13}C\{^{1}H\}$ (126 MHz, $CDCl_3$) NMR of 2-Chloro-6-(trifluoromethyl)-3-(trimethylsilyl)pyridine (1y)

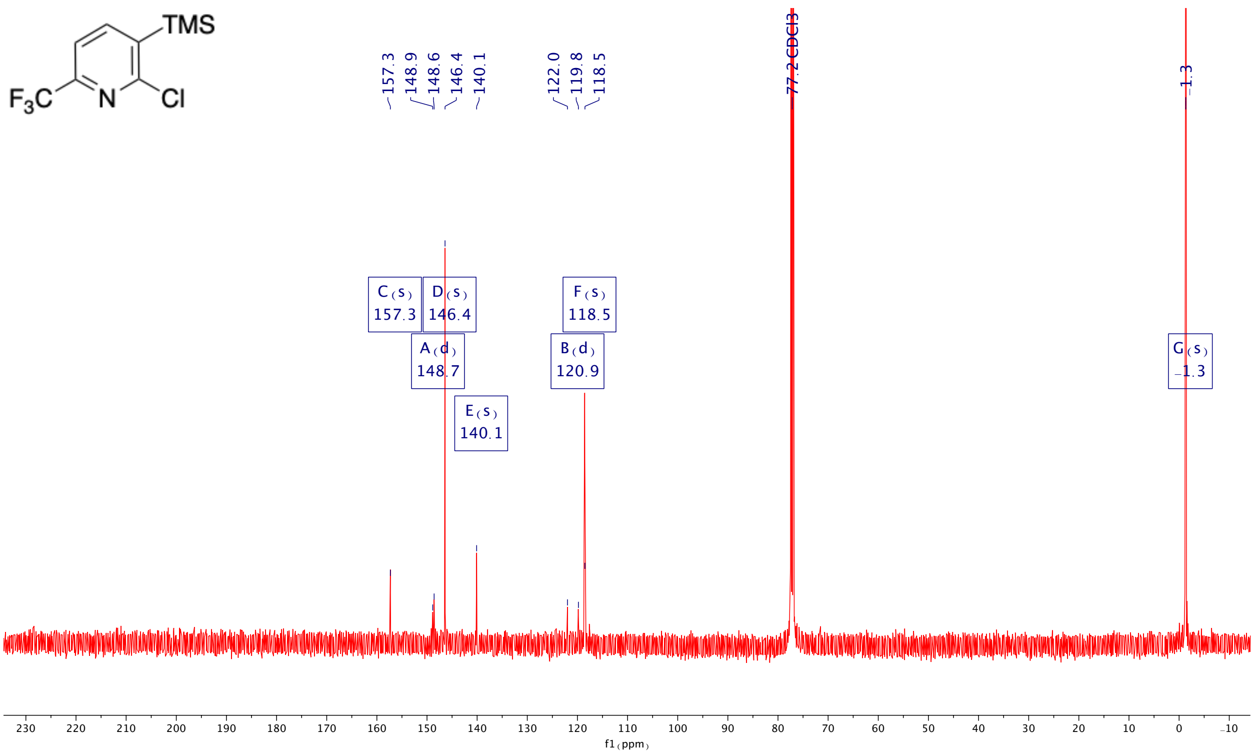

^{19}F NMR (470 MHz, $CDCl_3$) of 2-Chloro-6-(trifluoromethyl)-3-(trimethylsilyl)pyridine (1y)

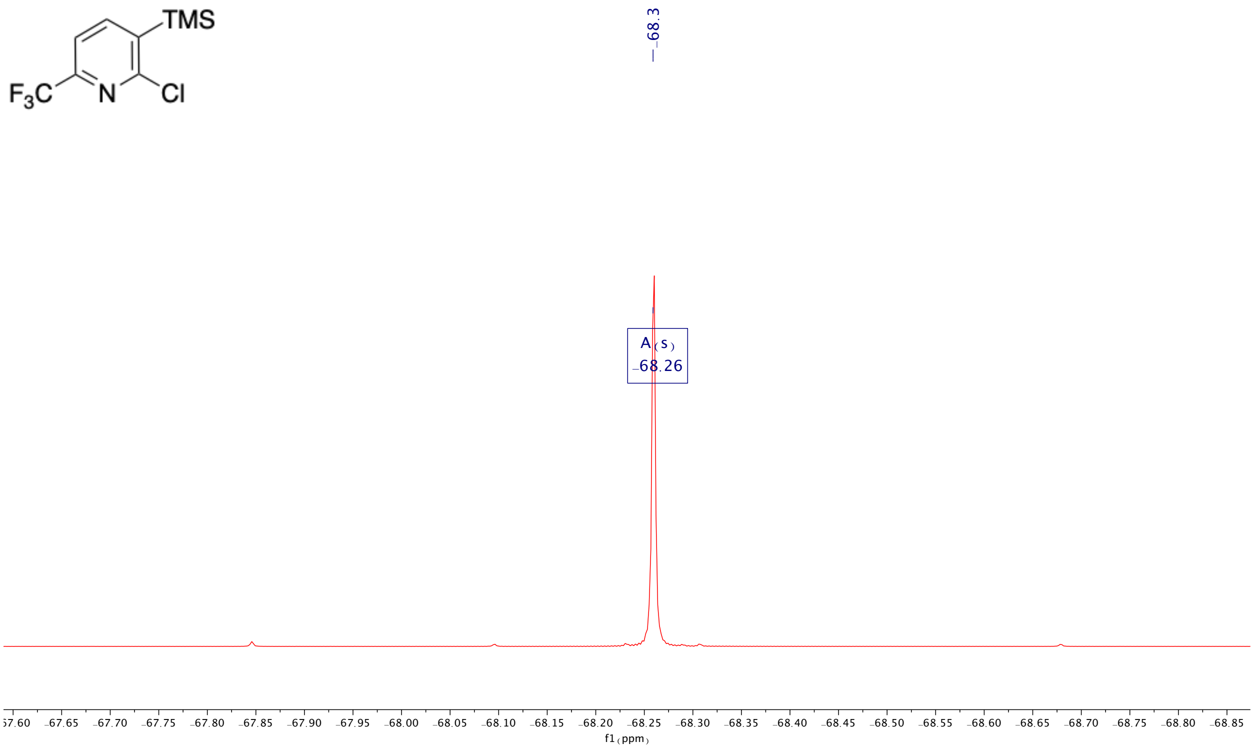

^{29}Si NMR (99MHz, $CDCl_3$) of 2-Chloro-6-(trifluoromethyl)-3-(trimethylsilyl)pyridine (1y)

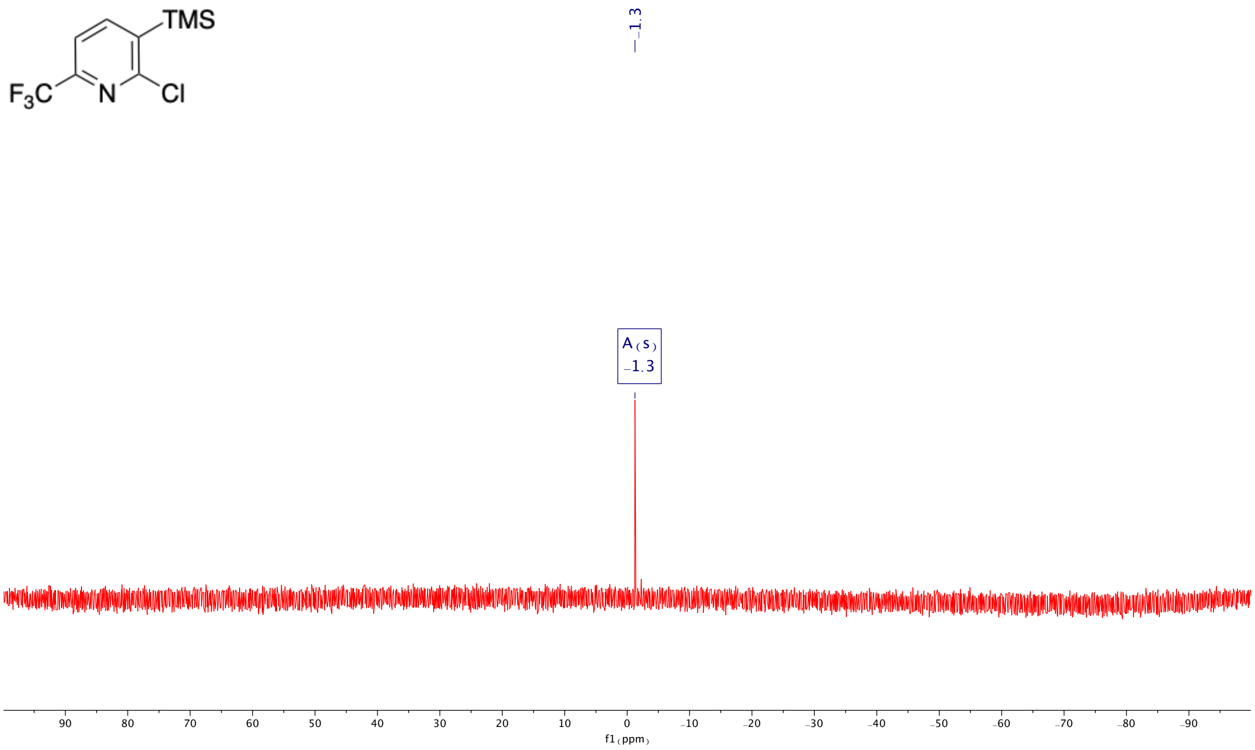

^{1}H NMR (500MHz, $CDCl_3$) 2-chloro-6-(trifluoromethyl)-4-(trimethylsilyl)pyridine (1x)

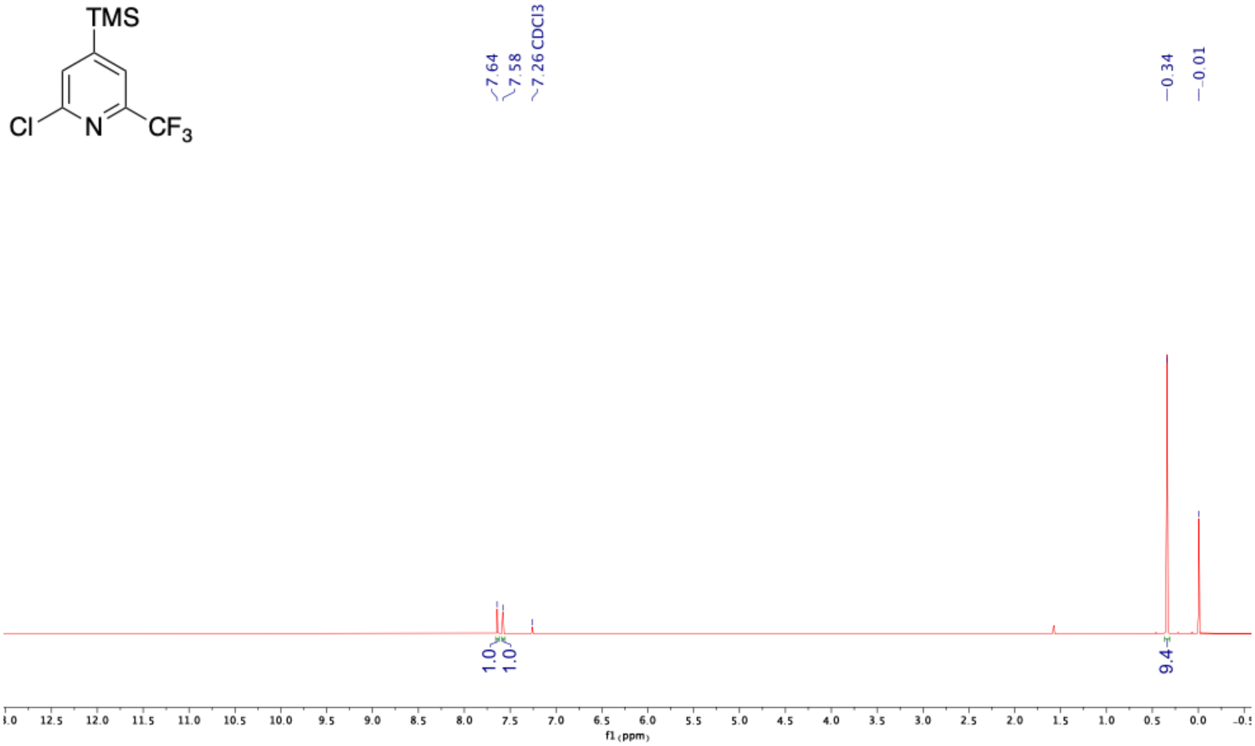

^{13}C{^{1}H} (126 MHz, $CDCl_3$) NMR of 2-chloro-6-(trifluoromethyl)-4-(trimethylsilyl)pyridine (1x)

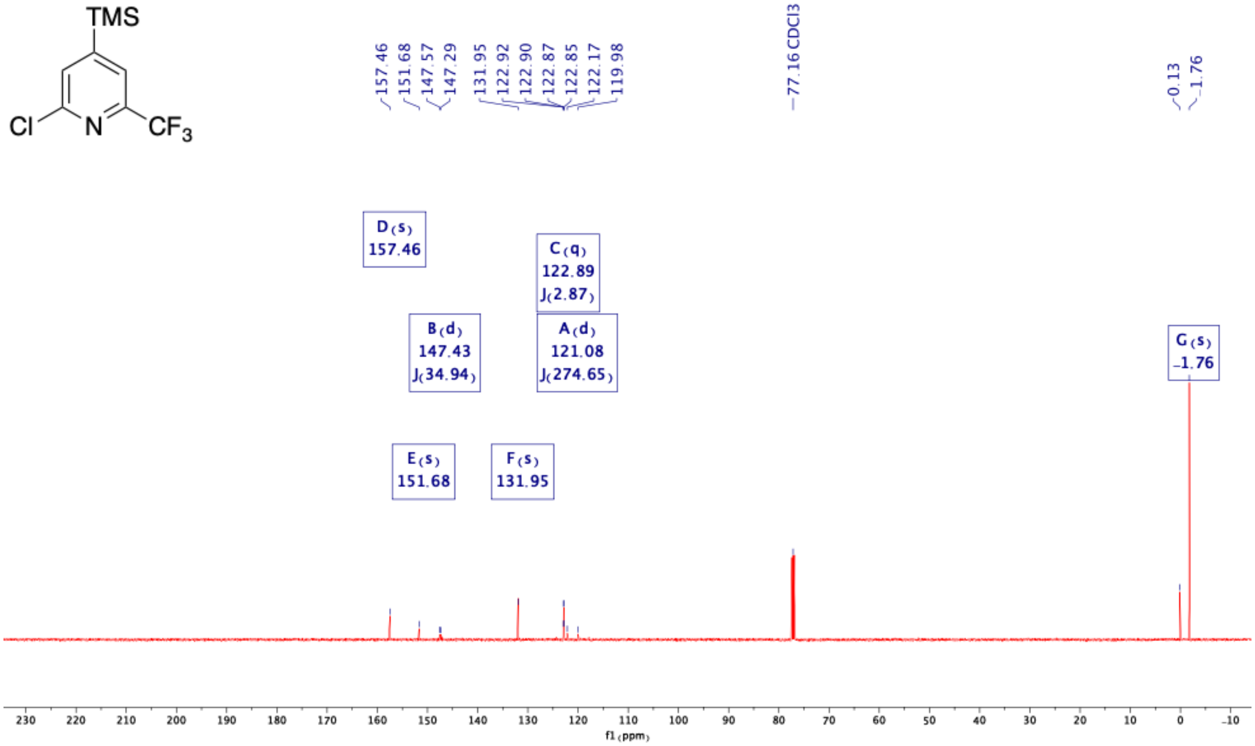

^{1}H NMR (500MHz, $CDCl_3$) *t*-butyl-4-(4,4,5,5-tetramethyl-1,3,2-dioxaborolan-2-yl)-2-(trimethylsilyl)pyrrole-1-carboxylate (1ab)

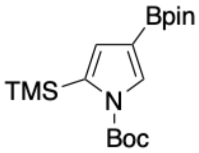

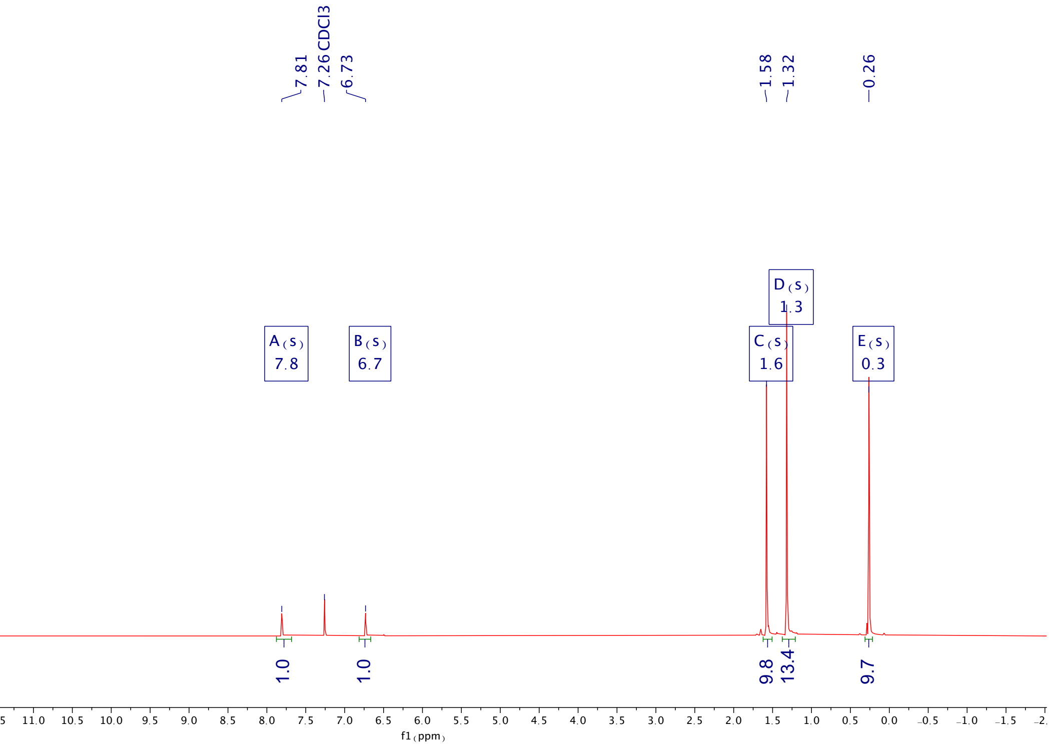

$^{13}C\{^{1}H\}$ (126 MHz, $CDCl_3$) of *t*-butyl-4-(4,4,5,5-tetramethyl-1,3,2-dioxaborolan-2-yl)-2-(trimethylsilyl)pyrrole-1-carboxylate (1ab)

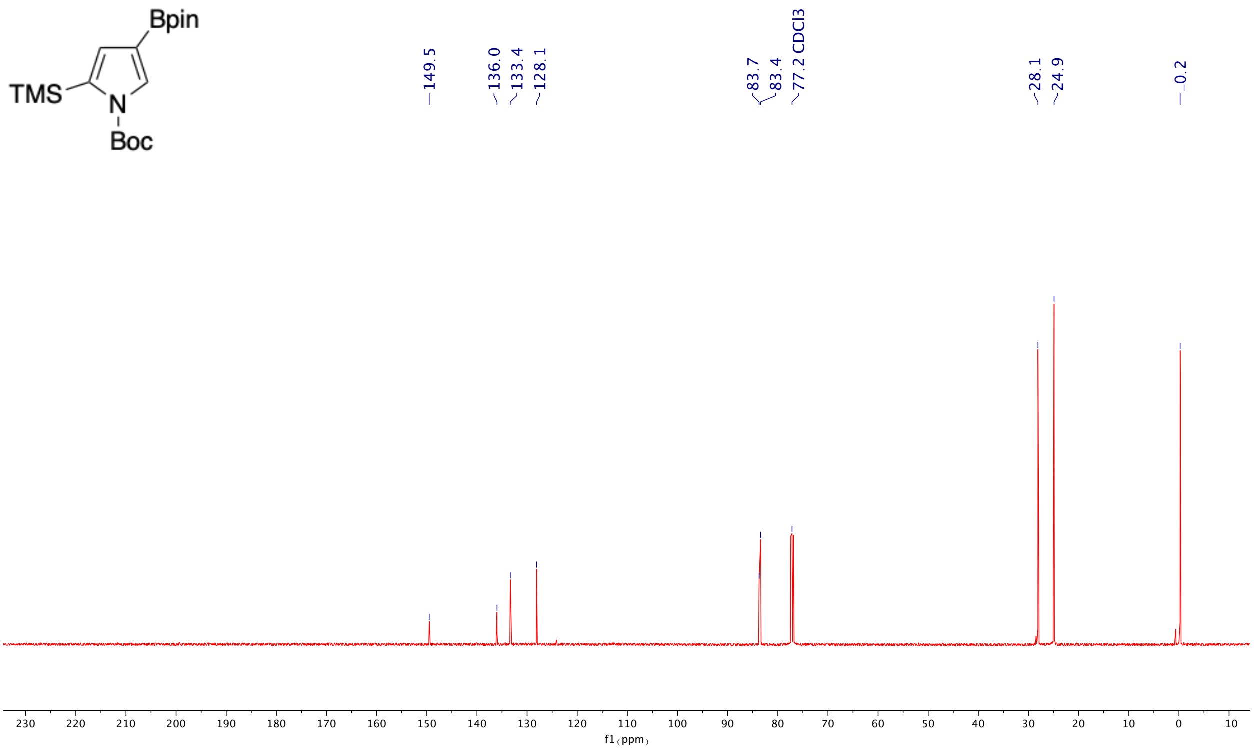

^{29}Si NMR (99 MHz, $CDCl_3$) of *t*-butyl-4-(4,4,5,5-tetramethyl-1,3,2-dioxaborolan-2-yl)-2-(trimethylsilyl)pyrrole-1-carboxylate (1ab)

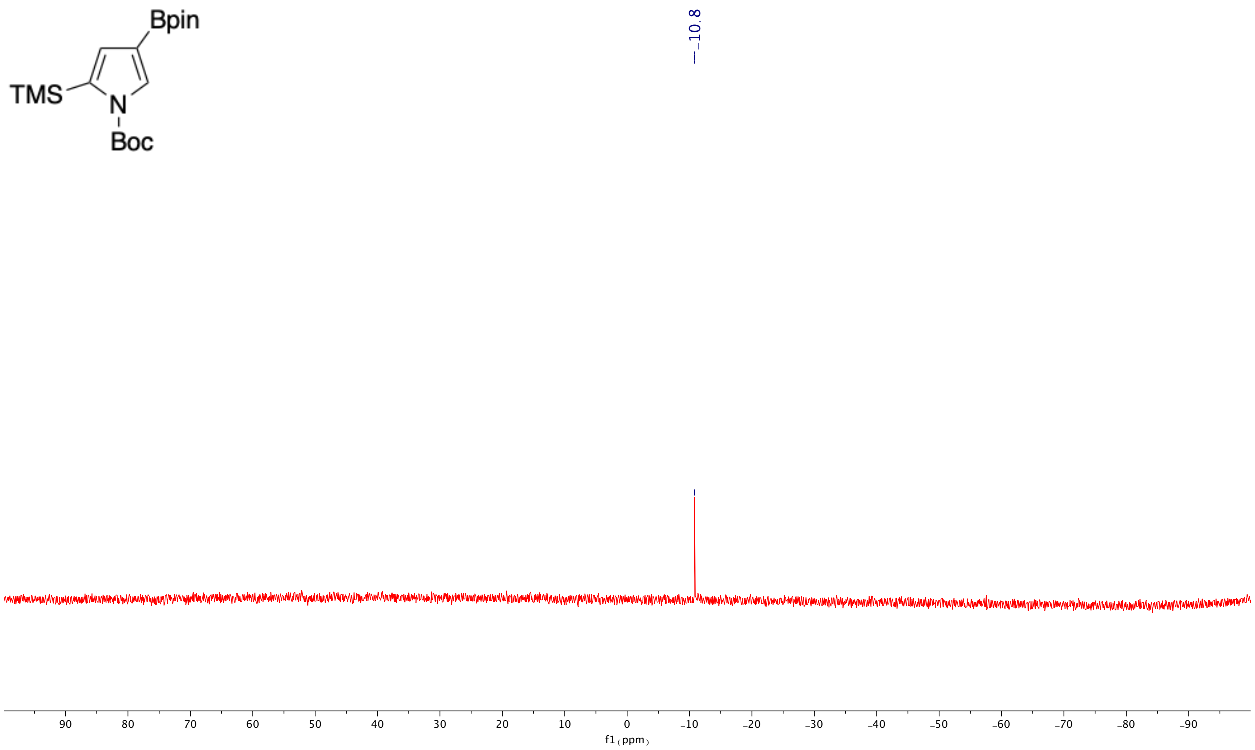

^{11}B NMR (160 MHz, $CDCl_3$) of *t*-butyl-4-(4,4,5,5-tetramethyl-1,3,2-dioxaborolan-2-yl)-2-(trimethylsilyl)pyrrole-1-carboxylate (1ab)

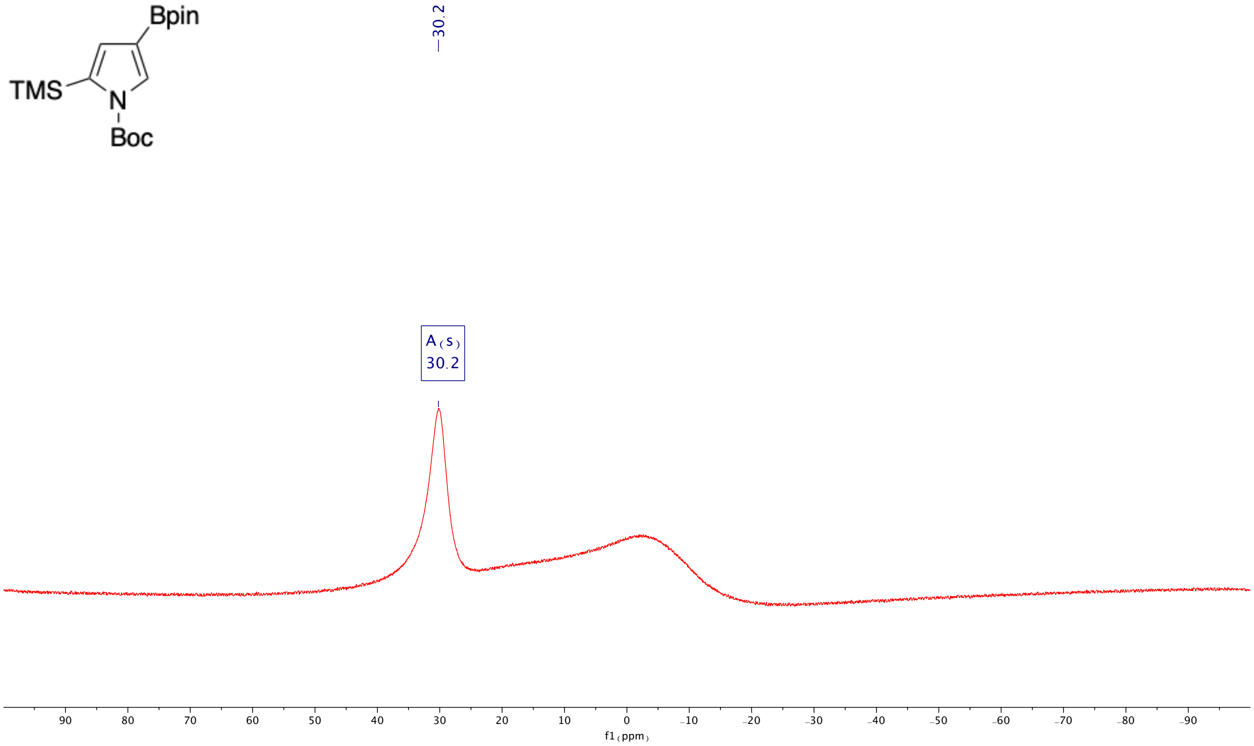

^{1}H NMR (500 MHz, $CDCl_3$) of 2-bromo-4-(4,4,5,5-tetramethyl-1,3,2-dioxaborolan-2-yl)-6-(trimethylsilyl)pyridine (1ac)

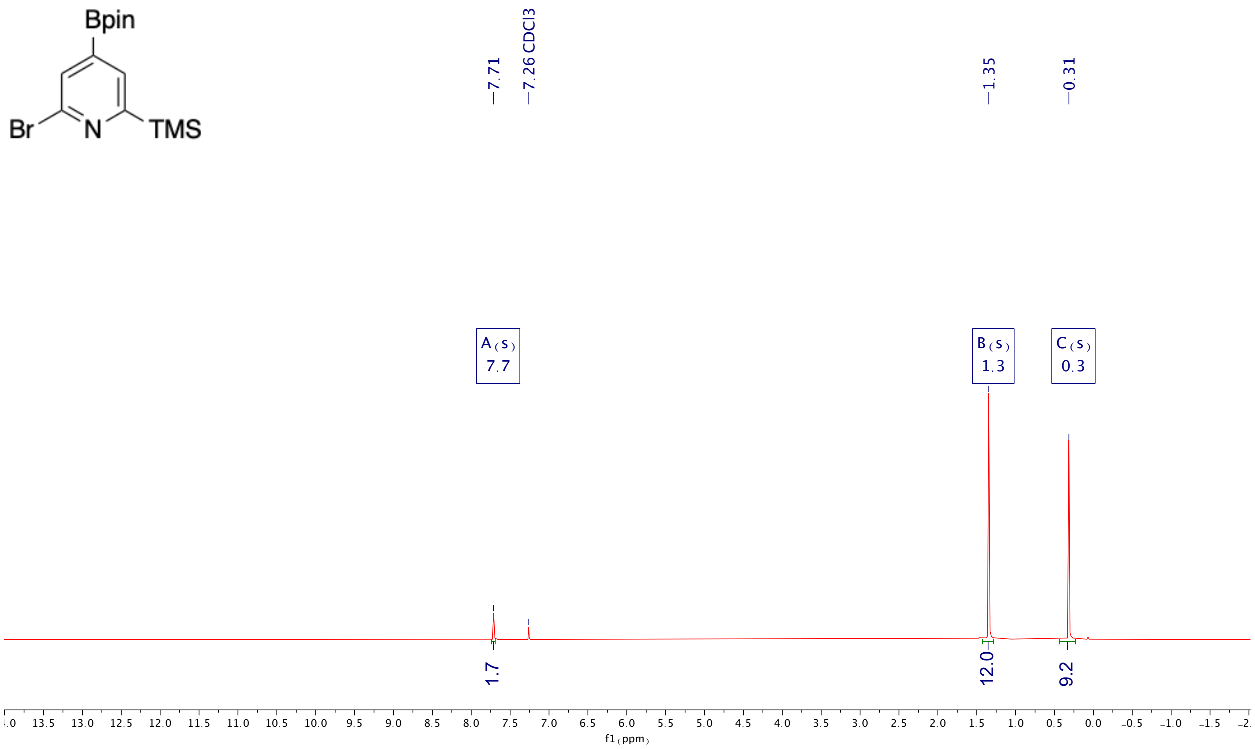

$^{13}C\{^{1}H\}$ (126 MHz, $CDCl_3$) NMR of 2-bromo-4-(4,4,5,5-tetramethyl-1,3,2-dioxaborolan-2-yl)-6-(trimethylsilyl)pyridine (1ac)

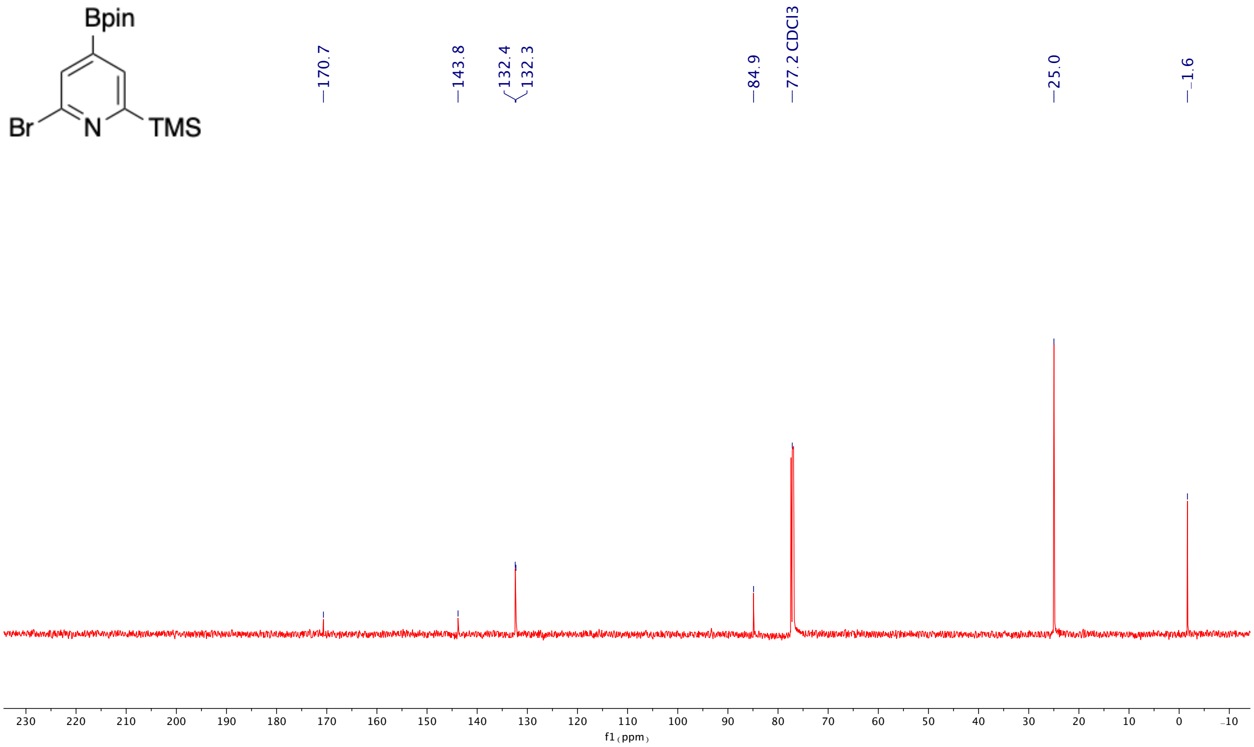

^{11}B NMR (160 MHz, $CDCl_3$) of 2-bromo-4-(4,4,5,5-tetramethyl-1,3,2-dioxaborolan-2-yl)-6-(trimethylsilyl)pyridine (1ac)

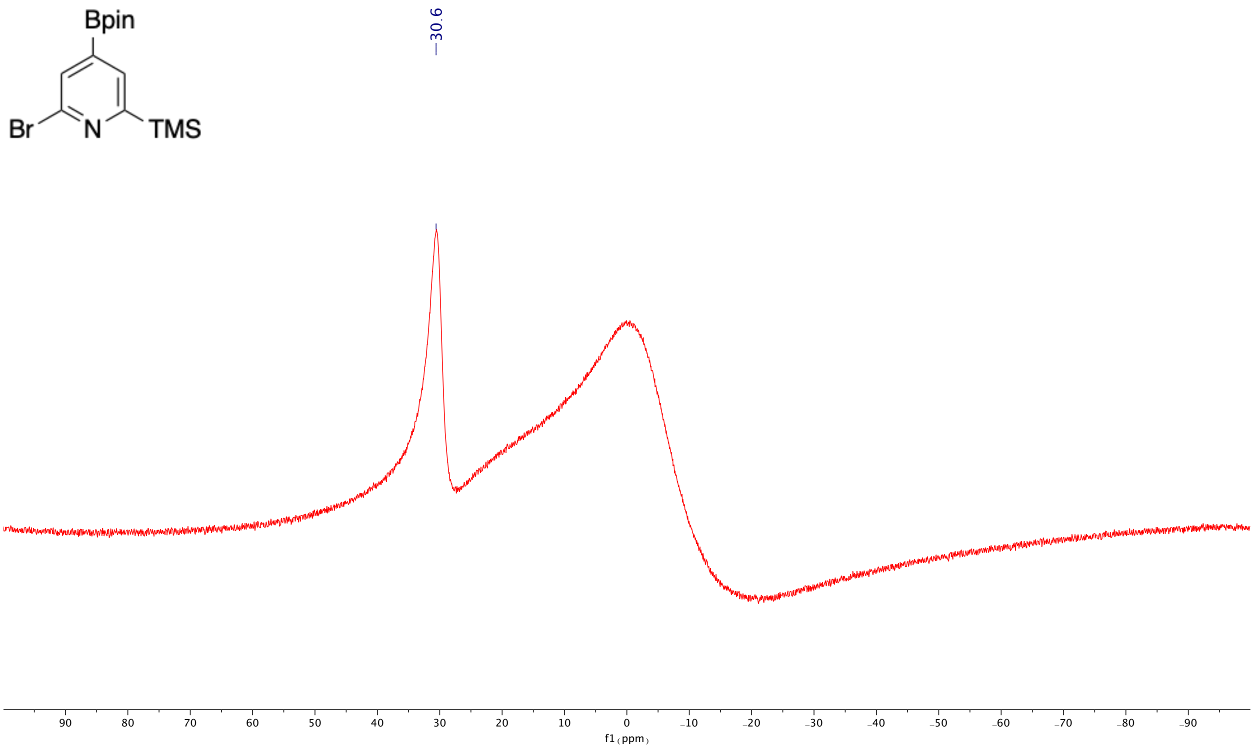

^{29}Si NMR (99 MHz, $CDCl_3$) of 2-bromo-4-(4,4,5,5-tetramethyl-1,3,2-dioxaborolan-2-yl)-6-(trimethylsilyl)pyridine (1ac)

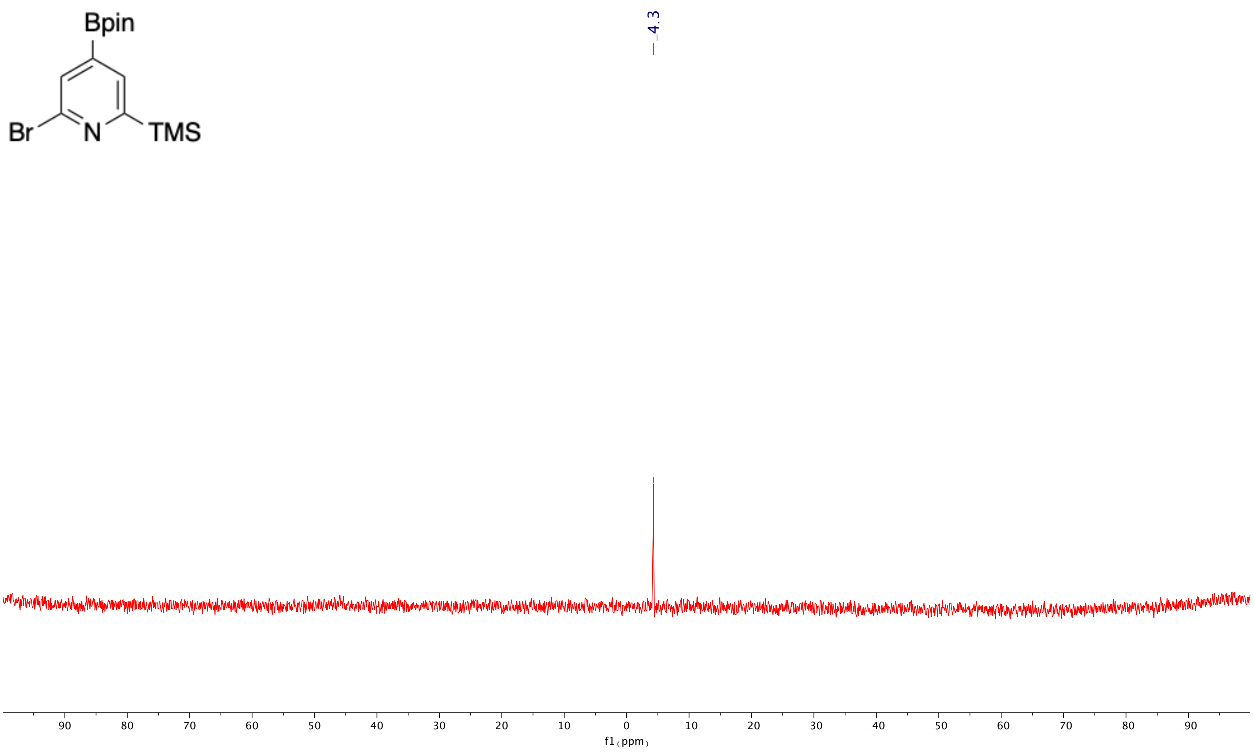

^{1}H NMR (500 MHz, $CDCl_3$) of trimethyl(2-(4,4,5,5-tetramethyl-1,3,2-dioxaborolan-2-yl)phenyl)silane (1ad)

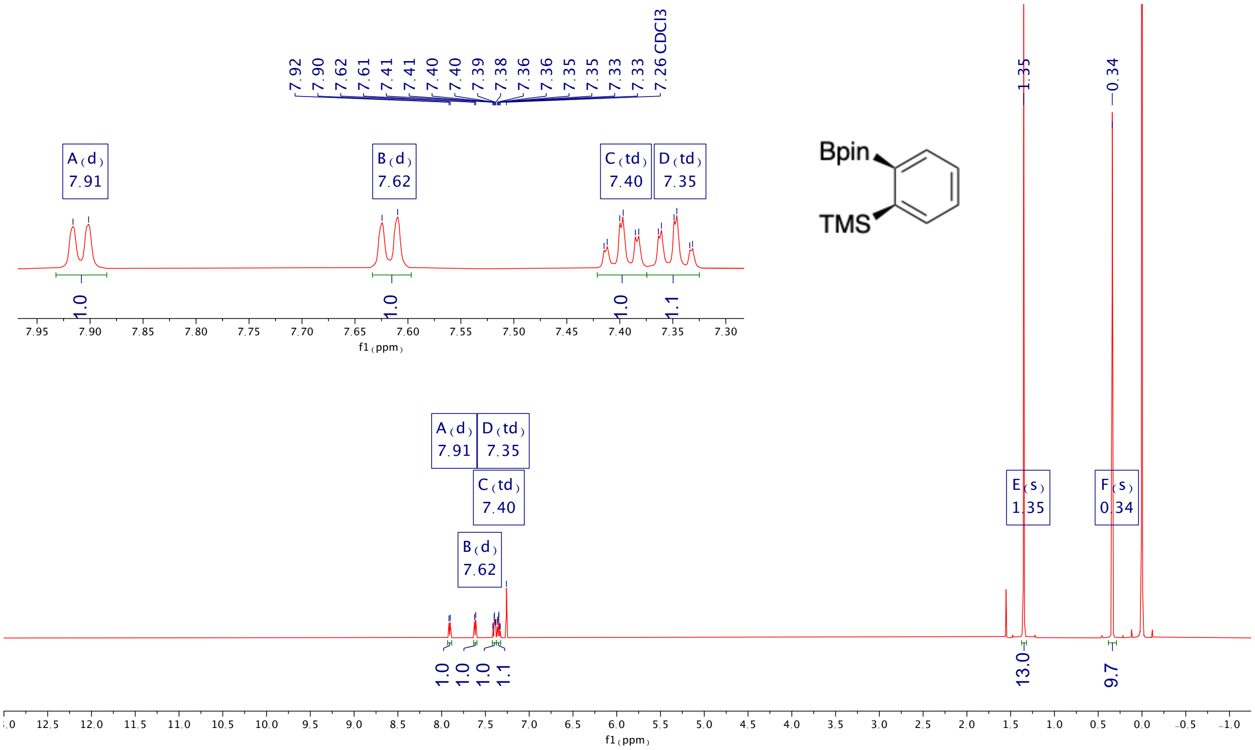

^{1}H NMR (500 MHz, $CDCl_3$) of trimethyl(2-(4,4,5,5-tetramethyl-1,3,2-dioxaborolan-2-yl)phenyl)silane (1v)

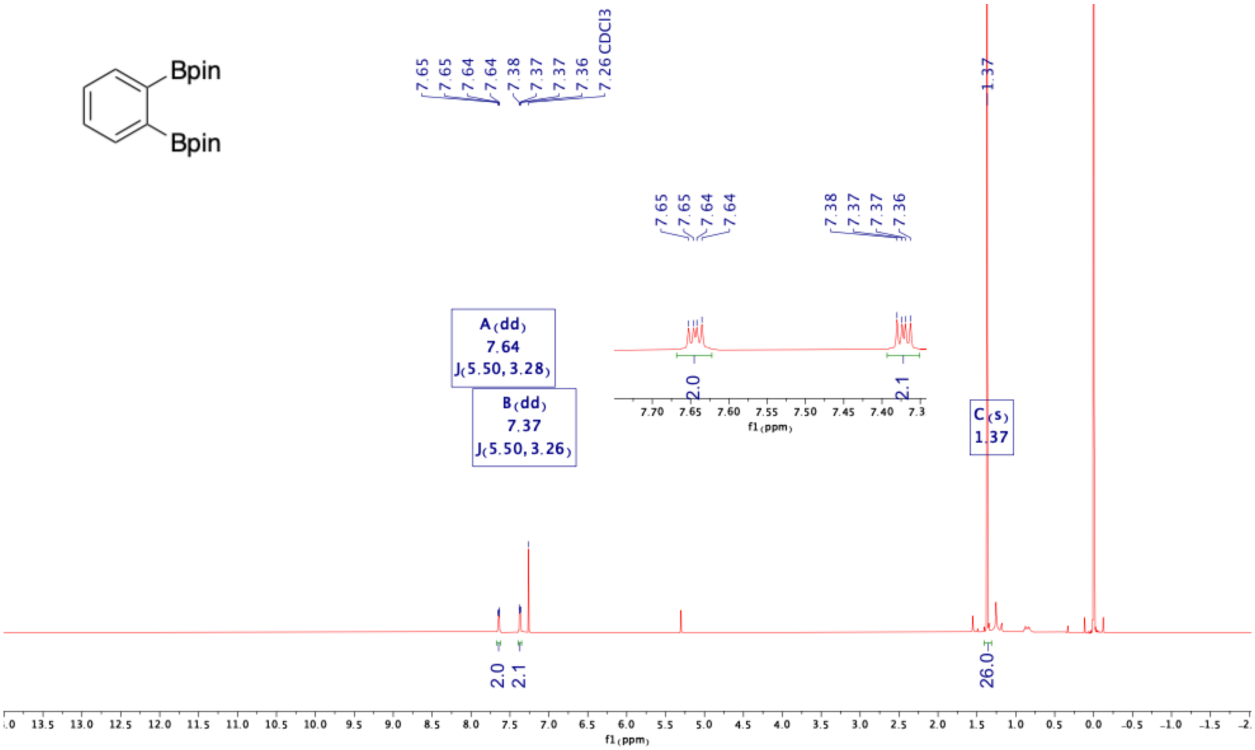

^{1}H NMR (500 MHz, D_2O) of 2-(4,4,5,5-tetramethyl-1,3,2-dioxaborolan-2-yl)piperidin-1-ium bromide (2c)

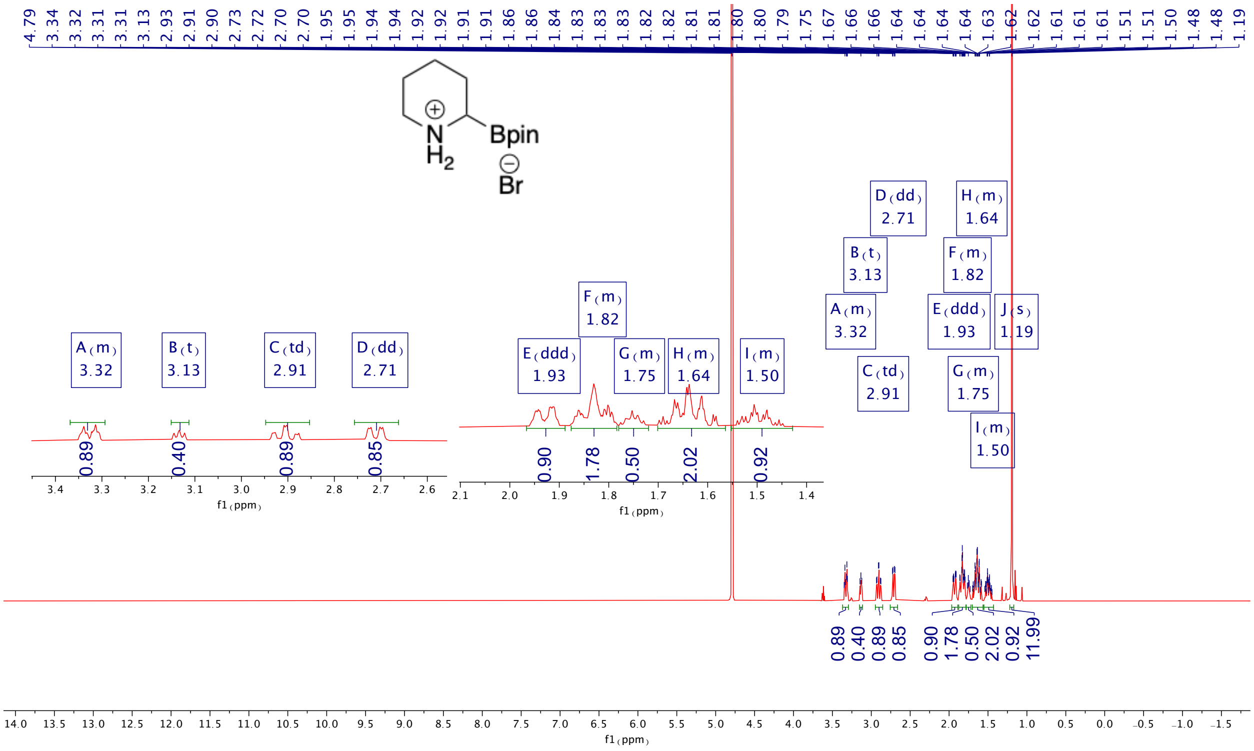

^{13}C{^{1}H} (126 MHz, D_2O) NMR of 2-(4,4,5,5-tetramethyl-1,3,2-dioxaborolan-2-yl)piperidin-1-ium bromide (2c)

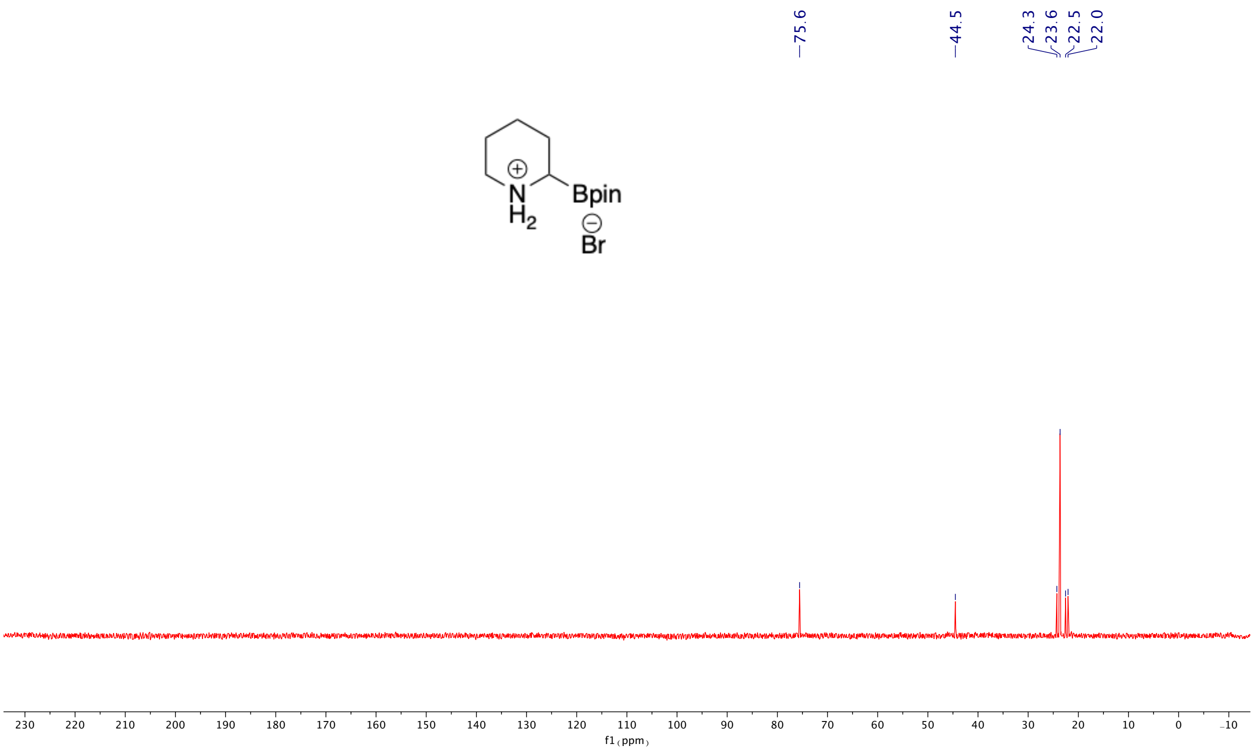

^{11}B NMR (160 MHz, D_2O) of 2-(4,4,5,5-tetramethyl-1,3,2-dioxaborolan-2-yl)piperidin-1-ium bromide (2c)

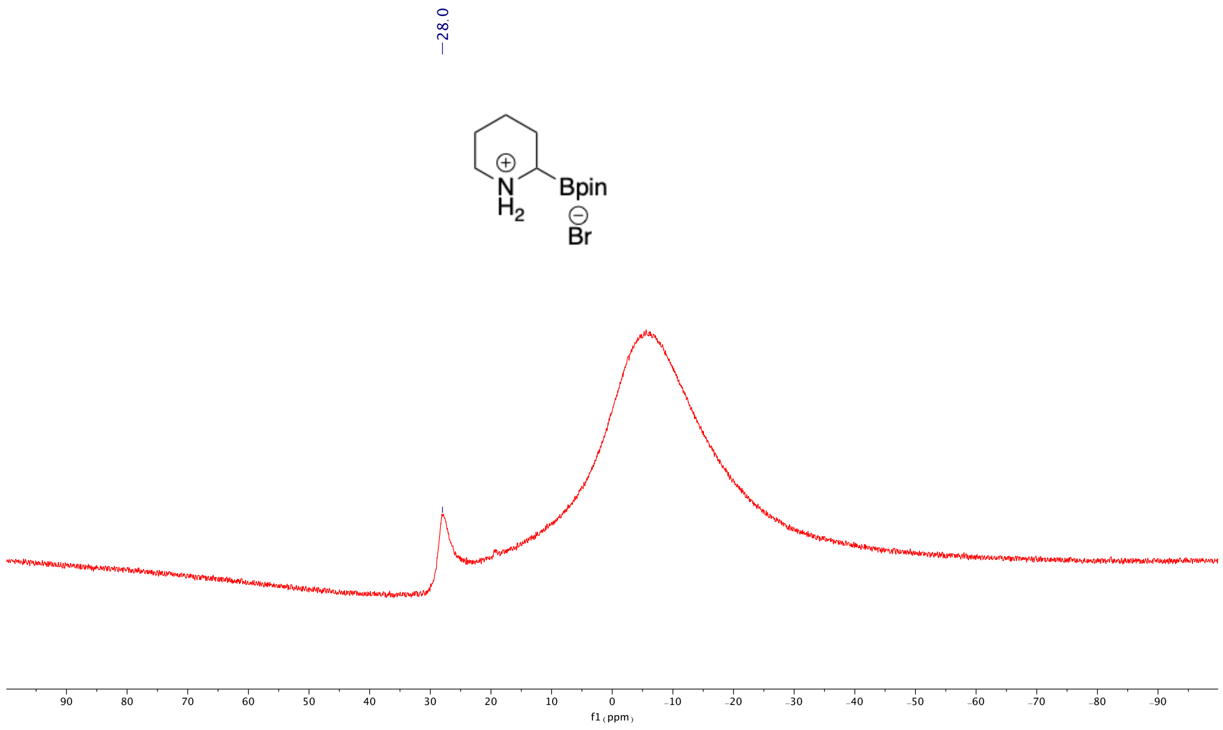

^{1}H NMR (500 MHz, D_2O) of 3-(4,4,5,5-Tetramethyl-1,3,2-dioxaborolan-2-yl)piperidin-1-ium bromide (2b)

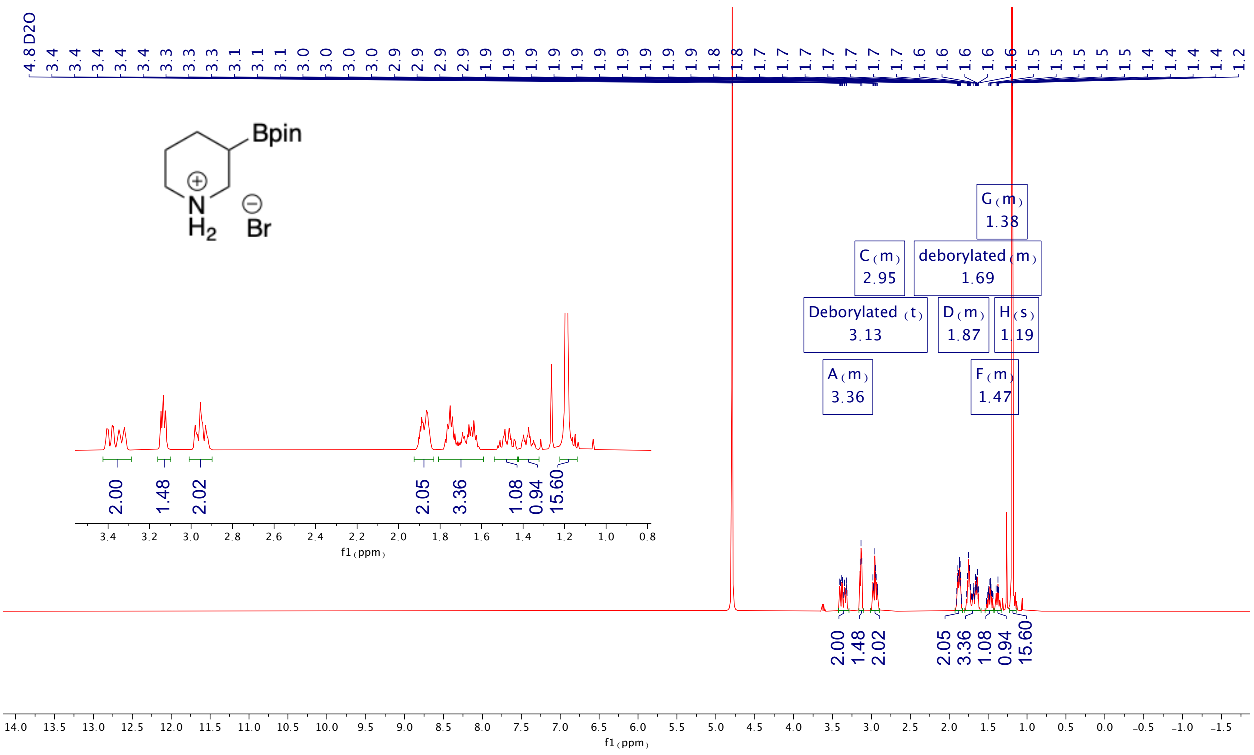

$^{13}C\{^1H\}$ (126 MHz, D_2O) NMR of 3-(4,4,5,5-Tetramethyl-1,3,2-dioxaborolan-2-yl)piperidin-1-ium bromide (2b)

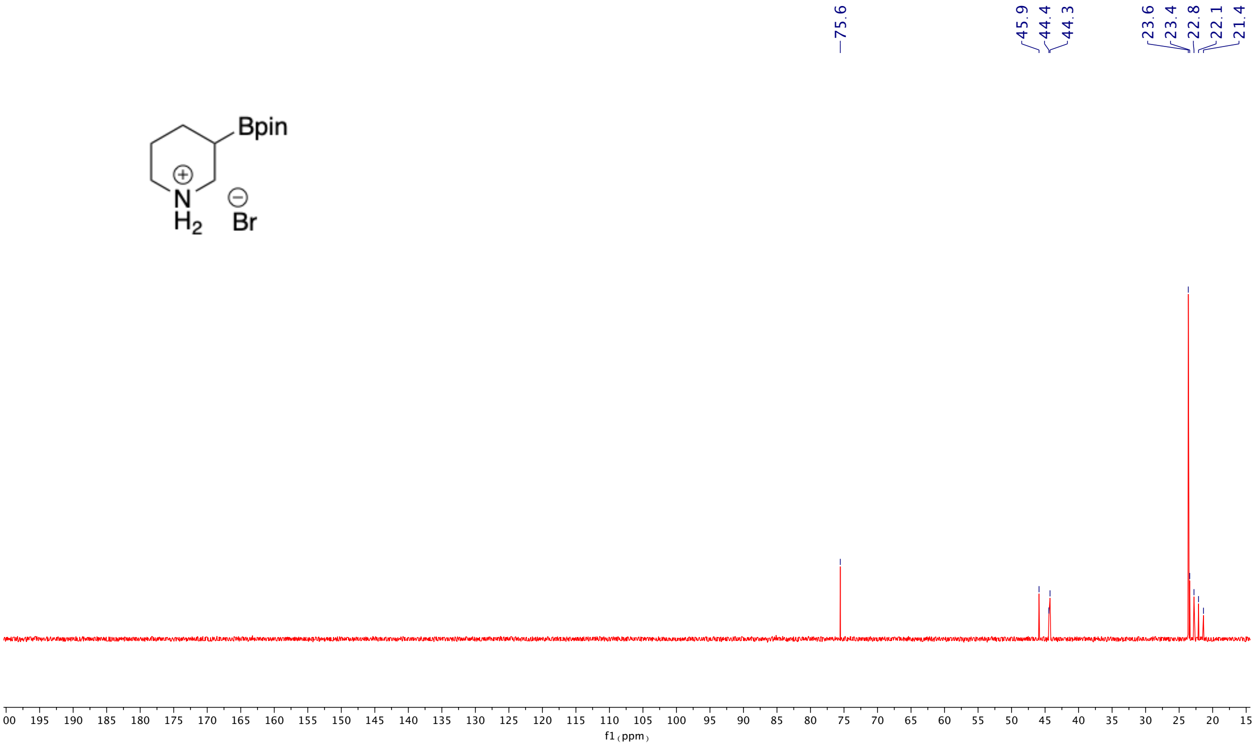

^{11}B NMR (160 MHz, D_2O) of 3-(4,4,5,5-Tetramethyl-1,3,2-dioxaborolan-2-yl)piperidin-1-ium bromide (2b)

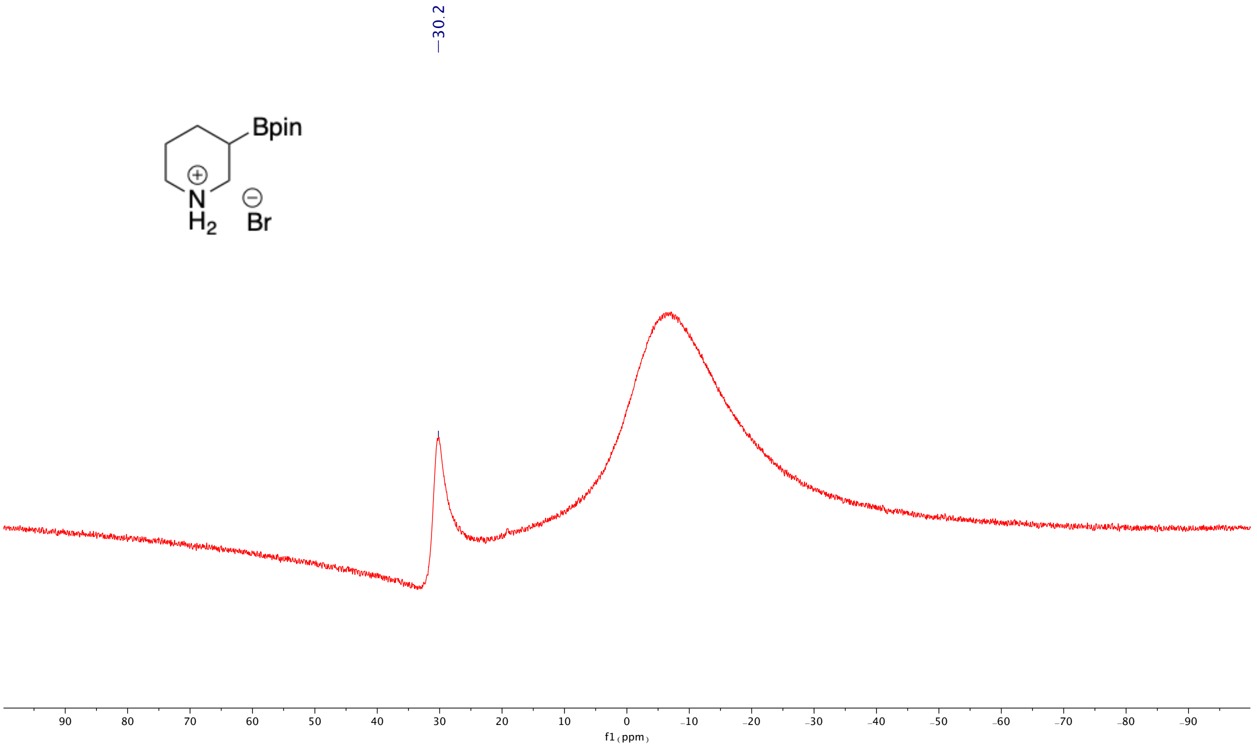

^{1}H NMR (500 MHz, DMSO) of 4-(4,4,5,5-tetramethyl-1,3,2-dioxaborolan-2-yl)piperidin-1-ium bromide (2d)

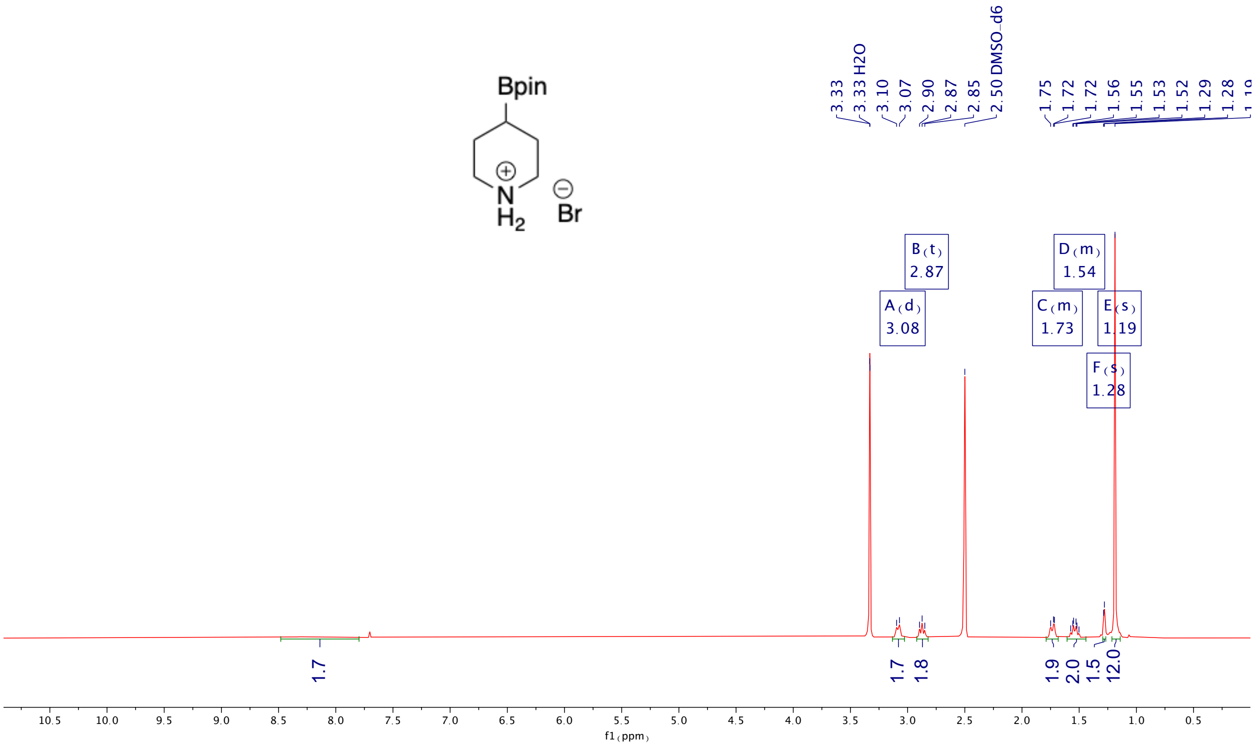

^{13}C{^{1}H} (126 MHz, DMSO) NMR of 4-(4,4,5,5-tetramethyl-1,3,2-dioxaborolan-2-yl)piperidin-1-ium bromide (2d)

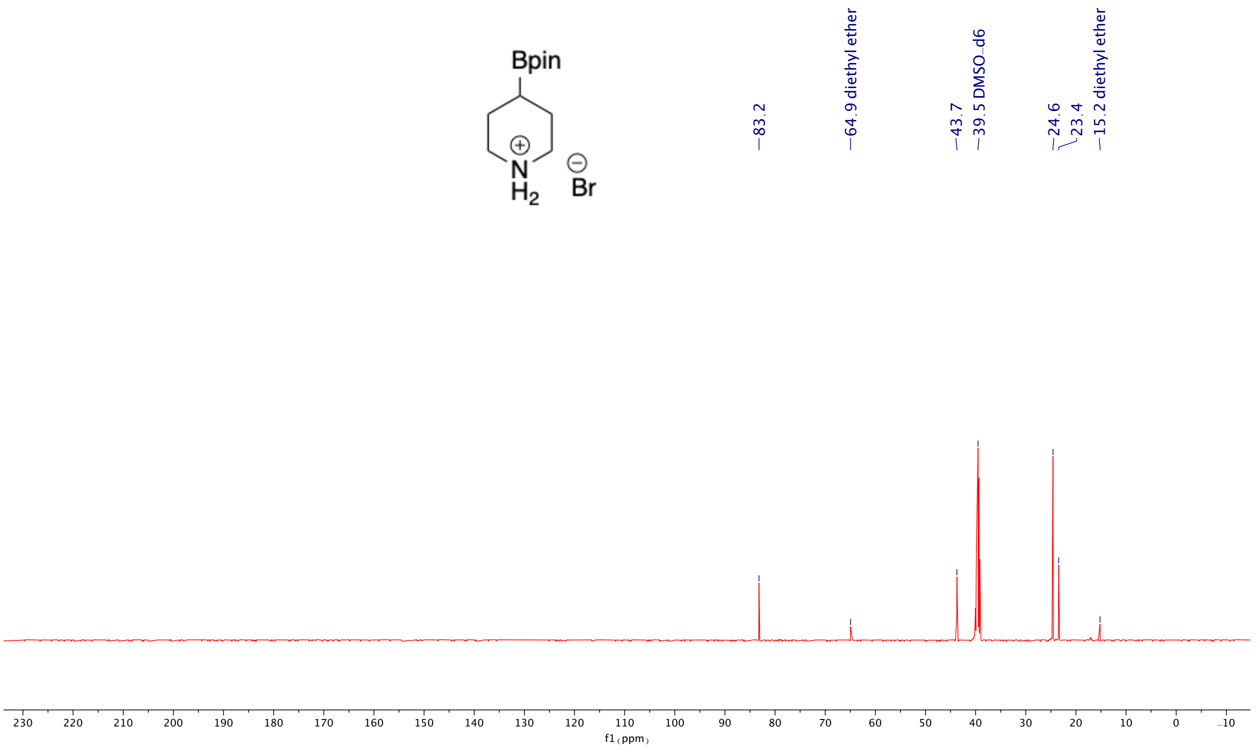

^{11}B NMR (160 MHz, DMSO) of 4-(4,4,5,5-tetramethyl-1,3,2-dioxaborolan-2-yl)piperidin-1-ium bromide (2d)

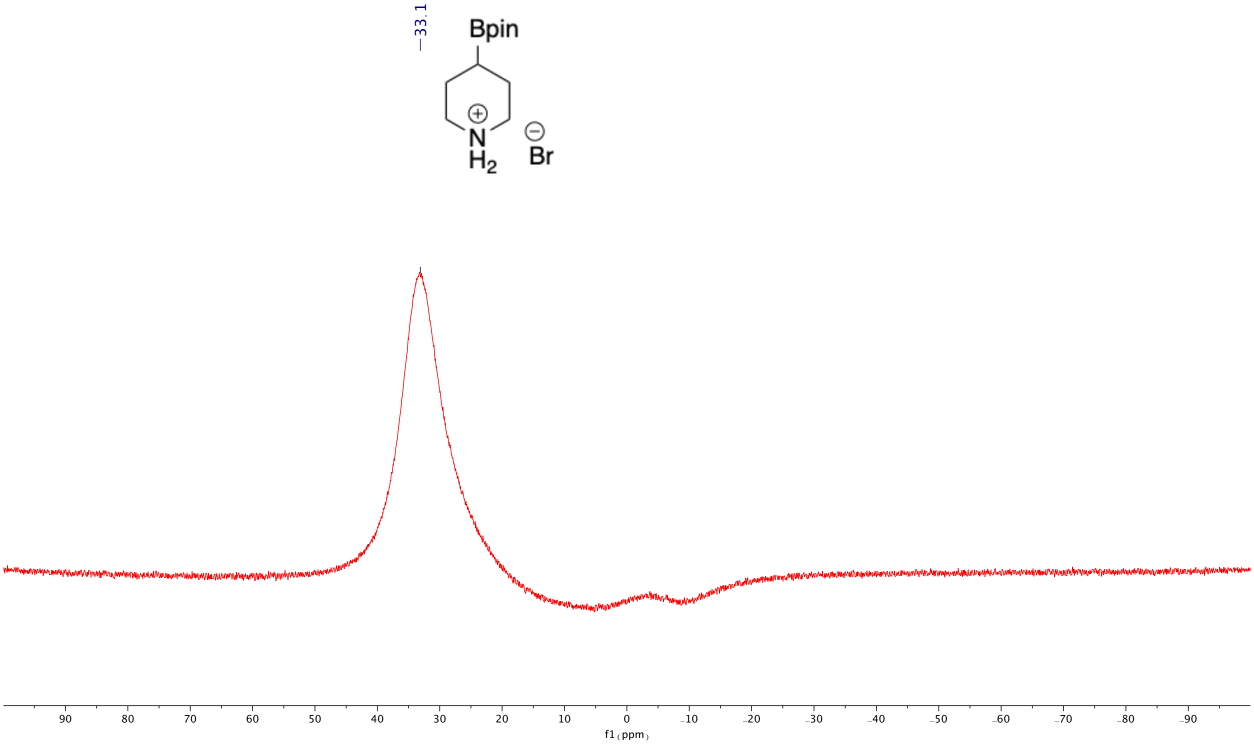

^{1}H NMR (500 MHz, D_2O) of 2-methyl-4-(4,4,5,5-tetramethyl-1,3,2-dioxaborolan-2-yl)piperidin-1-ium bromide (2e)

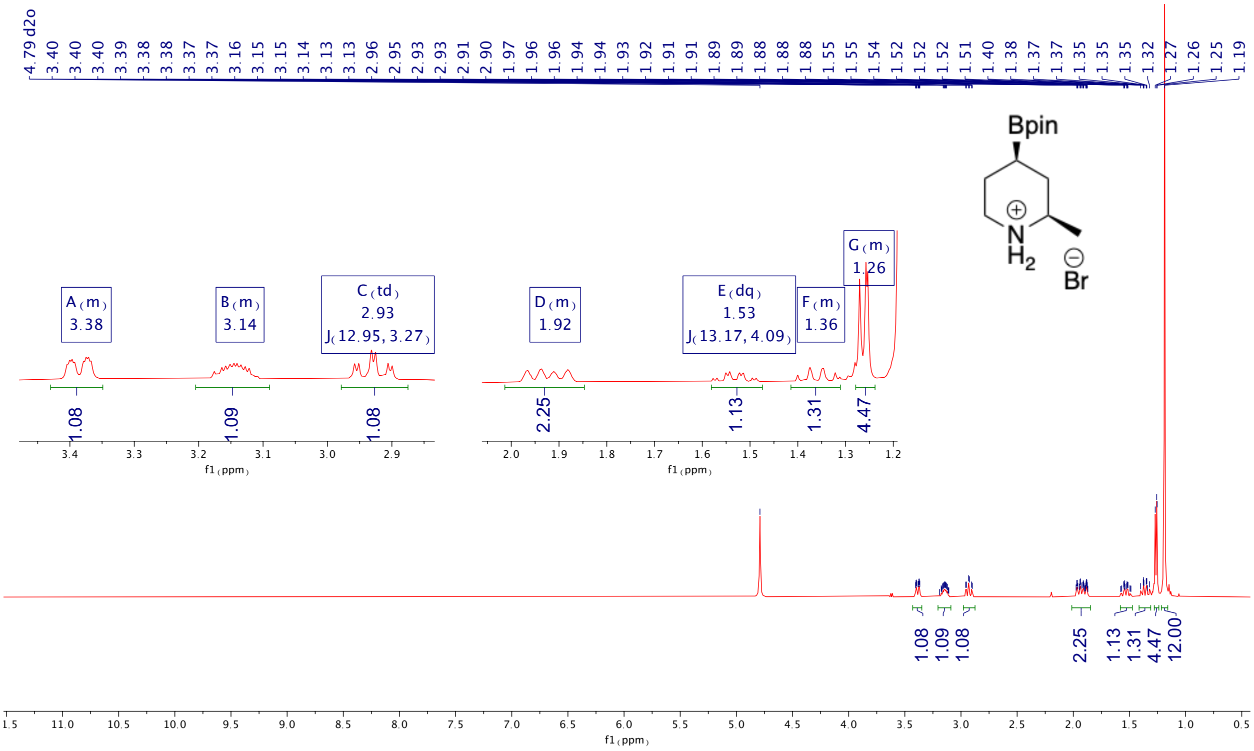

$^{13}C\{^{1}H\}$ (126 MHz, D_2O) NMR of 2-methyl-4-(4,4,5,5-tetramethyl-1,3,2-dioxaborolan-2-yl)piperidin-1-ium bromide (2e)

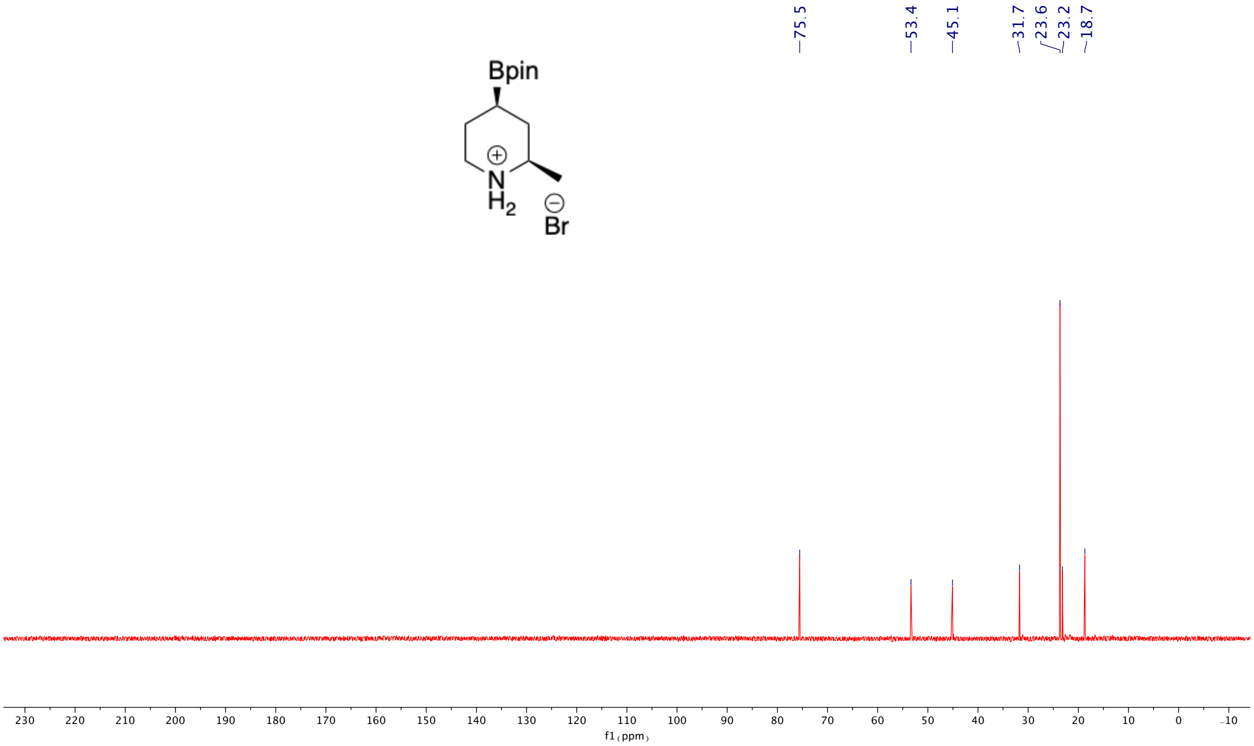

^{11}B NMR (160 MHz, D_2O) of 2-methyl-4-(4,4,5,5-tetramethyl-1,3,2-dioxaborolan-2-yl)piperidin-1-ium bromide (2e)

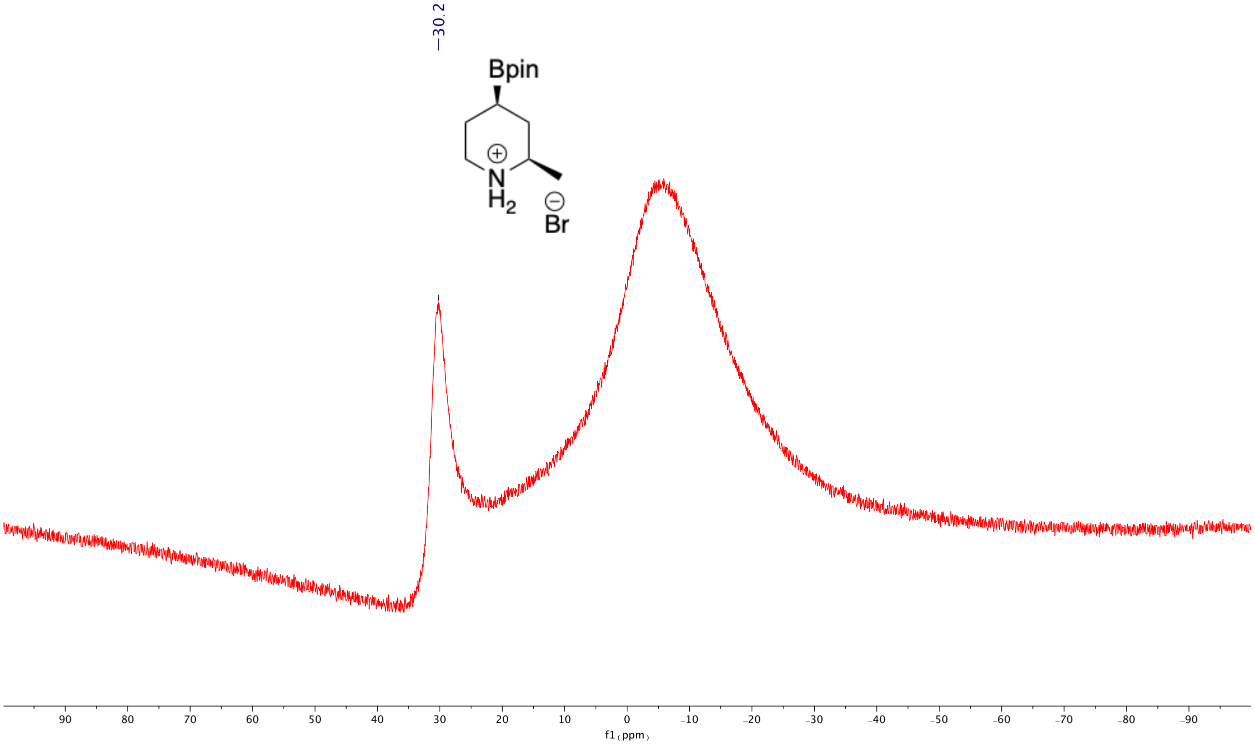

^{1}H NMR (500 MHz, $CDCl_3$) of 2,6-dimethyl-4-(4,4,5,5-tetramethyl-1,3,2-dioxaborolan-2-yl)piperidine (2f)

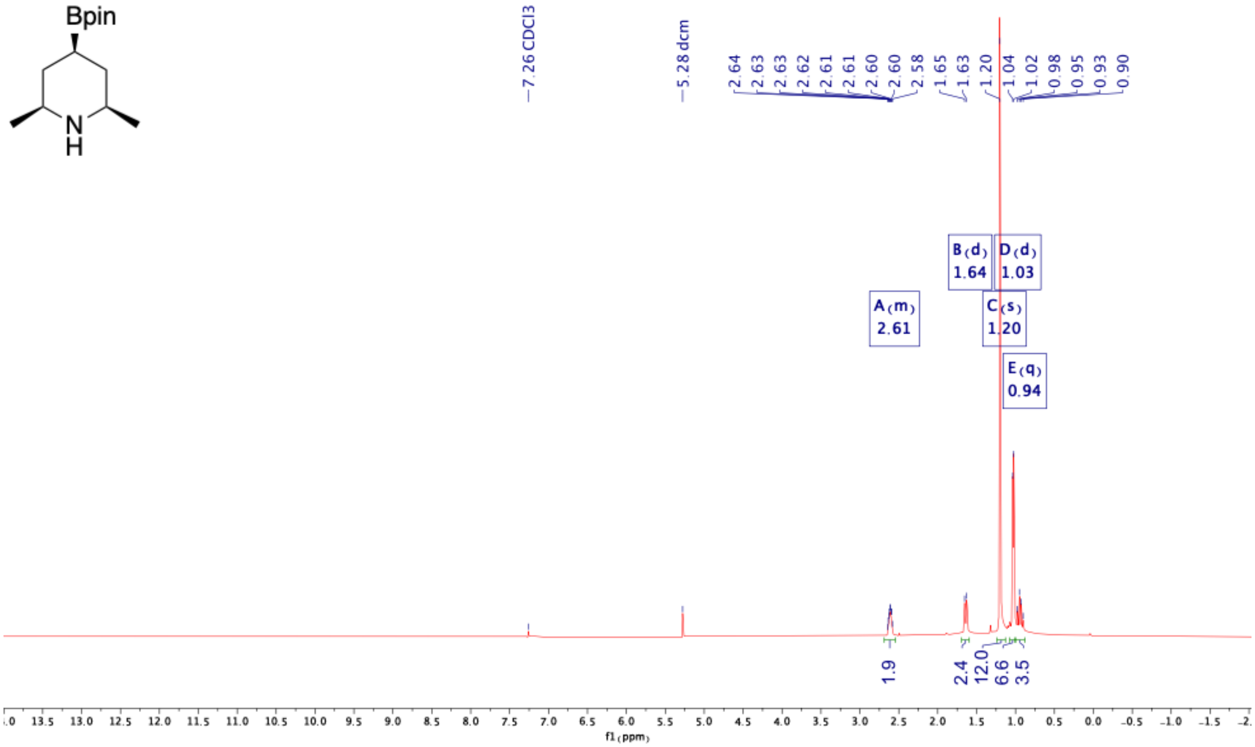

^{13}C{^{1}H} (126 MHz, $CDCl_3$) NMR of 2,6-dimethyl-4-(4,4,5,5-tetramethyl-1,3,2-dioxaborolan-2-yl)piperidine (2f)

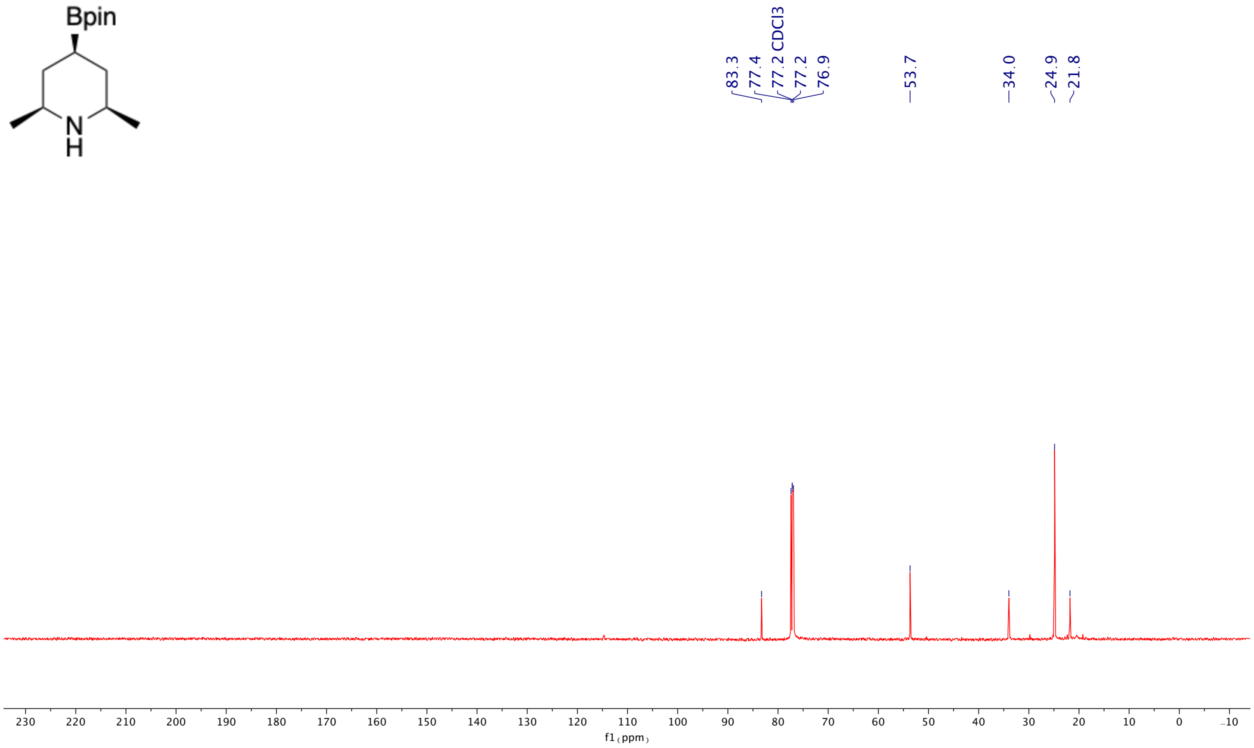

^{11}B NMR (160 MHz, $CDCl_3$) of 2,6-dimethyl-4-(4,4,5,5-tetramethyl-1,3,2-dioxaborolan-2-yl)piperidine (2f)

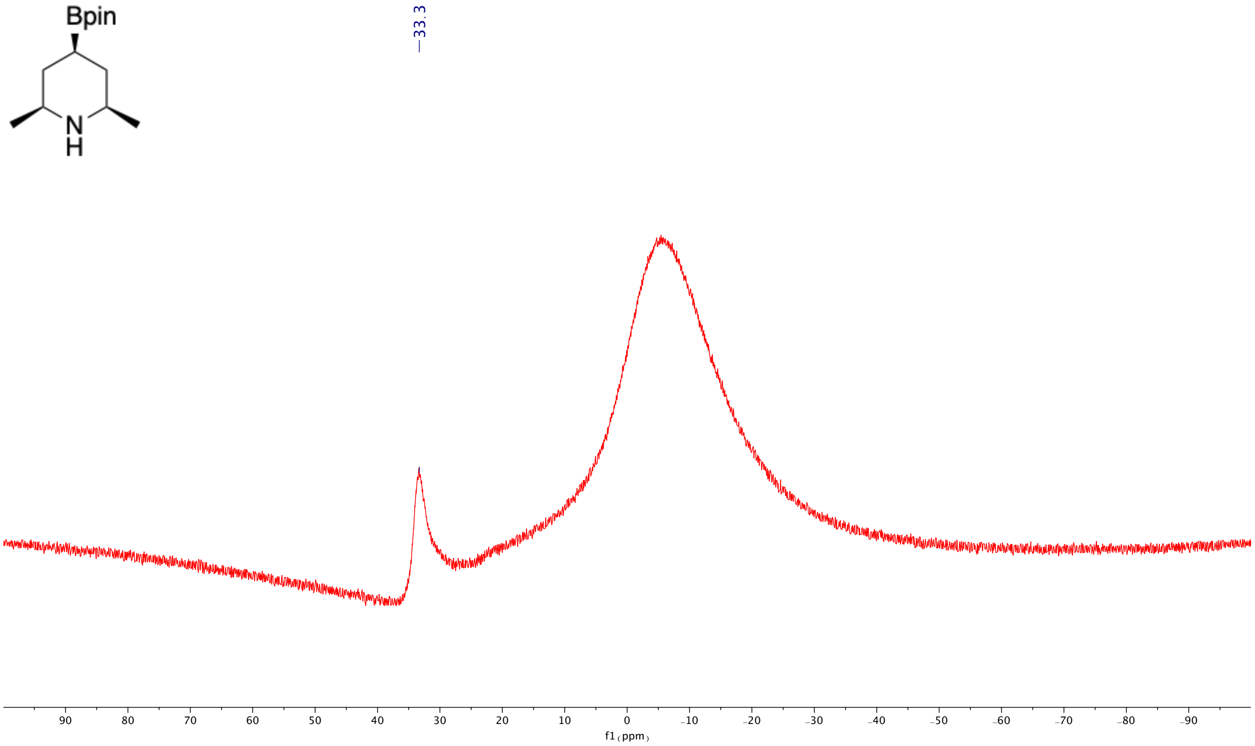

^{1}H NMR (500 MHz, C_6D_6) of *tert*-butyl 3-(4,4,5,5-tetramethyl-1,3,2-dioxaborolan-2-yl)pyrrolidine-1-carboxylate (2g)

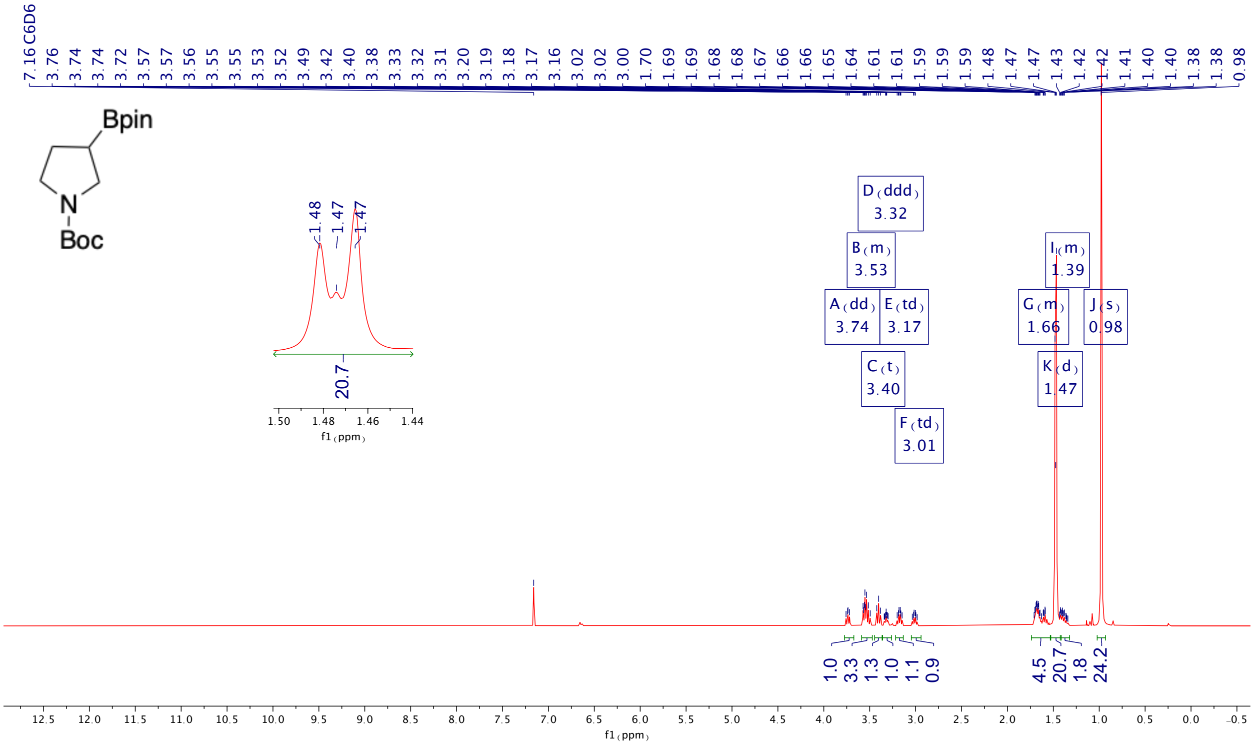

$^{13}C\{^1H\}$ (126 MHz, C_6D_6) NMR of *tert*-butyl 3-(4,4,5,5-tetramethyl-1,3,2-dioxaborolan-2-yl)pyrrolidine-1-carboxylate (2g)

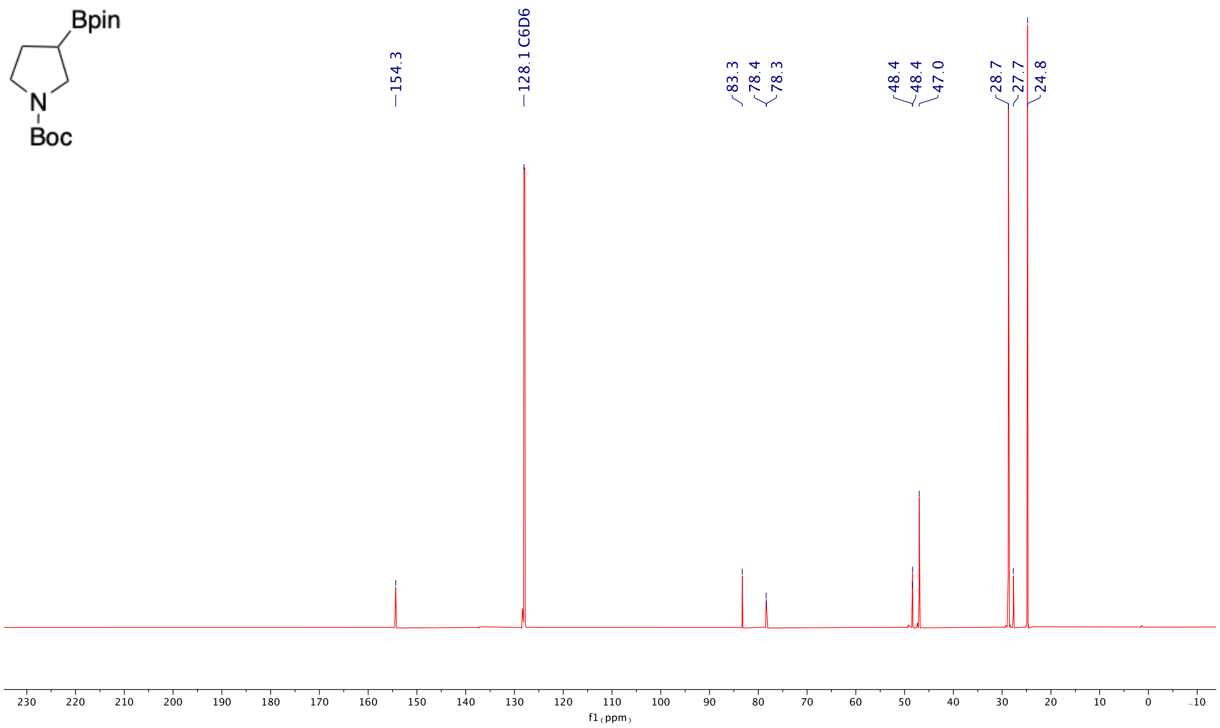

^{11}B NMR (160 MHz, C_6D_6) of *tert*-butyl 3-(4,4,5,5-tetramethyl-1,3,2-dioxaborolan-2-yl)pyrrolidine-1-carboxylate (2g)

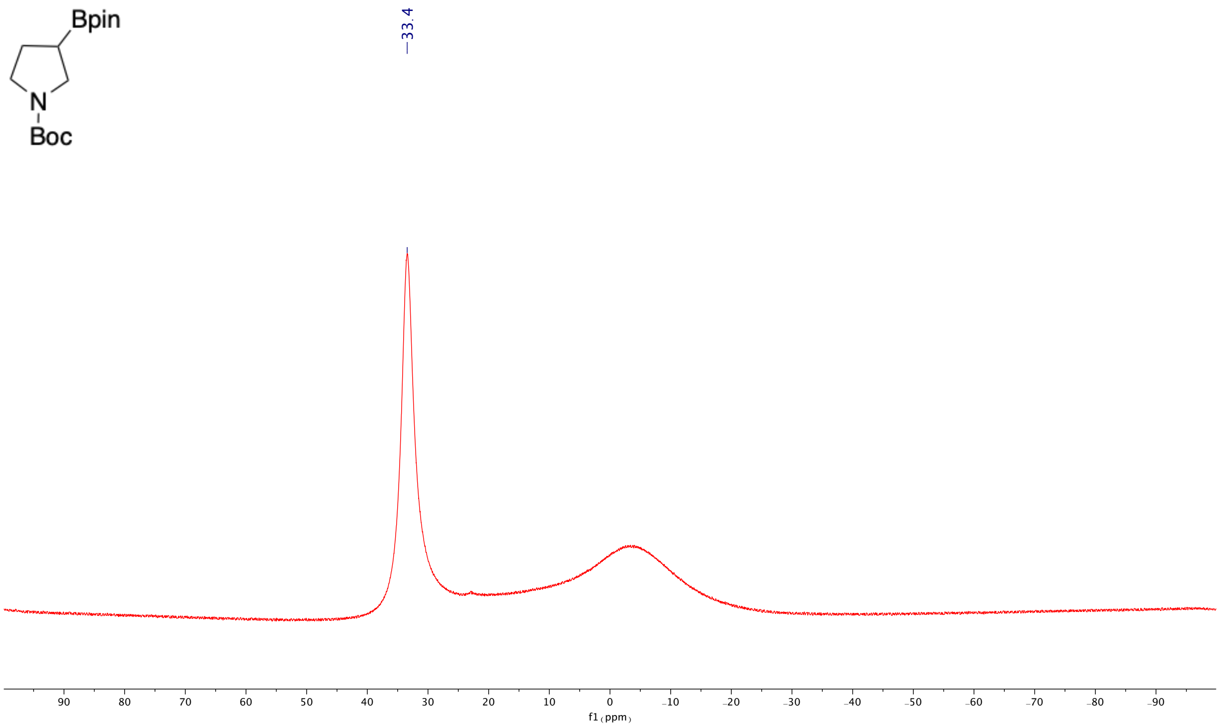

^{1}H NMR (500 MHz, $CDCl_3$) of 4,4,5,5-tetramethyl-2-(tetrahydrofuran-2-yl)-1,3,2-dioxaborolane (2h)

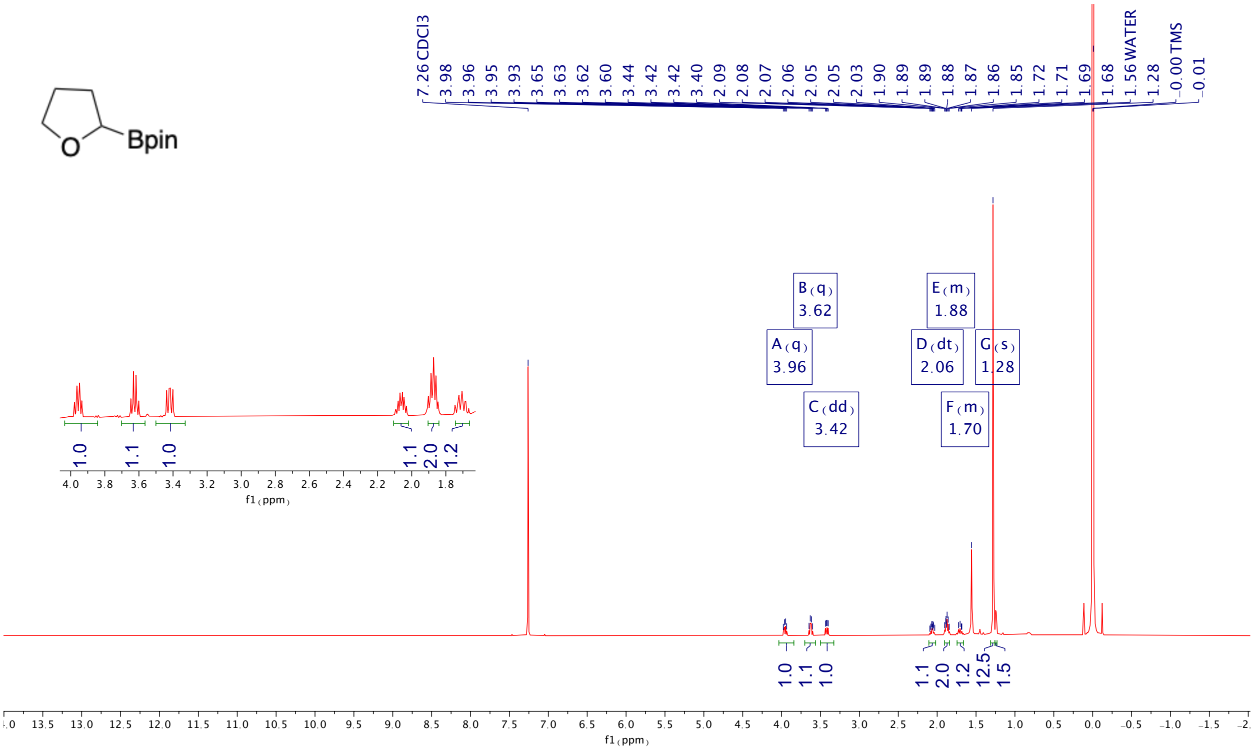

$^{13}C\{^{1}H\}$ (126 MHz, $CDCl_3$) NMR of 4,4,5,5-tetramethyl-2-(tetrahydrofuran-2-yl)-1,3,2-dioxaborolane (2h)

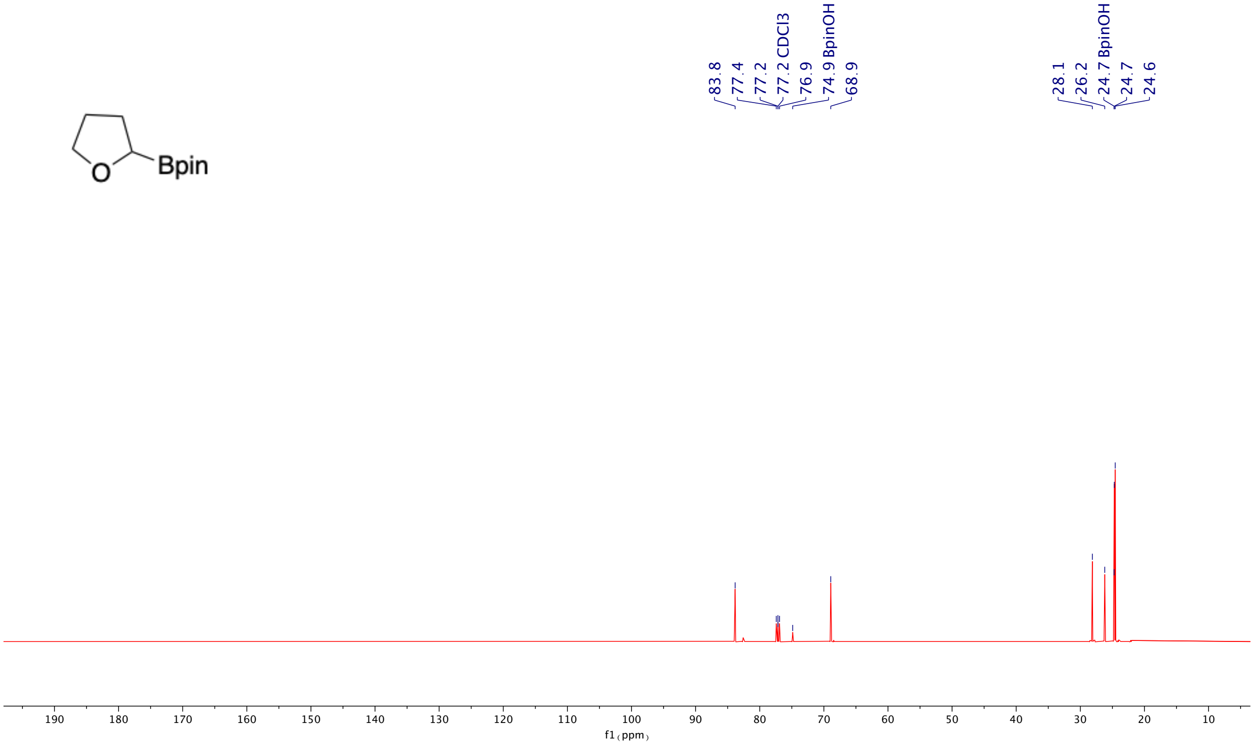

^{11}B NMR (160MHz, $CDCl_3$) of 4,4,5,5-tetramethyl-2-(tetrahydrofuran-2-yl)-1,3,2-dioxaborolane (2h)

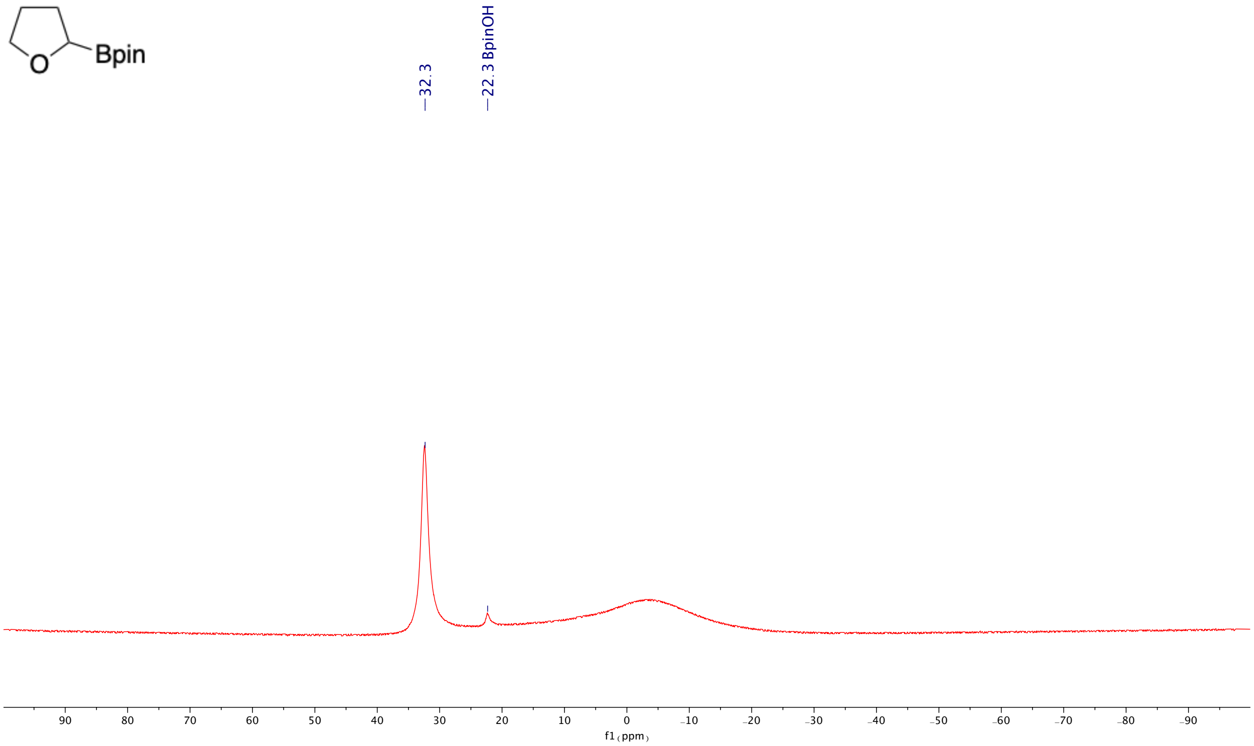

^{1}H NMR (500MHz, C_6D_6) of 4,4,5,5-Tetramethyl-2-(5-methyltetrahydrofuran-2-yl)-1,3,2-dioxaborolane (2i)

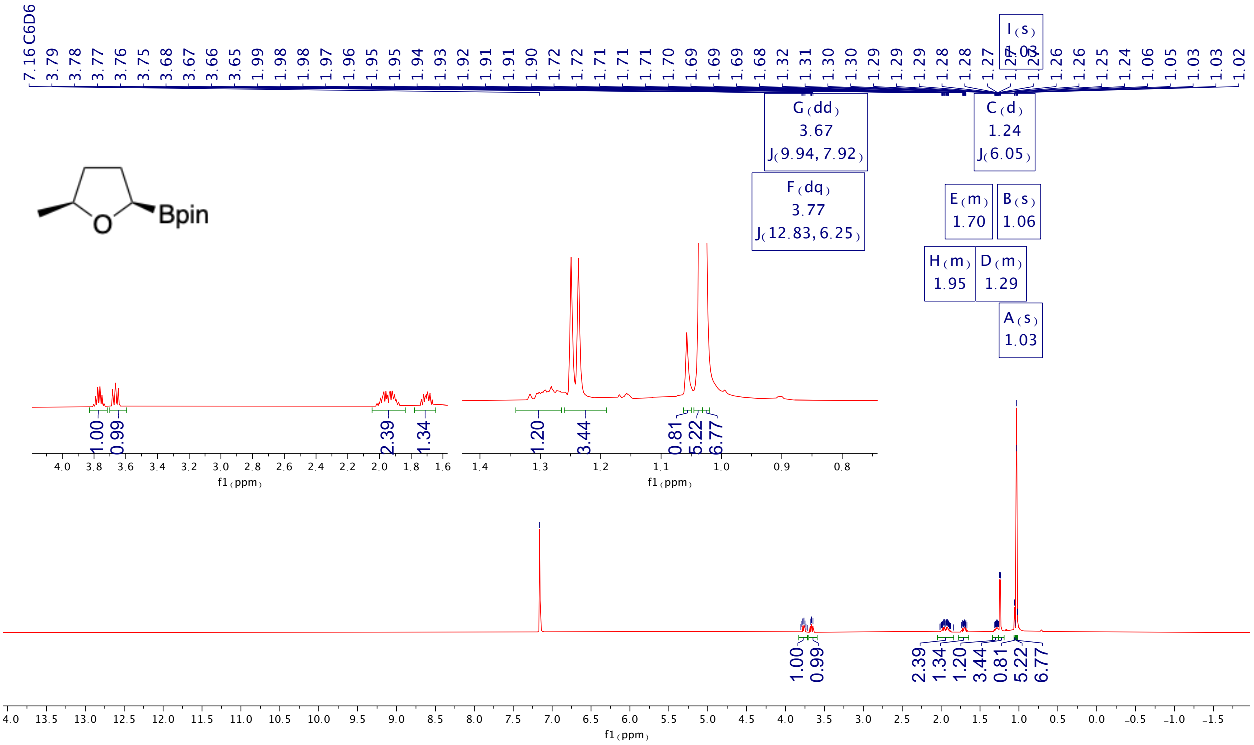

$^{13}C\{^{1}H\}$ (126 MHz, C_6D_6) NMR of 4,4,5,5-Tetramethyl-2-(5-methyltetrahydrofuran-2-yl)-1,3,2-dioxaborolane (2i)

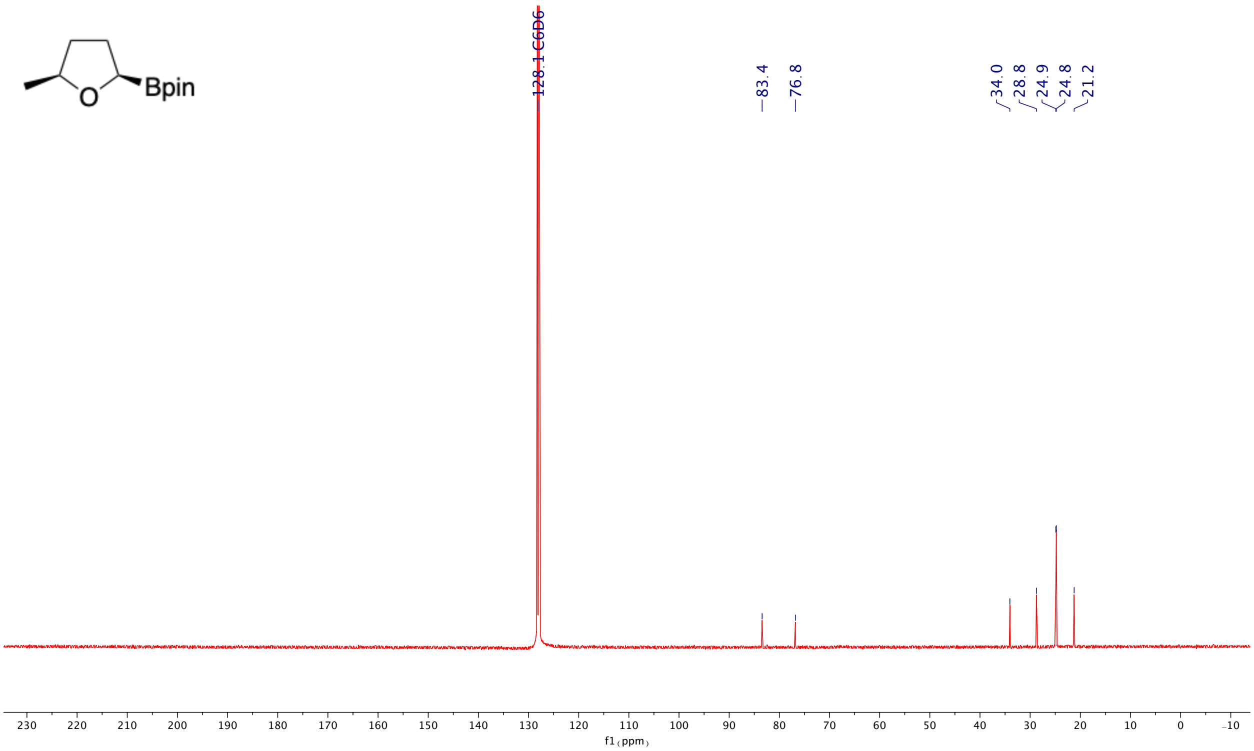

^{11}B NMR (160MHz, $CDCl_3$) of 4,4,5,5-Tetramethyl-2-(5-methyltetrahydrofuran-2-yl)-1,3,2-dioxaborolane (2i)

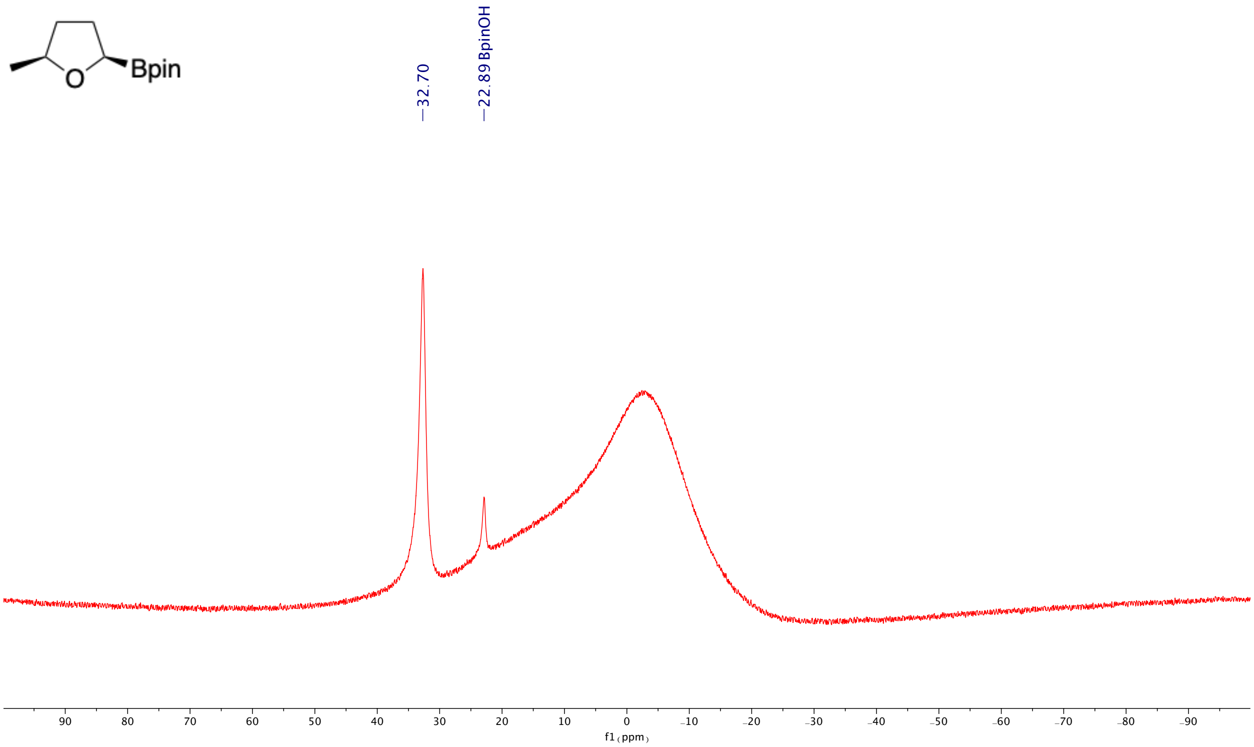

1D-NOE NMR of 4,4,5,5-Tetramethyl-2-(5-methyltetrahydrofuran-2-yl)-1,3,2-dioxaborolane (2i)

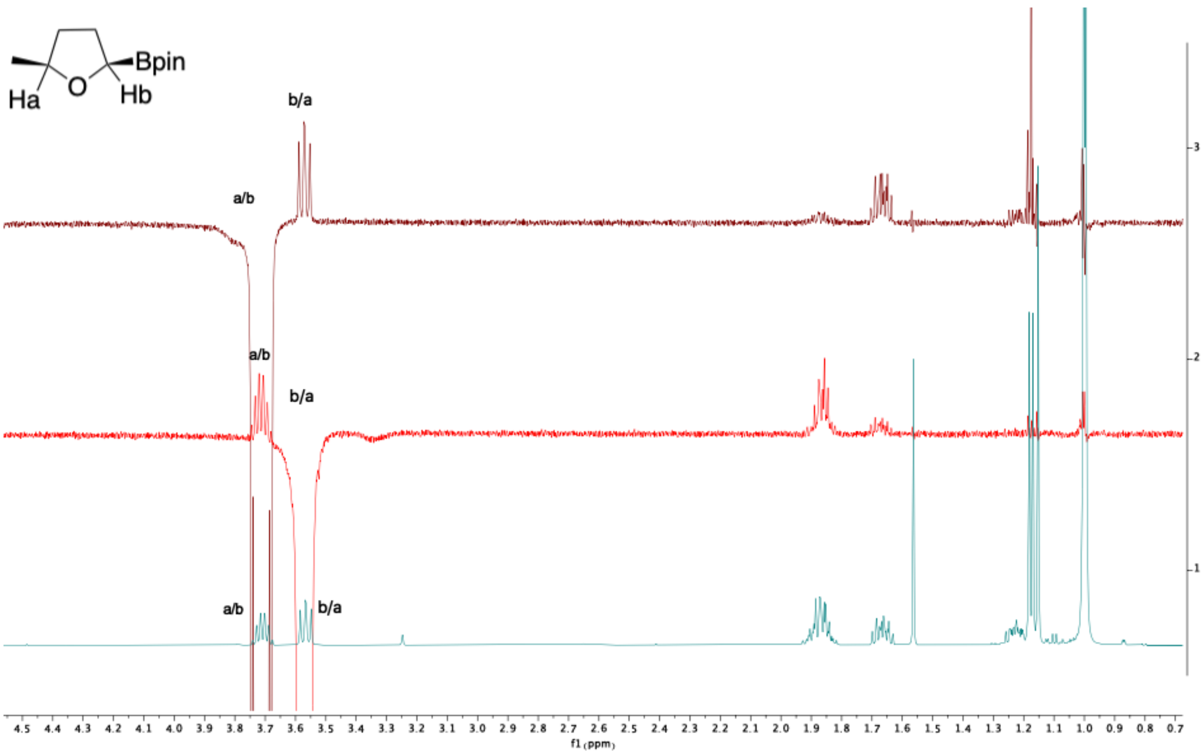

^{1}H NMR (500MHz, $CDCl_3$) of 4,4,5,5-tetramethyl-2-(octahydrobenzofuran-2-yl)-1,3,2-dioxaborolane (2j)

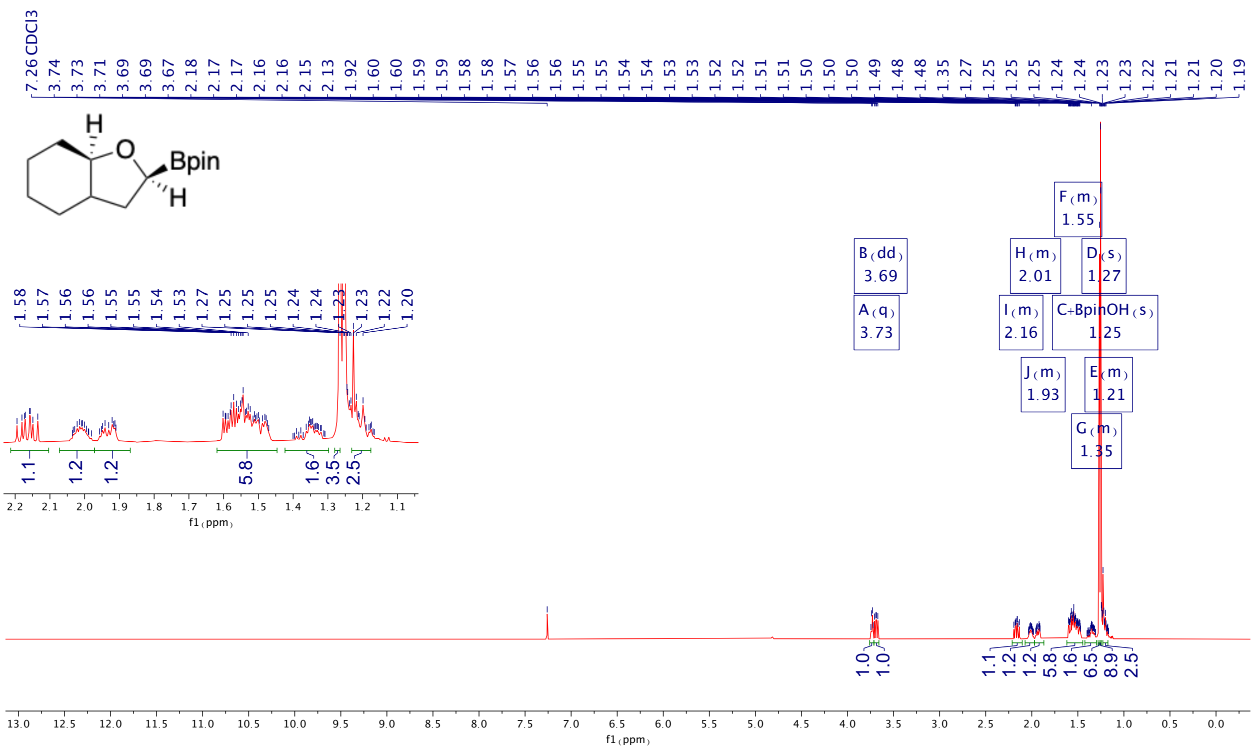

$^{13}C\{^{1}H\}$ (126 MHz, $CDCl_3$) NMR of 4,4,5,5-tetramethyl-2-(octahydrobenzofuran-2-yl)-1,3,2-dioxaborolane (2j)

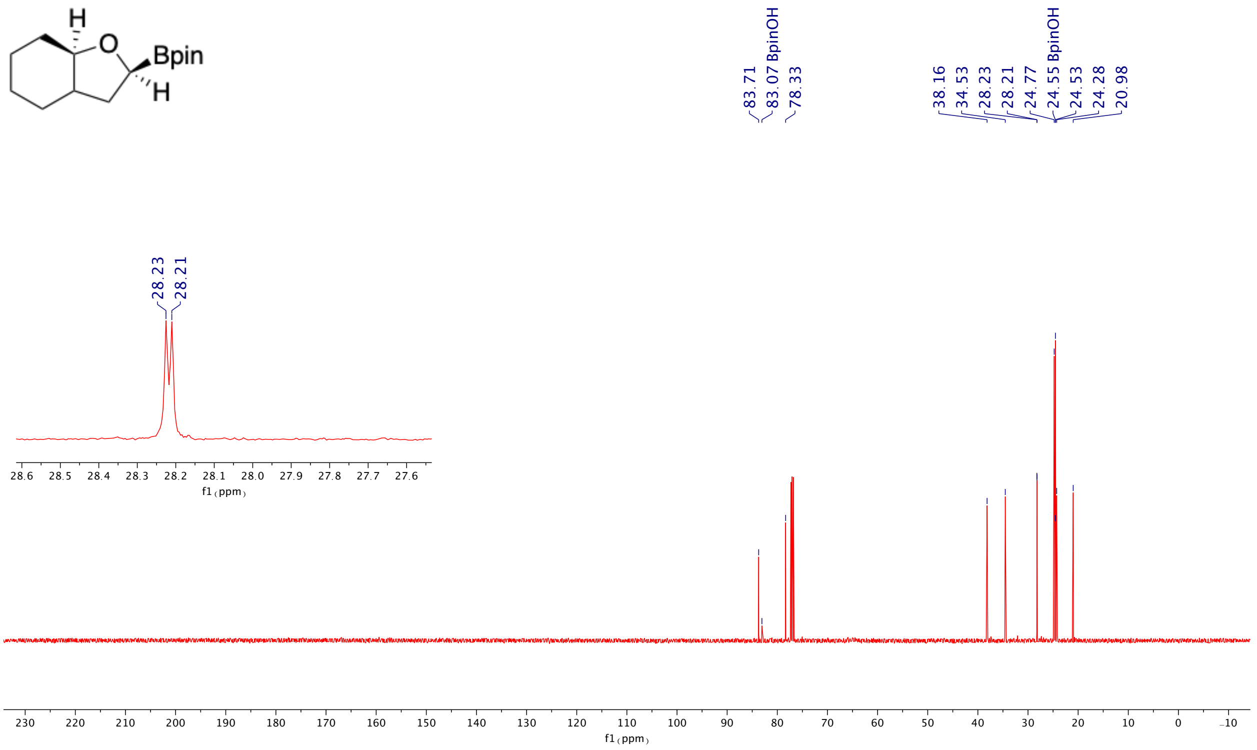

^{11}B NMR (160MHz, $CDCl_3$) of 4,4,5,5-tetramethyl-2-(octahydrobenzofuran-2-yl)-1,3,2-dioxaborolane (2j)

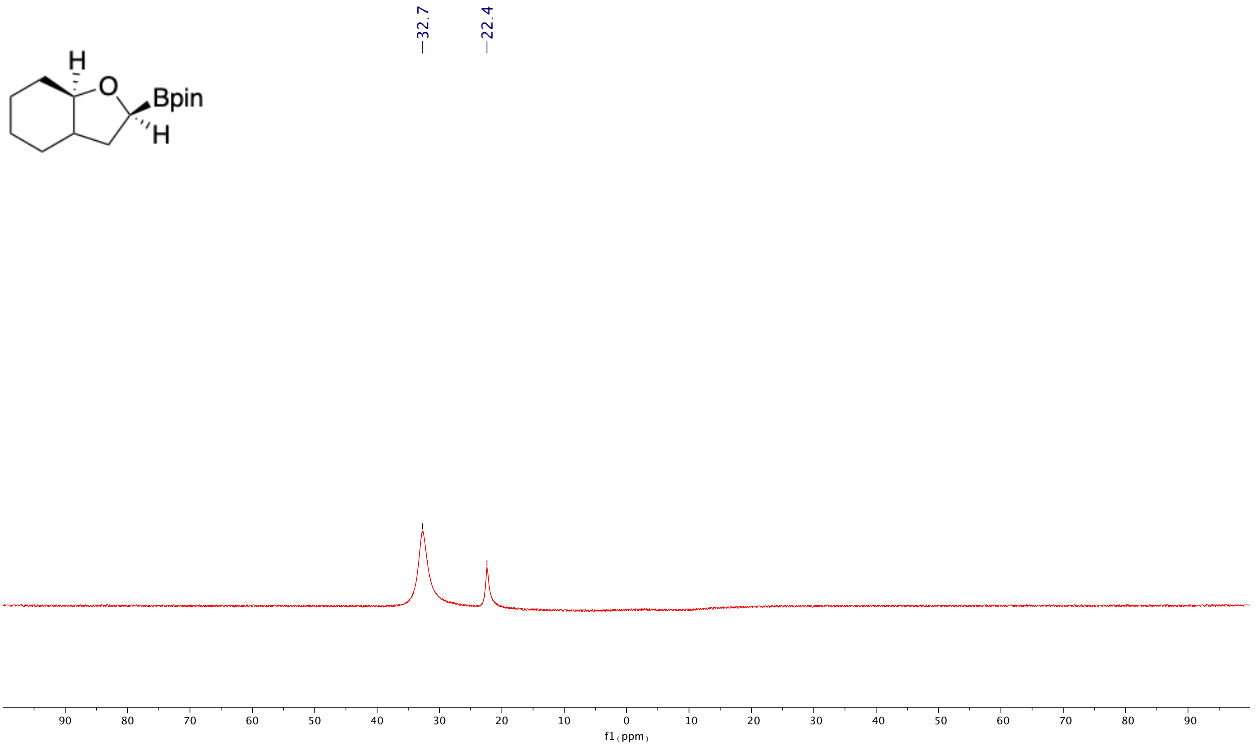

^{1}H NMR(500MHz, $CDCl_3$) of *t*-butyl 2-(4,4,5,5-tetramethyl-1,3,2-dioxaborolan-2-yl)octahydroindole-1-carboxylate (2l)

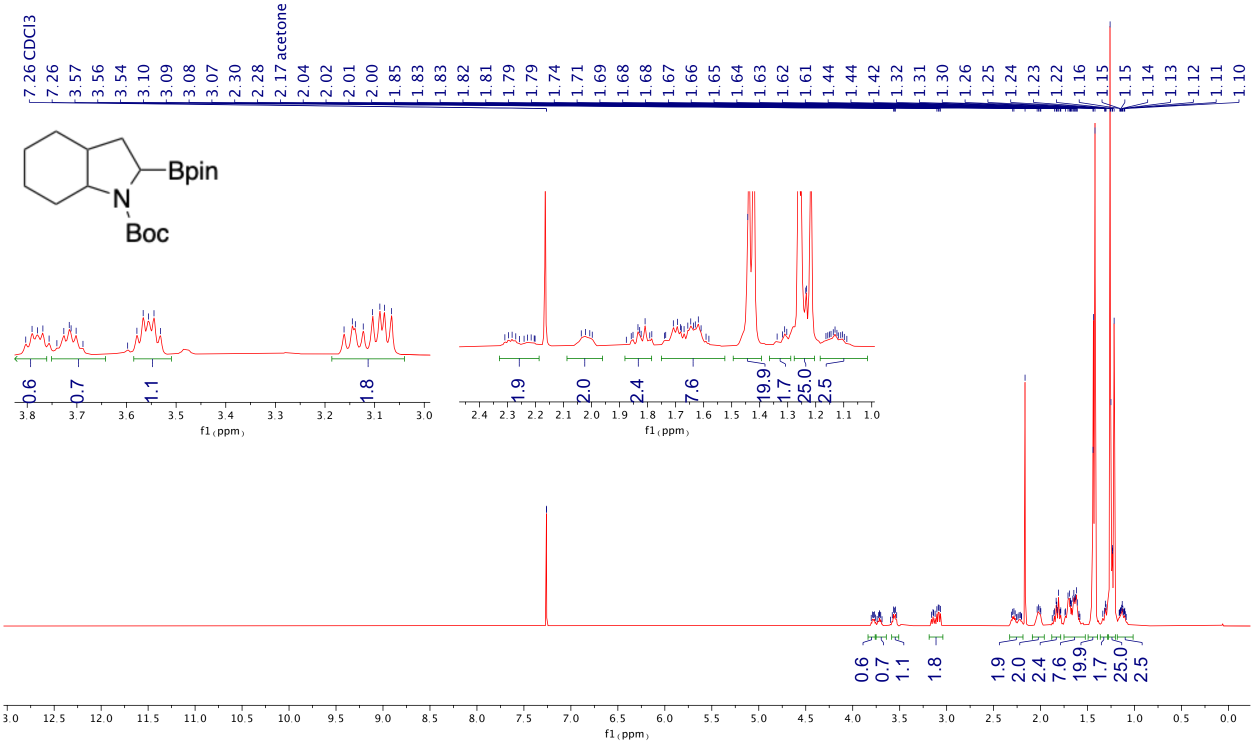

$^{13}C\{^{1}H\}$ (126 MHz, Toluene) NMR of *t*-butyl 2-(4,4,5,5-tetramethyl-1,3,2-dioxaborolan-2-yl)octahydroindole-1-carboxylate (2l)

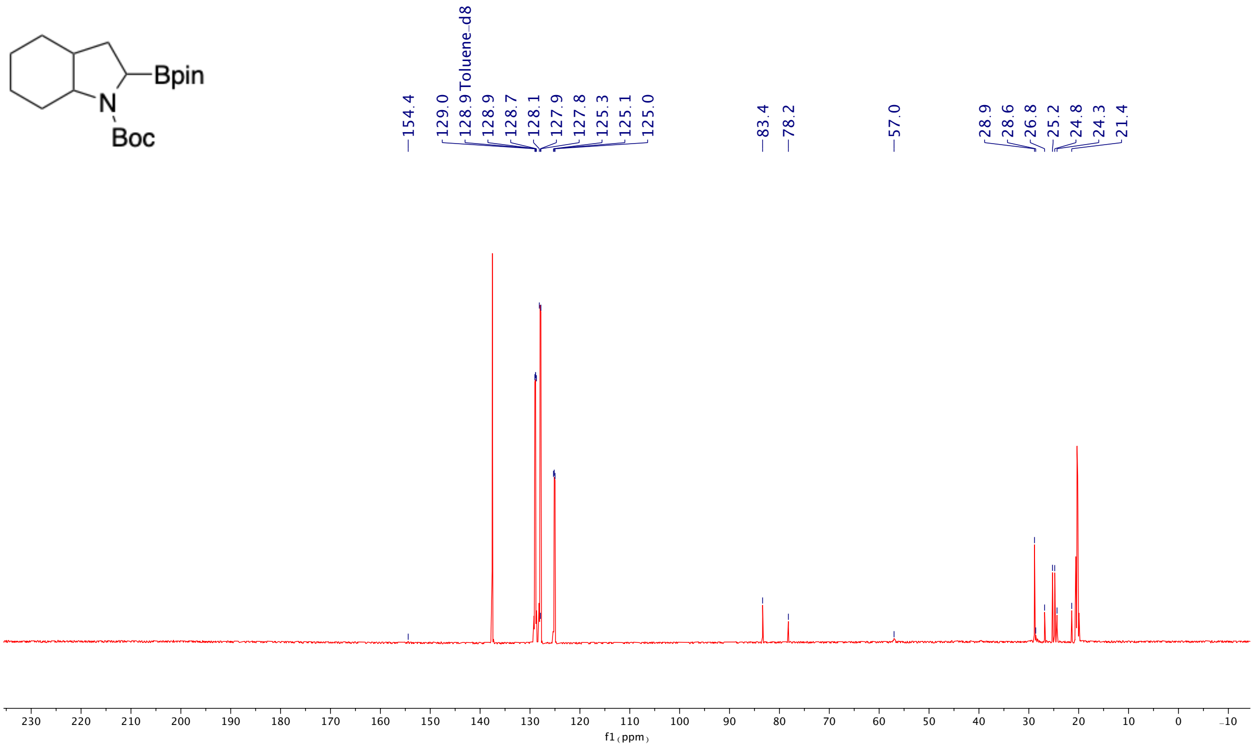

^{11}B NMR (160MHz, $CDCl_3$) of *t*-butyl 2-(4,4,5,5-tetramethyl-1,3,2-dioxaborolan-2-yl)octahydroindole-1-carboxylate (2l)

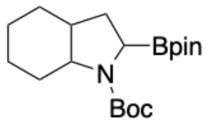

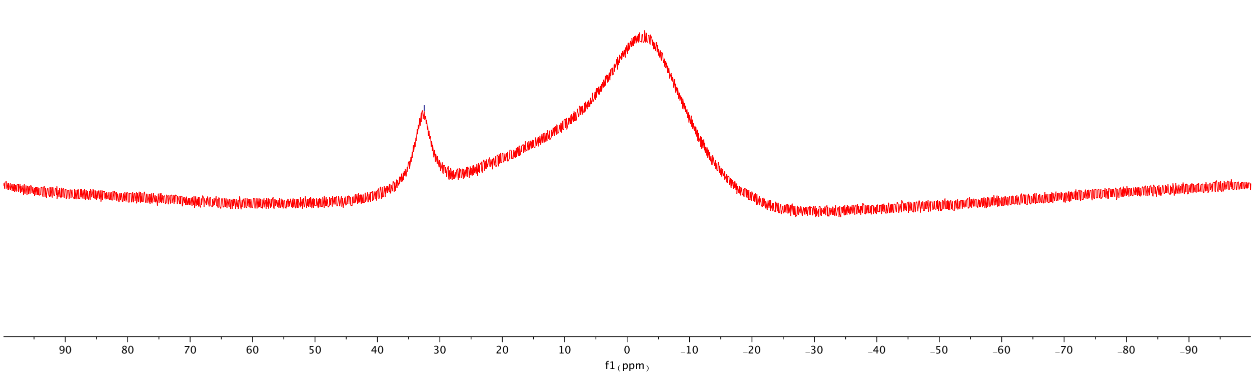

^{1}H NMR (500MHz, $CDCl_3$) of 2-(dodecahydrodibenzo[*b*,*d*]furan-4-yl)-4,4,5,5-tetramethyl-1,3,2-dioxaborolane (2k)

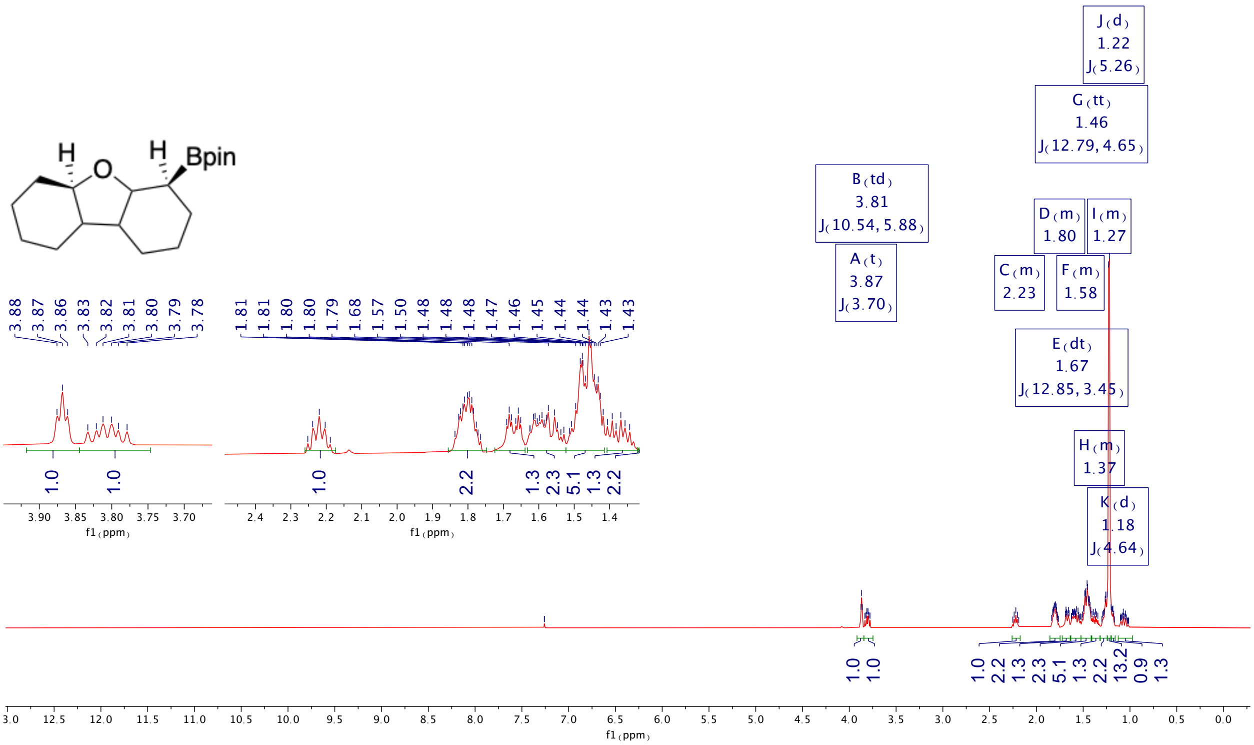

$^{13}C\{^{1}H\}$ (126 MHz, $CDCl_3$) NMR of 2-(dodecahydrodibenzo[*b*,*d*]furan-4-yl)-4,4,5,5-tetramethyl-1,3,2-dioxaborolane (2k)

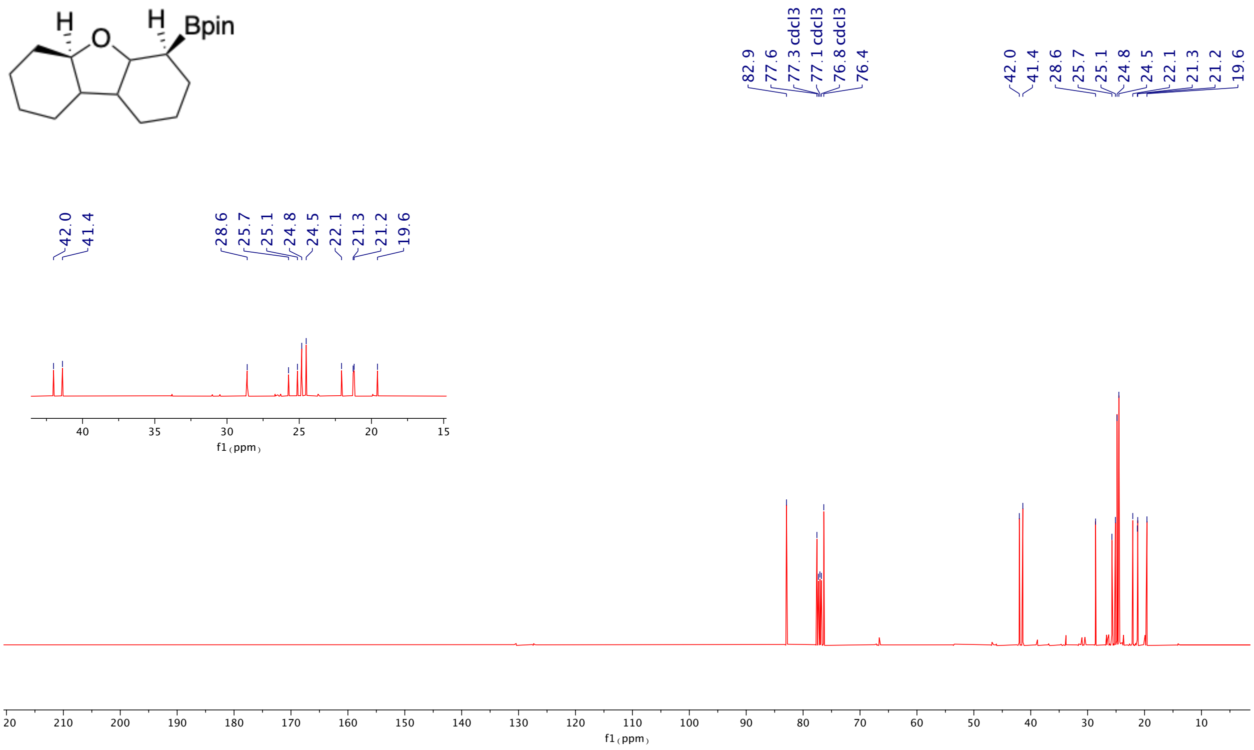

^{11}B NMR (160MHz, $CDCl_3$) of 2-(dodecahydrodibenzo[*b*,*d*]furan-4-yl)-4,4,5,5-tetramethyl-1,3,2-dioxaborolane (2k)

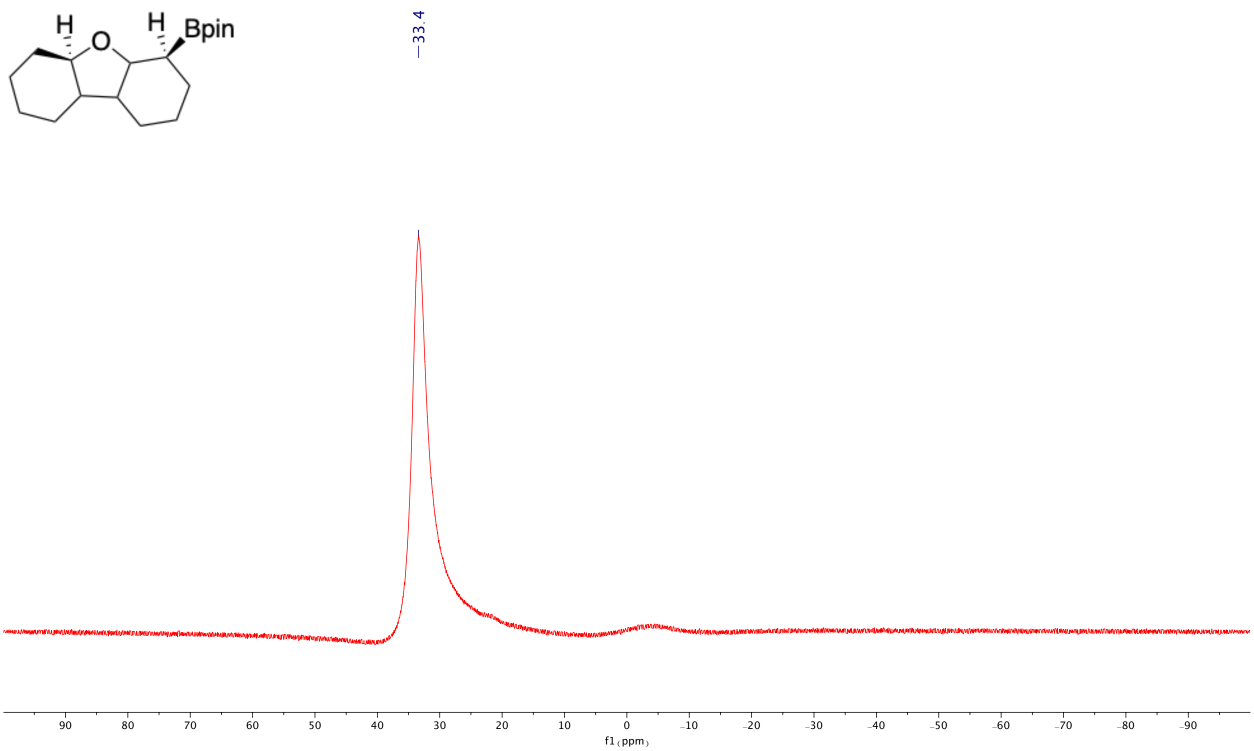

HMBC of 2-(dodecahydrodibenzo[*b*,*d*]furan-4-yl)-4,4,5,5-tetramethyl-1,3,2-dioxaborolane (2k)

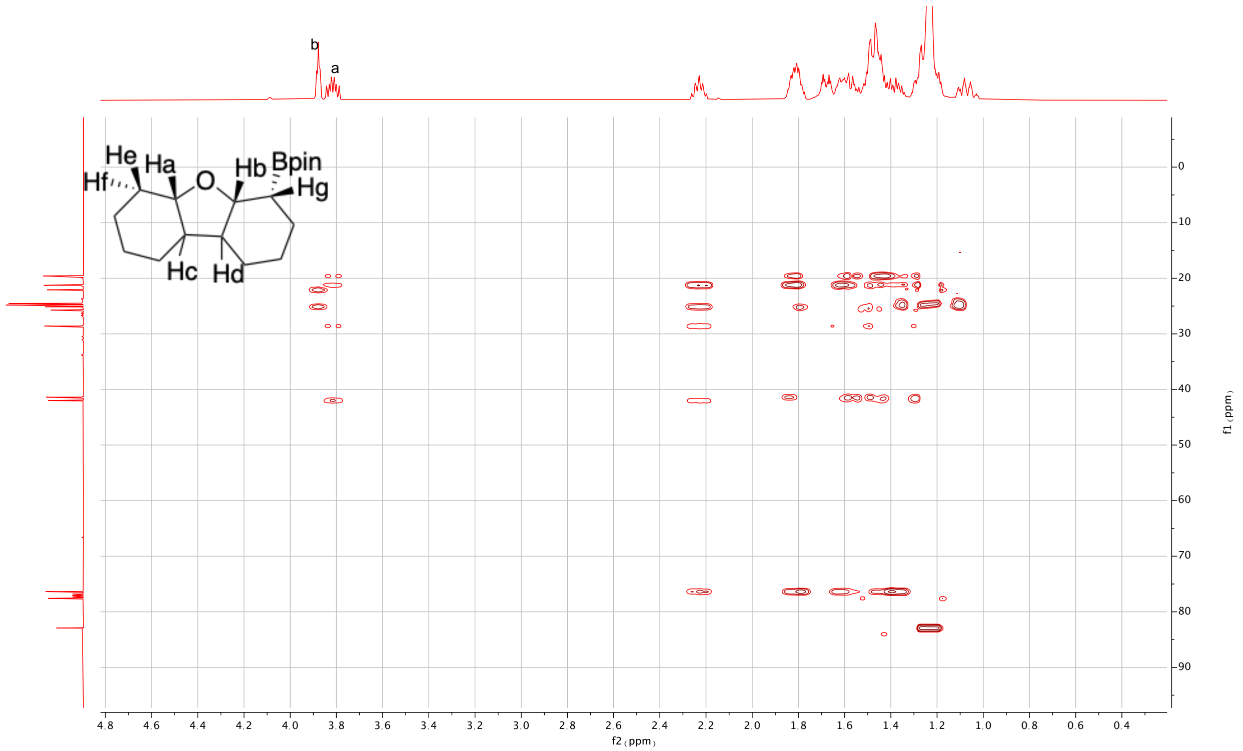

HSQC of 2-(dodecahydrodibenzo[*b*,*d*]furan-4-yl)-4,4,5,5-tetramethyl-1,3,2-dioxaborolane (2k)

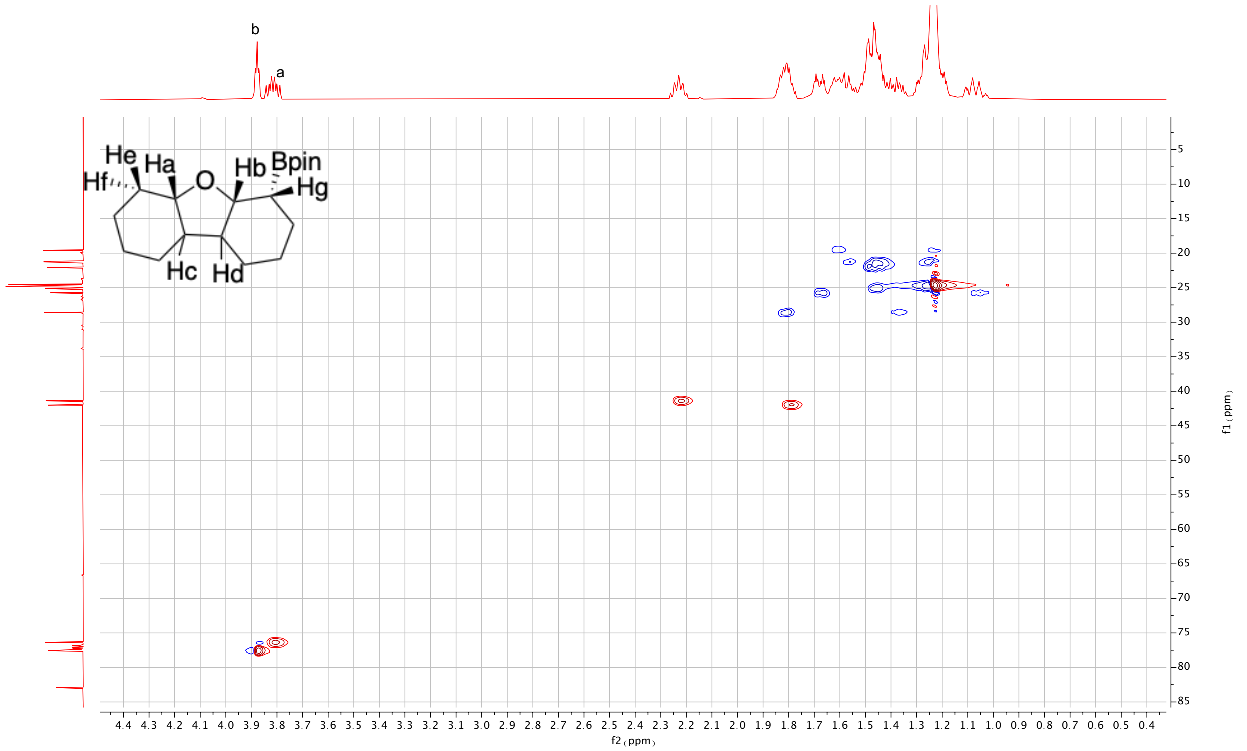

NOE of 2-(dodecahydrodibenzo[*b*,*d*]furan-4-yl)-4,4,5,5-tetramethyl-1,3,2-dioxaborolane (2k)

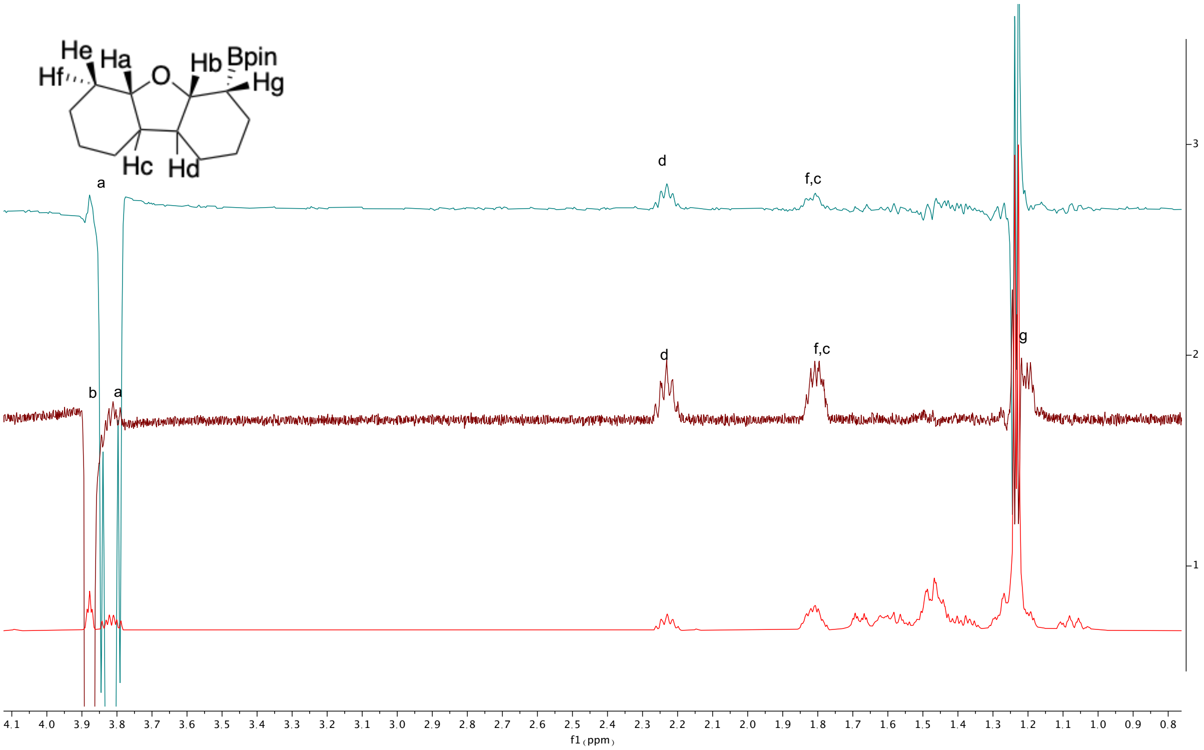

^{1}H NMR (500MHz, $CDCl_3$) of 2-cyclohexyl-4,4,5,5-tetramethyl-1,3,2-dioxaborolane (2p)

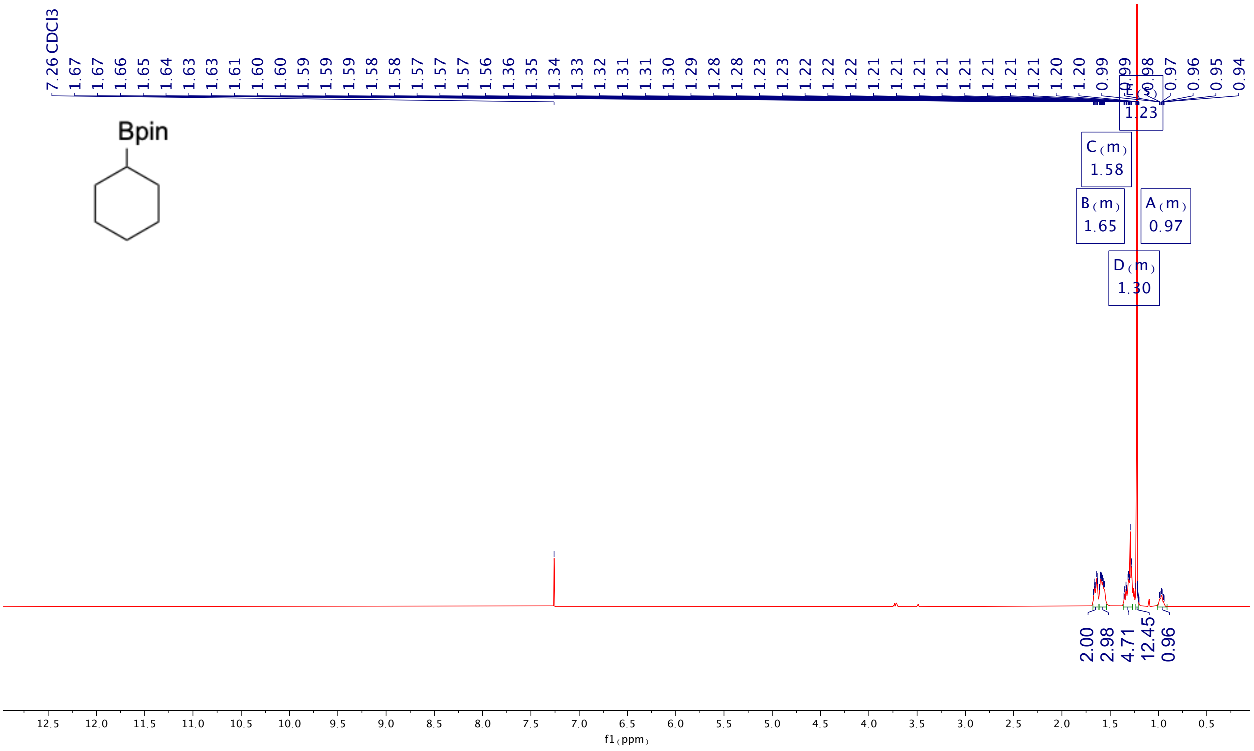

$^{13}C\{^{1}H\}$ (126 MHz, $CDCl_3$) NMR of 2-cyclohexyl-4,4,5,5-tetramethyl-1,3,2-dioxaborolane (2p)

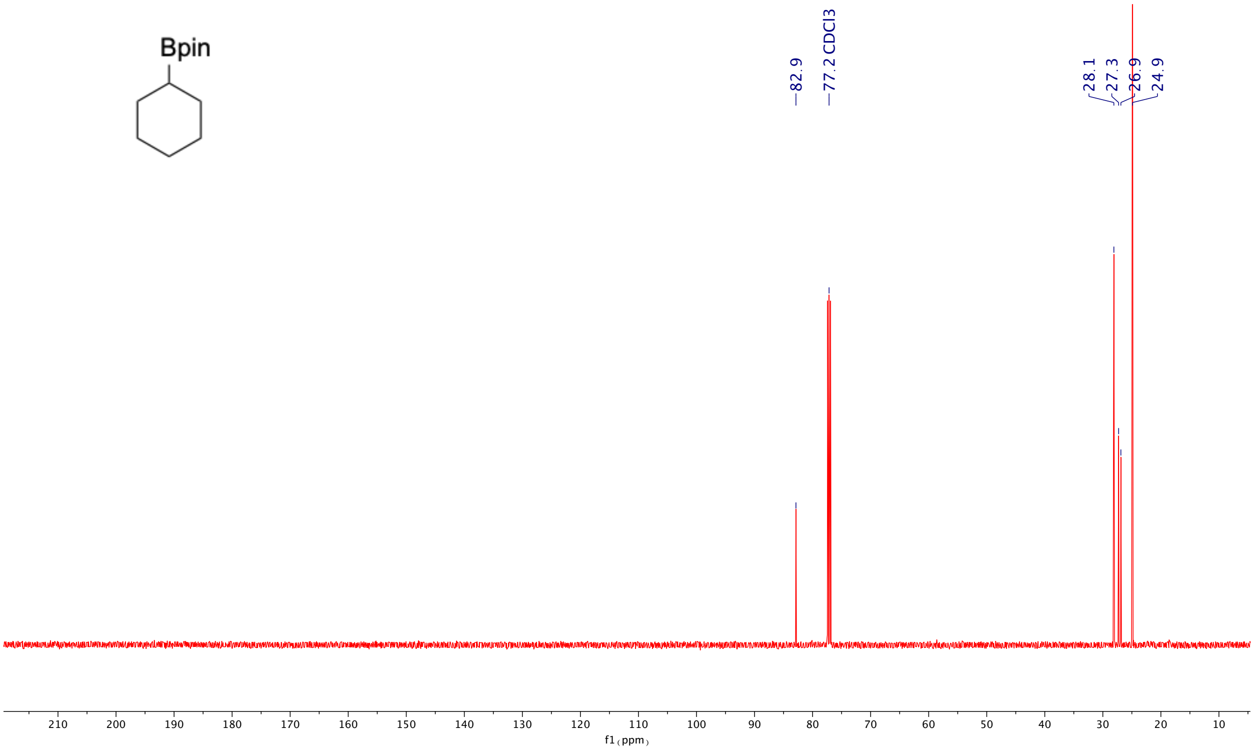

^{11}B NMR (160MHz, $CDCl_3$) of 2-cyclohexyl-4,4,5,5-tetramethyl-1,3,2-dioxaborolane (2p)

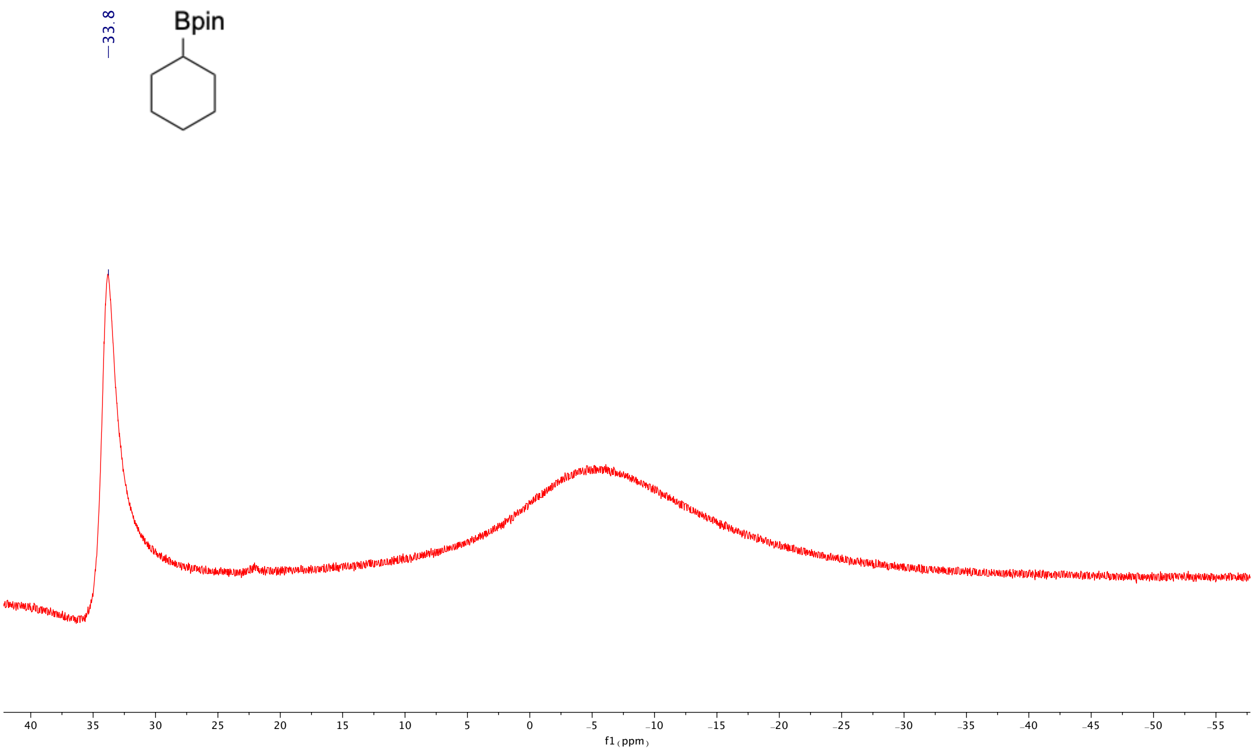

^{1}H NMR (500MHz, $CDCl_3$) of 3-(4,4,5,5-tetramethyl-1,3,2-dioxaborolan-2-yl)cyclohexan-1-ol (2q)

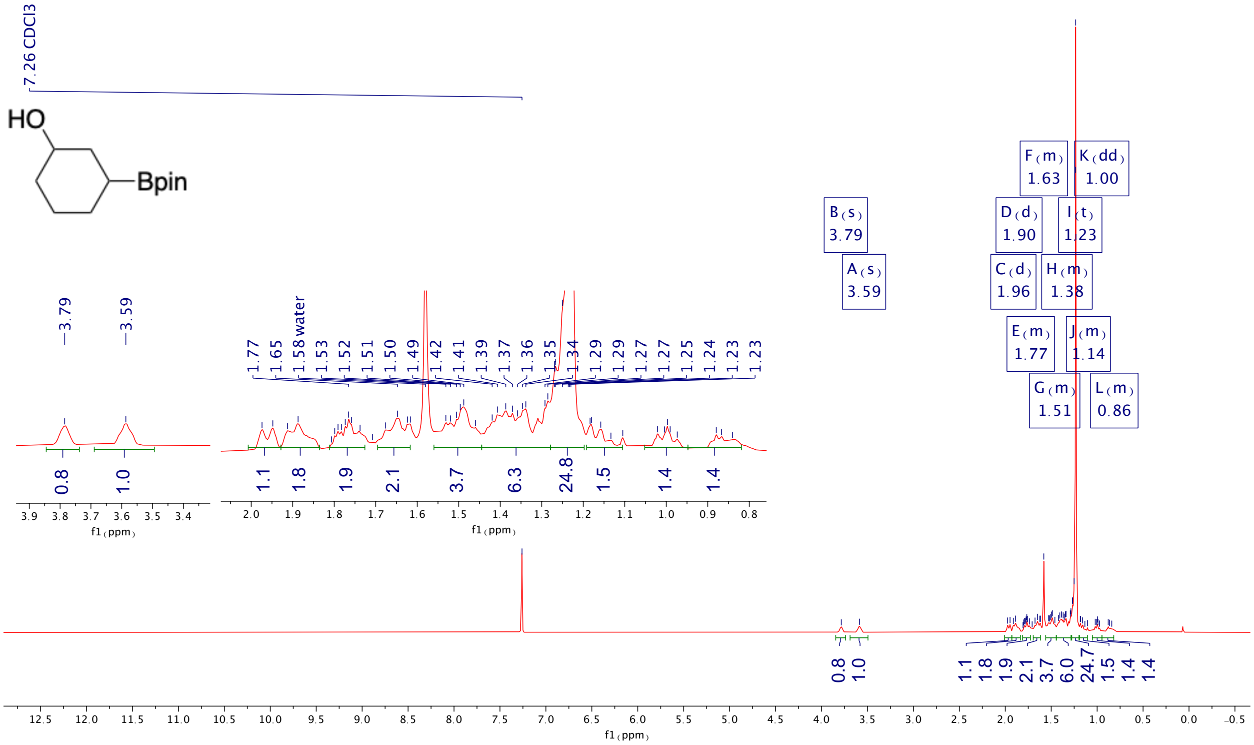

$^{13}C\{^{1}H\}$ (126 MHz, $CDCl_3$) NMR of 3-(4,4,5,5-tetramethyl-1,3,2-dioxaborolan-2-yl)cyclohexan-1-ol (2q)

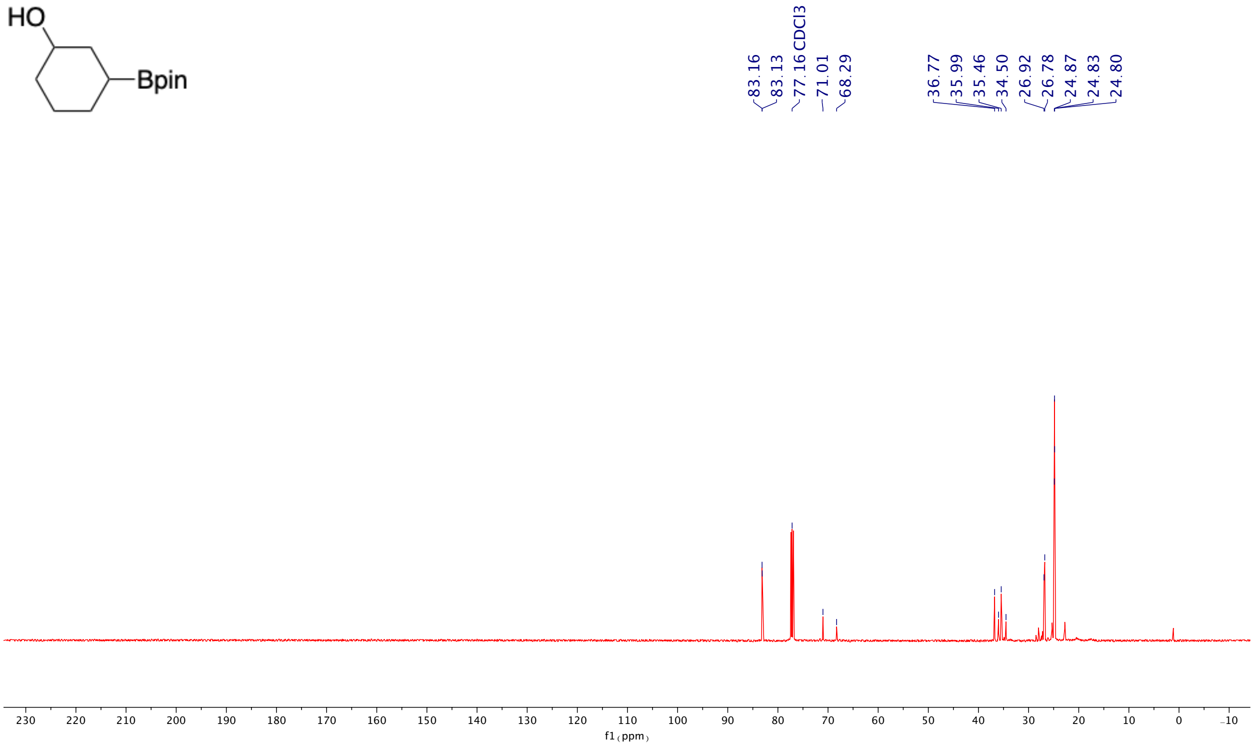

^{11}B NMR (160MHz, $CDCl_3$) of 3-(4,4,5,5-tetramethyl-1,3,2-dioxaborolan-2-yl)cyclohexan-1-ol (2q)

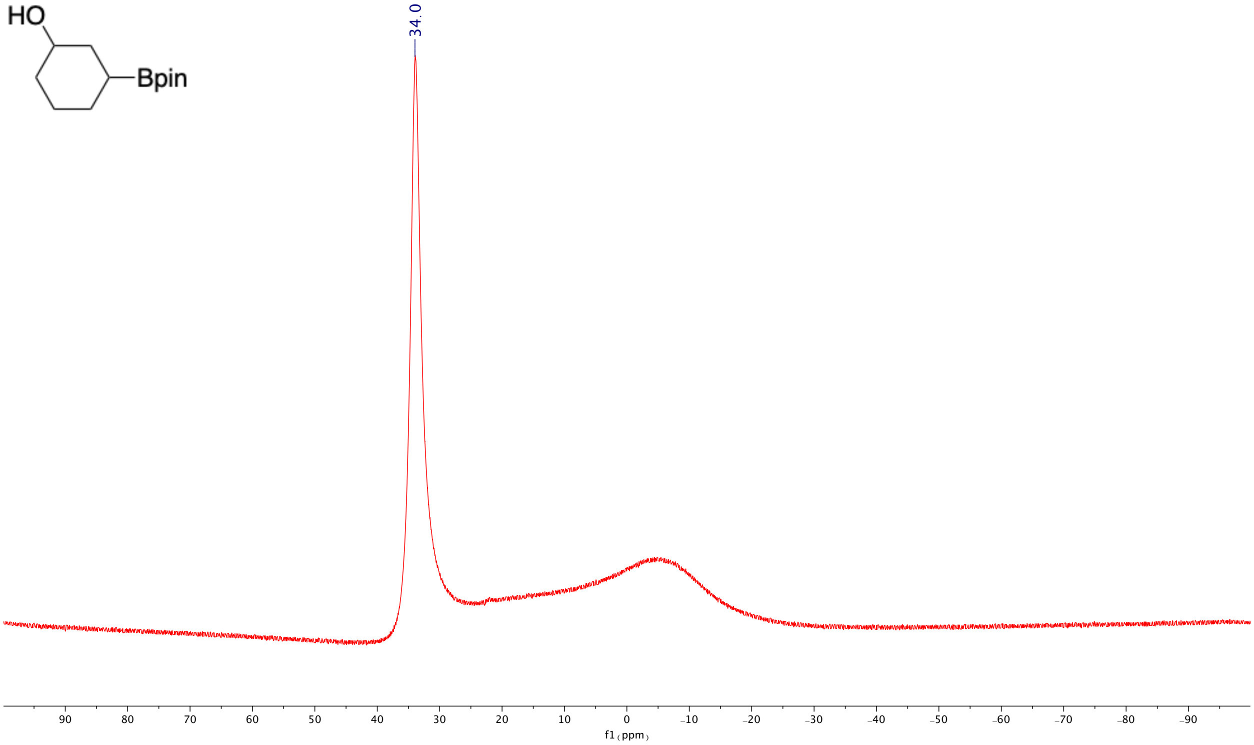

^1H NMR (500MHz, $CDCl_3$) of 4,4,5,5-tetramethyl-2-(-3-(trifluoromethyl)cyclohexyl)-1,3,2-dioxaborolane (2r)

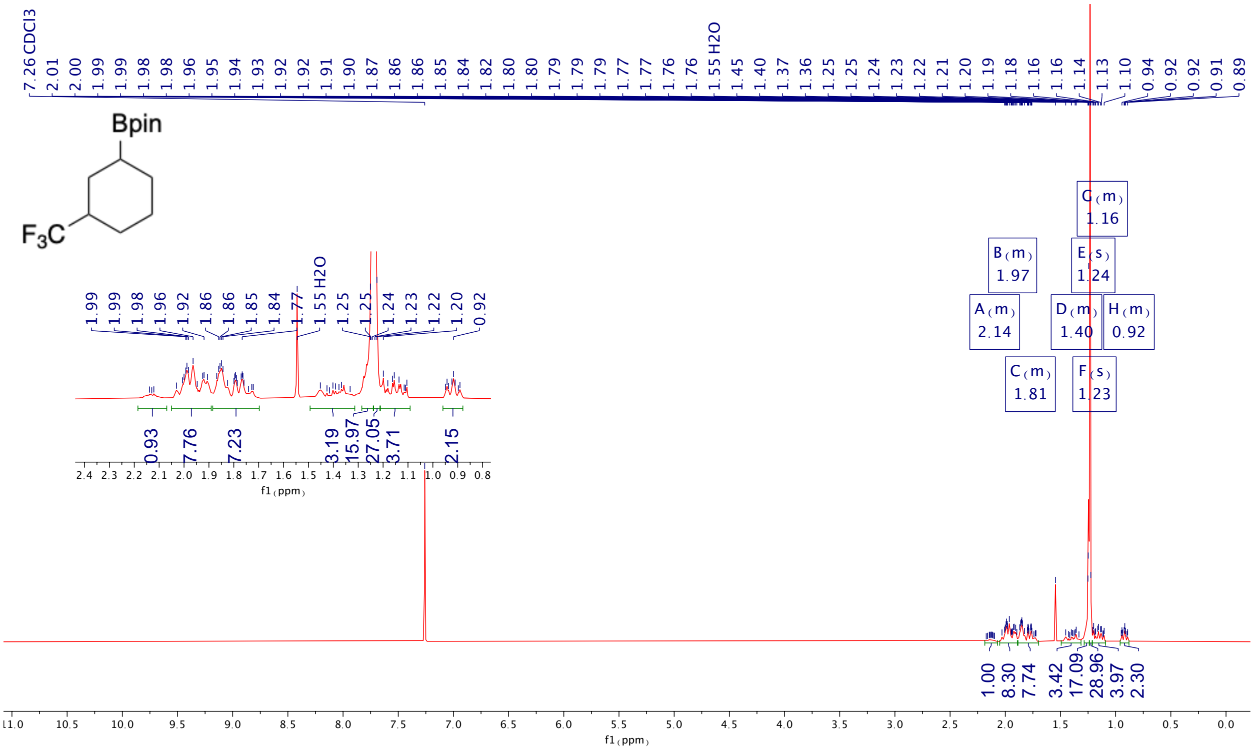

$^{13}C\{^1H\}$ (126 MHz, $CDCl_3$) NMR of 4,4,5,5-tetramethyl-2-(-3-(trifluoromethyl)cyclohexyl)-1,3,2-dioxaborolane (2r)

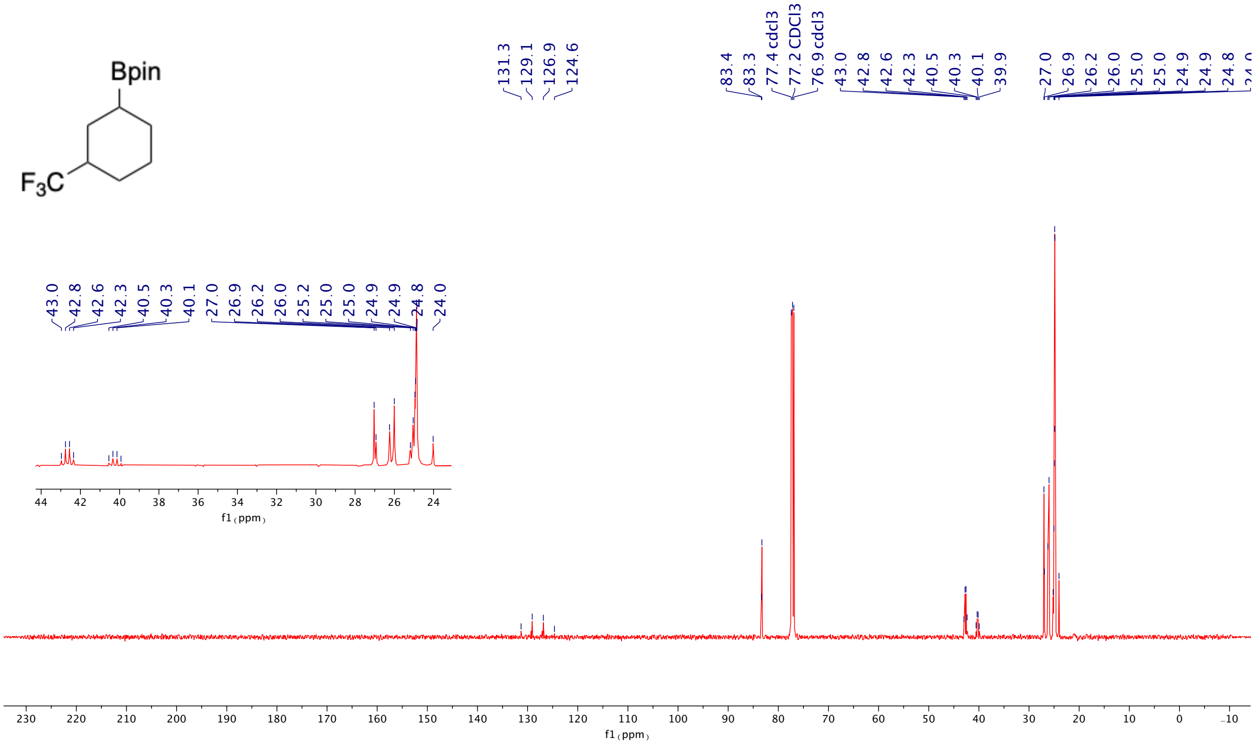

^{11}B NMR (160MHz, $CDCl_3$) of 4,4,5,5-tetramethyl-2-(-3-(trifluoromethyl)cyclohexyl)-1,3,2-dioxaborolane (2r)

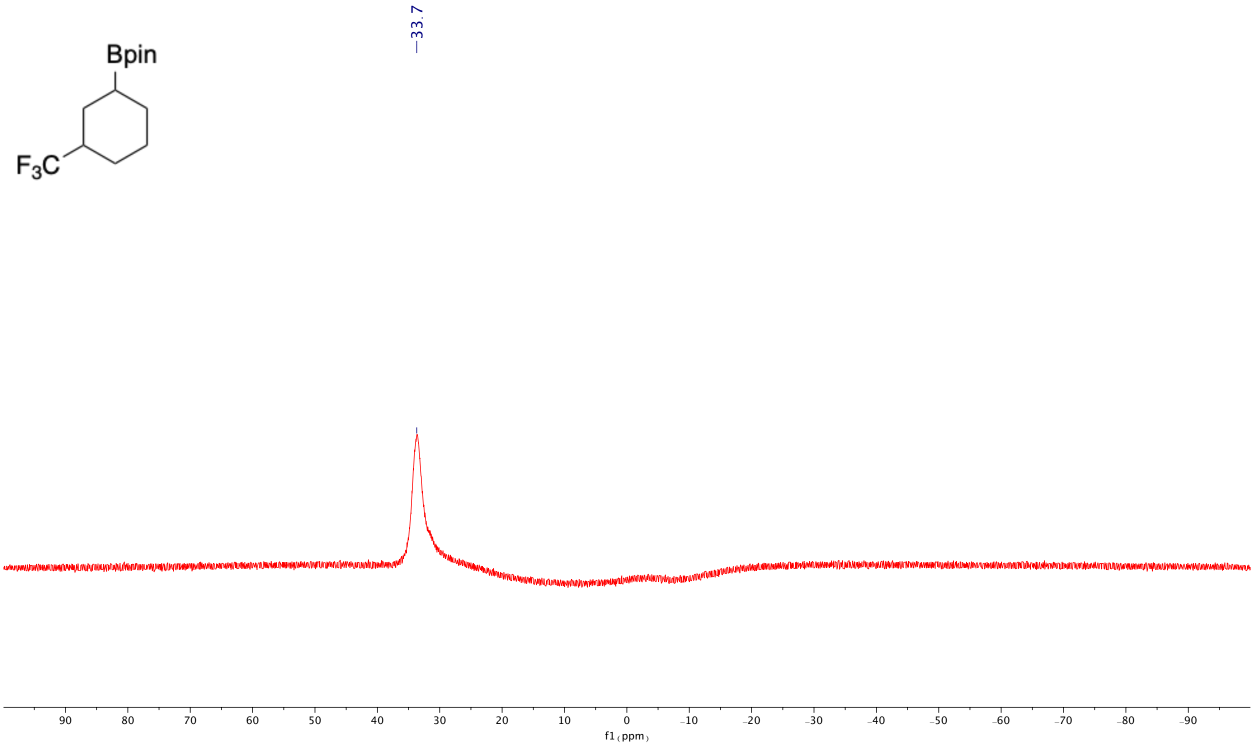

^{19}F NMR (470 MHz, $CDCl_3$) of 4,4,5,5-tetramethyl-2-(-3-(trifluoromethyl)cyclohexyl)-1,3,2-dioxaborolane (2r)

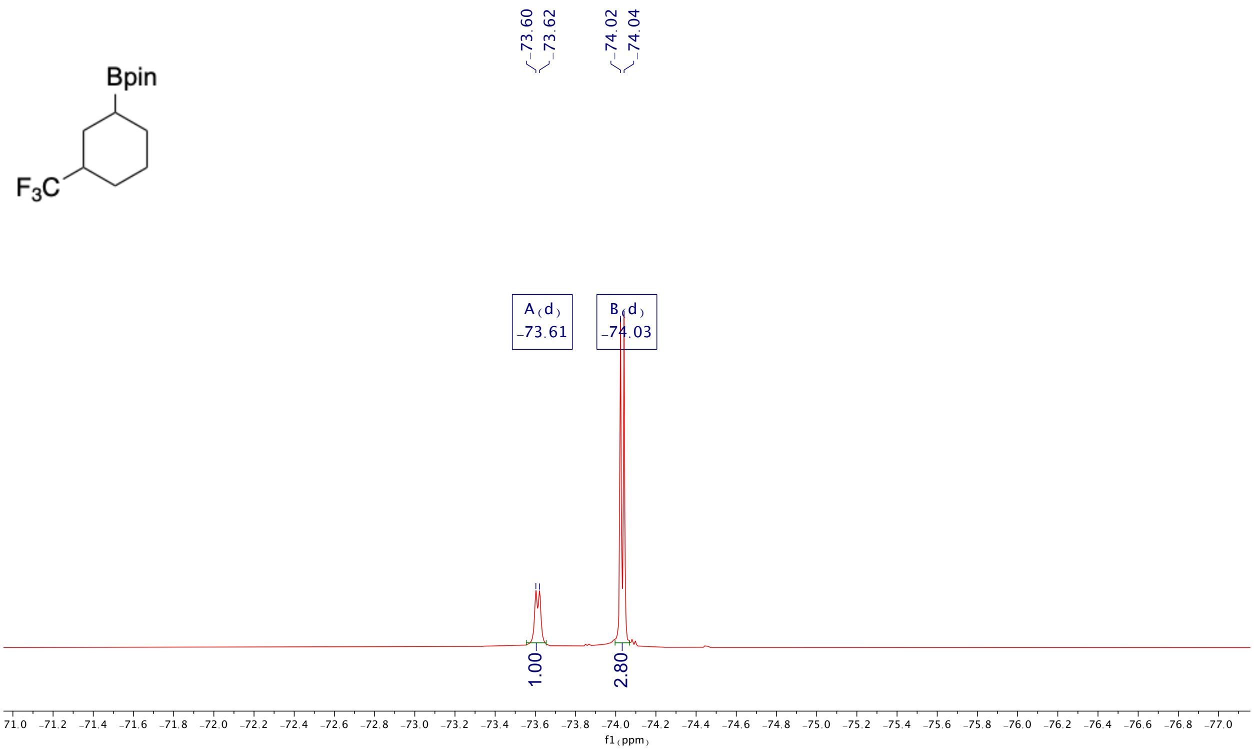

^{1}H NMR (500MHz, $CDCl_3$) of ethyl-3-(4,4,5,5-tetramethyl-1,3,2-dioxaborolan-2-yl)cyclohexane-1-carboxylate (2s)

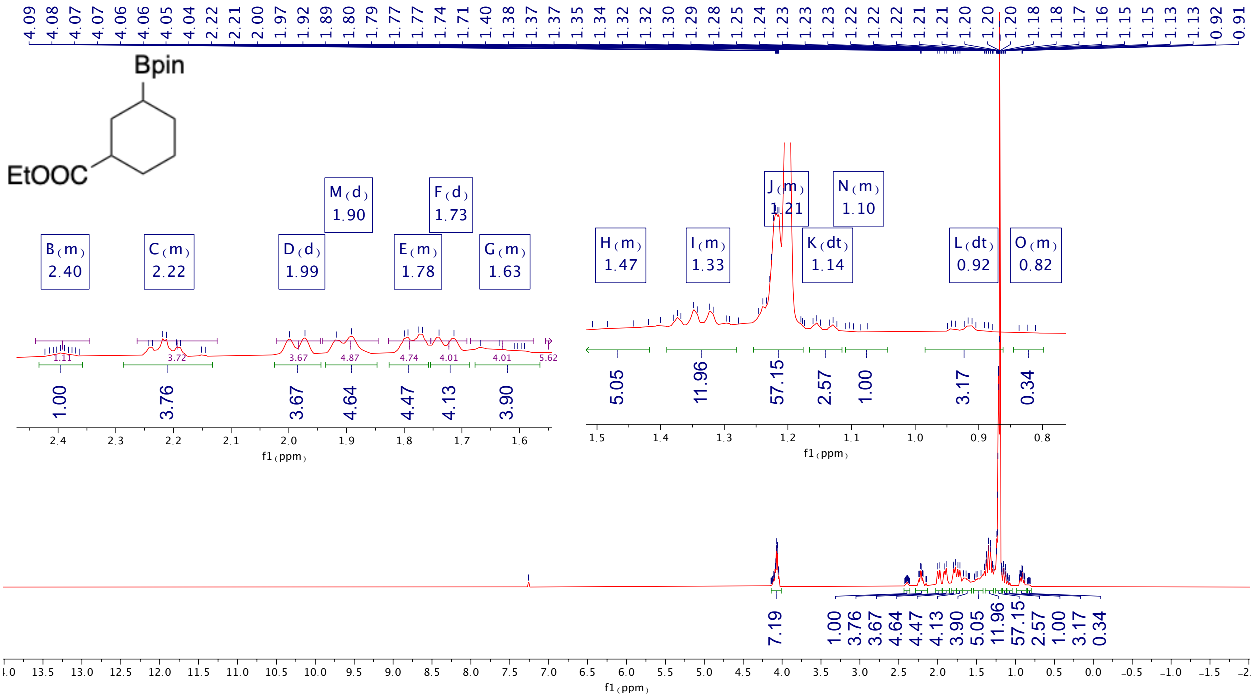

$^{13}C\{^{1}H\}$ (126 MHz, $CDCl_3$) NMR of ethyl-3-(4,4,5,5-tetramethyl-1,3,2-dioxaborolan-2-yl)cyclohexane-1-carboxylate (2s)

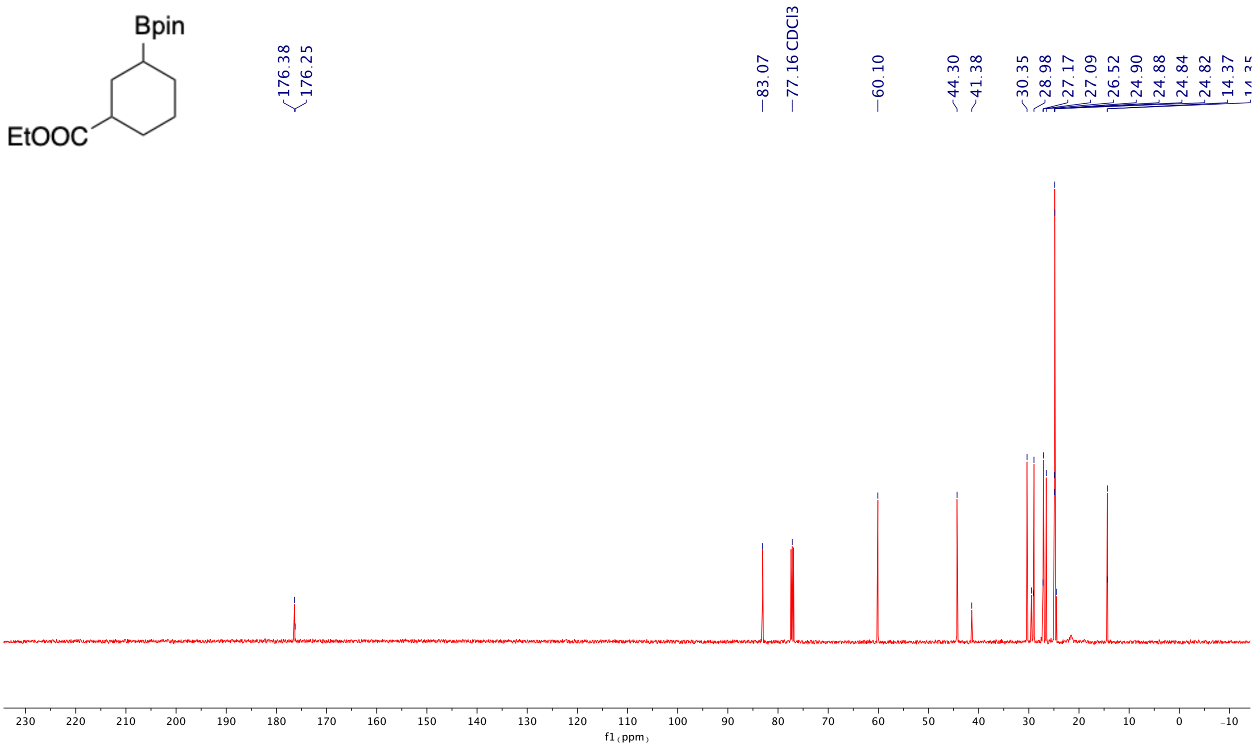

^{11}B NMR (160MHz, $CDCl_3$) of ethyl-3-(4,4,5,5-tetramethyl-1,3,2-dioxaborolan-2-yl)cyclohexane-1-carboxylate (2s)

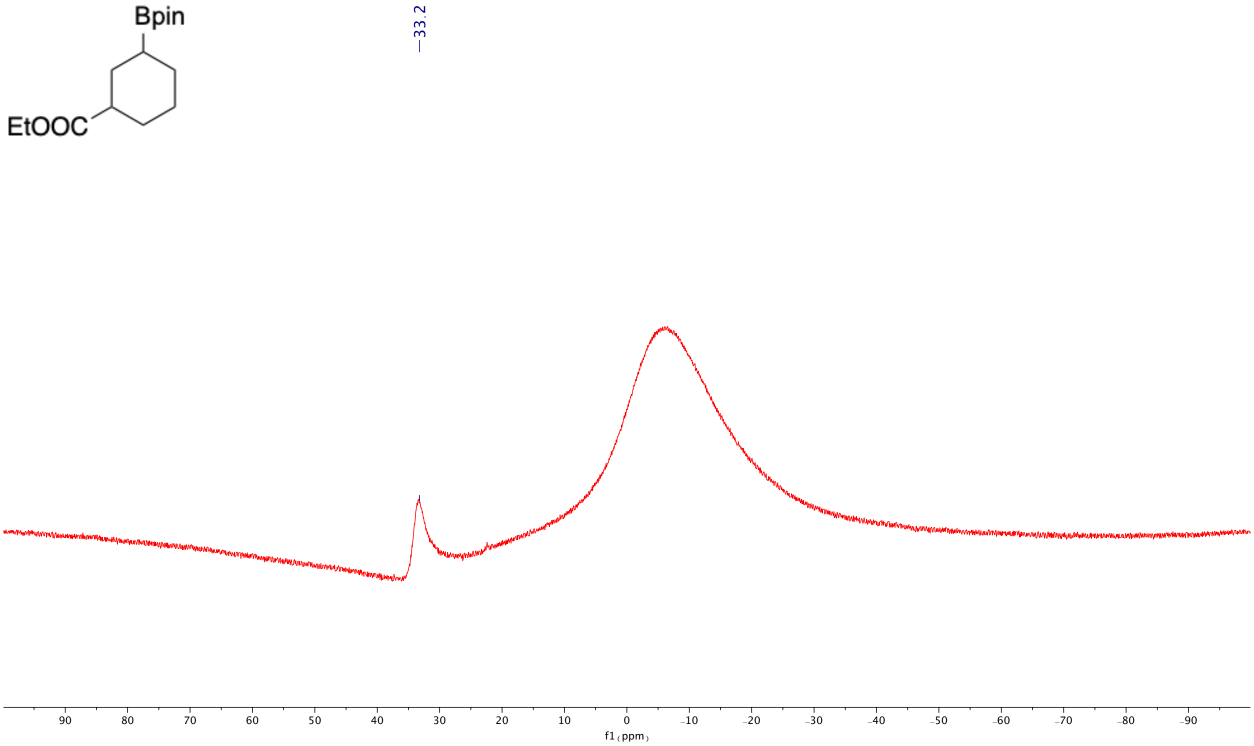

HSQC of ethyl-3-(4,4,5,5-tetramethyl-1,3,2-dioxaborolan-2-yl)cyclohexane-1-carboxylate (2s)

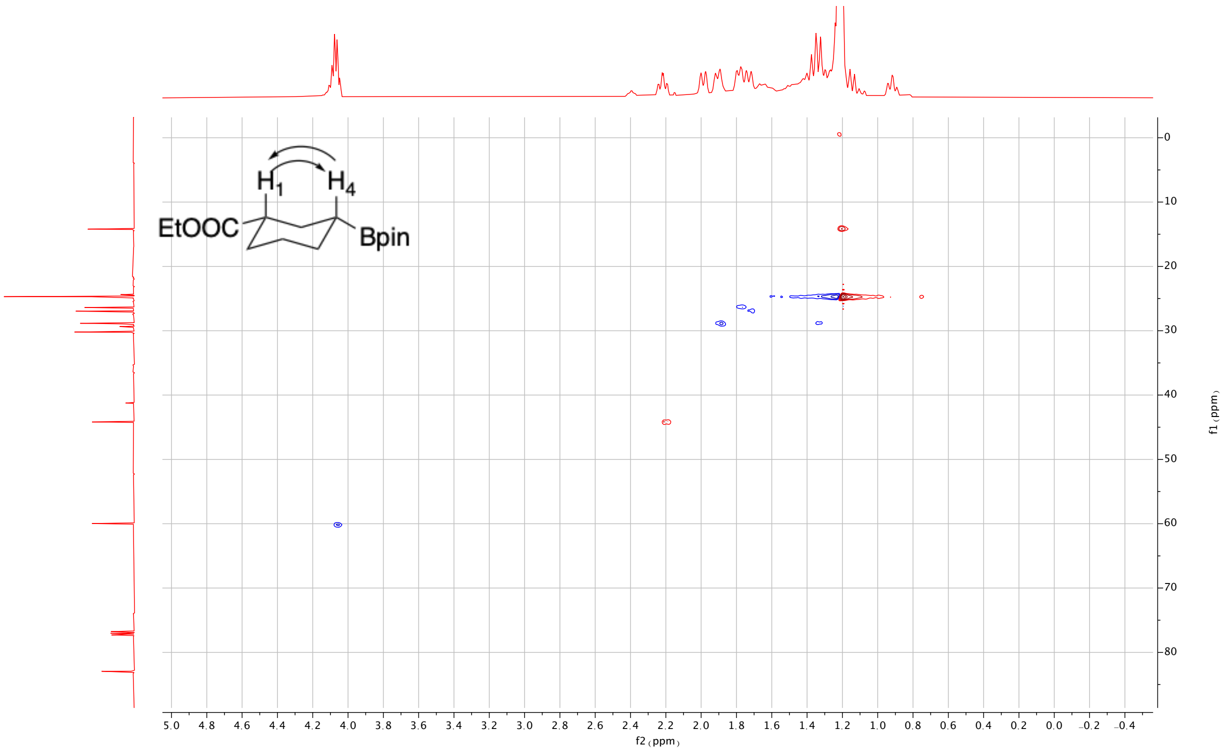

NOE of ethyl-3-(4,4,5,5-tetramethyl-1,3,2-dioxaborolan-2-yl)cyclohexane-1-carboxylate (2s)

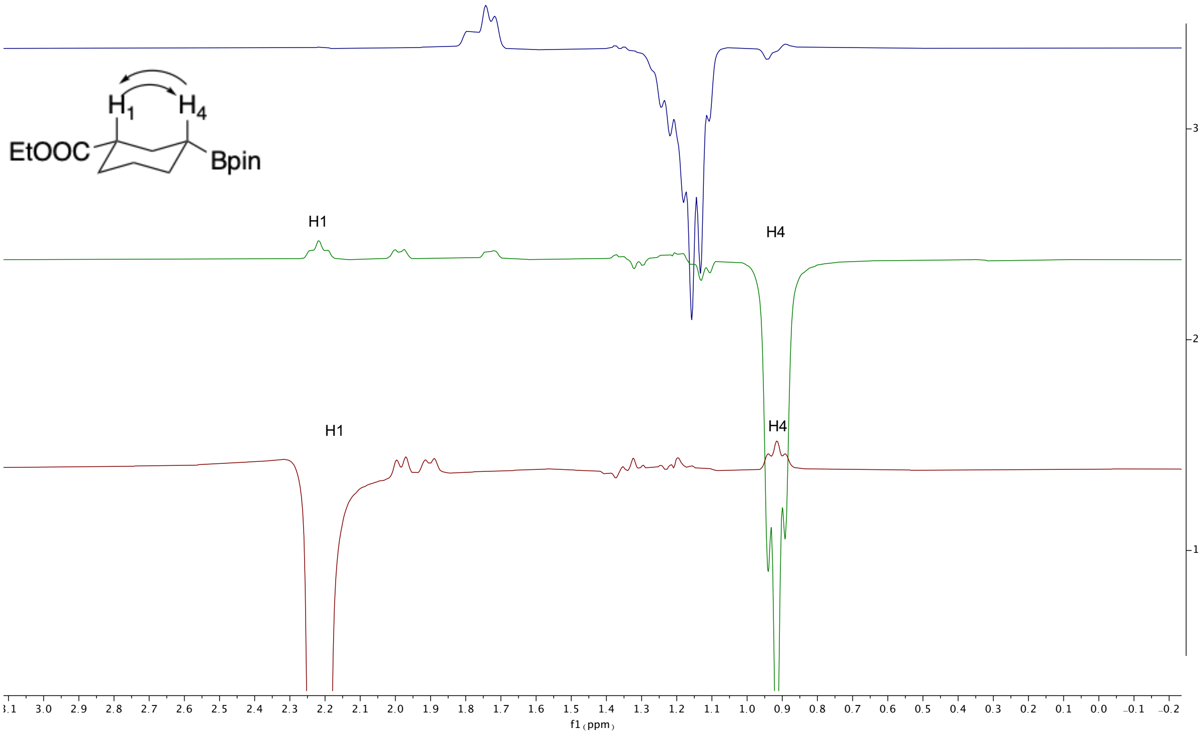

^{1}H NMR (500MHz, $CDCl_3$) of 4,4,5,5-tetramethyl-2-(3-methylcyclohexyl)-1,3,2-dioxaborolane (2t)

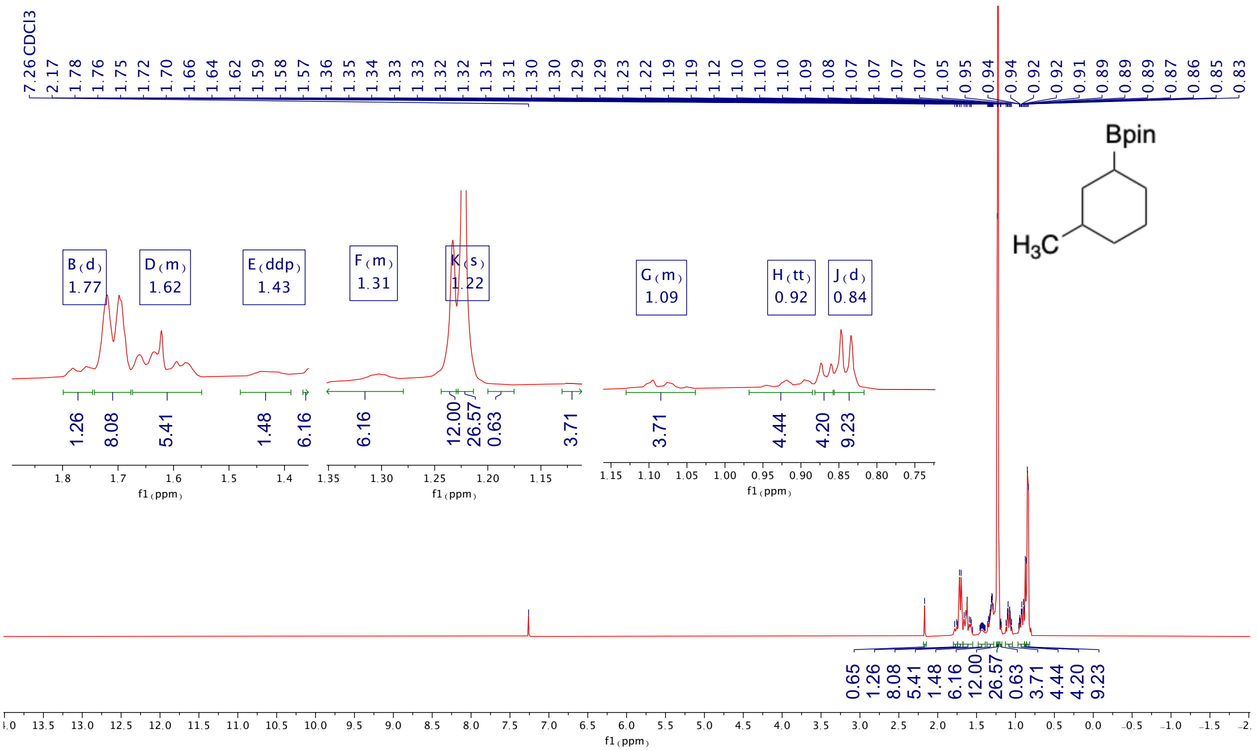

$^{13}C\{^{1}H\}$ (126 MHz, $CDCl_3$) NMR of 4,4,5,5-tetramethyl-2-(3-methylcyclohexyl)-1,3,2-dioxaborolane (2t)

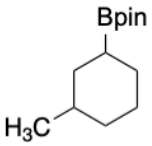

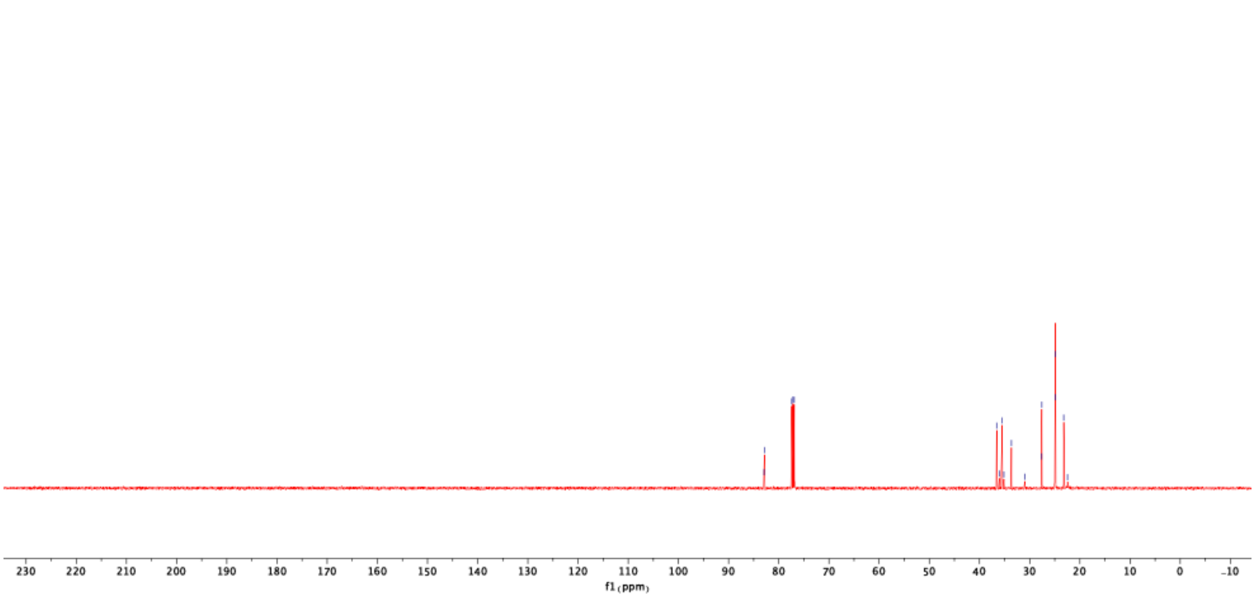

^{11}B NMR (160MHz, $CDCl_3$) of 4,4,5,5-tetramethyl-2-(3-methylcyclohexyl)-1,3,2-dioxaborolane (2t)

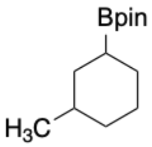

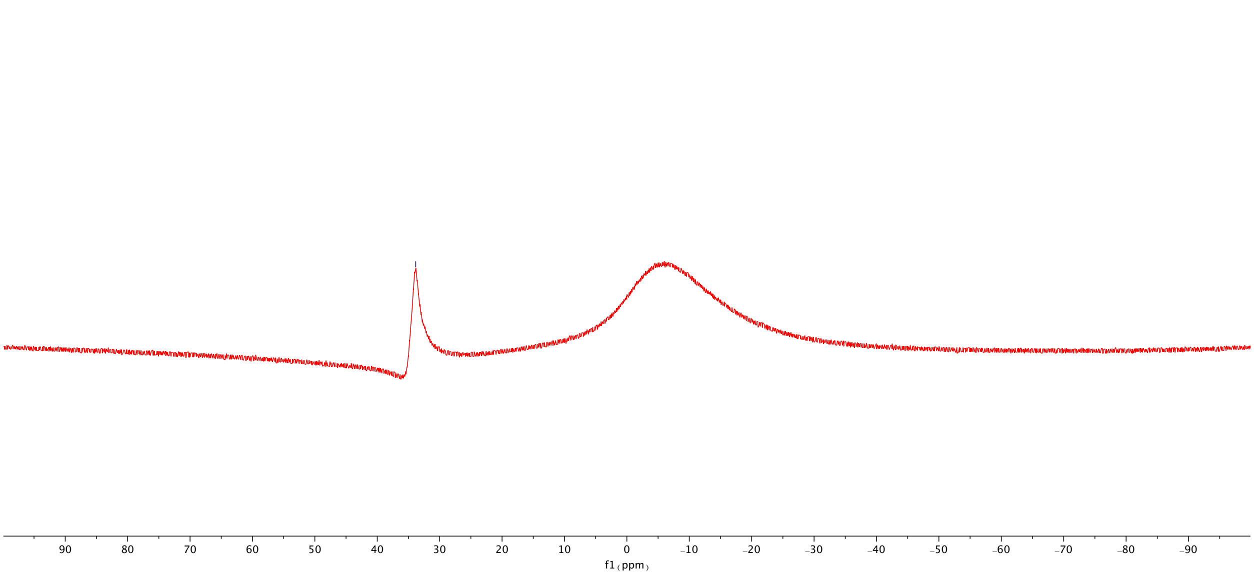

^{1}H NMR (500MHz, $CDCl_3$) of 2-(-3-methoxycyclohexyl)-4,4,5,5-tetramethyl-1,3,2-dioxaborolane (2u)

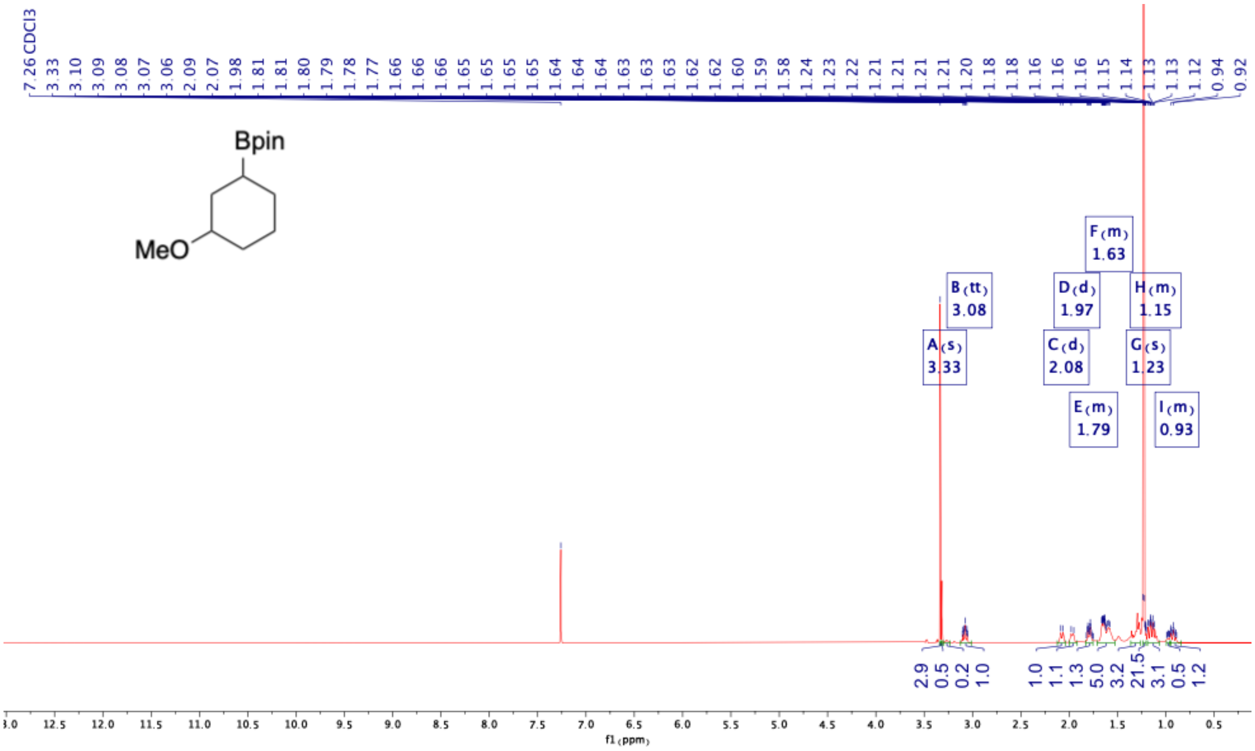

$^{13}C\{^{1}H\}$ (126 MHz, $CDCl_3$) NMR of 2-(-3-methoxycyclohexyl)-4,4,5,5-tetramethyl-1,3,2-dioxaborolane (2u)

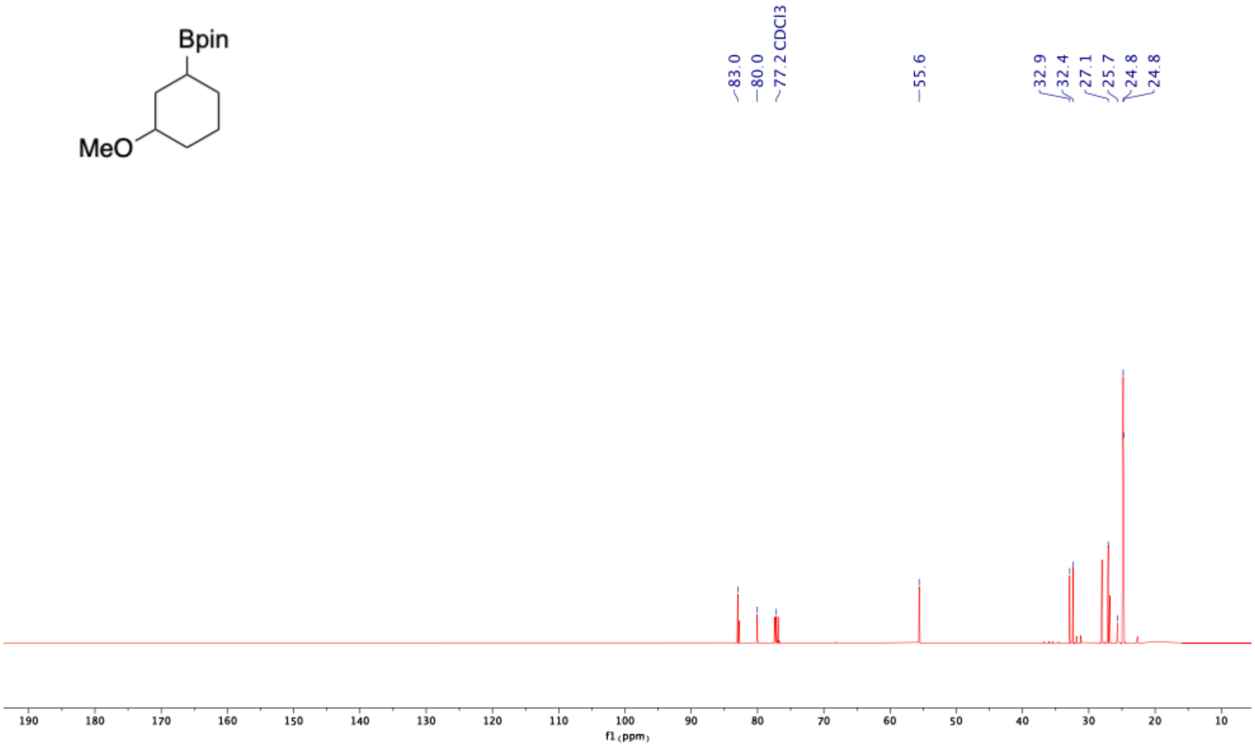

^{11}B NMR (160MHz, $CDCl_3$) of 2-(-3-methoxycyclohexyl)-4,4,5,5-tetramethyl-1,3,2-dioxaborolane (2u)

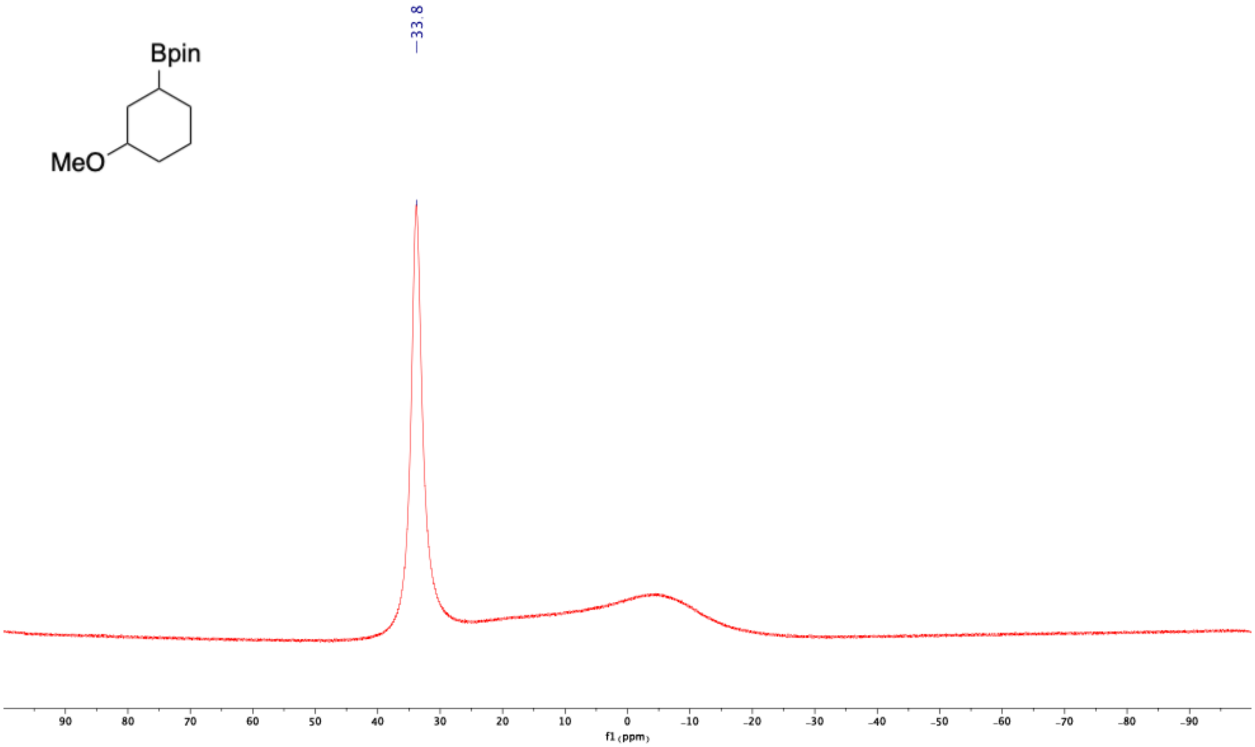

^{1}H NMR (500MHz, $CDCl_3$) of cis-1,2-bis(4,4,5,5-tetramethyl-1,3,2-dioxaborolan-2-yl)cyclohexane (2v)

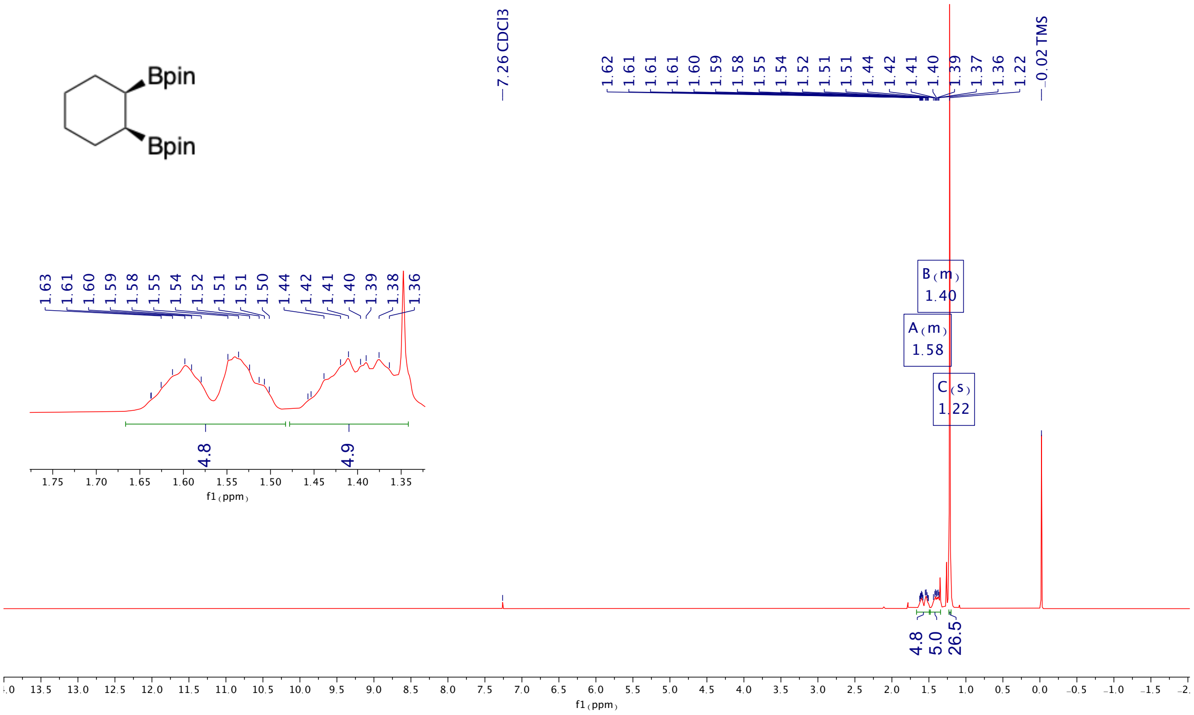

$^{13}C\{^{1}H\}$ (126 MHz, $CDCl_3$) of cis-1,2-bis(4,4,5,5-tetraethyl-1,3,2-dioxaborolan-2-yl)cyclohexane (2v)

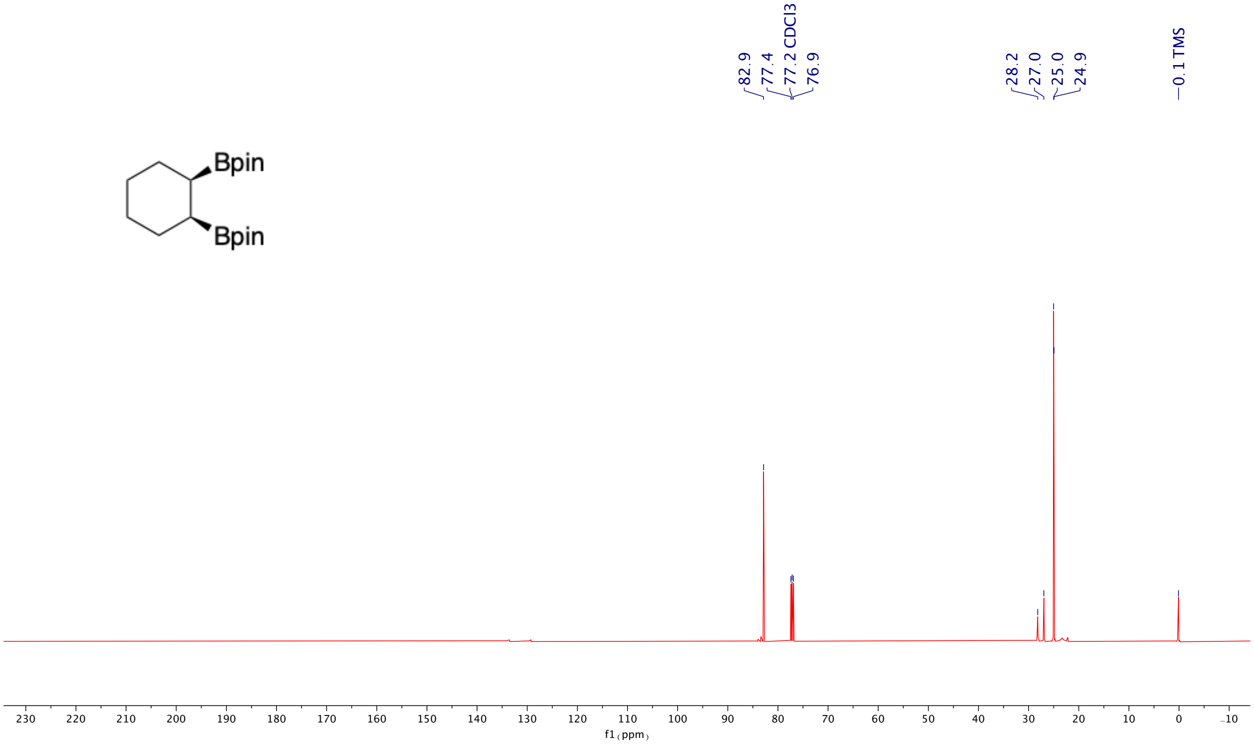

^{11}B NMR (160MHz, $CDCl_3$) of cis-1,2-bis(4,4,5,5-tetraethyl-1,3,2-dioxaborolan-2-yl)cyclohexane (2v)

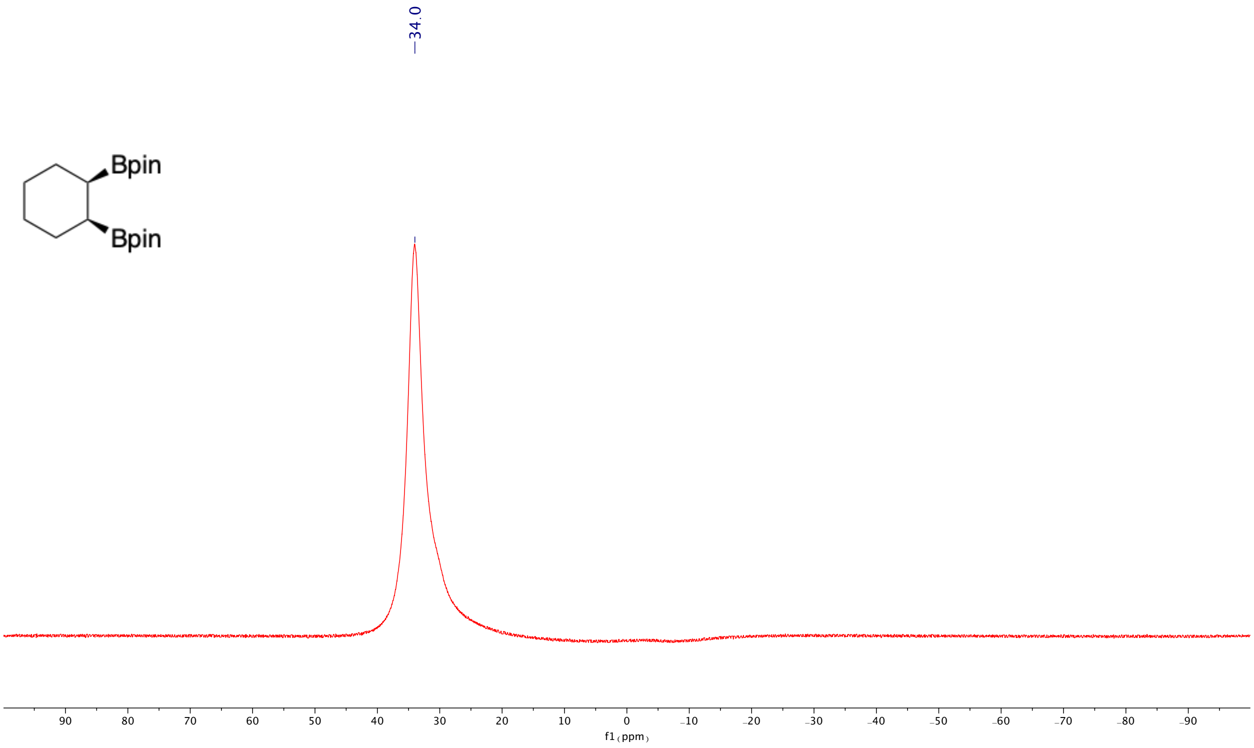

^{1}H NMR (500MHz, D_2O) of 2-(trimethylsilyl)piperidin-1-ium (+)-camphorsulfonate (2w)

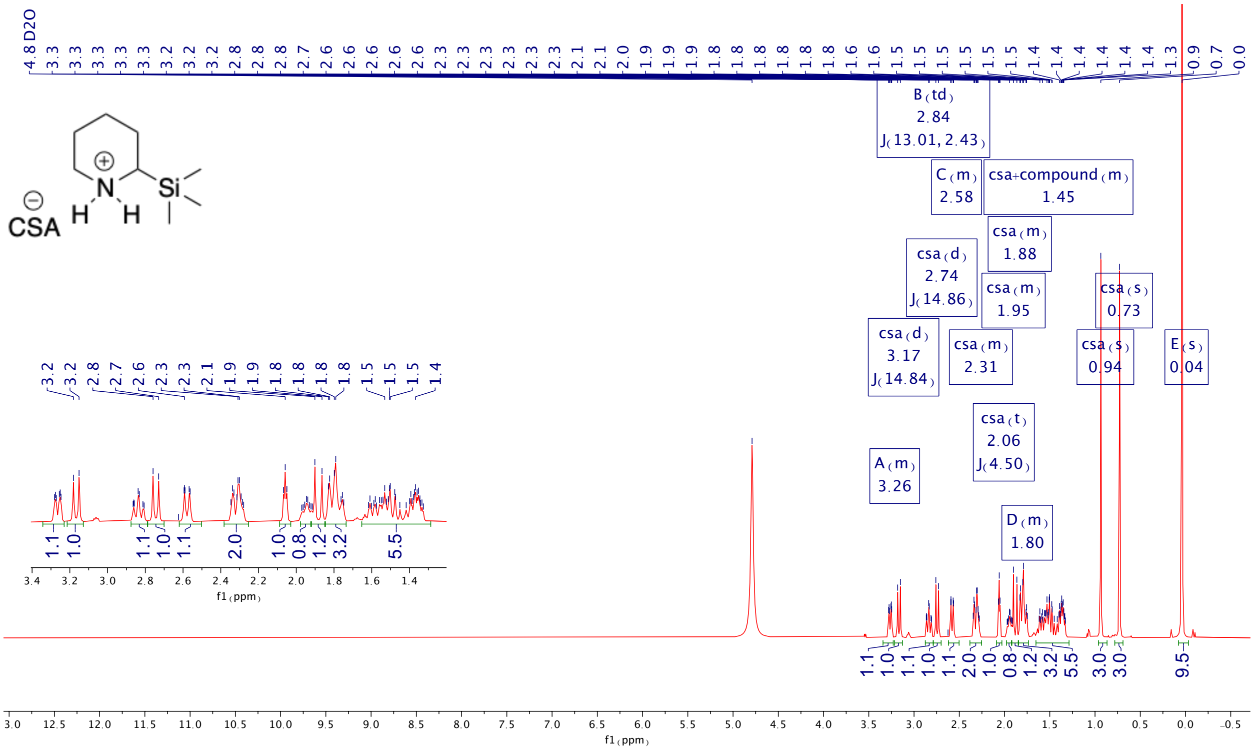

$^{13}C\{^{1}H\}$ (126 MHz, D_2O) NMR of 2-(trimethylsilyl)piperidin-1-ium (+)-camphorsulfonate (2w)

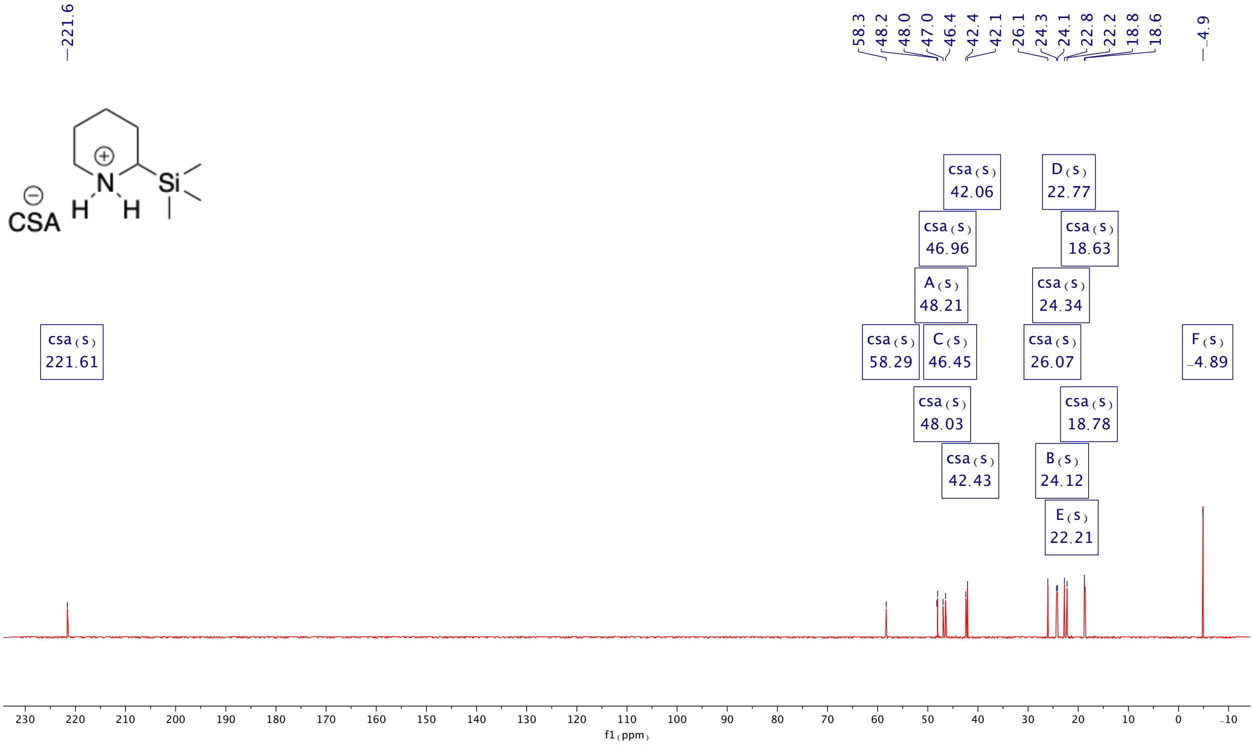

^{29}Si NMR (99MHz, D_2O) of 2-(trimethylsilyl)piperidin-1-ium (+)-camphorsulfonate (2w)

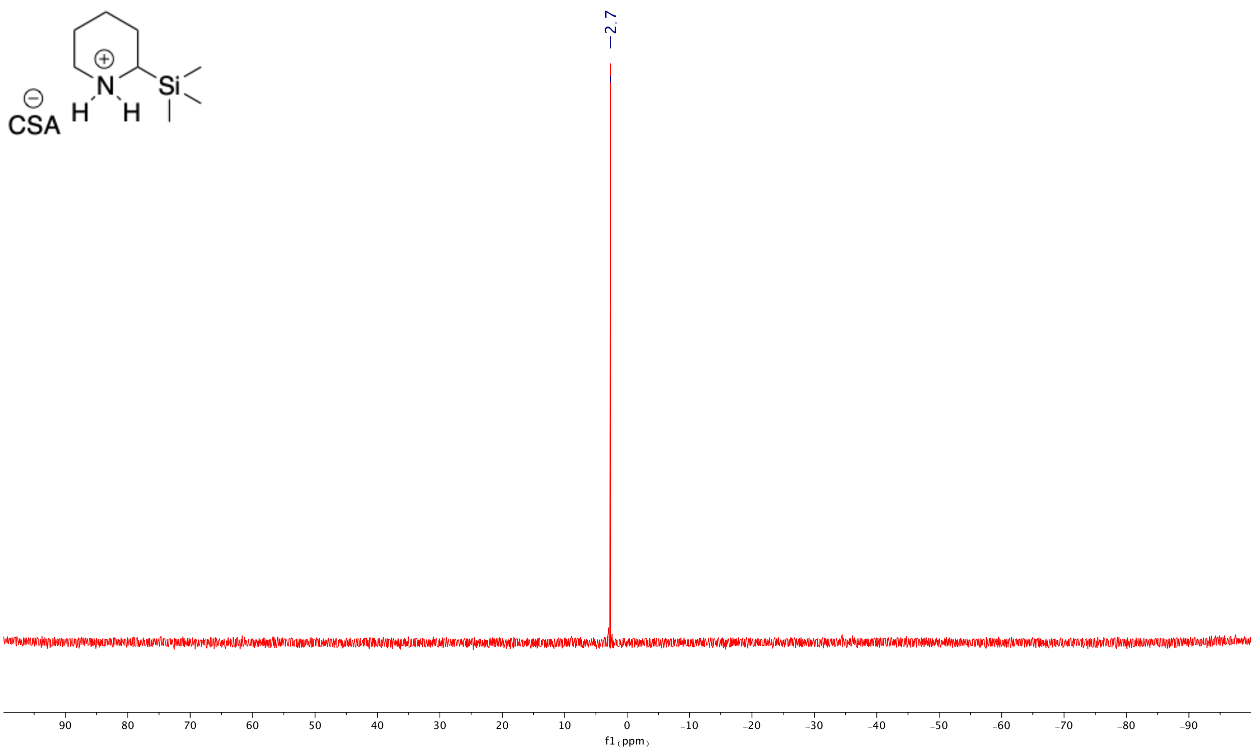

^{1}H NMR (500MHz, D_2O) of 2-(Trifluoromethyl)-4-(trimethylsilyl)piperidine (2x)

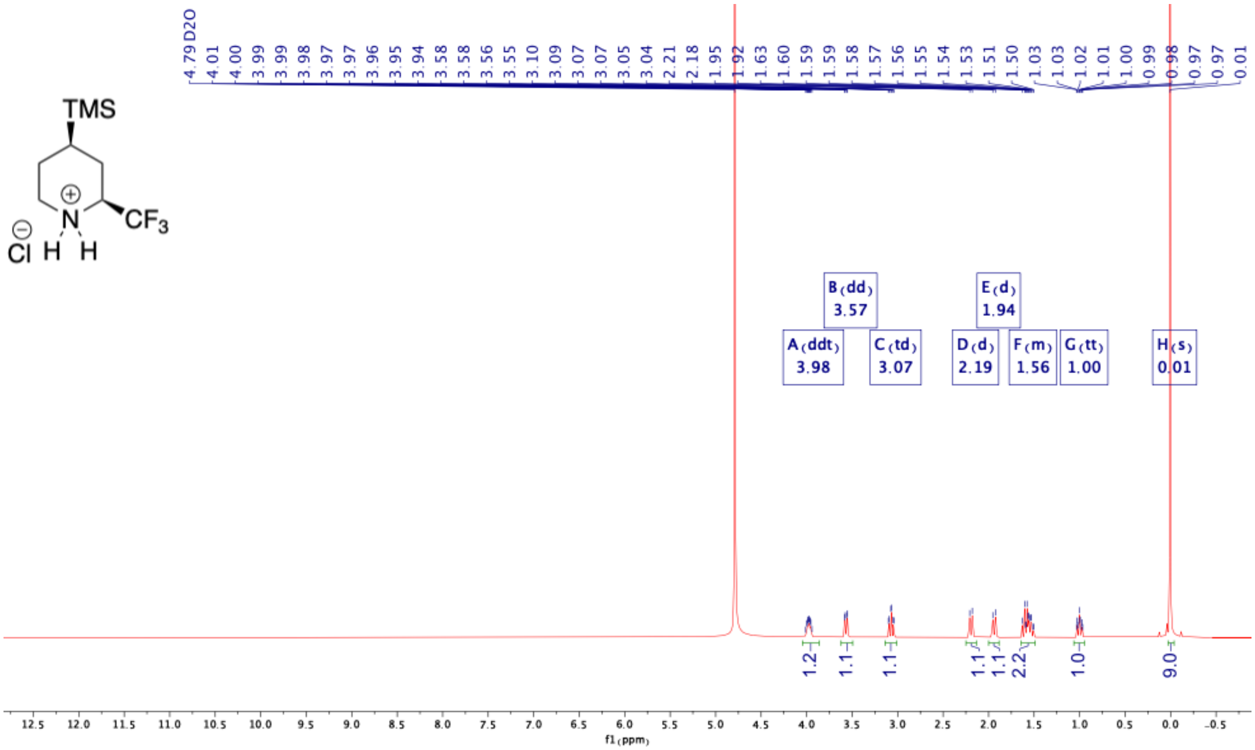

$^{13}C\{^{1}H\}$ (126 MHz, D_2O) NMR of 2-(Trifluoromethyl)-4-(trimethylsilyl)piperidine (2x)

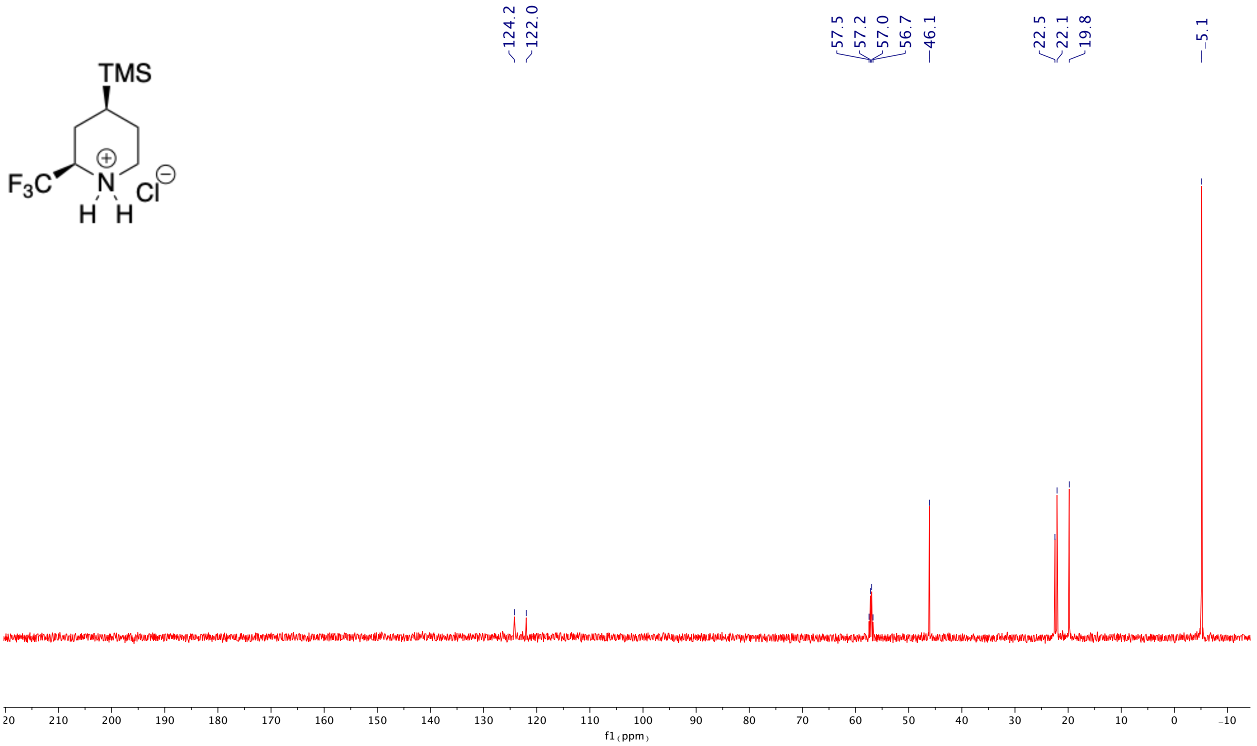

^{29}Si NMR (99MHz, D_2O) of 2-(Trifluoromethyl)-4-(trimethylsilyl)piperidine (2x)

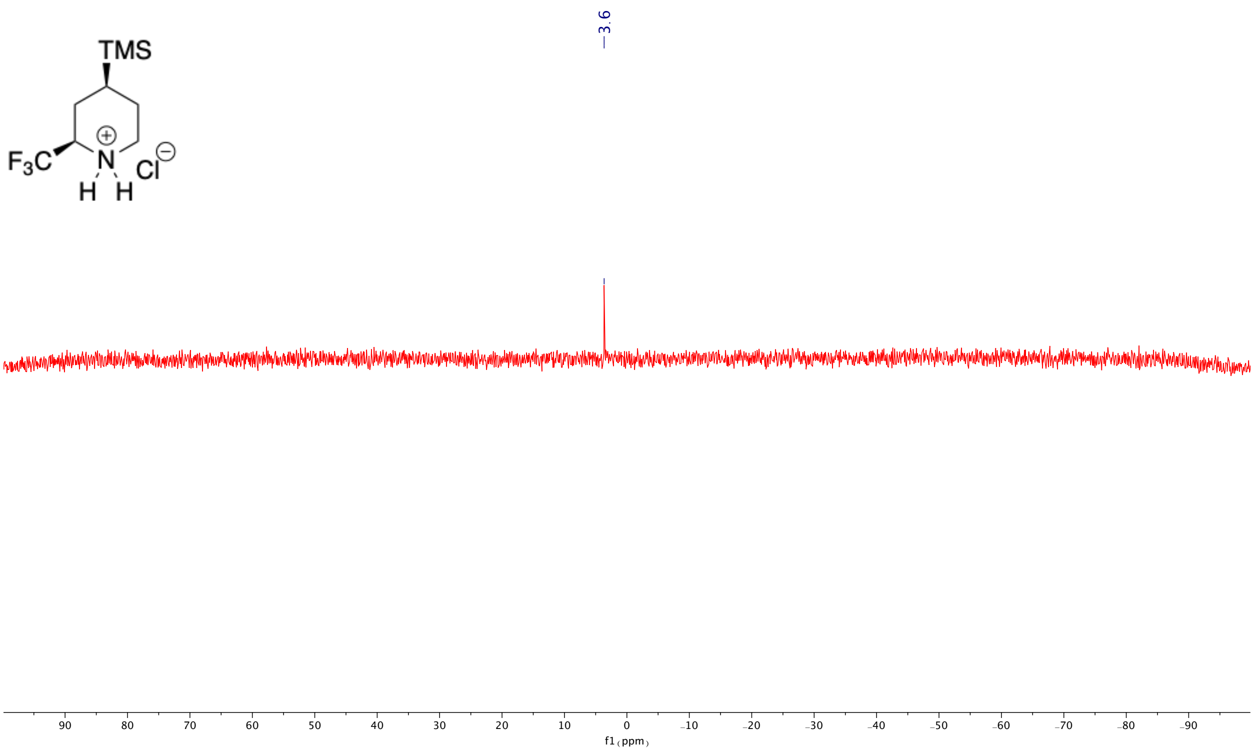

^{19}F NMR (470 MHz, D_2O) of 2-(Trifluoromethyl)-4-(trimethylsilyl)piperidine (2x)

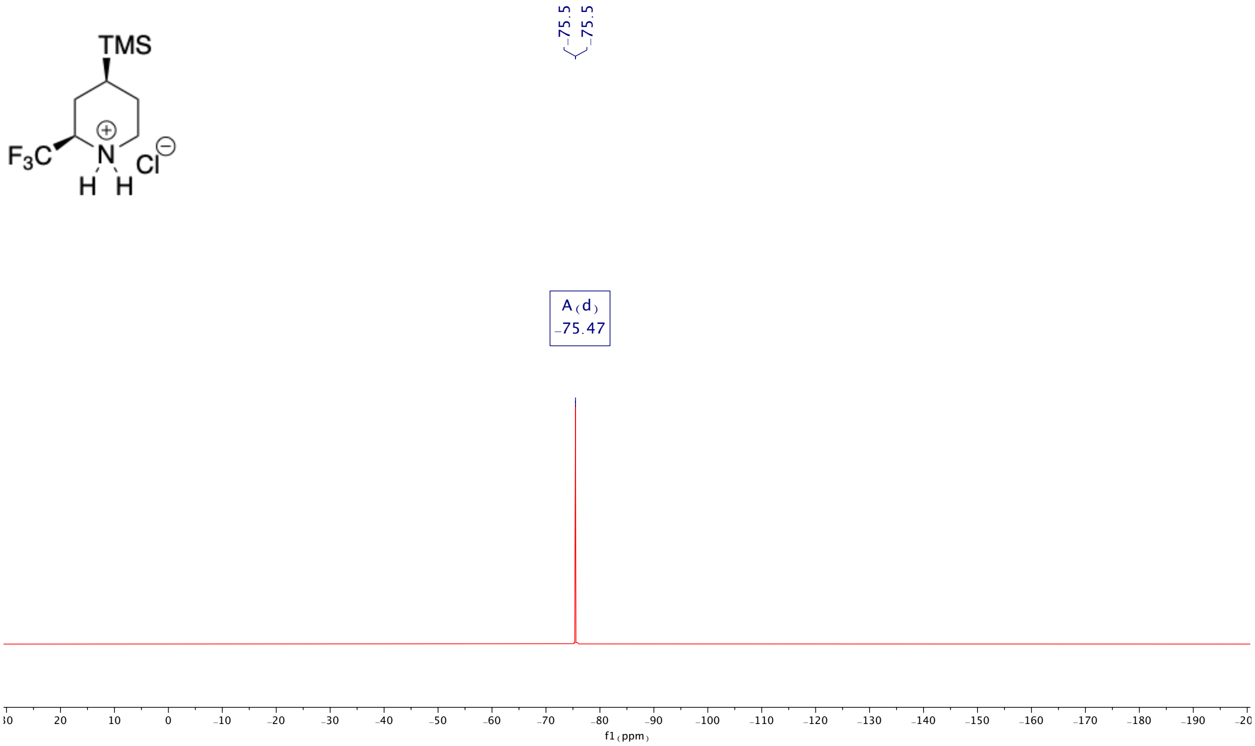

NOE of 2-(Trifluoromethyl)-4-(trimethylsilyl)piperidine (2x)

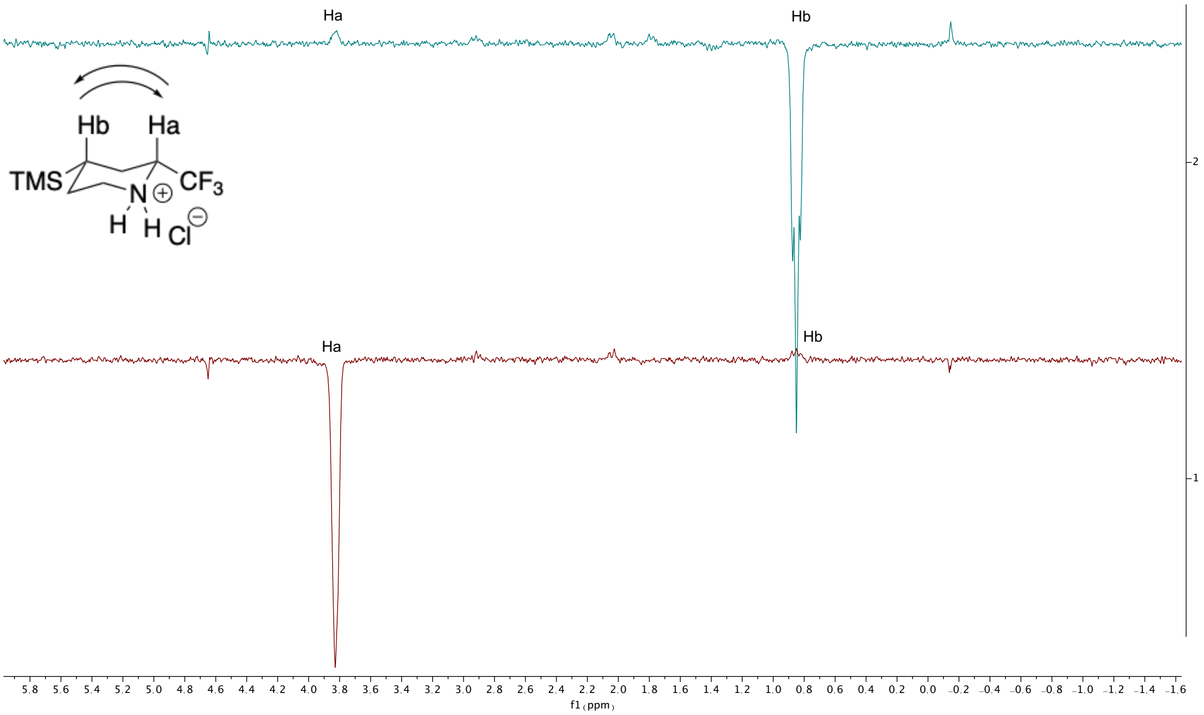

^{1}H NMR (500MHz, D_2O) of 2-(Trifluoromethyl)-5-(trimethylsilyl)piperidine (2y)

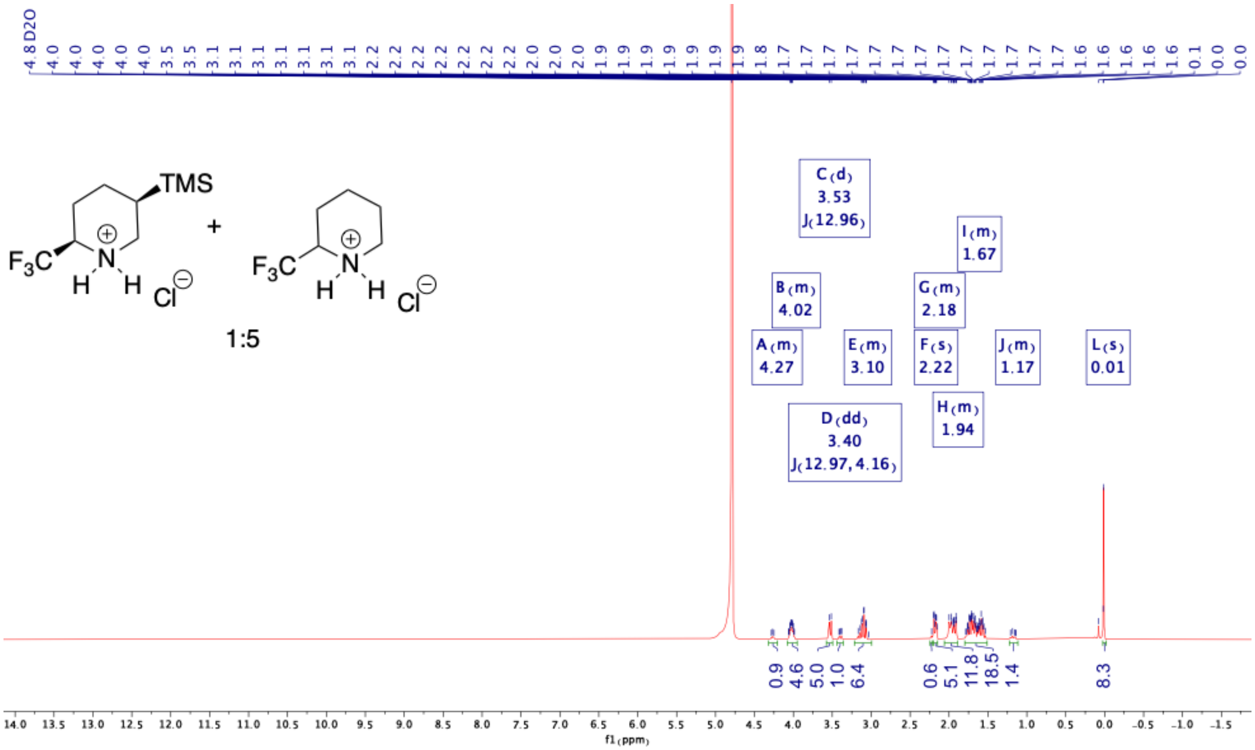

^{13}C{^{1}H} (126 MHz, D_2O) NMR of 2-(Trifluoromethyl)-5-(trimethylsilyl)piperidine (2y)

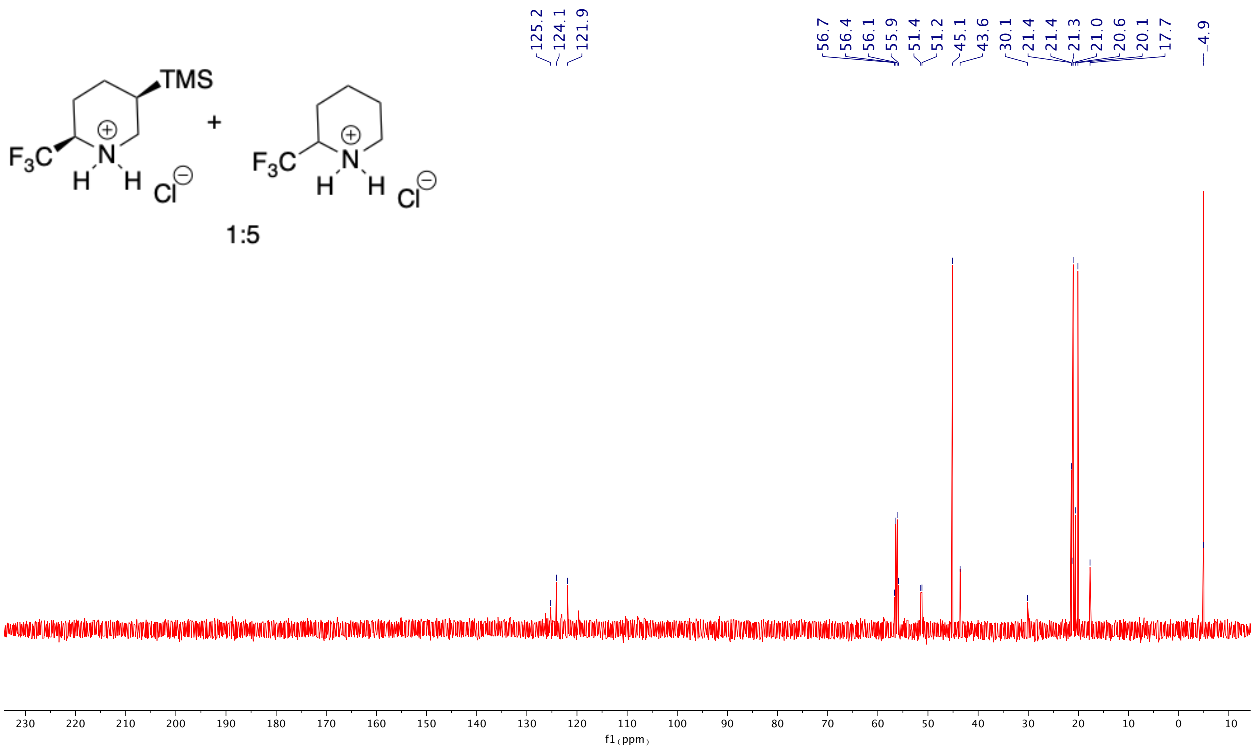

^{19}F NMR (470 MHz, D_2O) of 2-(Trifluoromethyl)-5-(trimethylsilyl)piperidine (2y)

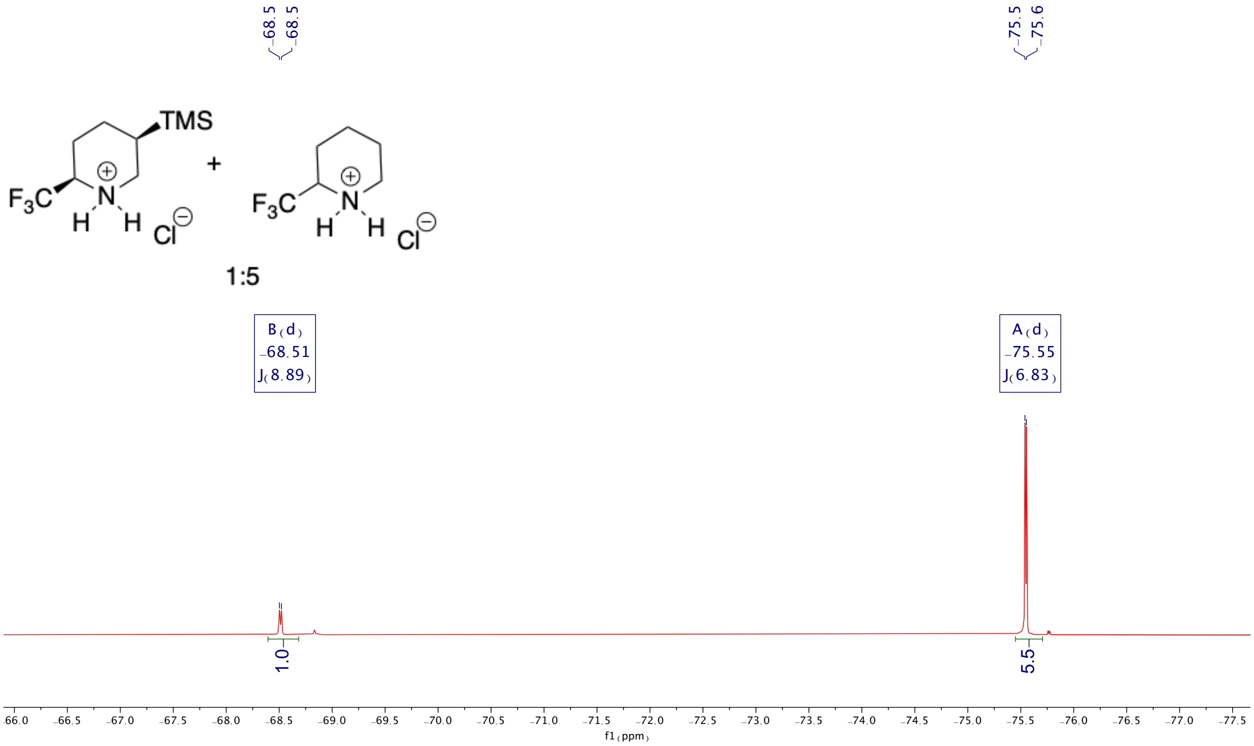

^{1}H NMR (500MHz, C_6D_6) of 3-(2,3-dihydrobenzofuran-2-yl)-1,1,1,3,5,5,5-heptamethyltrisiloxane (2z)

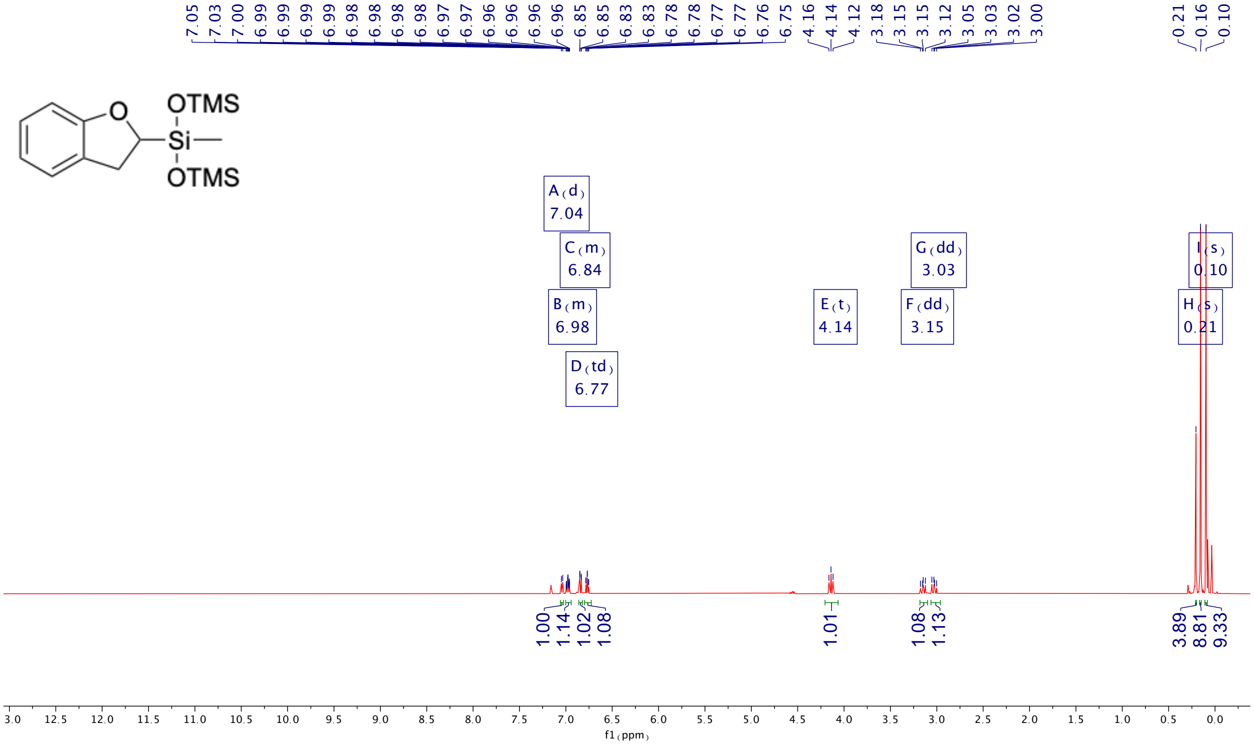

$^{13}C\{^{1}H\}$ (126 MHz C_6D_6) NMR of 3-(2,3-dihydrobenzofuran-2-yl)-1,1,1,3,5,5,5-heptamethyltrisiloxane (2z)

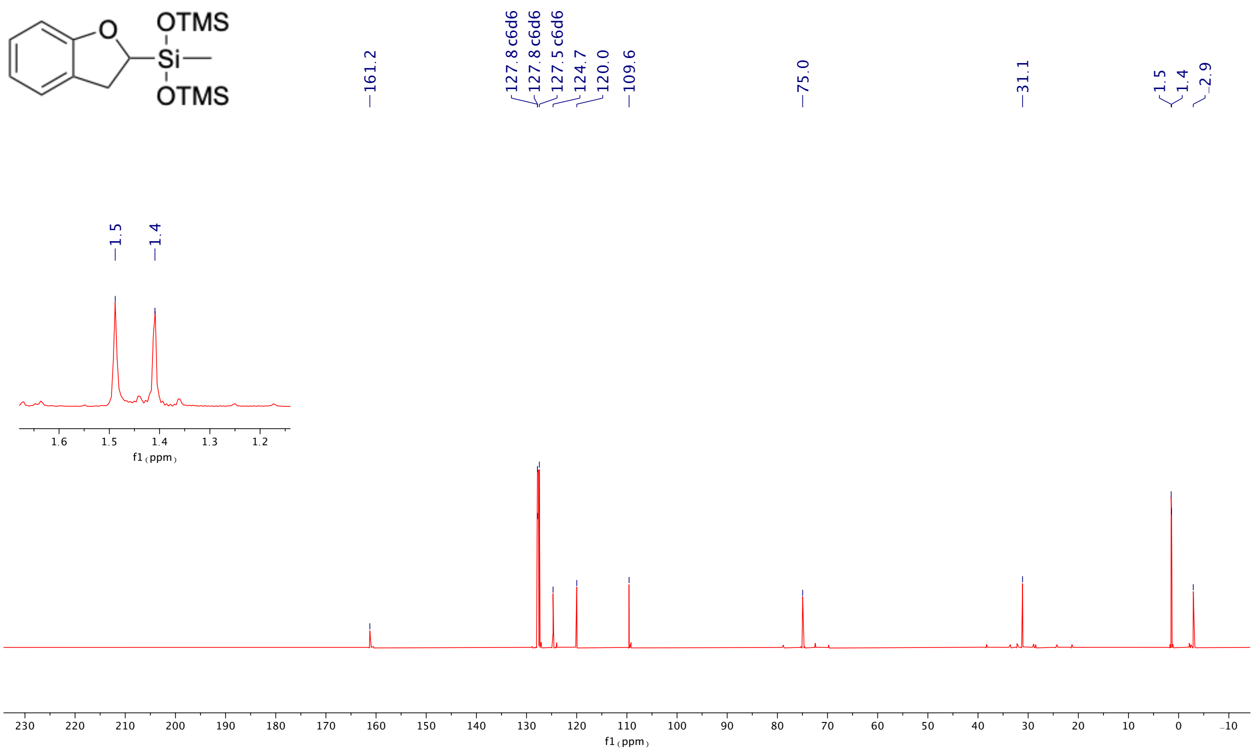

^{29}Si NMR (99MHz, $CDCl_3$) of 3-(2,3-dihydrobenzofuran-2-yl)-1,1,1,3,5,5,5-heptamethyltrisiloxane (2z)

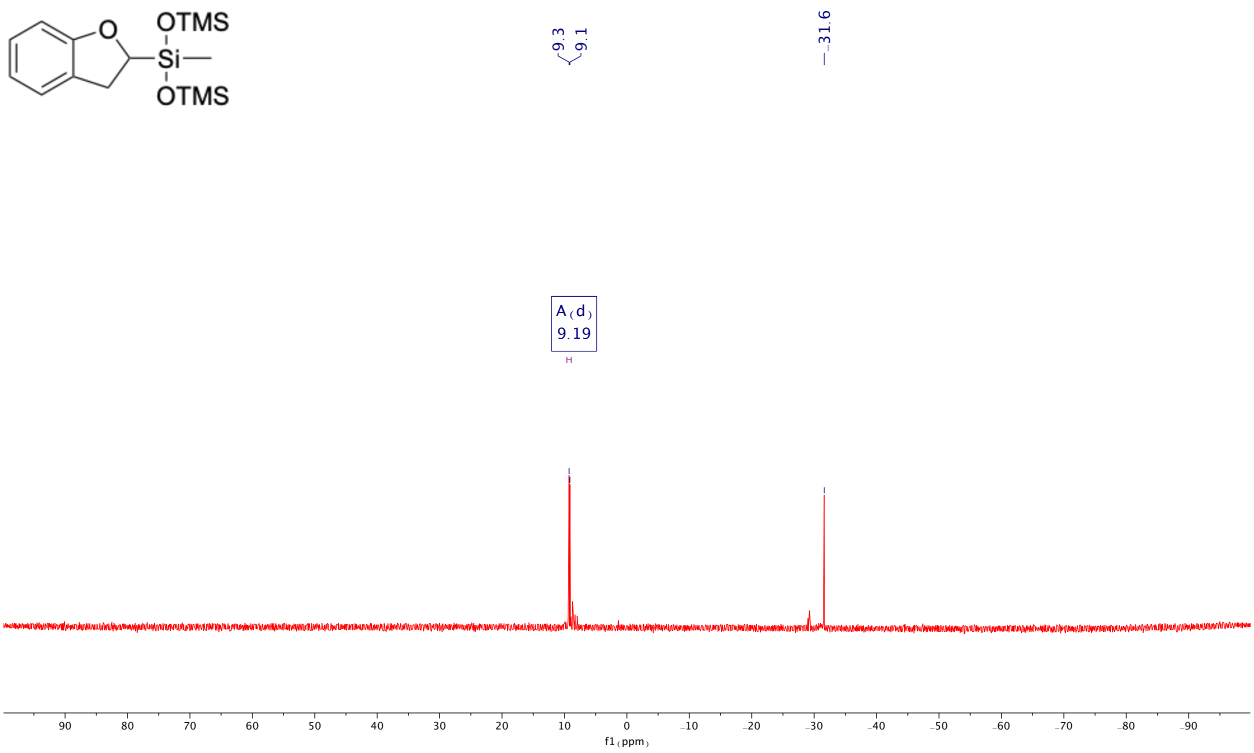

^{1}H NMR (500MHz, $CDCl_3$) of Cyclohexyltriethoxysilane (2aa)

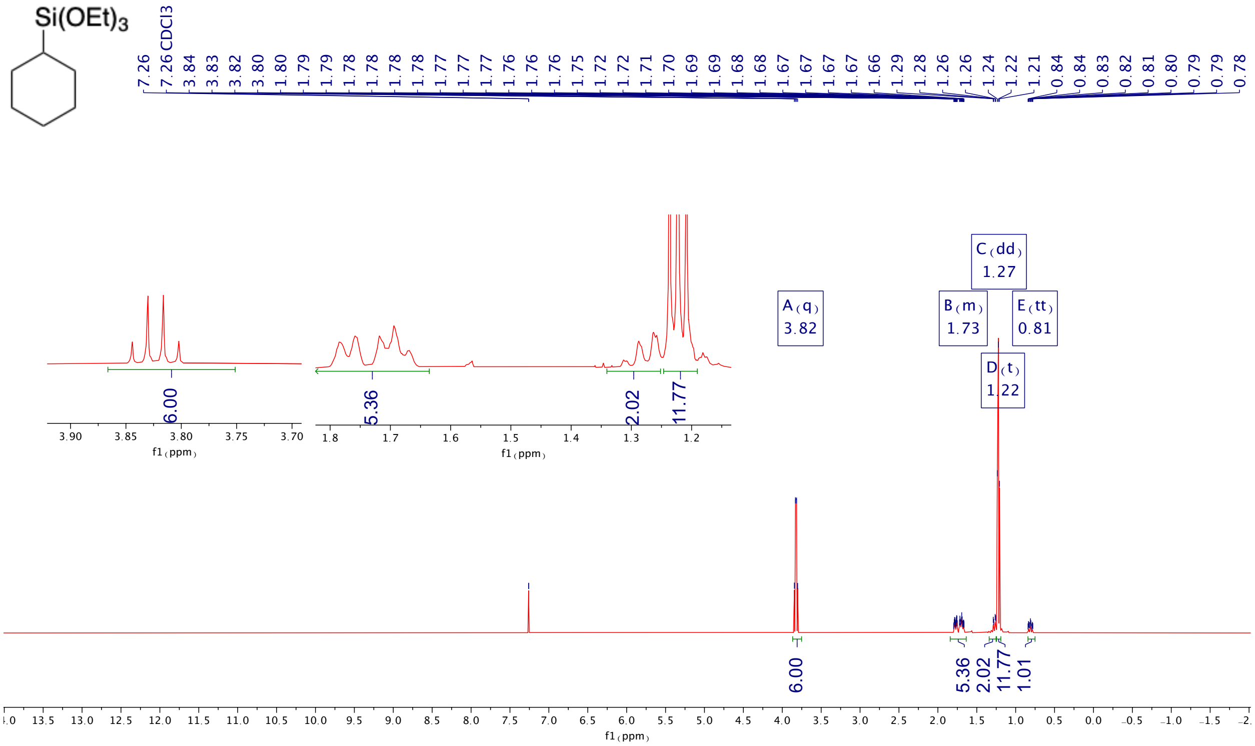

^{13}C{^{1}H} (126 MHz, $CDCl_3$) NMR of Cyclohexyltriethoxysilane (2aa)

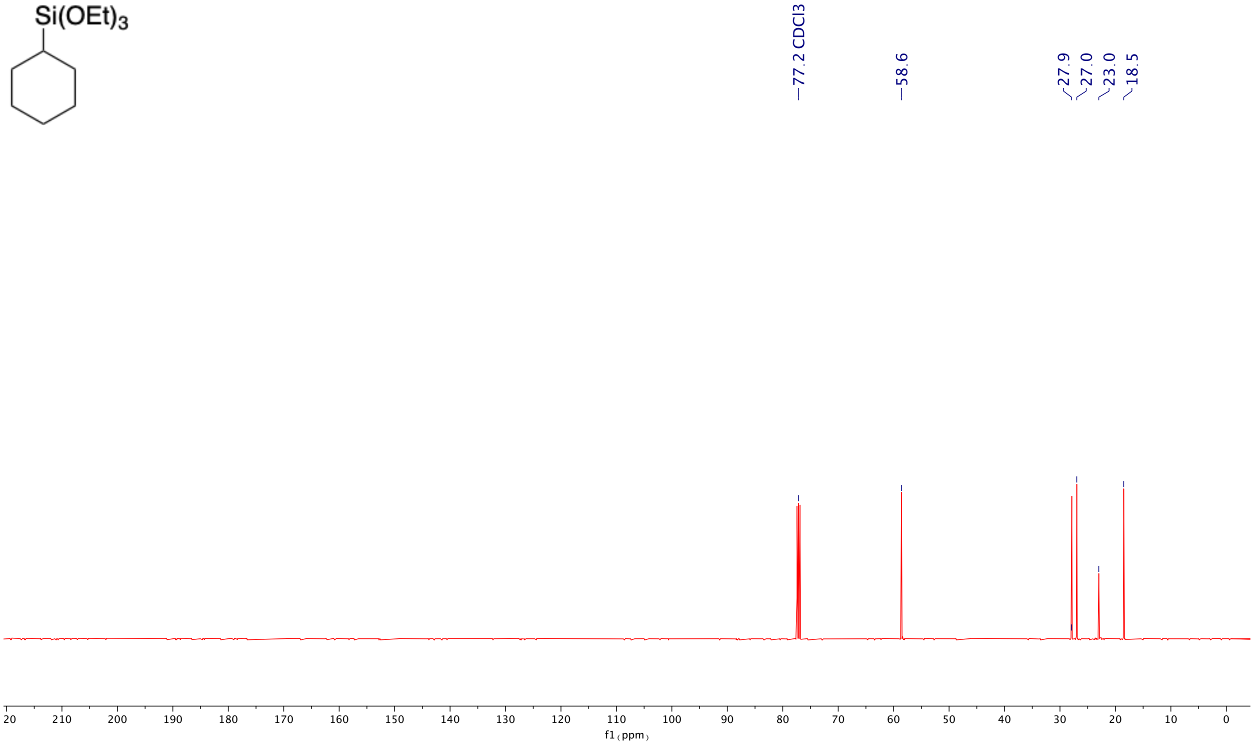

^{29}Si NMR (99MHz, $CDCl_3$) of Cyclohexyltriethoxysilane (2aa)

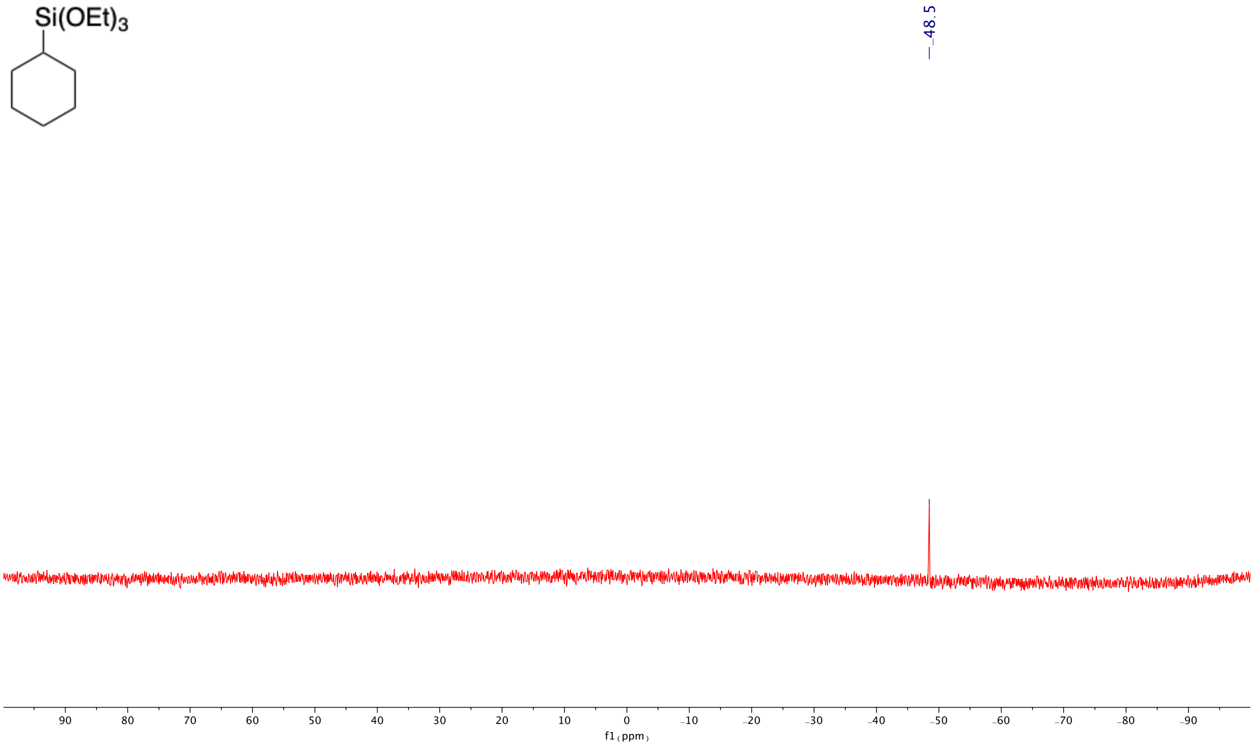

^{1}H NMR (500MHz, C_6D_6) of *t*-butyl-4-(4,4,5,5-tetramethyl-1,3,2-dioxaborolan-2-yl)-2(trimethylsilyl)pyrrolidine-1-carboxylate (2ab)

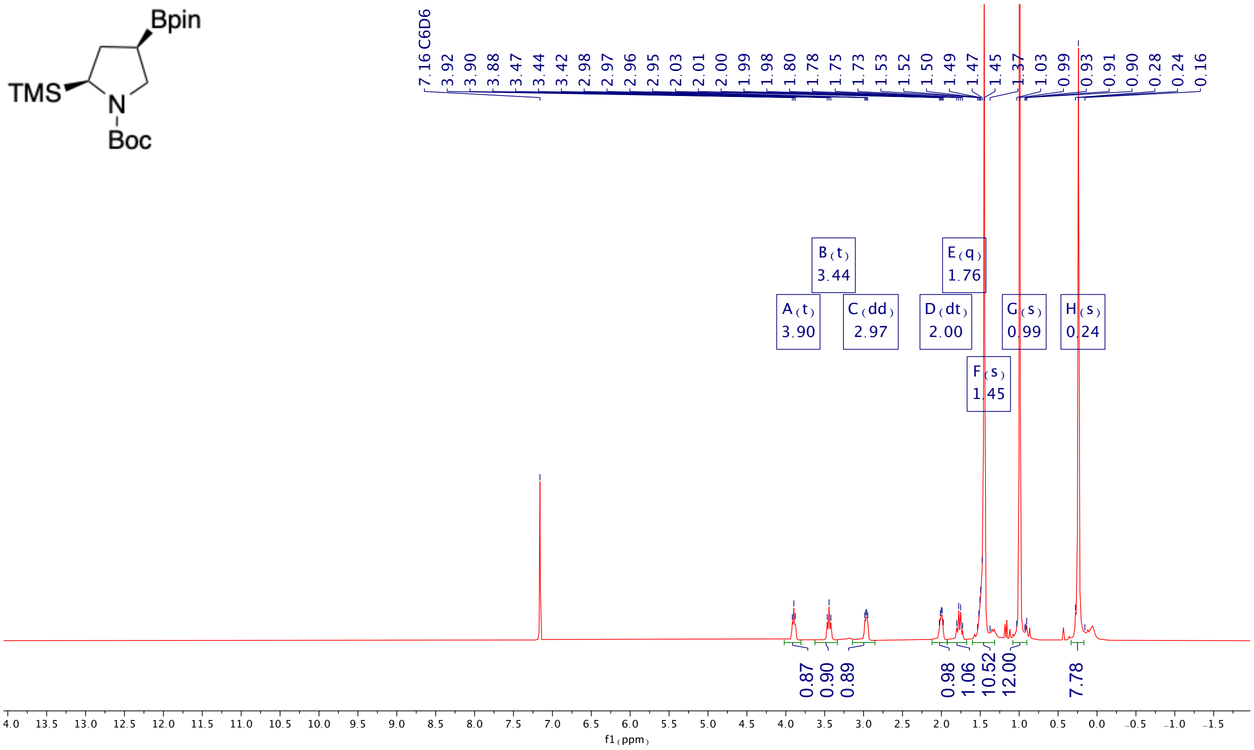

$^{13}C\{^{1}H\}$ (126 MHz, C_6D_6) of *t*-butyl-4-(4,4,5,5-tetramethyl-1,3,2-dioxaborolan-2-yl)-2(trimethylsilyl)pyrrolidine-1-carboxylate (2ab)

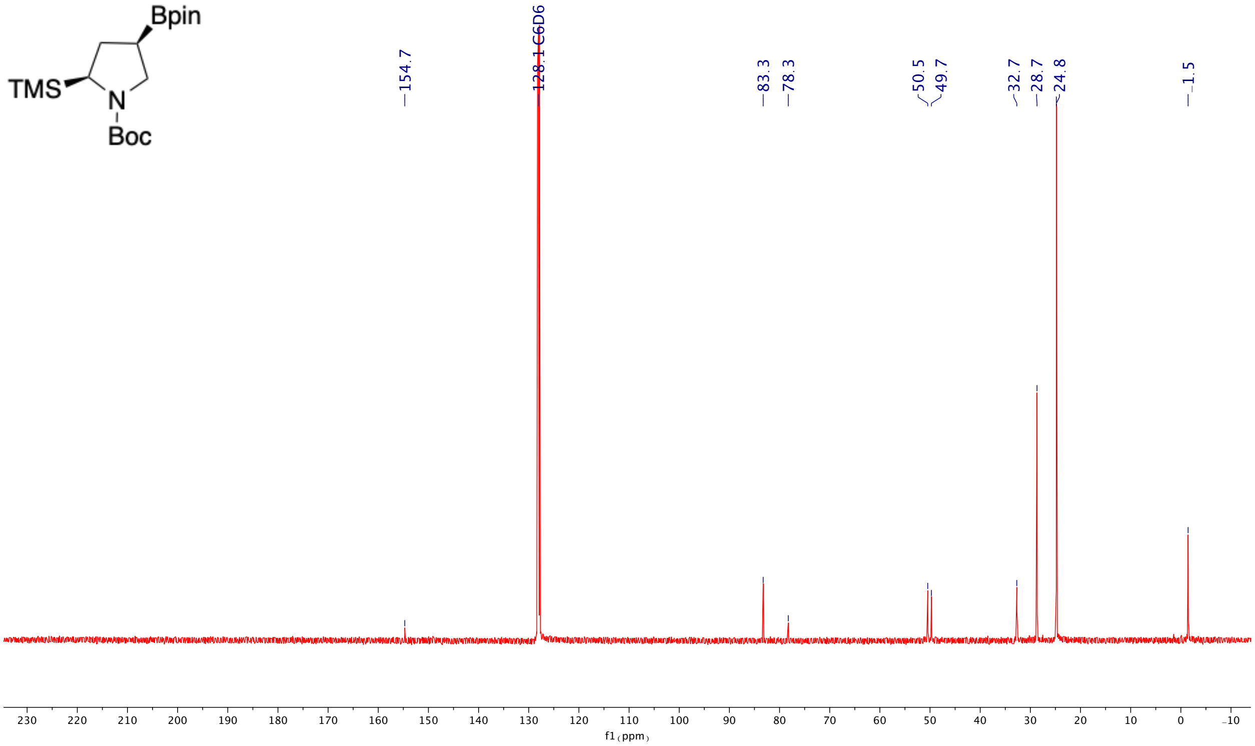

^{11}B NMR (160 MHz, C_6D_6)of *t*-butyl-4-(4,4,5,5-tetramethyl-1,3,2-dioxaborolan-2-yl)-2(trimethylsilyl)pyrrolidine-1-carboxylate (2ab)

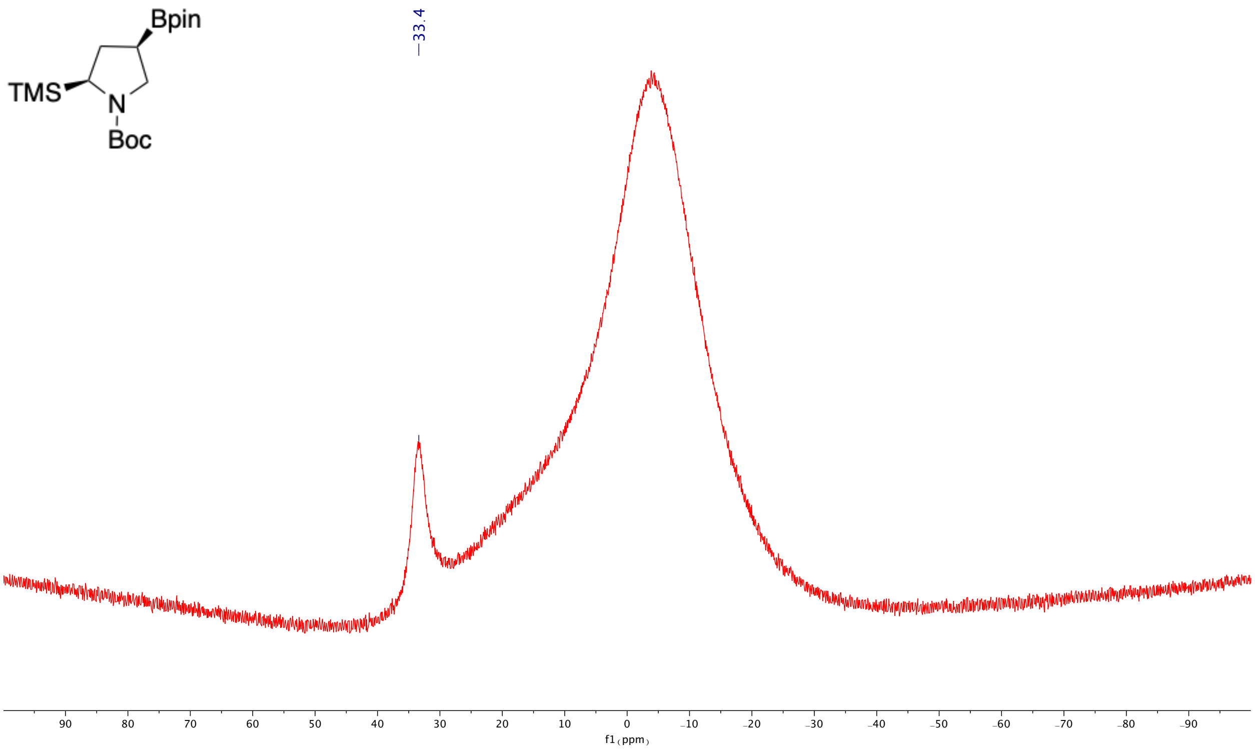

^{29}Si NMR (99 MHz, C_6D_6) *t*-butyl-4-(4,4,5,5-tetramethyl-1,3,2-dioxaborolan-2-yl)-2-trimethylsilyl)pyrrolidine-1-carboxylate (2ab)

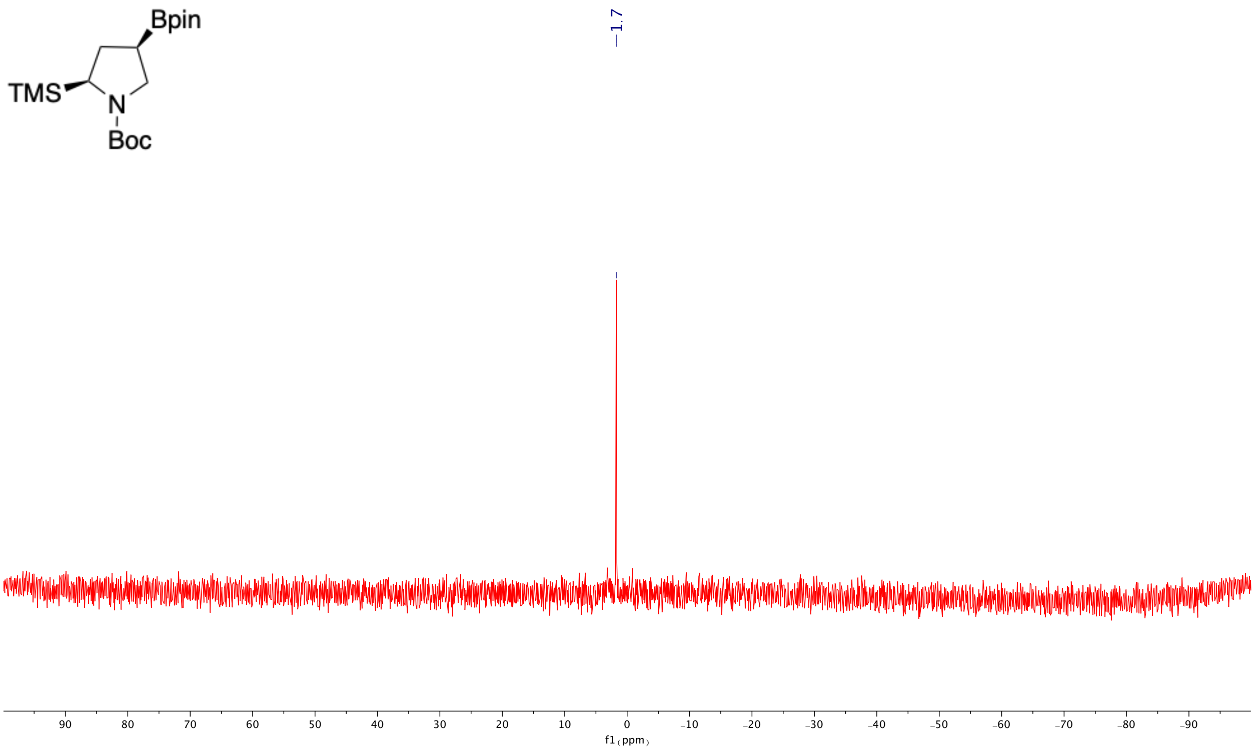

2D-NOE of *t*-butyl-4-(4,4,5,5-tetramethyl-1,3,2-dioxaborolan-2-yl)-2(trimethylsilyl)pyrrolidine-1-carboxylate (2ab)

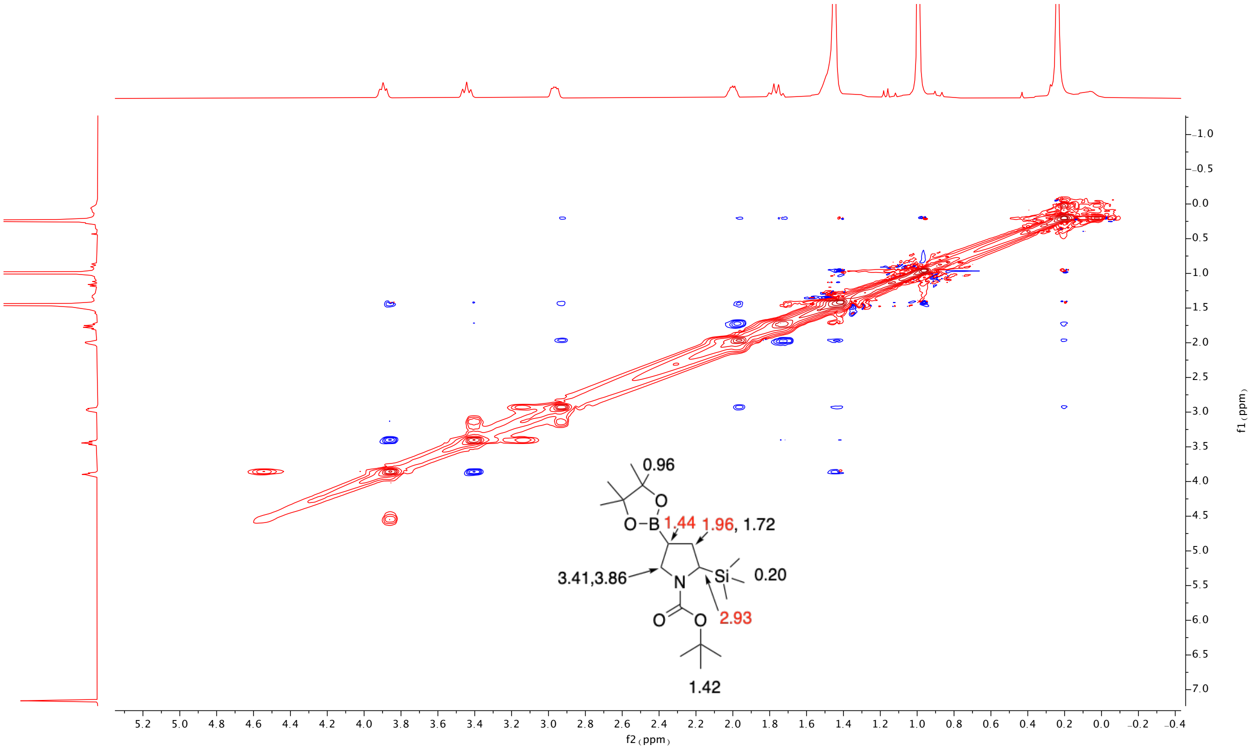

COSY of *t*-butyl-4-(4,4,5,5-tetramethyl-1,3,2-dioxaborolan-2-yl)-2(trimethylsilyl)pyrrolidine-1-carboxylate (2ab)

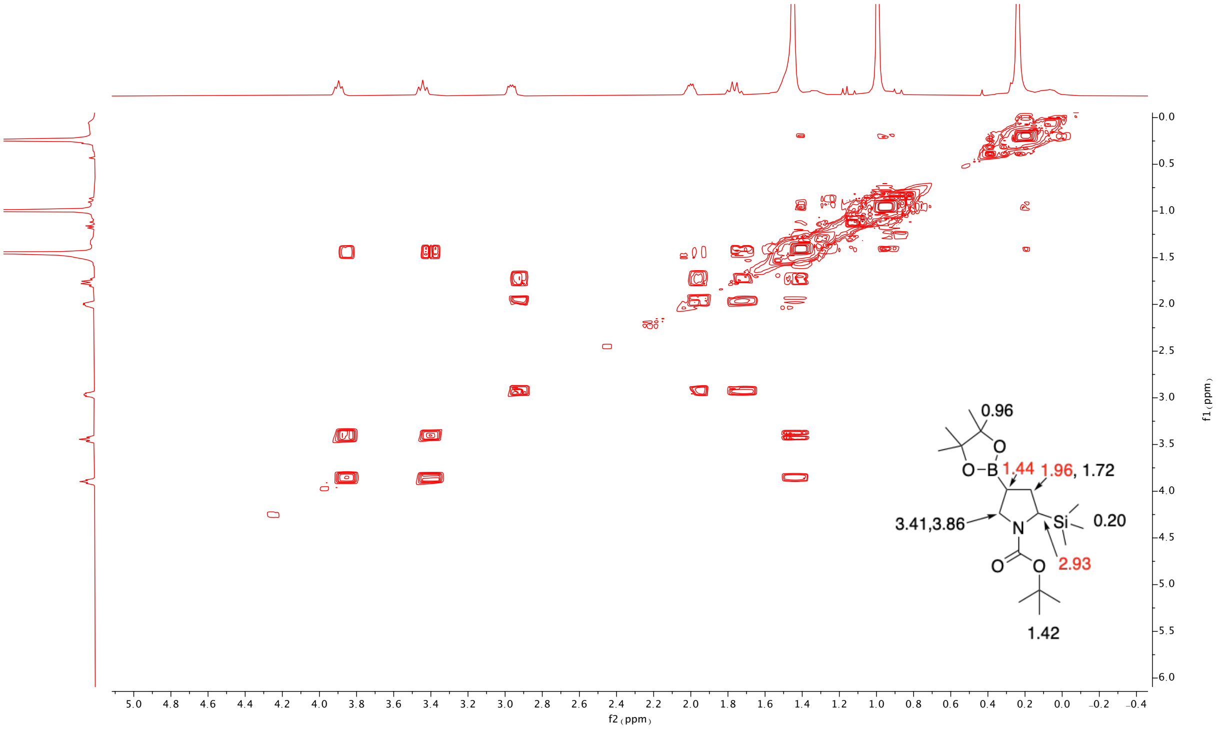

^{1}H NMR (500MHz, $CDCl_3$) of Trimethyl(3-(4,4,5,5-tetramethyl-1,3,2-dioxaborolan-2-yl)cyclohexyl)silane (2ae)

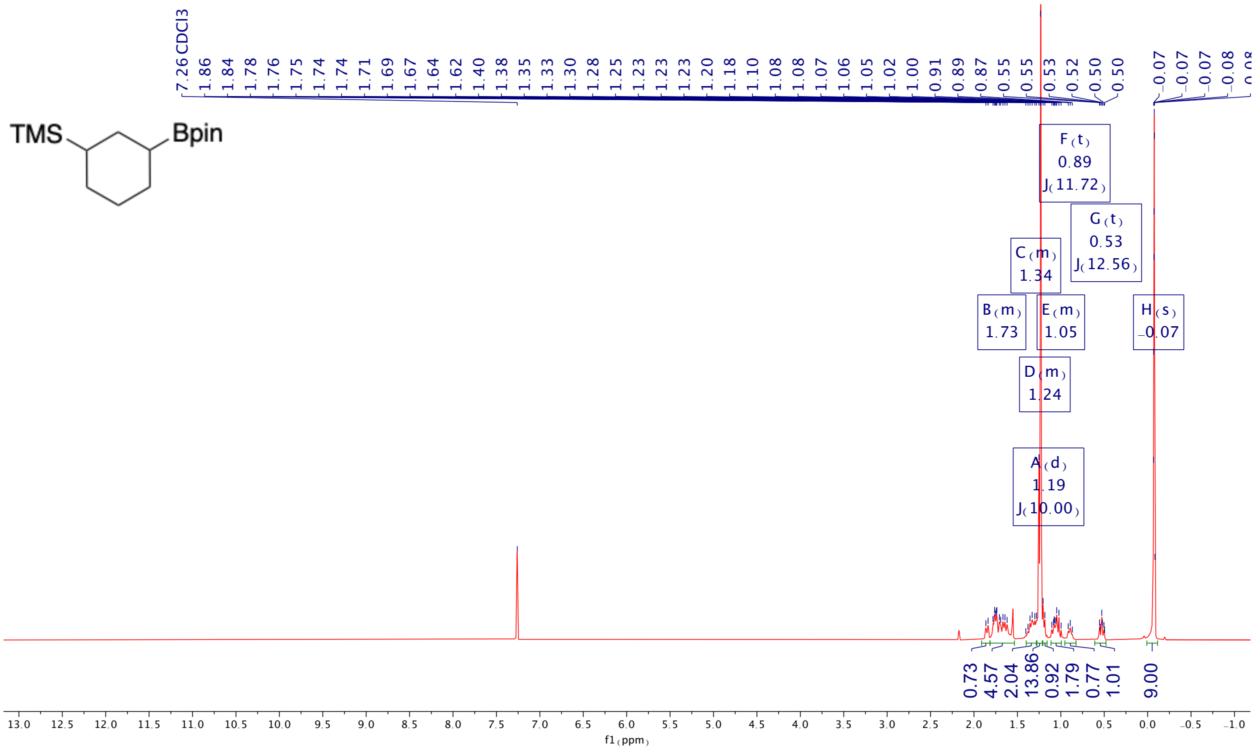

$^{13}C\{^1H\}$ (126 MHz, $CDCl_3$) NMR of Trimethyl(3-(4,4,5,5-tetramethyl-1,3,2-dioxaborolan-2-yl)cyclohexyl)silane (2ae)

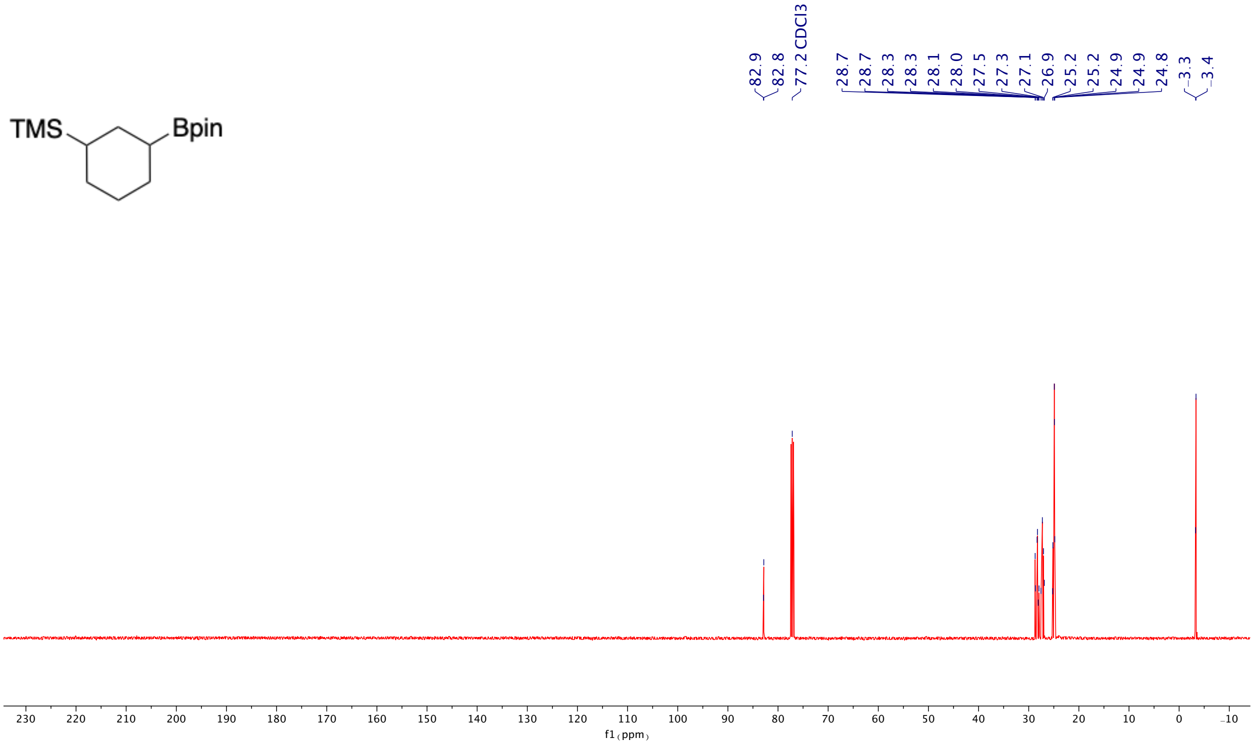

^{29}Si NMR (99MHz, $CDCl_3$) of Trimethyl(3-(4,4,5,5-tetramethyl-1,3,2-dioxaborolan-2-yl)cyclohexyl)silane (2ae)

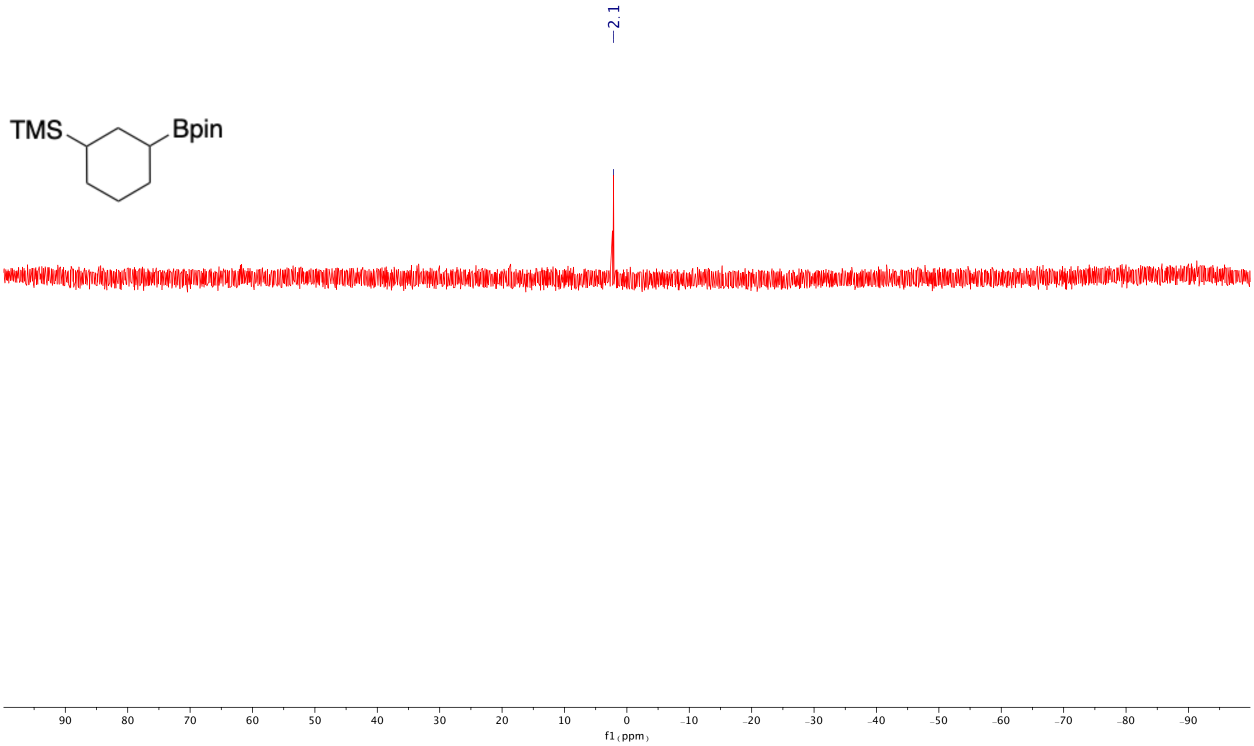

^{11}B NMR (160MHz, $CDCl_3$) of Trimethyl(3-(4,4,5,5-tetramethyl-1,3,2-dioxaborolan-2-yl)cyclohexyl)silane (2ae)

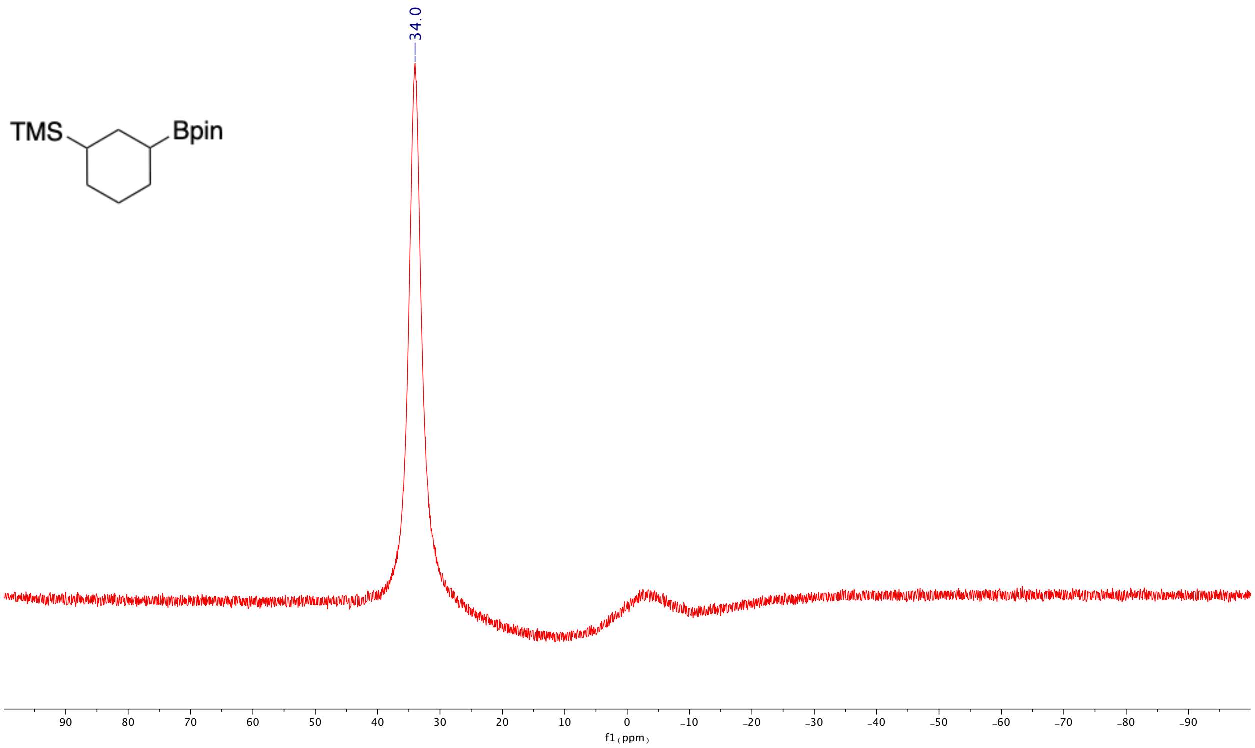

^{1}H NMR (500MHz, D_2O) of 4-(4,4,5,5-Tetramethyl-1,3,2-dioxaborolan-2-yl)-2-(trimethylsilyl)piperidin-1-ium bromide (2ac)

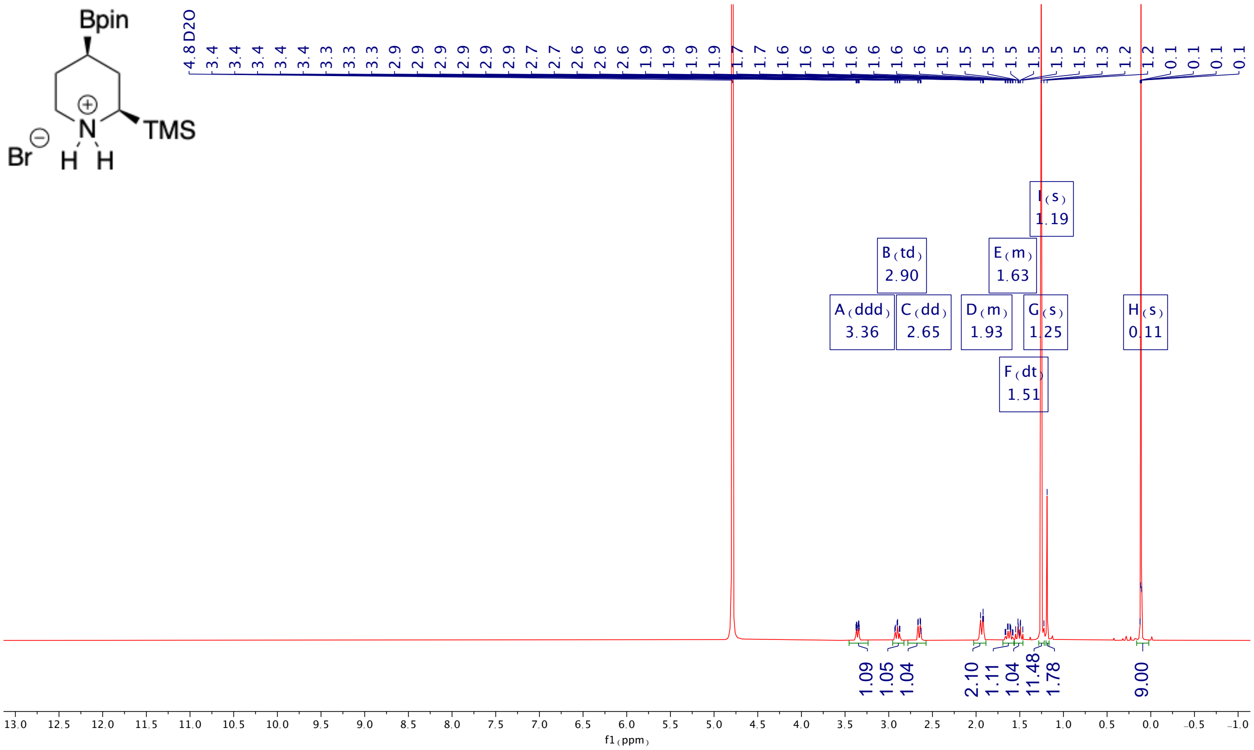

$^{13}C\{^{1}H\}$ (126 MHz, D_2O) of 4-(4,4,5,5-Tetramethyl-1,3,2-dioxaborolan-2-yl)-2-(trimethylsilyl)piperidin-1-ium bromide (2ac)

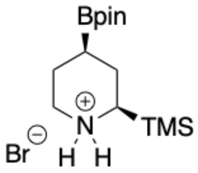

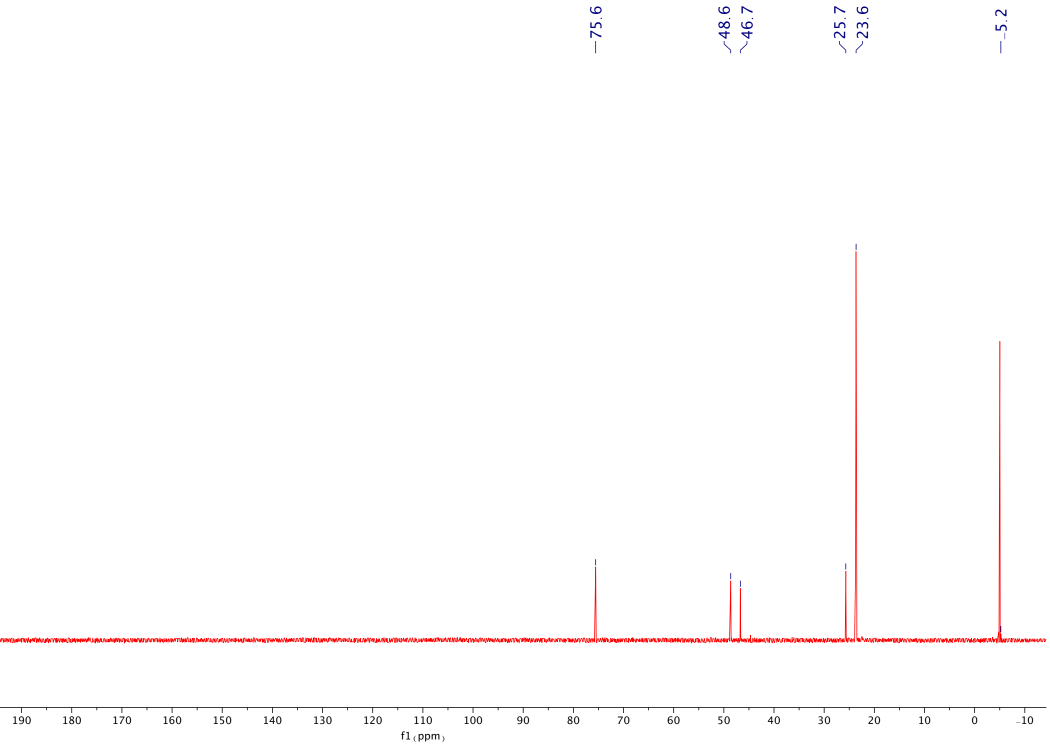

^{11}B NMR (160MHz, D_2O) of 4-(4,4,5,5-Tetramethyl-1,3,2-dioxaborolan-2-yl)-2-(trimethylsilyl)piperidin-1-ium bromide (2ac)

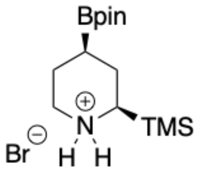

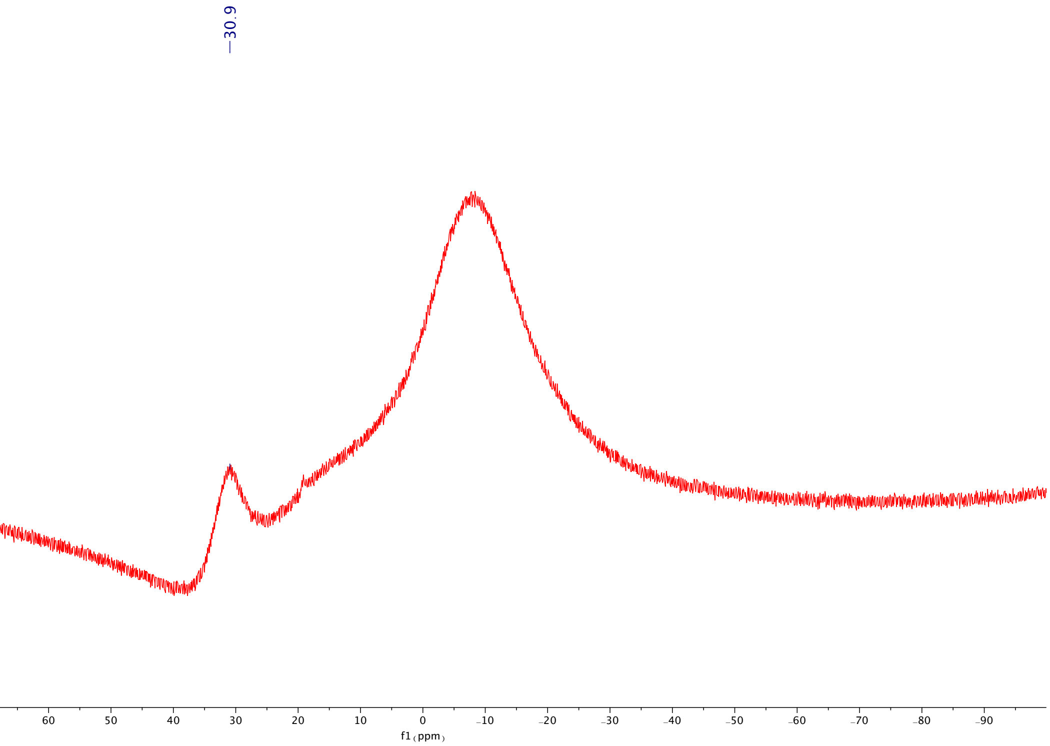

^{29}Si NMR (99MHz, $CDCl_3$) of 4-(4,4,5,5-Tetramethyl-1,3,2-dioxaborolan-2-yl)-2-(trimethylsilyl)piperidin-1-ium bromide (2ac)

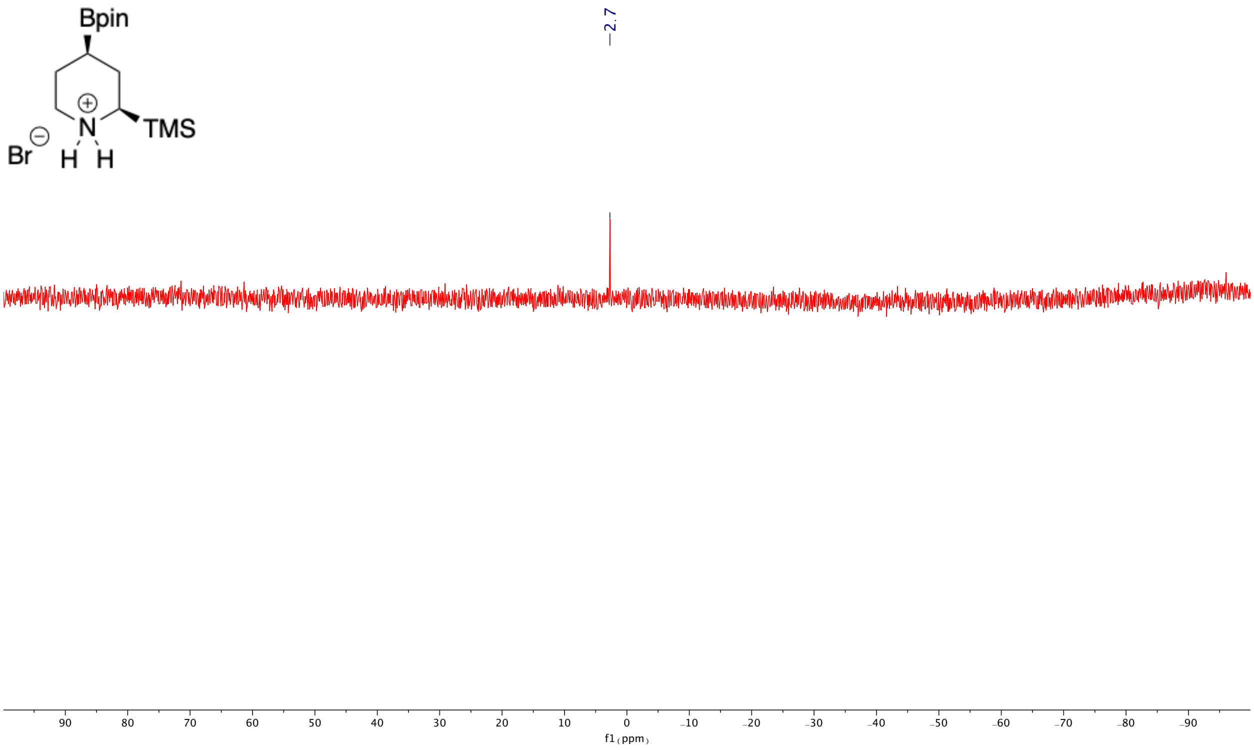

HSQC 4-(4,4,5,5-Tetramethyl-1,3,2-dioxaborolan-2-yl)-2-(trimethylsilyl)piperidin-1-ium bromide (2ac)

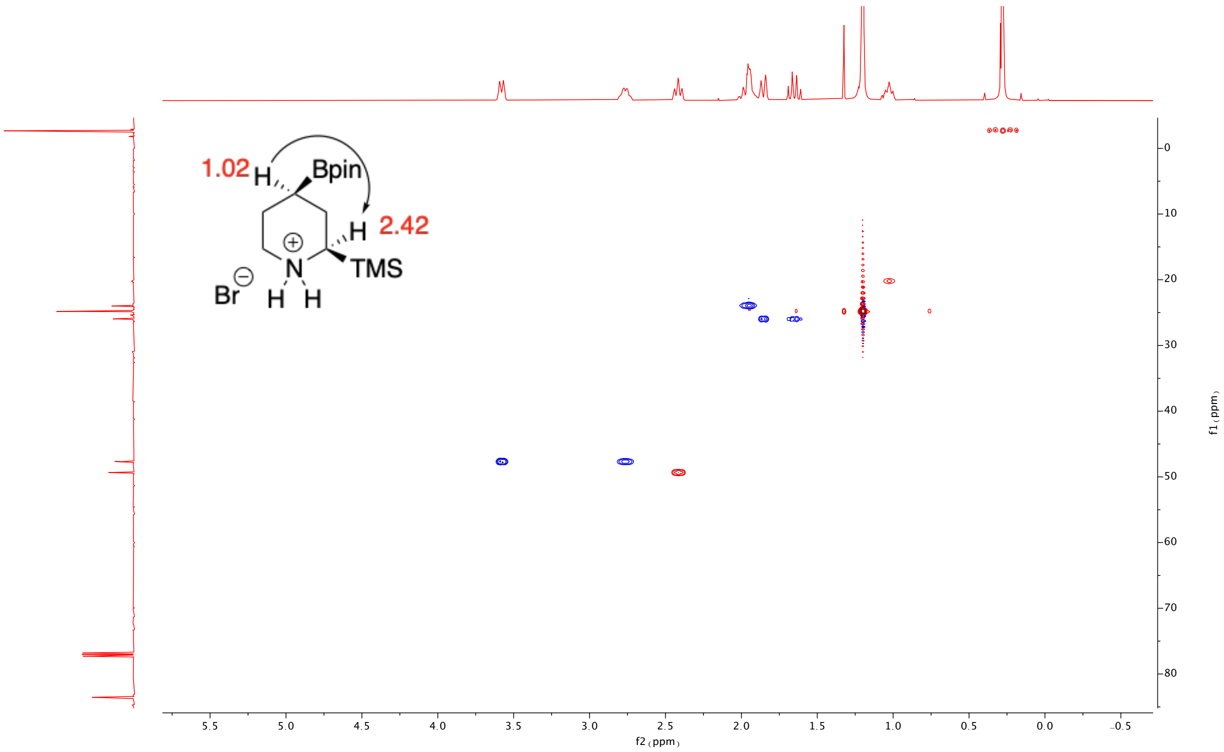

NOE of 4-(4,4,5,5-Tetramethyl-1,3,2-dioxaborolan-2-yl)-2-(trimethylsilyl)piperidin-1-ium bromide (2ac)

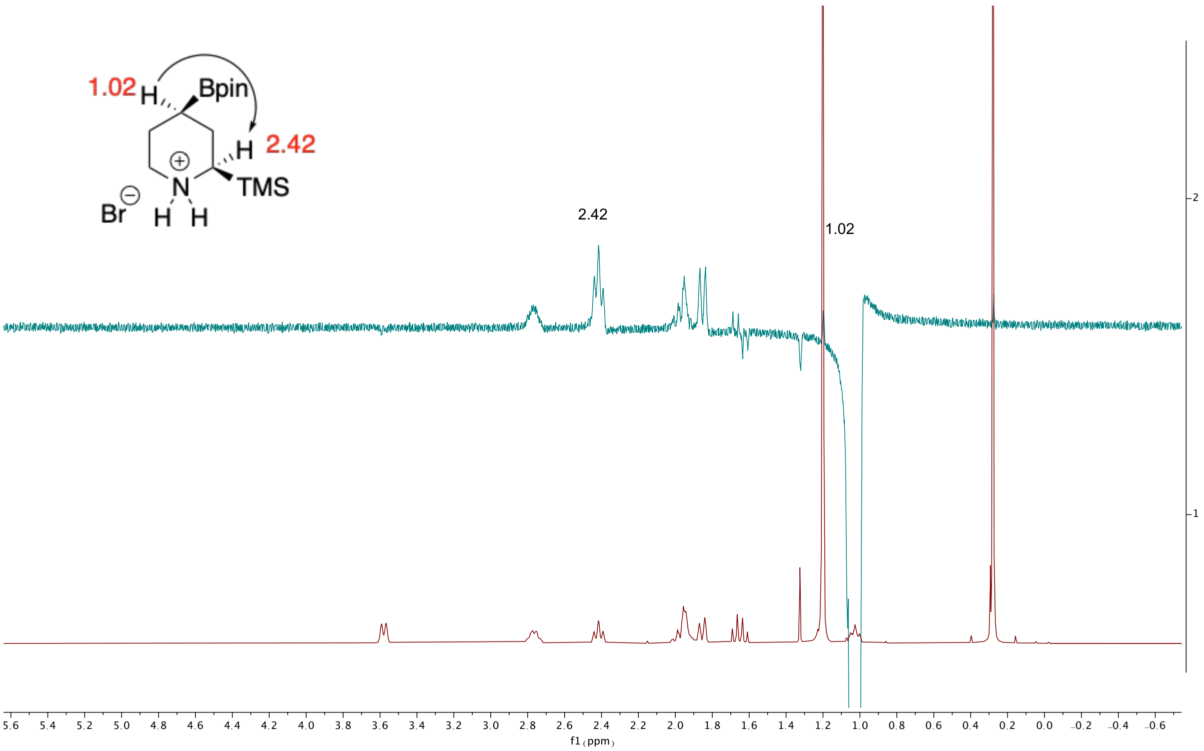

^{1}H NMR (500MHz, $CDCl_3$) of Trimethyl-4-(4,4,5,5-tetramethyl-1,3,2-dioxaborolan-2-yl)cyclohexyl)silane (2af)

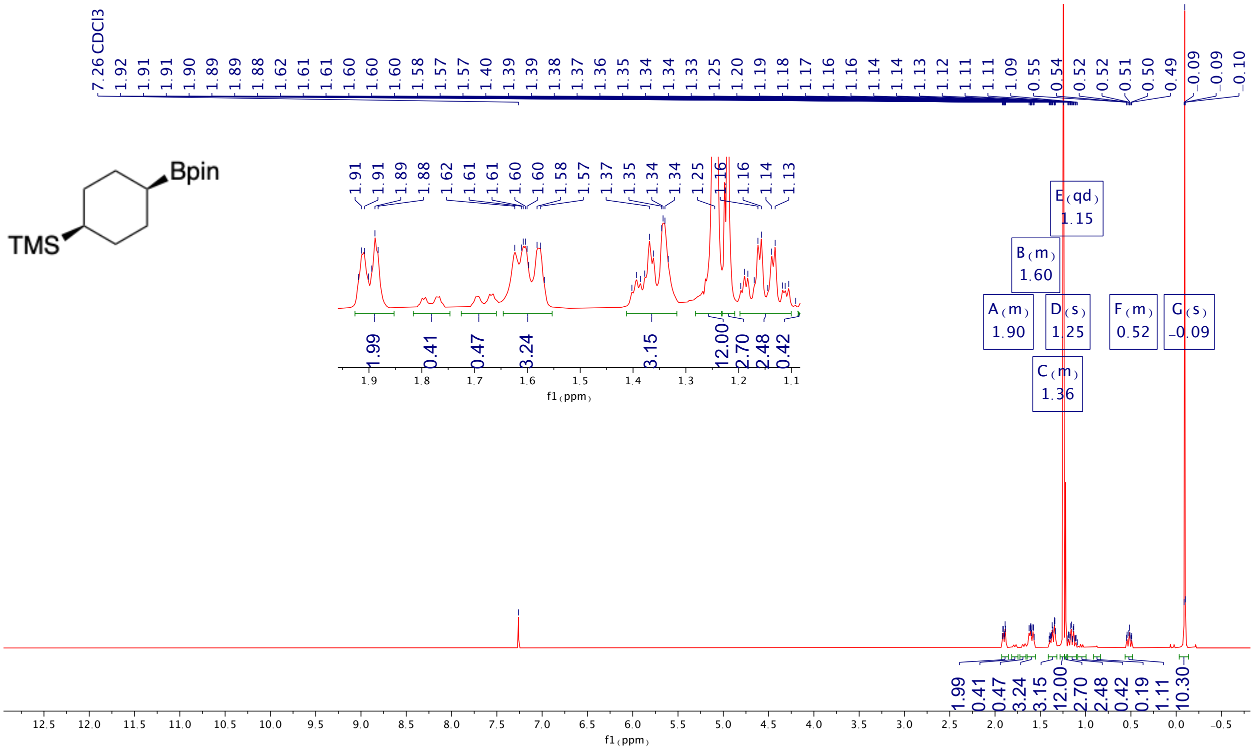

$^{13}C\{^{1}H\}$ (126 MHz, $CDCl_3$) of Trimethyl-4-(4,4,5,5-tetramethyl-1,3,2-dioxaborolan-2-yl)cyclohexyl)silane (2af)

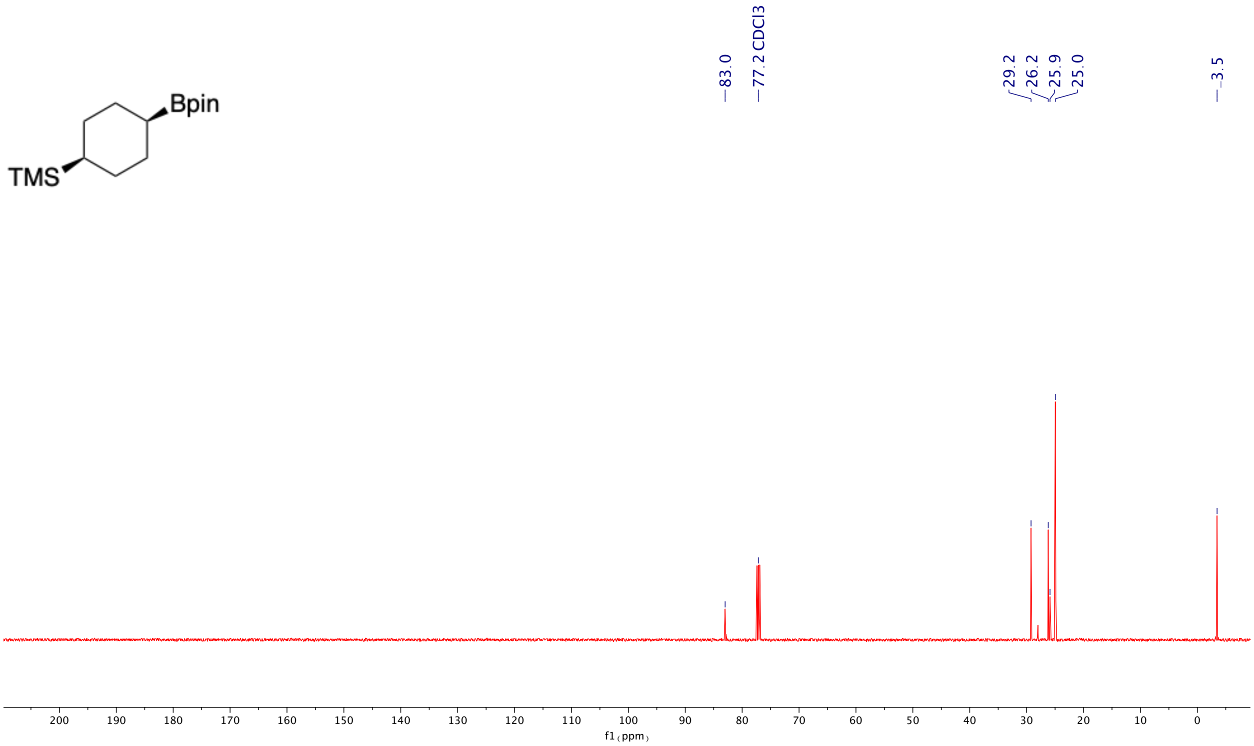

^{11}B NMR (160MHz, $CDCl_3$) of Trimethyl-4-(4,4,5,5-tetramethyl-1,3,2-dioxaborolan-2-yl)cyclohexyl)silane (2af)

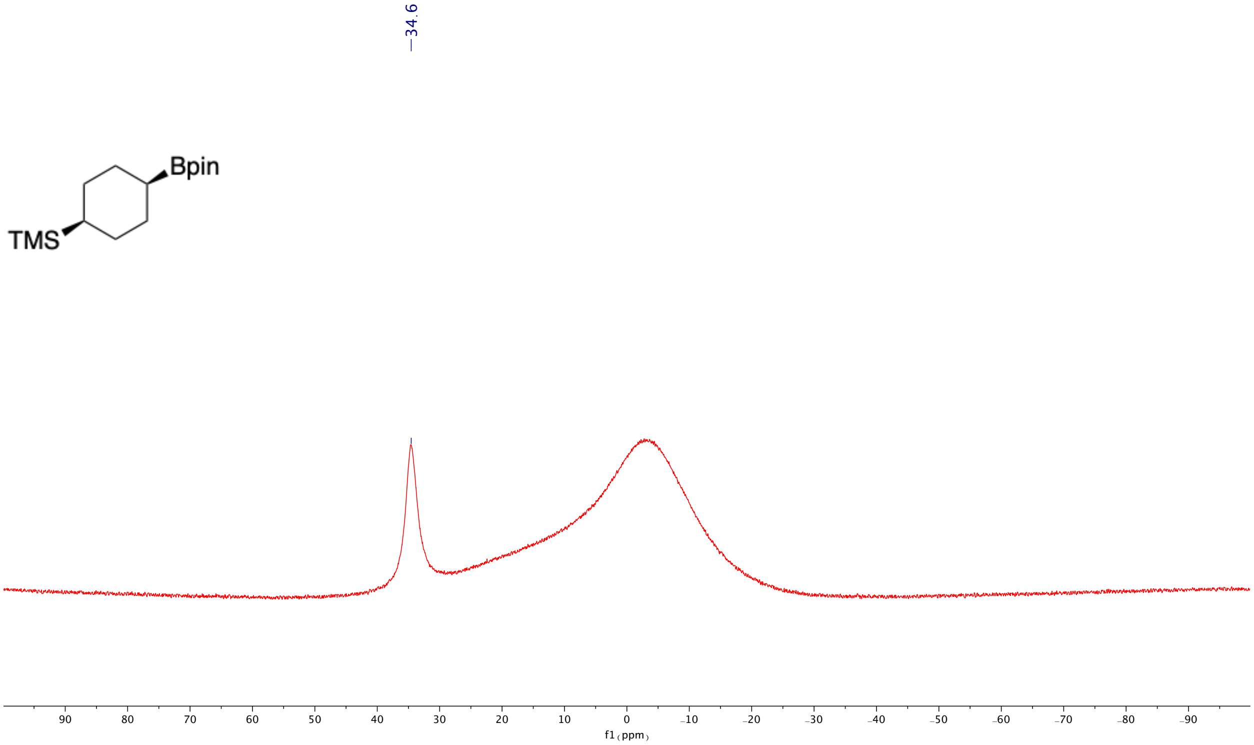

^{29}Si NMR (99MHz, $CDCl_3$) of Trimethyl-4-(4,4,5,5-tetramethyl-1,3,2-dioxaborolan-2-yl)cyclohexyl)silane (2af)

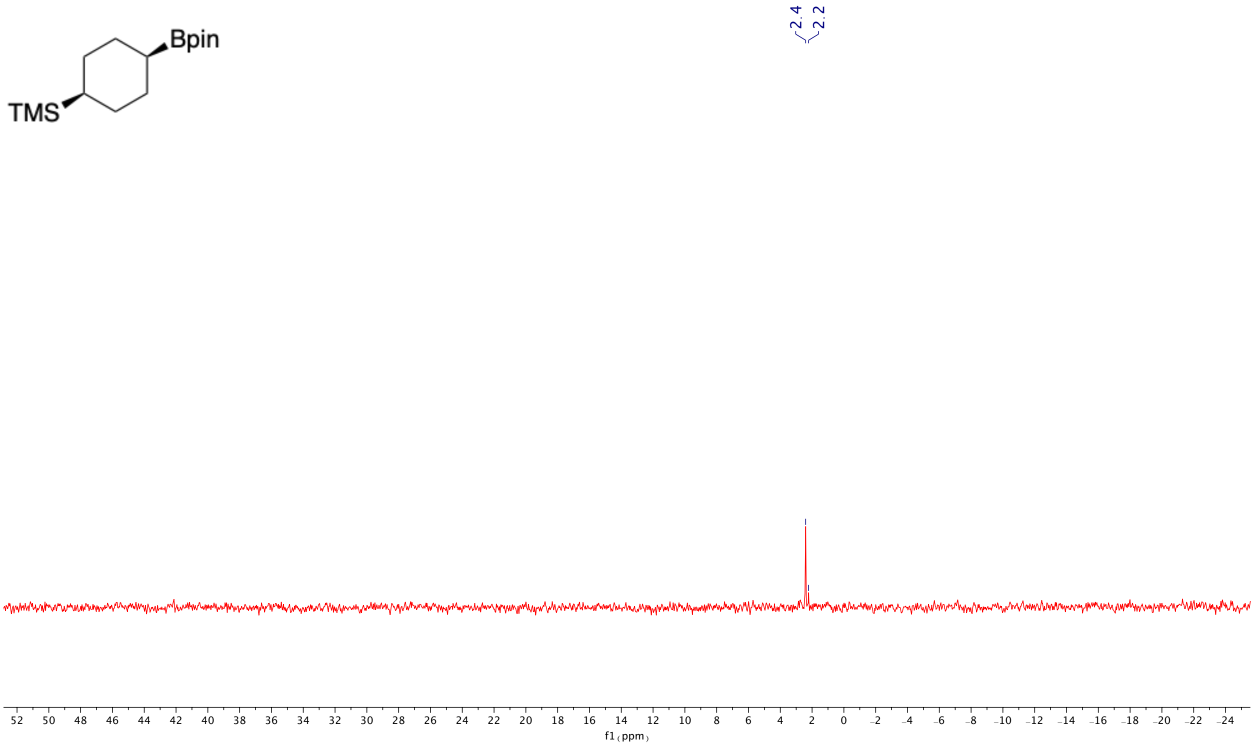

HSQC NMR Trimethyl-4-(4,4,5,5-tetramethyl-1,3,2-dioxaborolan-2-yl)cyclohexyl)silane (2af)

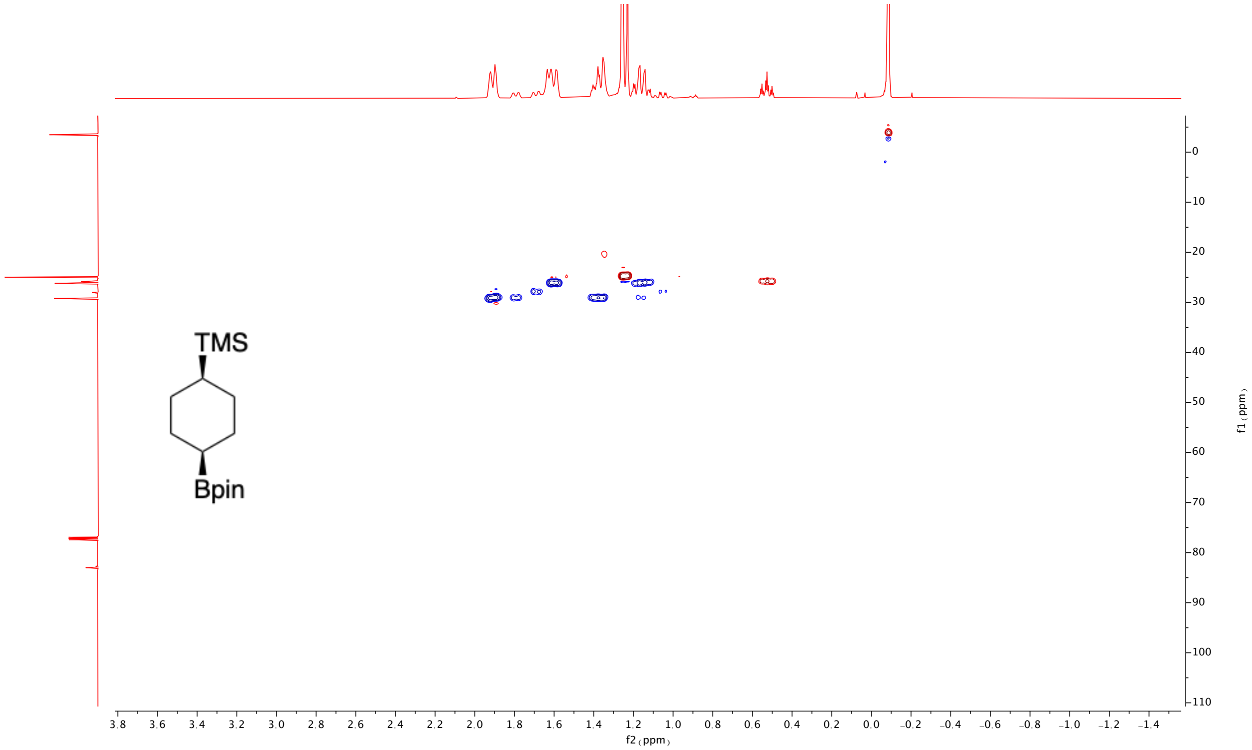

COSY NMR Trimethyl-4-(4,4,5,5-tetramethyl-1,3,2-dioxaborolan-2-yl)cyclohexyl)silane (2af)

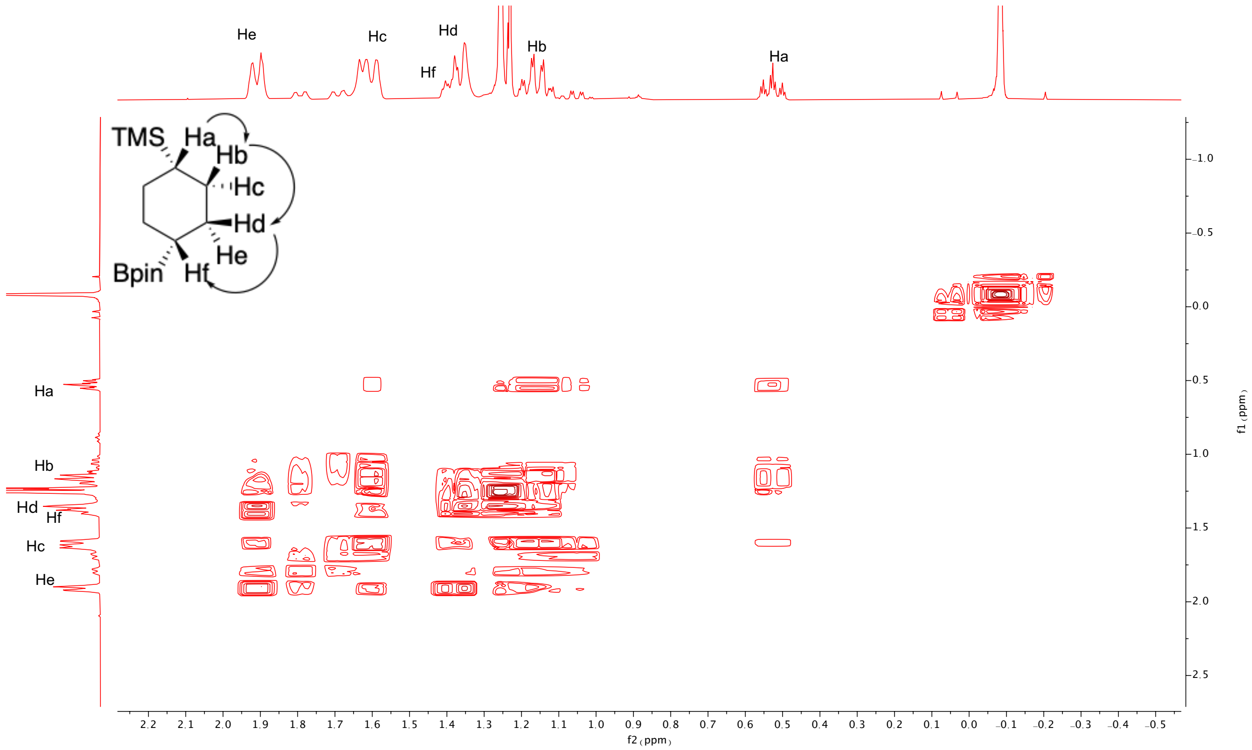

^{1}H NMR (500MHz, $CDCl_3$) of cis-trimethylsilyl-2-(4,4,5,5-tetramethyl-1,3,2-dioxaborolan-2-yl)cyclohexane (2ad)

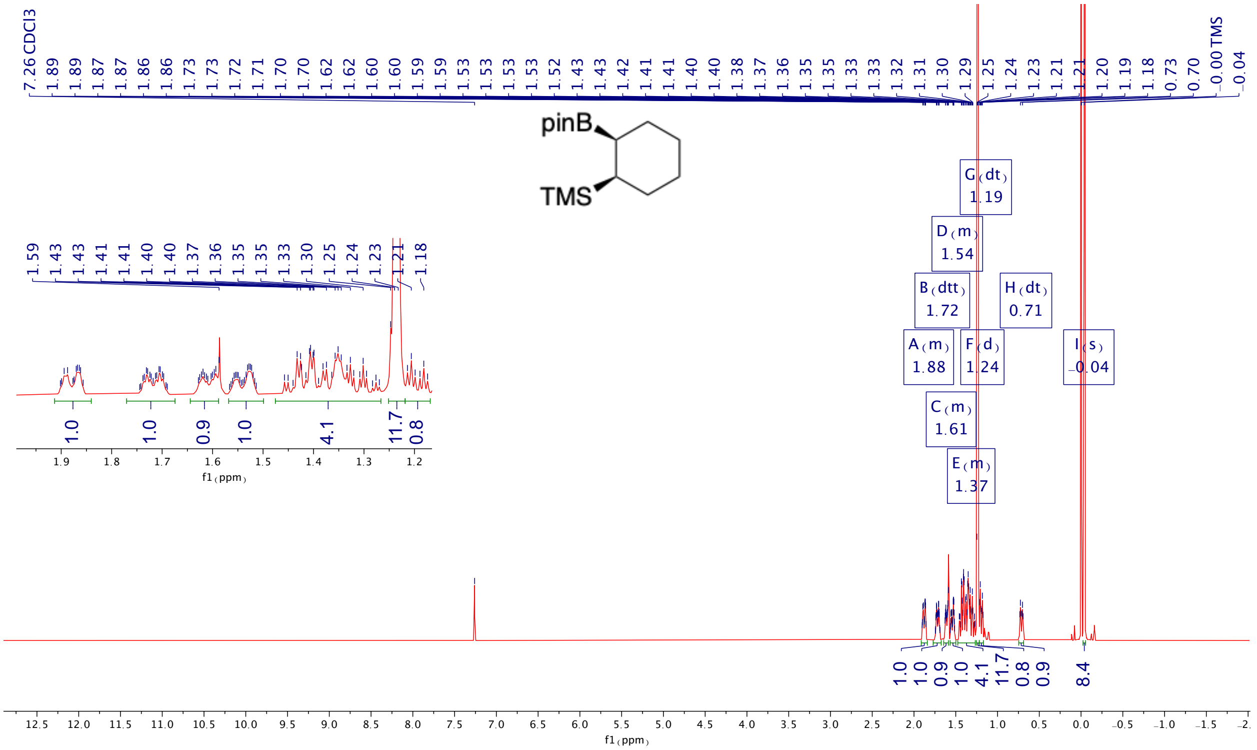

$^{13}C\{^1H\}$ (126 MHz, $CDCl_3$) of cis-trimethylsilyl-2-(4,4,5,5-tetramethyl-1,3,2-dioxaborolan-2-yl)cyclohexane (2ad)

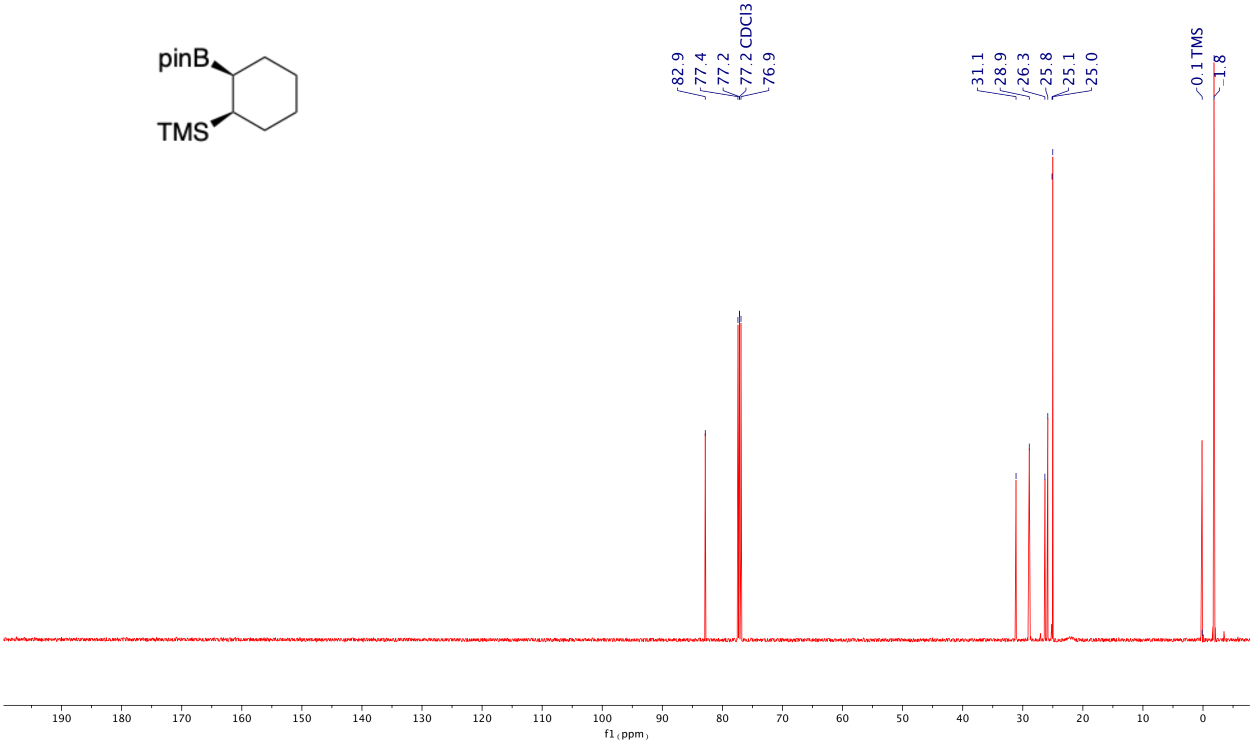

^{11}B NMR (160MHz, $CDCl_3$) of cis-trimethylsilyl-2-(4,4,5,5-tetramethyl-1,3,2-dioxaborolan-2-yl)cyclohexane (2ad)

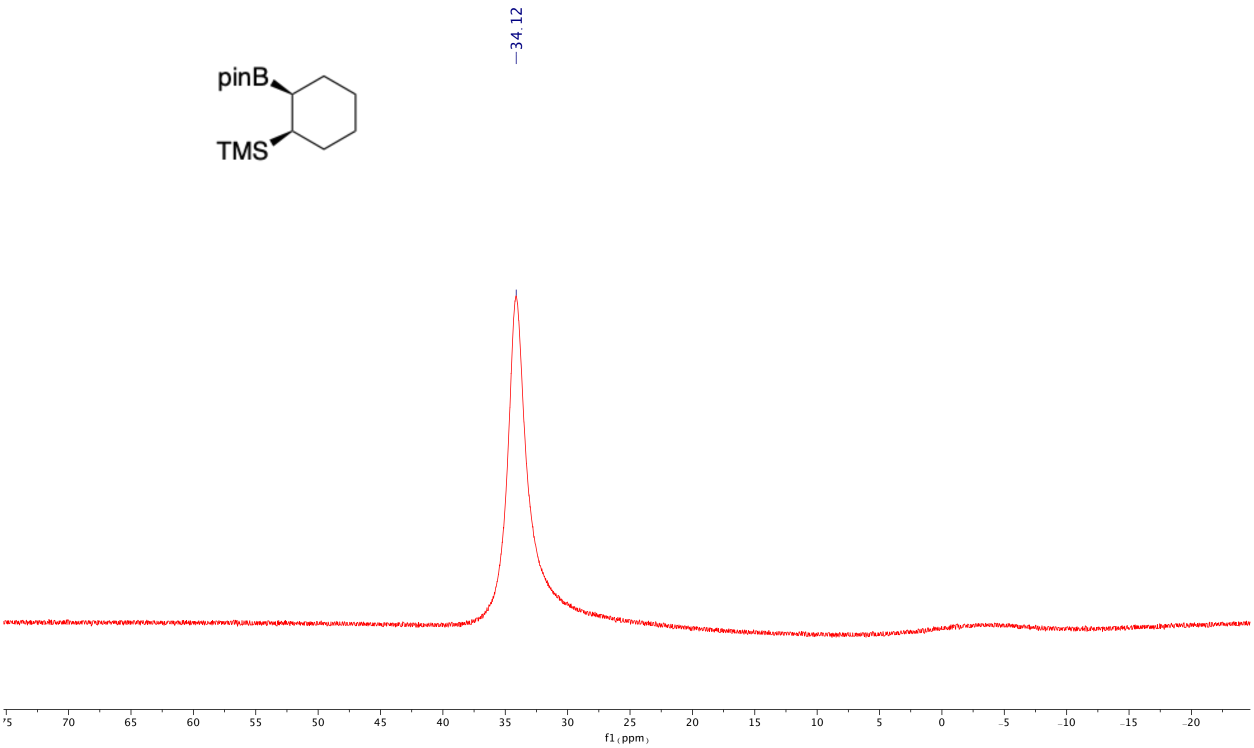

^{29}Si NMR (99MHz, $CDCl_3$) of cis-trimethylsilyl-2-(4,4,5,5-tetramethyl-1,3,2-dioxaborolan-2-yl)cyclohexane (2ad)

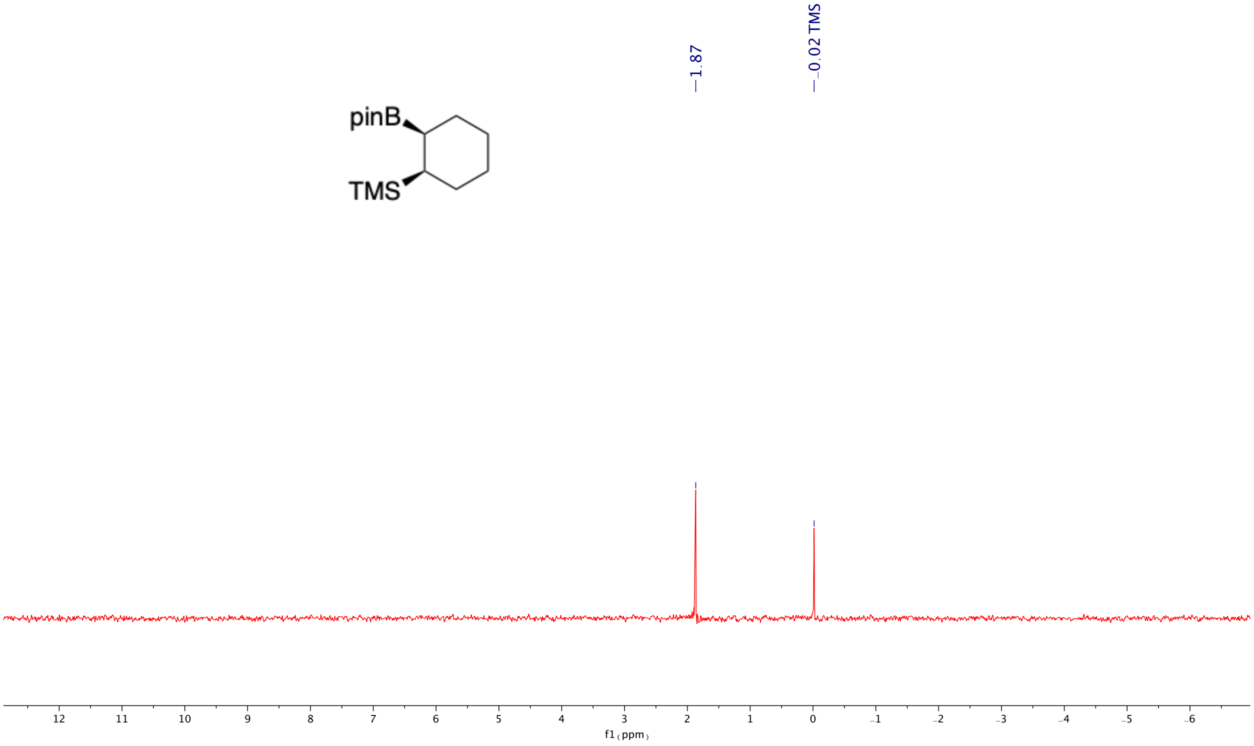

^{1}H NMR (500 MHz, CD_2Cl_2) of cis-trimethylsilyl-2-(4,4,5,5-tetramethyl-1,3,2-dioxaborolan-2-yl)cyclohexane (2ad)

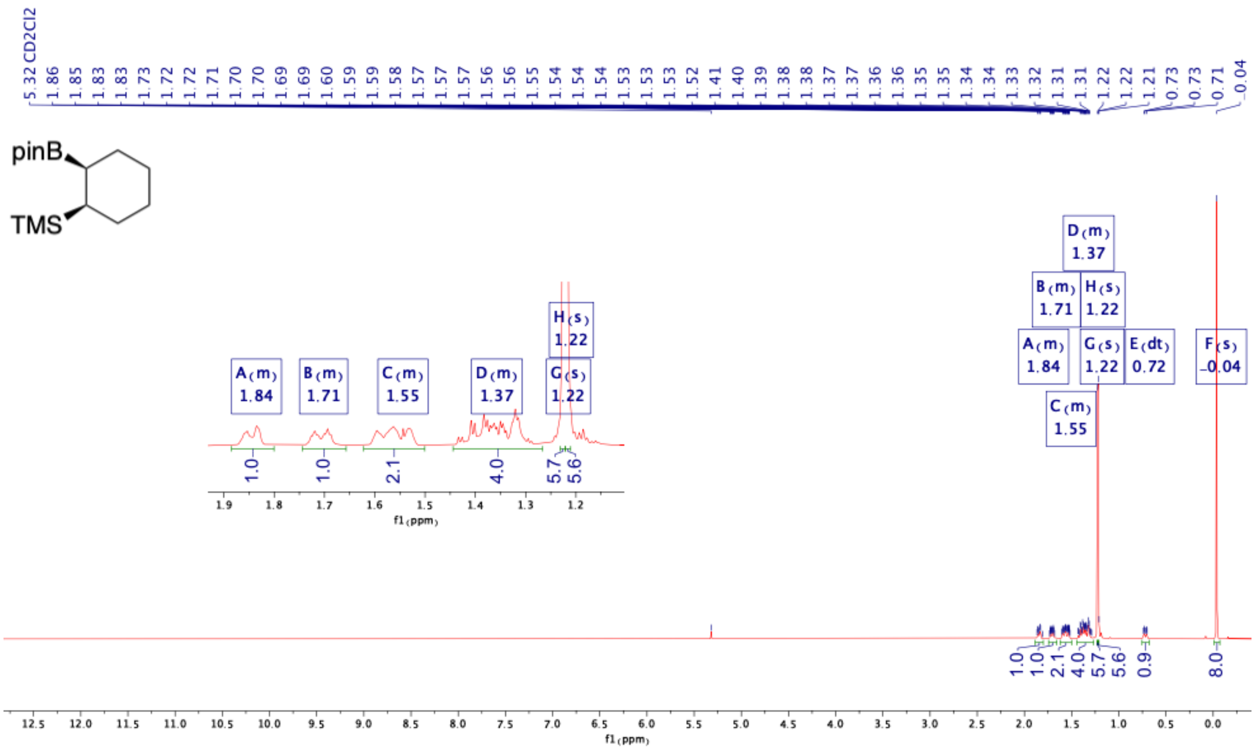

$^{13}C\{^{1}H\}$ **(126 MHz, CD_2Cl_2) of cis-trimethylsilyl-2-(4,4,5,5-tetramethyl-1,3,2-dioxaborolan-2-yl)cyclohexane (2ad)**

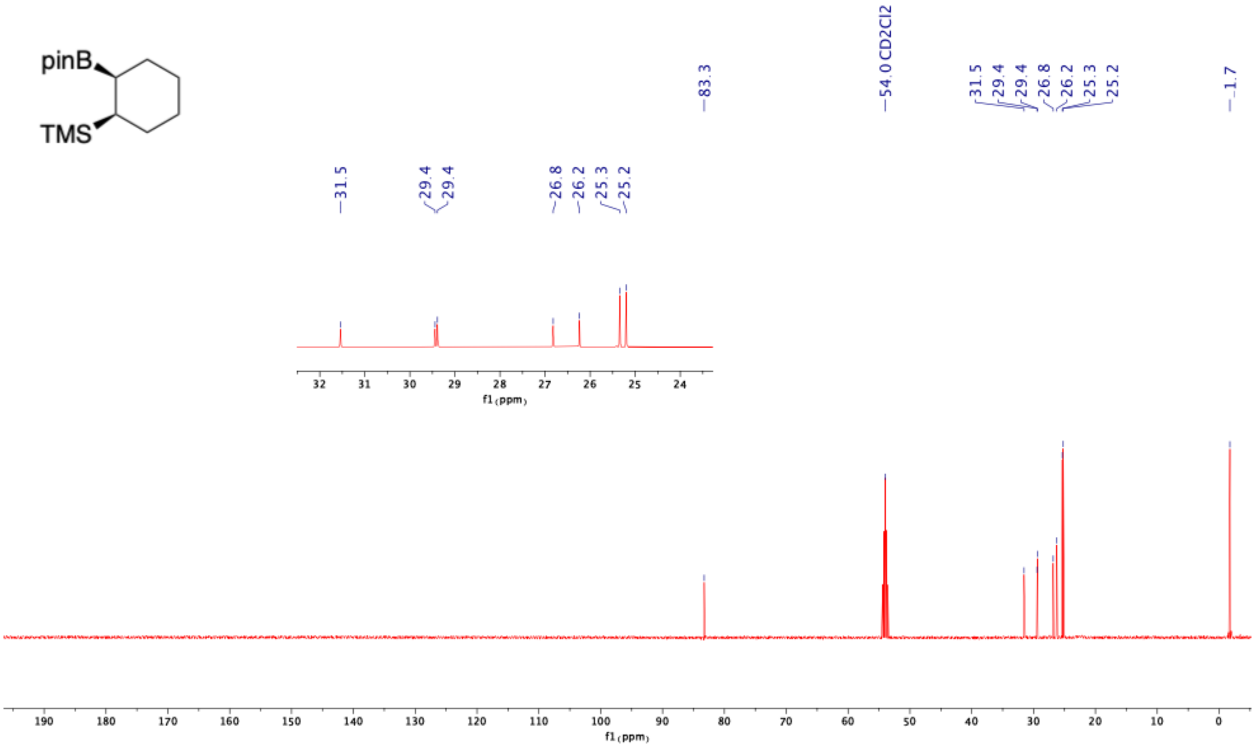

^{11}B **NMR (160MHz, CD_2Cl_2) of cis-trimethylsilyl-2-(4,4,5,5-tetramethyl-1,3,2-dioxaborolan-2-yl)cyclohexane (2ad)**

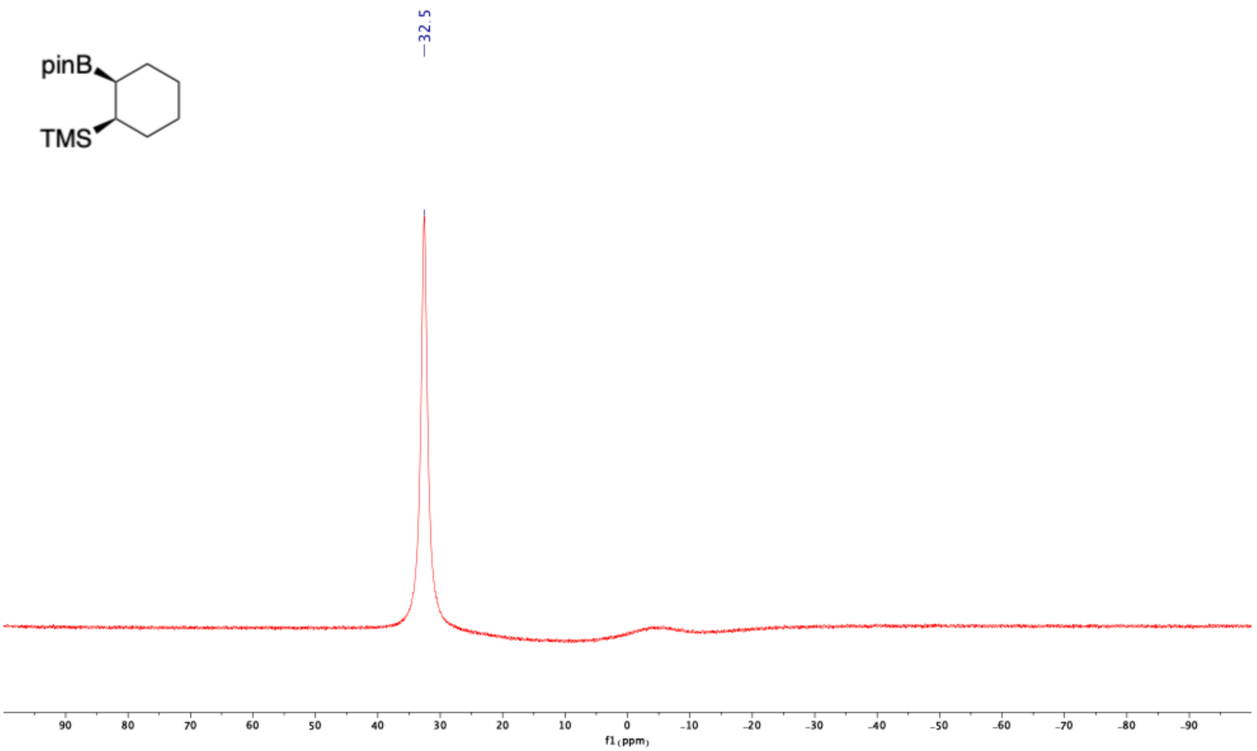

Supplement: RA-014-D4RA00491D-s001 [file RA-014-D4RA00491D-s001.pdf]
